# Global, regional, and national epidemiology of migraine and tension-type headache in youths and young adults aged 15–39 years from 1990 to 2019: findings from the global burden of disease study 2019

**DOI:** 10.1186/s10194-023-01659-1

**Published:** 2023-09-18

**Authors:** Xin-yu Li, Cheng-hao Yang, Jia-jie Lv, Hui Liu, Lu-yu Zhang, Min-yi Yin, Zhi-lin Guo, Ru-hong Zhang

**Affiliations:** 1 Department of Plastic and Reconstructive Surgery, Shanghai Ninth People’s Hospital, Shanghai JiaoTong University School of Medicine, Huangpu District, No.639 Zhizaoju Road, Shanghai, 200011 China; 2 Department of Neurosurgery, Shanghai Ninth People’s Hospital, Shanghai Jiao Tong University, Shanghai, People’s Republic of China; 3 Department of Vascular Surgery, School of Medicine, Shanghai Putuo People’s Hospital, Tongji University, Shanghai, China; 4 Department of Vascular Surgery, Shanghai Ninth People’s Hospital, Shanghai Jiao Tong University, Shanghai, China; 5 Department of Emergency, Shanghai Jiao Tong University Affiliated Sixth People’s Hospital, Shanghai, 200060 China; 6 The Department of Urology, Shangqiu First People’s Hospital, Shangqiu, China

**Keywords:** Global burden of disease, Migraine, Tension-type headache, Disability-adjusted life years, GBD, SDI

## Abstract

**Background:**

In recent years, headache disorders have garnered significant attention as a pressing global health issue. This concern is especially pronounced in low- to middle-income countries and exhibits a notable increase in prevalence among adolescents and young adults. Such a surge in these disorders has invariably diminished the quality of life for affected individuals. Despite its global impact, comprehensive studies exploring the ramifications of headache disorders in the younger population remain scant. Our study endeavored to quantify the global prevalence of headache disorders in individuals between the ages of 15 and 39, over a three-decade span from 1990 to 2019.

**Methods:**

Our study, conducted from 1990 to 2019, evaluated the impact of headache disorders, specifically migraines and tension-type headaches (TTH), in 204 different countries and territories. This comprehensive assessment included a detailed analysis of incidence rates, prevalence, and disability-adjusted life-years (DALYs) across various demographics such as age, gender, year, geographical location, and Socio-demographic Index (SDI).

**Results:**

In 2019, there were an estimated 581,761,847.2 migraine cases globally (95% UI: 488,309,998.1 to 696,291,713.7), marking a 16% increase from 1990. Concurrently, TTH cases numbered at 964,808,567.1 (95% UI: 809,582,531.8 to 1,155,235,337.2), reflecting a 37% rise since 1990. South Asia reported the highest migraine prevalence with 154,490,169.8 cases (95% UI: 130,296,054.6 to 182,464,065.6). High SDI regions exhibited the most substantial migraine prevalence rates both in 1990 (22,429 per 100,000 population) and 2019 (22,606 per 100,000 population). Among the five SDI classifications, the middle SDI region recorded the highest tally of TTH cases in both 1990 (210,136,691.6 cases) and 2019 (287,577,250 cases). Over the past 30 years, East Asia experienced the most pronounced surge in the number of migraine cases. On the whole, there was a discernible positive correlation between the disease burden of migraine and TTH and the SDI.

**Conclusion:**

Migraine and TTH represent formidable challenges in global health. The intensity of their impact exhibits marked disparities across nations and is distinctly elevated among women, individuals within the 30–39 age bracket, and populations characterized by a high SDI. The results of our research emphasize the imperative of assimilating migraine and TTH management into contemporary healthcare paradigms. Such strategic integration holds the potential to amplify public cognizance regarding pertinent risk factors and the spectrum of therapeutic interventions at hand.

**Supplementary Information:**

The online version contains supplementary material available at 10.1186/s10194-023-01659-1.

## Introduction

Primary headaches, the most prevalent type of headache, impose a significant economic burden [1–3]. These disorders can affect individuals across all age groups, but they typically peak in adult populations [3]. In 2019, headache disorders were the 14th leading cause of Disability Adjusted Life Years (DALYs) globally, affecting both genders and all age groups [4]. Notably, they were outpaced by seven other non-communicable disorders. In a closer examination of the female demographic, headache disorders secured the tenth rank, edging out depressive disorders which held the 11th position. This observation was particularly stark among young adult females aged 15 to 49 years, where headache disorders trailed only behind gynecological diseases [4]. The most common forms of primary headaches are migraines and tension-type headaches (TTH) [2, 3, 5]. Migraine is a recurring disorder that primarily causes moderate to severe headaches. These headaches are often accompanied by reversible neurological and systemic symptoms [6]. Common symptoms encompass photophobia, phonophobia, cutaneous allodynia, and gastrointestinal disturbances. Notably, the most prevalent triggers for migraines include alcohol, coffee, stress, and fatigue [7]. TTH is a prevalent neurological disorder. It is commonly observed not only in the general population but also among patients attending hospital outpatient clinics [8]. TTH is typified by pain of mild to moderate intensity, frequently likened to a tight band encircling the head. This bilateral headache distinctly differs from migraines, which are usually accompanied by pronounced neurological symptoms. For TTH patients, various strategies can be employed to alleviate discomfort. These encompass ensuring adequate sleep, taking suitable medication, maintaining proper posture, and employing massages [9].

Despite extensive research into the global and regional disease burden, there has been a noticeable lack of emphasis on the effects of headache disorders in adolescents and young adults aged 15–39 years. This gap is particularly evident in low- and middle-income countries, where research, education, and clinical services for migraine and TTH are significantly lacking. Even in wealthier nations, a substantial number of patients suffering from migraines or TTH either do not have access to suitable treatment or fail to receive it [10].

It is imperative to consistently re-evaluate the Global Burden of Disease (GBD) database, specifically regarding migraines and TTH in young adults, to mitigate long-term complications linked with these conditions. Remarkably, as of now, no global reports comprehensively delve into the epidemiology of headache disorders in youths and young adults using the 2019 datasets. Addressing this lacuna, our study leverages the GBD database, examining the incidence, prevalence, and DALYs trends of headache disorders in this demographic from 1990 to 2019. Furthermore, we project the future disease burden. Our objective is to offer a nuanced understanding of the global distribution of migraines and TTH, potentially shaping subsequent public health strategies.

## Methods

We sourced our data from the GBD 2019, which is available at http://ghdx.healthdata.org/gbd-results-tool. This exhaustive study gathered data on 369 diseases and injuries, in addition to 87 risk factors, spanning 204 countries and territories [11]. Data for the GBD was amassed from various sources, including censuses, surveys, vital statistics, and diverse health repositories. Three sophisticated methods-the Cause of Death Ensemble model, spatiotemporal Gaussian process regression, and the Bayesian meta-regression tool, DisMod-MR 2.1—are employed by the GBD model to synthesize this vast amount of data [4]. This blend ensures robust and accurate estimations of disease burdens. For a focused overview of migraines and TTH based on age, we categorized patients into five age brackets: 15–19, 20–24, 25–29, 30–34, and 35–39 years. Linear regression was then used to calculate the mean estimated annual percentage changes (EAPCs). Our study adhered to the Strengthening the Reporting of Observational Studies in Epidemiology guidelines for reporting. The cross-sectional nature of our study was approved by the ethical board of the Shanghai Ninth People's Hospital, which granted a waiver of informed consent, given that the study solely involved data analysis and did not contain any personal identifying information.

The GBD 2019 list of causes includes migraines, which are identified by specific codes from the International Classification of Disease [4]. A migraine is definitively diagnosed if a patient's symptoms meet all five major diagnostic criteria as proposed by the International Classification of Headache Disorders, 3rd edition (ICHD-3 [12]. Similarly, the diagnostic process for TTH follows the same procedure. A TTH is definitively diagnosed if a patient's symptoms meet all five major diagnostic criteria as outlined by the ICHD-3. In our recent study, we used the codes G43-G43.919, G44.2-G44.229, and G44.4-G44.41 from the International Classification of Diseases, 10th revision (ICD-10) to represent migraines and TTH [12].

### Sociodemographic index

The Sociodemographic Index (SDI) serves as an indicator of a country's or region's developmental status [13]. The SDI was developed using the geometric mean of three indicators: per capita income, average years of education among those aged 15 and older, and total fertility rate [14]. In the GBD 2019, countries and territories were stratified into five SDI categories: low (< 0.46), low-middle (0.46–0.60), middle (0.61–0.69), high-middle (0.70–0.81), and high (> 0.81) SDI. It should be underscored that a diminished SDI value indicates a reduced level of societal development [15].

### Statistical analysis

In the present study, we investigated the influence of migraines and TTH on health outcomes. To assess the magnitude of this impact, we employed multiple metrics, including incidence, prevalence, DALYs, each with their respective rates. DALYs, a comprehensive measure of disease burden, were calculated by combining the years of life lost due to premature death and years lived with disability. The disease burden was estimated and presented with 95% uncertainty intervals (UI) in this study. For a thorough comprehension of the methodology employed, readers are directed to the pertinent literature. In light of the diverse age distributions and populations represented in the GBD dataset, it is imperative to adjust for variations in age structures. Accordingly, the age-standardized rate (ASR) was computed per 100,000 individuals utilizing the subsequent formula [16]:

$$\mathrm{ASR}=\frac{\sum_{i=1}^Aa_iw_{i,}}{\sum_{i=1}^Aw_i}\times100,00$$

(${a}_{ i}$: the age-specific rate in *i*^th^ the age group; w: the number of people in the corresponding *i*.^th^ age group among the standard population; *A*: the number of age groups)

To examine the temporal patterns of incidence, mortality, and DALYs, we calculated the EAPC rates. The EAPC serves as a prevalent metric in epidemiological studies to ascertain temporal evolutions in ASRs of diseases. The coefficient, denoted as $\upbeta$, is derived from the natural logarithm of the ASRs. Herein, y represents ln(ASR) while.

x corresponds to the calendar years. The EAPC, accompanied by its 95% confidence interval (CI), was determined utilizing the ensuing linear regression model [16]:

$$\mathrm y=\mathrm\alpha+\mathrm\beta\mathrm x+\mathrm\varepsilon$$

$$\mathrm{EAPC}=100\ast(\exp\left(\mathrm\beta\right)-1$$

The trend of the ASR can be discerned by analyzing the EAPC and its corresponding 95% CI. If the EAPC value and the lower limit of the 95% CI are both positive, this indicates an upward trend in the ASR. Conversely, if both the EAPC value and the upper limit of the 95% CI are negative, this suggests a downward trend in the ASR [16]. To predict the future disease burden from 1990 to 2045, we utilized a log-linear age-period-cohort model. This model restricts linear trend projection and curbs exponential growth, rendering it suitable for fitting recent trends. We implemented the model in R using the NORDPRED package. To explore the factors influencing the changes of disease burden, the relationships between ASRs and SDI were calculated globally and in 21 geographic regions using Pearson’s correlation analysis from 1990 to 2019. Analyses and graphical representations were executed using the R statistical software (version 4.2.2). A two-tailed *P*-value below 0.05 was deemed statistically significant.

## Results

### Global level

#### Prevalence

**Fig. 1:**
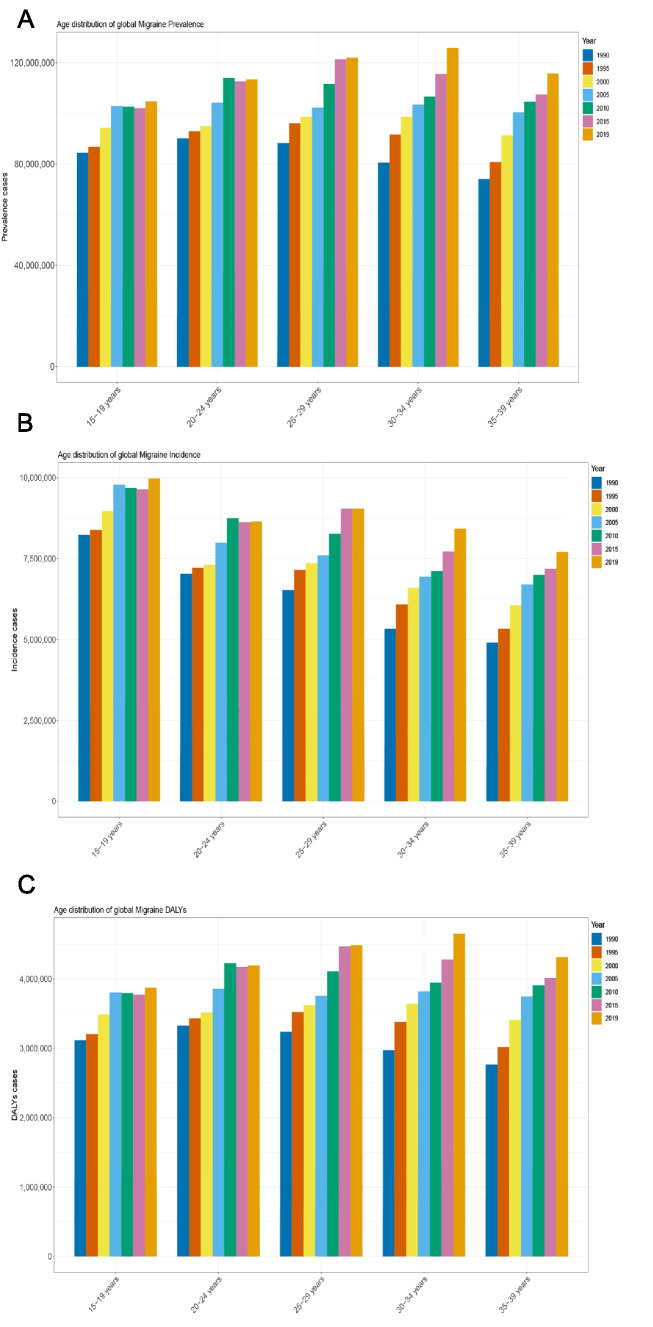
Trends in migraine prevalence, incidence and Disability-Adjusted Life-Years (DALYs) From 1990 to 2019. **A** Trends in prevalence cases. **B** Trends in incidence cases. **C** Trends in DALYs cases

The global prevalence of migraine was estimated at 581,761,847.2 cases (95% UI: 488,309,998.1 to 696,291,713.7) in 2019, representing a 16% increase since 1990 (417,624,741.1 cases, 95% UI: 349,971,811.1 to 499,587,739) (Table S1). However, the global age-standardized prevalence rate (ASPR) of migraine remained relatively constant between 1990 (19,039 per 100,000 population) and 2019 (19,602 per 100,000 population), with an EAPC of 0.07 (95% CI: 0.06 to 0.09). The reported prevalence of migraine increased across all age groups from 1990 to 2019 (Fig. 1A). Concurrently, ASPR demonstrated increases across all age strata over the same interval (Fig S1A). Upon examination of ASPR by sex, rates were consistently higher among females compared to males at the global level, with the greatest disparity observed in the 35–39 year age group (Fig S2A).

The global prevalence of TTH was estimated at 964,808,567.1 cases (95% UI: 809,582,531.8 to 1,155,235,337.2) in 2019, representing a 37% increase since 1990 (702,507,085.3 cases, 95% UI: 588,427,399.4 to 839,701,792.4) (Table S2). However, the global ASPR of TTH increased only slightly between 1990 (32,027 per 100,000 population) and 2019 (32,509 per 100,000 population), with an EAPC of 0.04 (95% CI: 0.02 to 0.06) (Table S2). In terms of ASPR, except for a slight decrease in the 30–34 age group and 35–39 age group, the other age groups show an upward trend, with the largest increase in the 15–19 age group (Fig S3A). The ratio of prevalence of TTH between men and women was the highest in the 35–39 age group and the lowest in the 20–24 age group (Fig S4A).

#### Incidence

In 2019, the global incidence of migraine was estimated to be 43,792,977.8 cases (95% UI: 35,587,917.8 to 53,516,837.4), representing a 36.7% increase since 1990, as shown in Table 1. The age-standardized incidence rate (ASIR) of migraine remained relatively stable worldwide between 1990 and 2019, with rates of 1,460 and 1,476 per 100,000 population, respectively (Table 1). The number of new cases of migraine has risen markedly across all age groups, with the highest incidence occurring in the 15–19 age group (Fig. 1B). However, ASIR increased in all age groups except for the 25–29 age group, with the highest ASIR observed in the 15–19 age group (Fig S1B). The global ASIR trends mirrored the ASPR trends across all age groups, as illustrated in Fig S2B.

**Table 1:** Incidence of migraine between 1990 and 2019 in 15 to 39 years at the global and regional level

	1990		2019		
Location	Number_95%UI	ASR	Number_95%UI	ASR	EAPC_95%CI
Global	32,034,316.3 (26,003,892.1–39,067,354.9)	1460.4 (1185.5–1781.1)	43,792,977.8 (35,587,917.8–53,516,837.4)	1475.6 (1199.1–1803.2)	0.02 (0–0.04)
High SDI	5,338,023.7 (4,374,182–6,434,531)	1652.3 (1354–1991.7)	5,497,608.9 (4,508,388.4–6,597,680)	1659.4 (1360.8–1991.4)	0.03 (0.01–0.04)
High-middle SDI	6,796,703.9 (5,544,902.1–8,272,209)	1405.7 (1146.8–1710.9)	7,348,321.9 (6,000,457.8–8,897,769)	1422.1 (1161.3–1722)	0.02 (0.01–0.04)
Middle SDI	10,617,452.4 (8,628,237.2–12,949,911.3)	1422.1 (1155.7–1734.5)	13,840,858.6 (11,304,213.5–16,825,011.5)	1480.2 (1208.9–1799.3)	0.12 (0.11–0.14)
Low-middle SDI	6,626,608.6 (5,321,565.6–8,146,071.4)	1488 (1195–1829.2)	10,993,656.1 (8,857,396.5–13,472,817.1)	1495.1 (1204.6–1832.3)	-0.02 (-0.05–0)
Low SDI	2,638,058.9 (2,092,433.9–3,265,000.6)	1361.1 (1079.6–1684.6)	6,087,081 (4,844,316–7,546,180.1)	1359.6 (1082–1685.5)	-0.02 (-0.03–0.01)
Andean Latin America	140,794.5 (110,715.4–177,891.8)	911.1 (716.4–1151.1)	250,654.8 (195,347.7–318,392.6)	976.1 (760.7–1239.9)	0.28 (0.21–0.35)
Australasia	126,193.4 (100,668.4–155,865.6)	1547.5 (1234.5–1911.4)	148,906.3 (118,794.2–184,410.1)	1532.4 (1222.5–1897.7)	-0.01 (-0.02–0)
Caribbean	195,178.7 (150,241.8–247,359.9)	1315.6 (1012.7–1667.3)	236,579.5 (183,543.5–297,172.6)	1305 (1012.5–1639.3)	-0.02 (-0.02–0.02)
Central Asia	410,664.6 (319,157.7–515,387.1)	1442.3 (1120.9–1810.1)	540,838 (423,721.9–670,386.9)	1427.6 (1118.4–1769.5)	-0.03 (-0.04–0.02)
Central Europe	657,962.1 (525,039.3–813,105)	1429.8 (1140.9–1766.9)	507,014.1 (407,377.3–621,617.7)	1423.7 (1143.9–1745.5)	-0.03 (-0.03–0.02)
Central Latin America	917,134.9 (729,112.9–1,134,070.6)	1344.6 (1068.9–1662.6)	1,358,807.8 (1,079,766.5–1,674,559.2)	1345.6 (1069.2–1658.2)	0.01 (0.01–0.02)
Central Sub-Saharan Africa	280,024.7 (217,207.9–354,929)	1348.8 (1046.2–1709.6)	698,231.1 (542,104.5–883,713.2)	1347.9 (1046.5–1706)	0 (0–0)
East Asia	6,948,514.6 (5,696,296.8–8,390,731.3)	1225.5 (1004.7–1479.9)	6,676,434.8 (5,513,119.9–8,057,427.9)	1294.6 (1069.1–1562.4)	0.18 (0.14–0.22)
Eastern Europe	1,199,093.2 (987,888.6–1,445,658.3)	1397.7 (1151.5–1685.1)	952,226.5 (786,424.6–1,147,708.4)	1387.5 (1145.9–1672.3)	0 (-0.03–0.02)
Eastern Sub-Saharan Africa	708,754.9 (556,813.1–881,003.6)	1005.3 (789.8–1249.6)	1,684,934.7 (1,325,348.8–2,094,604.8)	1010.4 (794.8–1256.1)	0.03 (0.03–0.04)
High-income Asia Pacific	842,473.8 (685,230.1–1,023,251.2)	1247.2 (1014.4–1514.9)	637,199.3 (519,999–774,581.5)	1212.7 (989.7–1474.2)	-0.1 (-0.15–0.05)
High-income North America	2,102,636.3 (1,753,529.3–2,494,778.7)	1859.5 (1550.8–2206.3)	2,268,051.2 (1,883,391.6–2,686,545.6)	1866.6 (1550–2211)	0.03 (0.01–0.05)
North Africa and Middle East	2,055,917.6 (1,595,107.7–2,588,083.4)	1514.1 (1174.7–1906)	3,849,979.4 (3,062,926.6–4,800,263.6)	1488.7 (1184.3–1856.1)	-0.07 (-0.07–0.06)
Oceania	39,763.1 (30,663.9–49,861.2)	1510.5 (1164.8–1894.1)	82,132.5 (64,005.4–102,732.4)	1509.2 (1176.1–1887.8)	0 (0–0)
South Asia	6,745,521.9 (5,476,441.3–8,212,820)	1561.6 (1267.8–1901.3)	12,041,286 (9,803,921.6–14,652,737.7)	1566.1 (1275.1–1905.8)	-0.06 (-0.09–0.03)
Southeast Asia	3,409,210.2 (2,732,393.7–4,158,424.4)	1732.2 (1388.4–2112.9)	4,628,188.8 (3,754,584.7–5,628,502.1)	1704.1 (1382.4–2072.4)	-0.06 (-0.07–0.05)
Southern Latin America	242,108.9 (187,771.5–300,933.7)	1268.5 (983.8–1576.8)	329,418.8 (255,711.3–409,744.1)	1295.3 (1005.5–1611.1)	0.12 (0.09–0.15)
Southern Sub-Saharan Africa	310,808 (249,547.7–380,525.1)	1417.5 (1138.1–1735.5)	469,033 (381,645.4–572,176.4)	1392.5 (1133.1–1698.8)	-0.06 (-0.07–0.06)
Tropical Latin America	908,124.7 (730,125.4–1,112,829.3)	1412 (1135.2–1730.2)	1,187,320.3 (963,308–1449828.3)	1332.6 (1081.2–1627.3)	-0.28 (-0.38–0.19)
Western Europe	2,694,729.1 (2,179,101.3–3,271,735.4)	1870.5 (1512.6–2271)	2,460,063.9 (1,989,628.1–2,969,979.4)	1877.3 (1518.3–2266.5)	0.05 (0.03–0.07)
Western Sub-Saharan Africa	1,098,706.9 (870,131–1364069.3)	1545.2 (1223.7–1918.4)	2,785,677.2 (2,195,344.6–3,456,832.8)	1555 (1225.4–1929.6)	0.01 (0.01–0.02)

Abbreviations: *EAPC* Estimated annual percentage change, *SDI* Sociodemographic Index, *UI* Uncertainty interval

Globally, the estimated incidence of TTH was 329,572,706.8 cases (95% UI: 267,549,826.6 to 393,981,605.7) in 2019, a 37.4% increase from 239,729,661.3 cases (95% UI: 195,868,914.9 to 287,251,453.3) in 1990 (Table 2). However, the global ASIR of TTH only slightly increased from 10,929 per 100,000 population in 1990 to 11,105 per 100,000 population in 2019, with EAPC of 0.05 (95% CI: 0.03 to 0.06). Over the past three decades, the number of TTH incidence cases has significantly increased in all age groups, while the degree of change of ASIR in all age groups remains relatively stable, as indicated in Fig S3B. The male-to-female incidence ratio of TTH was highest in the 30–34 age group and lowest in the 15–19 age group (Fig S4B).

**Table 2:** Incidence of tension-type headache between 1990 and 2019 in 15 to 39 years at the global and regional level

	1990		2019		
Location	Number	ASR	Number	ASR	EAPC_95%CI
Global	239,729,661.3 (195,868,914.9–287,251,453.3)	10,929.1 (8929.5–13,095.6)	329,572,706.8 (267,549,826.6–393,981,605.7)	11,104.7 (9014.9–13,274.9)	0.05 (0.03–0.06)
High SDI	44,628,145.8 (36,137,436.8–53,412,593)	13,814 (11,185.8–16,533.1)	45,109,288.5 (36,512,867–54,000,246.1)	13,615.6 (11,020.9–16,299.2)	-0.08 (-0.09–0.07)
High-middle SDI	53,068,497.7 (43,604,762.4–63,255,771.3)	10,975.6 (9018.3–13,082.5)	57,082,218.3 (46,505,090.9–68,433,467.9)	11,047 (9000.1–13,243.8)	0.02 (-0.01–0.04)
Middle SDI	72,642,170.5 (59,672,321.1–87,682,954.1)	9729.8 (7992.6–11,744.4)	98,305,842.8 (79,715,357.4–118,093,819.3)	10,513 (8524.9–12,629.1)	0.28 (0.26–0.29)
Low-middle SDI	48,656,105.4 (39,811,897.1–58,516,686)	10,925.7 (8939.7–13,139.9)	81,958,855.6 (66,846,042–98332852.8)	11,146.4 (9091.1–13,373.3)	0.04 (0.03–0.06)
Low SDI	20,608,804.9 (16,753,057.2–24,815,223.5)	10,633.3 (8643.9–12,803.7)	46,932,112.4 (38,103,091.8–56,604,800.7)	10,482.7 (8510.6–12,643.1)	-0.07 (-0.09–0.06)
Andean Latin America	1,314,085.1 (1,056,395–1594178.6)	8503.4 (6835.9–10,315.9)	2,247,752 (1,828,936.8–2,729,540.8)	8753.3 (7122.3–10,629.5)	0.13 (0.11–0.15)
Australasia	997,847.3 (809,359.5–1,199,511.9)	12,236.7 (9925.2–14,709.7)	1,193,287 (959,727.3–1,434,531.9)	12,279.9 (9876.4–14,762.5)	0 (-0.01–0)
Caribbean	1,537,569.3 (1,244,281.9–1,853,602.5)	10,363.8 (8386.9–12,494)	1,885,680.8 (1,525,390.9–2,258,477.2)	10,401.8 (8414.4–12,458.3)	0.01 (0–0.01)
Central Asia	3,843,975 (3,127,657.6–4,612,757.7)	13,500.1 (10,984.4–16,200.1)	5,137,289.3 (4,152,077.3–6,156,349.4)	13,560.1 (10,959.6–16,249.9)	0.01 (0–0.02)
Central Europe	6,175,004.1 (5,011,563.3–7,400,129.2)	13,418.4 (10,890.2–16,080.6)	4,810,748.6 (3,880,641.8–5,754,574.2)	13,508.5 (10,896.7–16,158.7)	0.03 (0.03–0.04)
Central Latin America	7,355,078.3 (5,964,477.6–8,880,279.7)	10,782.8 (8744.2–13,018.8)	10,919,311.5 (8,899,286.4–13,041,697.4)	10,812.9 (8812.5–12,914.6)	0 (0–0.01)
Central Sub-Saharan Africa	2,190,670 (1,766,748.6–2,678,063.8)	10,551.9 (8510–12899.6)	5,471,231.1 (4,412,608.1–6,687,698.8)	10,562.3 (8518.6–12,910.7)	0 (0–0)
East Asia	43,616,660 (36,011,950.7–52,881,675)	7692.8 (6351.6–9326.9)	43,199,921.5 (34,624,993.7–52,353,148.8)	8376.9 (6714.2–10,151.9)	0.38 (0.3–0.45)
Eastern Europe	12,702,347.2 (10,285,493.9–15,108,741.4)	14,805.9 (11,988.8–17,610.8)	10,237,733.6 (8,200,770.4–12,224,870.3)	14,917.4 (11,949.3–17,812.9)	0.01 (0–0.03)
Eastern Sub-Saharan Africa	6,443,362.9 (5,225,204.2–7,809,726.4)	9139.3 (7411.5–11,077.4)	14,894,226.7 (12,094,618.1–18,078,505.2)	8931.7 (7252.8–10,841.2)	-0.11 (-0.13–0.09)
High-income Asia Pacific	9,006,221.3 (7,350,379.1–10,689,822.8)	13,333.2 (10,881.8–15,825.7)	7,092,111.2 (5,766,620.8–8,549,159.6)	13,497.9 (10,975.2–16,271)	0.05 (0.04–0.06)
High-income North America	17,366,641.3 (13,959,146.2–20,786,027.6)	15,358.7 (12,345.2–18,382.7)	18,434,902.7 (15,006,436–21976833.5)	15,171.7 (12,350.1–18,086.7)	-0.07 (-0.08–0.05)
North Africa and Middle East	13,588,051.8 (11,009,646–16433627.5)	10,007.2 (8108.3–12,102.9)	26,070,649.9 (21,025,583.9–31,637,848.3)	10,080.6 (8129.9–12,233.3)	0 (-0.01–0.01)
Oceania	257,851.6 (208,398.9–312,988.2)	9794.9 (7916.4–11,889.4)	536,407.8 (435,518.2–649,536)	9856.8 (8002.9–11,935.6)	0.02 (0.01–0.02)
South Asia	50,478,948 (41,381,424.9–60,912,766.5)	11,685.8 (9579.7–14,101.2)	90,138,800.5 (73,763,193–108,381,706.1)	11,723.6 (9593.8–14,096.3)	-0.05 (-0.08–0.02)
Southeast Asia	21,753,619.5 (17,635,541.6–26,115,630.7)	11,053.2 (8960.8–13,269.6)	30,365,389.5 (24,508,512.3–36,453,815.1)	11,180.4 (9023.9–13,422.2)	0.04 (0.03–0.04)
Southern Latin America	2,285,327.6 (1,856,687.2–2,735,719.2)	11,974.1 (9728.2–14,333.9)	3,069,986.4 (2,479,222.7–3,675,108.3)	12,071.2 (9748.3–14,450.5)	0.03 (0.03–0.04)
Southern Sub-Saharan Africa	2,446,098.3 (1,992,436.5–2,929,712.3)	11,156.2 (9087.1–13,361.9)	3,815,945 (3,083,405.4–4,594,034.8)	11,329.4 (9154.5–13,639.5)	0.05 (0.04–0.06)
Tropical Latin America	7,866,415.5 (6,380,831.9–9,464,492.9)	12,230.8 (9921–14,715.5)	10,906,868.1 (8,863,260.6–13,156,294.4)	12,241.9 (9948.1–14,766.6)	-0.02 (-0.04–0)
Western Europe	20,357,516.9 (16,555,699.2–24,474,327)	14,130.7 (11,491.8–16,988.3)	18,659,939.6 (15,108,379.1–22,418,249.8)	14,239.9 (11,529.6–17,108)	0.02 (0.01–0.02)
Western Sub-Saharan Africa	8,146,370.5 (6,581,974.6–9,810,011.9)	11,456.8 (9256.7–13,796.5)	20,484,524 (16,538,361.8–24,629,001.4)	11,434.5 (9231.7–13,748)	-0.02 (-0.02–0.01)

Abbreviations: *EAPC* Estimated annual percentage change, *SDI* Sociodemographic Index, *UI* Uncertainty interval, *TTH* Tension-type headaches

#### DALYs

In 2019, the number of DALYs attributed to migraine reached 21,529,090.4 (95% UI: 2,541,050.5 to 49,944,979.9), marking a 40% increase from the 1990 count of 15,424,064 (95% UI: 1,814,454.8 to 35,653,837.1) as shown in Table S3. Yet, the age-standardized DALY rate per 100,000 population witnessed a modest rise, shifting from 703.2 in 1990 to 725.4 in 2019. This was accompanied by an EAPC of 0.08 (95% CI: 0.07 to 0.1) as reflected in Table S3. Breaking it down by age groups, the 30–34 bracket bore the highest number of DALYs, with the 25–29 age group trailing right behind (Fig. 1C). Moreover, the age-standardized DALY rate positively correlated with age throughout all groups, with the peak rate being recorded in the 35–39 age cohort (Fig S1C). Notably, the male-to-female DALY ratio dwindled as age increased, indicating that migraines pose a higher burden on older women (Fig S2C).

From a global perspective, TTH-related DALYs saw a 38% surge over the span of three decades. Specifically, DALYs escalated from 1,446,380.2 (95% UI: 386,965.6–5,450,782.5) in 1990 to 1,992,931.2 (95% UI: 529,490.2–7,415,829.4) by 2019, as detailed in Table S3. Nonetheless, the age-standardized DALY rate for every 100,000 individuals experienced a marginal elevation, moving from 65.9 in 1990 to 67.2 in 2019. With an EAPC standing at 0.05 (95% CI: 0.03 to 0.06), it is evident that the worldwide disability burden attributed to tension headaches, when adjusted for population, witnessed only a slight change over this 30-year timeframe (Table S3). Over the years, DALYs linked to TTH have increased across all age brackets, with the age-standardized DALYs peaking in the 35–39 year cohort (Fig S3C). Intriguingly, the disparity in DALYs between genders was most pronounced in the 30–34 age group, which then began to taper off. Meanwhile, the smallest disparity was observed in the 20–24 age bracket (Fig S4C).

### SDI regional

#### Prevalence

The middle SDI region had the highest number of migraine cases among the 5 SDI regions in both 1990 (135,930,245.3 cases) and 2019 (184,019,869.2 cases) (Table S1). In terms of ASPR, the high SDI regions had the highest rates in both 1990 (22,429/100,000) and 2019 (22,606/100,000). However, it is worth noting that the middle SDI regions experienced the most significant increase in migraine prevalence over the past three decades. The number of cases rose from 135,930,245.3 in 1990 to 184,019,869.2 in 2019, with an EAPC of 0.24 (95% CI: 0.22–0.25). In all five SDI regions, the prevalence of males was consistently lower than that of females. Generally, this ratio further declined with advancing age (Fig S2A).

The middle SDI region recorded the highest number of TTH cases among the five SDI regions in both 1990 (210,136,691.6 cases) and 2019 (287,577,250 cases) (Table S2). This region experienced the most significant increase in TTH prevalence over the past three decades, with cases rising from 210,136,692 in 1990 to 287,577,250 in 2019 (EAPC = 0.32(95%CI:0.29–0.34)). On the other hand, the high SDI regions demonstrated the highest ASPR in both 1990 (42,102/100,000) and 2019 (41,280/100,000). Despite the substantial growth in TTH prevalence in the middle SDI regions, the high SDI areas showed minimal change (EAPC = -0.1; 95% CI: -0.12 to -0.08). However, these high SDI regions consistently maintained the highest ASPR throughout this period. Unlike migraine, the prevalence of TTH is greater in males than in females in the 30–39 age group in low − middle SDI and in the 25–39 age group in low SDI (Fig S4A).

#### Incidence

The middle SDI region recorded the highest number of migraine cases in both 1990 and 2019, with 10,617,452.4 and 13,840,858.6 cases respectively. This region also experienced the most significant growth, with EAPC of 0.12 (95% CI: 0.11–0.14). In contrast, the high SDI regions had the highest ASIR in both 1990 and 2019, with rates of 1652.3 and 1659.4 per 100,000 population respectively (Table 1). The high-middle SDI regions saw an increase in migraine incidence from 6,796,703.9 cases in 1990 to 7,348,321.9 cases in 2019 (Table 1). Concurrently, the ASIR in these regions also experienced a slight increase from 1405.7 to 1422.1 per 100,000 population. The low-middle SDI regions, on the other hand, experienced a significant increase in migraine incidence from 6,626,608.6 cases in 1990 to 10,993,656.1 cases in 2019. However, the ASIR in these regions remained relatively stable at around 1495.1 per 100,000 population. Lastly, the low SDI regions saw an increase in migraine incidence from 2,638,058.9 cases in 1990 to 6,087,081 cases in 2019. Despite this increase, the ASIR in these regions slightly declined from 1361.1 to 1359.6 per 100,000 population over the same period (Table 1). With the exception of high SDI areas, the ratio of male-to-female incidence decreased gradually with the increase of age in the remaining SDI areas, while the opposite trend was shown in the 15–34 age group in high SDI areas (Fig S2B).

The middle SDI region recorded the highest number of TTH incident cases among the five SDI regions in both 1990 and 2019 (Table 2). Specifically, the cases in this region surged from 72,642,170.5 in 1990 to 98,305,842.8 in 2019, marking the most significant growth in TTH incidence over the past three decades (EAPC = 0.28, 95% CI = 0.26–0.29). On the other hand, the high SDI regions had the highest ASIR in both 1990 (13,814/100,000) and 2019 (13,615.6/100,000) (Table 2). This decrease was mirrored in the ASIR, which fell from 13,814 to 13,616 per 100,000 between 1990 and 2019 (Table 2). The high-middle SDI regions also experienced an increase in the absolute number of TTH cases, from 53,068,497.7 in 1990 to 57,082,218.3 in 2019. However, the ASIR in these regions remained relatively stable at around 11,000 per 100,000 over the past three decades, fluctuating between 10,976 and 11,047 per 100,000 (Table 2). In the low-middle SDI regions, the number of cases significantly increased from 48,656,105.4 in 1990 to 81,958,855.6 in 2019. Yet, similar to the high-middle SDI regions, the ASIR in these areas remained relatively unchanged, hovering around 11,100 per 100,000 between 1990 and 2019 (Table 2). Except for high SDI and low SDI, the EAPC of other SDI areas is positive. In conclusion, while the total number of TTH incident cases increased across all SDI regions over the past three decades, the middle SDI region experienced the most substantial growth. The ASIR remained relatively stable across most regions, with the exception of a slight decline in the high SDI and low SDI regions, which consistently had the highest ASIR from 1990 to 2019. In contrast to migraine, the male-to-female ratio of TTH incidence increased with age, and the incidence of TTH in men was higher than that in women in low-middle SDI and low SDI (Fig S4B).

#### DALYs

Over the past three decades, the middle SDI region has experienced the most significant relative growth in DALYs due to migraines. Specifically, total DALYs in this region surged by 35.5%, from 5,070,200.1 in 1990 to 6,873,547.7 in 2019 (Table S3). In contrast, the high SDI regions, while maintaining the highest age-standardized DALYs among the five SDI regions, only saw a slight increase in total DALYs. The figures rose from 2,638,694.3 in 1990 to 2,734,053.3 in 2019 (Table S3). The age-standardized DALYs in most other SDI regions remained relatively stable from 1990 to 2019. The high-middle SDI regions, however, saw a slight increase in DALYs, from 3,332,280.1 in 1990 to 3,745,745.3 in 2019. The age-standardized DALYs in these regions also rose marginally, from 689.2 to 724.9 per 100,000. The low SDI regions also experienced a significant increase in DALYs, from 1,238,411.3 in 1990 to 2,873,183.4 in 2019. However, the ratio of age-standardized DALYs for both sexes was less than 1 in all five SDI regions, indicating a general trend of decreasing ratios with age (Fig S2C).

In 2019, regions with middle SDI recorded the highest number of DALYs associated with TTH, amounting to 606,396.7 (95% UI, 163,865.3–2,242,322.2). On the other hand, the low-middle SDI regions saw the most significant reduction in age-standardized DALYs (EAPC = -0.03, 95%CI = -0.05 to -0.02). With the exception of high SDI, the ratio of male-to-female age-standardized DALYs in the rest of the SDI regions shows a U-shaped trend (Fig S4C).

### Geographic regions level

#### Prevalence

The study spanned 21 geographic regions. South Asia exhibited the highest migraine prevalence at 154,490,169.8 (95% UI, 130,296,054.6–182,464,065.6). In stark contrast, Oceania recorded the lowest at 1,022,009.1 (95% UI, 830,207.7–1,243,315.4, Table S1). Over the last thirty years, East Asia has seen the steepest rise in migraine prevalence, reflecting an EAPC of 0.29 (95% CI, 0.26–0.33). On the opposite end, South Asia experienced the most significant decline, with an EAPC of -0.08 (95% CI, -0.1 to -0.05). The ASPR for migraines peaked in Western Europe at 26,919.8 per 100,000 population (95% UI, 22,527–32,252.7), while Eastern Sub-Saharan Africa posted the lowest ASPR at 356.38 per 100,000 population (95% UI, 230.01–519.3) (Table S1). As illustrated in FigS5A, a positive correlation exists between the ASPR of migraines and the SDI (*R* = 0.28, *P* < 0.001). Eight regions, including but not limited to Western Europe, Tropical Latin America, and High-income North America, surpassed the global mean prevalence (19,602), whereas 13 regions, including Eastern Europe and Central Latin America, stayed beneath this threshold. In terms of TTH, 2019 saw South Asia at the forefront with 260,079,112.3 cases (95% UI, 219,342,370.5–308,660,465.3), and Oceania at the tail end with 1,575,549.3 cases (95% UI, 1,266,839.4–1,969,940.4, Table S2). The period from 1990 to 2019 marked East Asia's significant increase in TTH prevalence, which was evidenced by an EAPC of 0.46 (95% CI, 0.36 to 0.56). Contrarily, Tropical Latin America registered a considerable reduction, denoted by an EAPC of -0.18 (95% CI, -0.22 to -0.13, Table S2).

Similar to migraines, the ASPR of TTH showed a positive correlation with SDI (*R* = 0.64,* P* < 0.001, Fig S5B). Thirteen regions, including High-income North America, Eastern Europe, and Western Europe, had a higher TTH prevalence than the global mean (32,508.5). Conversely, eight regions, such as Central Latin America and the Caribbean, had a lower prevalence than the global mean.

#### Incidence

In this cross-sectional study spanning 21 geographical regions, we observed the highest incidence of migraine cases in South Asia, with a figure of 12,041,286 (95% UI, 9,803,921.6 to 14,652,737.7; Table 1). Conversely, Oceania reported the lowest incidence, with a count of 82,132.5 (95% UI, 64,005.4 to 102,732.4; Table 1). Over the course of three decades, the incidence of migraines in South Asia has witnessed a substantial increase, with an EAPC of 1.23 (95% CI, 0.36 to 2.11). In contrast, Oceania exhibited the most pronounced decrease, with an EAPC of -0.28 (95% CI, -0.38 to -0.19). Pertaining to the ASIR, Western Europe topped the chart with 1,877.3 per 100,000 population (95% UI, 1,518.3 to 2,266.5), while Andean Latin America reported the lowest ASIR of migraines with 976.1 per 100,000 population (95% UI, 760.7 to 1,239.9; Table 1). As depicted in Fig S5C, a positive correlation was discernible between the incidence of migraines and the SDI (*R* = 0.34, *P* < 0.001). Furthermore, eight regions, including Western Europe, high-income North America, and Southeast Asia, reported migraine incidence rates exceeding the global mean (1,475.6). Conversely, 13 regions such as Central Asia and Central Europe reported rates lower than the global mean.

With regard to TTH, 2019 data revealed that South Asia had the highest incidence with 90,138,800.5 cases (95% UI, 73,763,193 to 108,381,706.1), while Oceania reported the lowest incidence with 536,407.8 cases (95% UI, 435,518.2 to 649,536; Table 2). From 1990 to 2019, we observed a notable increase in the incidence of TTH in East Asia, evidenced by an EAPC of 0.38 (95% CI, 0.30 to 0.45). In stark contrast, Eastern Sub-Saharan Africa experienced a significant decline, denoted by an EAPC of -0.11 (95% CI, -0.13 to -0.09; Table 2). Analogous to migraines, the incidence of TTH exhibited a positive correlation with the SDI (*R* = 0.63, *P* < 0.001; Fig S5D). Thirteen regions, including high-income North America, Eastern Europe, and Western Europe, reported TTH incidence rates surpassing the global mean (11,104.7). In contrast, eight regions, such as Central Latin America and Central Sub-Saharan Africa, recorded rates lower than the global mean.

#### DALYs

According to Table S3, South Asia recorded the highest number of migraine-associated DALYs, with 5,570,590.8 (95% UI, 535,111.6–13,023,883.2), whereas Oceania reported the lowest number, with 37,543.3 (95% UI, 3,811.6–91,419.1). Western Europe ranked first in terms of the DALY rate per 100,000 population, with 980.8 (95% UI, 106.9–2,296.7), whereas Eastern Sub-Saharan Africa had the lowest rate of 454.3 (95% UI, 77.5–1,031.2). Notably, 7 regions, including High-income North America, North Africa and the Middle East, and Southeast Asia, reported DALY rates exceeding the global average of 725.4, while 14 regions, such as South Asia, Australasia, and East Asia, had DALY rates below this global average (refer to Table S3 for details). Moreover, a positive correlation between migraine-associated DALYs and the SDI was discernible, as shown in Fig S5E (*R* = 0.28, *P* < 0.001).

In terms of TTH-associated DALYs, South Asia had the highest number, with 471,509.7 (95% UI, 112,341.7–1,986,731.2), as indicated in Table S3. Eastern Europe had the highest TTH-associated DALY rate, with 117.7 per 100,000 population (95% UI, 36.1–366.9), whereas East Asia reported the lowest rate of 53.2 per 100,000 population (95% UI, 15.4–189.2, according to Table S3). Notably, East Asia experienced a significant increase in the TTH-associated DALY rate (EAPC, 0.25; 95% CI, 0.22–0.28), while High-income North America saw a substantial decline in the DALY rate (EAPC = 0.16; 95% CI, -0.19 to -0.13, Table S3). Additionally, 11 regions, including Central Europe, Western Europe, and High-income North America, had DALY rates exceeding the global average of 67.2, while 10 regions, such as Tropical Latin America, Central Latin America, and the Caribbean, had DALY rates below this global average (refer to TableS3 for details). Furthermore, a positive correlation between TTH-associated DALYs and the SDI was observable, as depicted in Fig S5F (*R* = 0.56, *P* < 0.001).

### 204 countries level

#### Prevalence

**Fig. 2:**
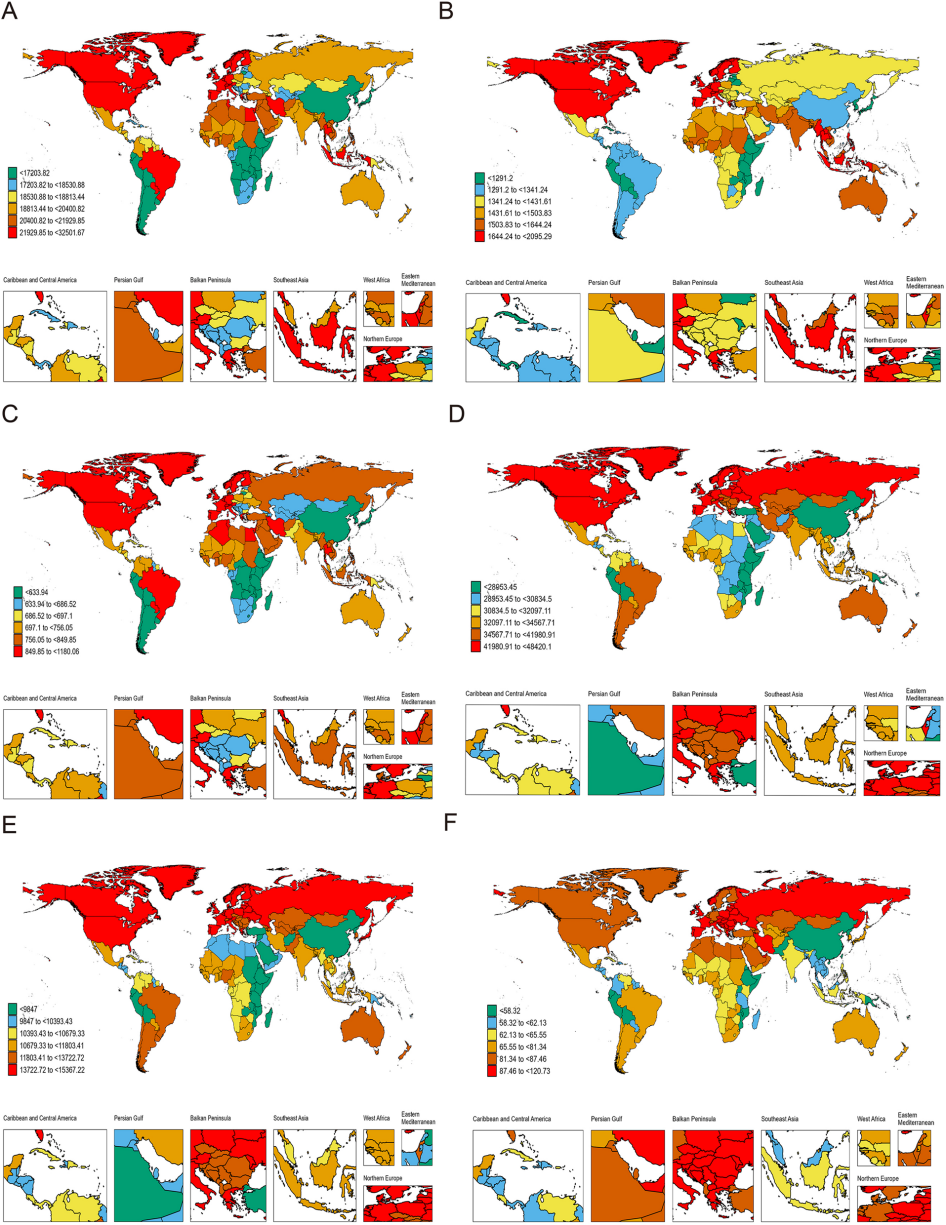
The Global Disease Burden of Migraine and TTH for Both Sexes in 204 Countries and Territories. **A** Prevalence rate for migraine. **B **Incidence rate for migraine. **C** DALYs rate for migraine. **D** Prevalenc rate for TTH. **E** Incidence rate for TTH. **F** DALYs rate for TTH

**Fig. 3:**
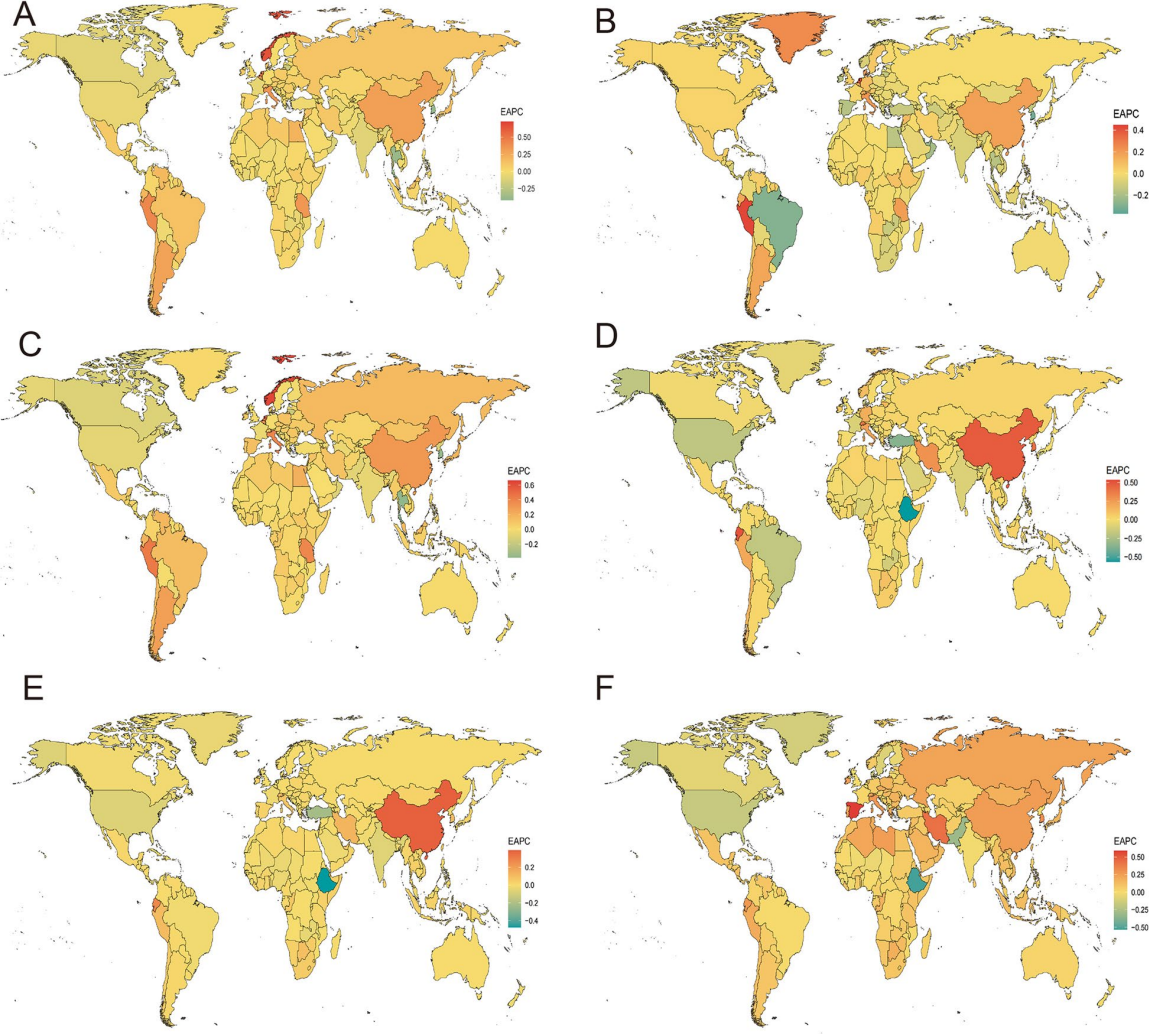
The Global Disease Burden of Migraine and TTH for Both Sexes in 204 Countries and Territories. **A** EAPC for migraine prevalence. **B** EAPC for migraine incidence. **C** EAPC for migraine DALYs. **D** EAPC for TTH prevalence. **E** EAPC for TTH incidence. **F** EAPC for TTH DALYs

**Fig. 4:**
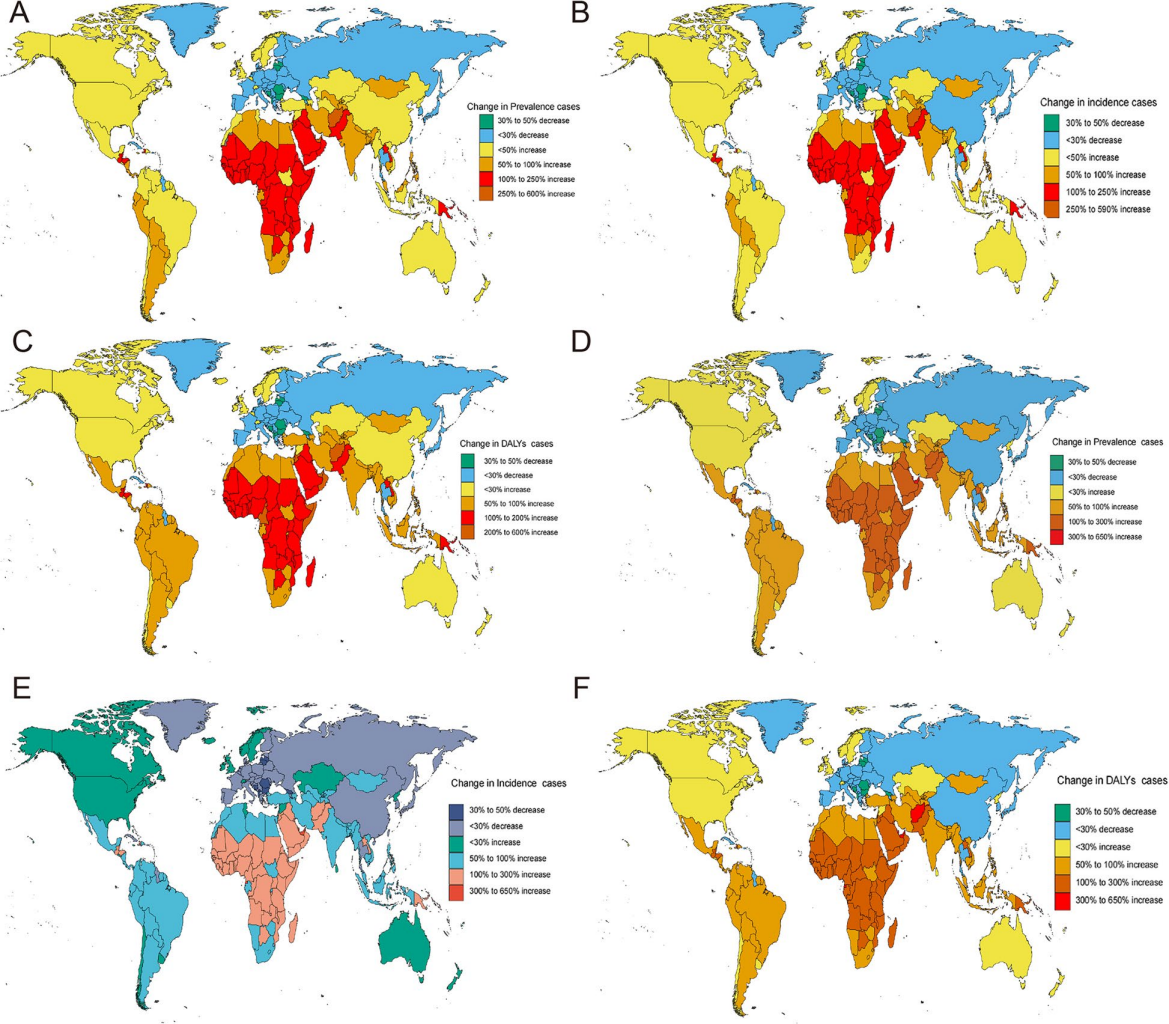
Change Cases of Migraine and TTH for Both Sexes in 204 Countries and Territories. **A **Change prevalence cases of migraine. **B** Change incidence cases of migraine. **C **Change dalys cases of migraine. **D **Change prevalence cases of TTH. **E **Change incidence cases of TTH. **F** Change DALYs cases of TTH

Globally, the prevalence trend of migraine among individuals aged 15 to 39 years, spanning from 1990 to 2019, exhibited significant variations across nations. In 2019, among 204 countries, Belgium had the highest incidence rate of migraine, with 32,501.7 cases per 100,000 population (95% UI, 26,861–39,157.7, Table S4, Fig. 2A). In contrast, Ethiopia reported the lowest rate, with 11,104.5 cases per 100,000 population (95% UI, 9,290–13,139.4, Table S4, Fig. 2A). Over the period from 1990 to 2019, Singapore experienced the most significant increase in the prevalence rate of migraine (EAPC, 0.73; 95% CI, 0.50–0.96, Table S4, Fig. 3A). Conversely, the Republic of Korea saw the most substantial decrease (EAPC, -0.42; 95% CI, -0.47 to -0.37, Table S4, Fig. 3A). Concerning the number of cases, Qatar had the largest increase (up 597%), followed by the United Arab Emirates with a 363% increase. In contrast, Bosnia and Herzegovina reported a 45% decrease (Fig. 4A).

Regarding TTH, Italy had the highest ASPR in 2019, with 48,420.1 cases per 100,000 population (95% UI, 41,125.9–56,614.5, Table S5). Ethiopia reported the lowest rate, with 20,992.6 cases per 100,000 population (95% UI, 16,881.6–25,752.9, Table S5). From 1990 to 2019, Singapore saw the largest increase in the prevalence rate of TTH (EAPC, 0.53; 95% CI, 0.37–0.69, Table S5, Fig. 3D). In contrast, Ethiopia experienced the most significant decrease (EAPC, -0.56; 95% CI, -0.67 to -0.46, Table S5, Fig. 3D). Concerning the number of cases, Qatar had the largest increase (up 625%), while Bosnia and Herzegovina reported a 45% decrease (Fig. 4D).

#### Incidence

In 2019, Italy reported the highest incidence rate of migraines among 204 countries, with 2095.3 cases per 100,000 population (95% UI, 1758.3–2492.4, Table S6, Fig. 2B). Conversely, Peru had the lowest rate, with 930.6 cases per 100,000 population (95% UI, 728.6–1167.2, Table S6, Fig. 2B). From 1990 to 2019, Singapore saw the most significant increase in migraine incidence rate (EAPC, 0.45; 95% CI, 0.29–0.61, TableS6, Fig. 3B). In contrast, Qatar experienced the most substantial decrease (EAPC, -0.37; 95% CI, -0.48 to -0.26, Table S6, Fig. 3B). When considering the number of cases, Qatar had the largest increase (up 580 percent), followed by the United Arab Emirates with a 344 percent increase. Bosnia and Herzegovina, however, reported a 45 percent decrease (Fig. 4B). Regarding TTH, Italy again had the highest incidence rate in 2019, with 15,367.2 cases per 100,000 population (95% UI, 12,466–18,512.1, Table S7, Fig. 2E). The Democratic People's Republic of Korea reported the lowest rate, with 7976 cases per 100,000 population (95% UI, 6441.3–9771.6, Table S7, Fig. 2E). Between 1990 and 2019, China experienced the most significant increase in the incidence rate of TTH, with an EAPC of 0.39 (95% CI, 0.31–0.46, Table S7, Fig. 3E). Conversely, Ethiopia saw the most significant decrease, with an EAPC of -0.47 (95% CI, -0.56 to -0.38, Table S7, Fig. 3E). In terms of the number of cases, Qatar had the most extensive increase, with a rise of 627 percent, while Bosnia and Herzegovina experienced a 45 percent decrease (Fig. 4E).

#### DALYs

In 2019, out of 204 countries, Belgium, Italy, and Norway documented the highest age-standardized DALY rates for migraine, registering 1,180.1, 1,105, and 1,028.9 per 100,000 population, respectively (Table S8, Fig. 2C). In contrast, Ethiopia, Somalia, and South Sudan reported the lowest rates, with values of 410.4, 450.6, and 454.5 per 100,000 population, respectively (Table S8). Between 1990 and 2019, Singapore, Norway, and the Netherlands exhibited the most marked increases in age-standardized DALY rates, with EAPCs of 0.66, 0.63, and 0.47, respectively. Conversely, the most significant declines were observed in Thailand, the Republic of Korea, and the Northern Mariana Islands, with EAPCs of -0.38, -0.36, and -0.25, respectively (Table S8, Fig. 3C). Regarding absolute changes, Qatar witnessed the most substantial rise in migraine-associated DALYs by 600%, whereas Bosnia and Herzegovina experienced the steepest decrease at 45% (Fig. 4C). Pertaining to TTH-associated DALYs in 2019, the Russian Federation recorded the highest rate at 120.7 per 100,000 population. In stark contrast, Ethiopia had the lowest, with 46.4 per 100,000 population (Table S9, Fig. 2F). Once again, Qatar led with the most considerable increase in TTH-associated DALYs by 626%, while the Northern Mariana Islands noted the most substantial decline at 44% (Fig. 4F). Concerning rate changes, Ethiopia underwent the most pronounced decrease in DALY rate, with an EAPC of -0.53. Conversely, Spain registered the highest increase, with an EAPC of 0.59 (Fig. 3F).

### Future burden of migraine and TTH

Figure S6 illustrates the projected trends in global migraine prevalence. The graph indicates a steady rise in the number of individuals affected by migraines worldwide. Notably, regions with high SDI consistently exhibit a higher prevalence compared to other areas and the global average. Conversely, regions with low and lower-middle SDI are expected to experience a slight decrease in migraine prevalence. It is also worth noting that the prevalence are higher in women than in men (Fig S6). The prevalence of TTH is projected to increase between now and 2040. Interestingly, the number of men suffering from TTH is expected to surpass that of women. Globally, as well as in the five SDI regions, a slight uptick in the prevalence rate is anticipated (refer to Fig S7 for more details).

## Discussion

To date, the literature lacks a comprehensive analysis detailing the prevalence, incidence, and DALYs associated with migraine and TTH across 204 countries and territories, specifically targeting the age group of 15–39 years. Prior investigations have either restricted their focus to particular age cohorts or select global regions or have relied upon antiquated datasets without giving precedence to distinct demographic segments [17–19]. We believe that the burden associated with migraine and TTH needs to be updated regularly and policymakers need to be kept informed to help identify best practices for headache prevention and control worldwide. This study, the first of its kind, investigates the burden of migraine and TTH in youths and young adults aged 15–39 years from 1990 to 2019. It offers a comprehensive analysis of the global, regional, and national impact of these conditions, utilizing data from the GBD 2019.

In 2019, the global incidence of migraines among individuals aged 15 to 39 years stood at 581,761,847.2 (95% UI: 488,309,998.1 to 696,291,713.7), representing a 16% increase from the 1990 figures. Simultaneously, the incidence of TTH in individuals aged 15 to 39 years surged to 964,808,567.1 (95% UI: 809,582,531.8 to 1,155,235,337.2), reflecting a marked 37% increase from the baseline in 1990.

Regionally, the most rapid increments in migraine and tension-type headache prevalence were observed in Andean Latin America, East Asia, and Southern Latin America. Currently, South Asia, East Asia, and Southeast Asia bear the highest burdens of these disorders. Striking intercountry disparities exist, with some nations exhibiting up to a six-fold difference in prevalence. High-income nations, notably Singapore, Greenland, and Italy, aren't exempt, revealing sharp upward trends. Consequently, country-specific strategies for prevention and treatment are paramount. Over-reliance on regional or global models might inadvertently misdirect national health policies.

Consistent with previous findings, our study demonstrated that the burden of migraine and TTH gradually increases with age, peaking in the 30–39 age group. Additionally, we found that women bear a higher burden of these disorders than men across all age groups [19]. In addition, in a study of adolescent headache disorders, the study identified a higher incidence in the 15–24 year age group and in males. The discrepancy observed may be attributed to the age structure of the population studied. Prior epidemiological studies have indicated a significant gender disparity in the prevalence of headache disorders, with women being three times more likely to be affected than men [20]. This gender-related difference has been corroborated by results from both structural and functional MRI studies [20]. Furthermore, biological and psychological differences between males and females may contribute to the higher prevalence in females. Possible factors include sex hormones, genetic predispositions, exposure to environmental stressors, and differing responses to stress and pain [21, 22]. The onset of puberty and menarche marks a significant rise in headache disorder prevalence among women, a trend that persists during menstruation, pregnancy, and menopause. This pattern implies that fluctuations in female sex hormones, especially estrogen, play a pivotal role [21]. The aforementioned studies highlight the need for targeted interventions that effectively address the most prevalent risk factors among these priority groups.

Our study revealed a global increase in the prevalence of migraine and TTH over a 30-year period. We also observed a positive correlation between prevalence, incidence, and SDI, particularly in regions with high SDI. These regions exhibited higher levels of ASIR and ASPR compared to global averages and other regions. This upward trend in the worldwide prevalence of migraine and TTH, particularly among youths and young adults, underscores the urgent need for enhanced preventative measures and control initiatives targeting this demographic. Several potential factors may contribute to this observed increase. Possible contributing factors include shifts in societal behaviors, heightened dependence on electronic devices, insufficient sleep hygiene, and escalating psychological stress among younger demographics [23–26].

The rapid escalation in prevalence across Andean Latin America, East Asia, and South Asia may reflect swift socioeconomic changes in these regions that could impact headache rates among younger populations. Significant contributing factors include urbanization, healthcare access, and air quality, all of which are undergoing transformation in these areas and likely play critical roles in headache incidence among youth [27–29]. A similar rationale likely underlies the sharp escalations in Singapore, Greenland, and Italy. Conversely, the downward trends in Thailand, Pakistan, and the United States imply that health promotion and public health initiatives have successfully decreased headache triggers and improved disease management in these nations. Sustained monitoring of these patterns can facilitate recognition of optimal global strategies for headache prevention and control.

Our findings have significant implications for healthcare services, as primary headache disorders, including migraines and TTH, rank among the leading causes of disability worldwide. The high prevalence across age groups underscores the need for targeted intervention strategies. It is critical to integrate primary headache care into health systems globally, as such integration is deemed an effective, sustainable approach. This could strengthen identification of at-risk patients and bolster prevention and treatment strategies to mitigate disease burden. Additionally, comprehensive data collection on migraine and tension-type headache would be facilitated. Past research reveals poor adherence to preventive medications among headache patients; however, patient education guided by specific protocols can improve adherence.

### Limitations

Our analysis primarily focused on the trends associated with two prominent headache types: migraines and TTH. Consequently, this study does not encompass trends for other headache disorders. It's important to note that our research is grounded in the GBD studies, which do not utilize primary data. Many nations, especially in the Global South—including various African countries grappling with conflicts and unrest—rely heavily on unverified estimates. These nations often face challenges in terms of resources, expertise, and the overall infrastructure required for comprehensive headache epidemiology at a population scale. Such reliance could introduce bias and potential inaccuracies into our estimates.The diagnostic criteria for migraines and TTH might vary, potentially resulting in inconsistent incidence rates. Such discrepancies can be exacerbated by cultural, regional, and methodological differences. We must acknowledge that these inconsistencies might also be affected by regional and cultural nuances in identifying cases. The noticeable increase in headache burden since the early 1990s may be attributed in part to advancements in diagnosis and heightened public awareness. However, our research doesn't distinguish the potential influence of these developments. Without specific studies addressing this influence, our conclusions might be skewed. While we have made efforts to account for methodological differences across studies, it's worth mentioning that some observed variations could stem from measurement inaccuracies or inherent methodological biases, rather than true differences.

## Conclusions

Migraine and TTH manifest as substantial burdens on a global scale. In 2019, worldwide data revealed that among individuals aged 15 to 39 years, there were approximately 581,761,847.2 documented cases of migraines, signifying a 16% increase since 1990. Simultaneously, TTH cases within this demographic reached a staggering 964,808,567.1, marking a 37% ascent compared to 1990 figures. Noteworthy disparities exist among countries; regions with a high SDI reported the most pronounced prevalence rates for migraines in 2019, while the mid-SDI regions registered the zenith of TTH cases that year. In addition, our predictions suggest that a rise in migraine prevalence, particularly in high SDI regions, with females predominantly affected. Concurrently, TTH are expected to escalate, with upcoming male cases surpassing female ones. Our study highlights increased incidence, prevalence, and DALYs of migraine and TTH in individuals aged 15–39. Understanding this burden is key to creating cost-effective interventions, which could reduce morbidity and mortality while improving global health management. This knowledge enables better allocation of preventive and therapeutic measures, promoting a more balanced global health landscape.

### Supplementary Information

**Additional file 1: Fig S1. **Trends in Migraine Prevalence, Incidence and Disability-Adjusted Life-Years (DALYs) From 1990 to 2019. (A) Trends in prevalence Rate (B) Trends in incidence Rate (C)Trends in DALYs Rate

**Additional file 2: Fig S2.** Ratio of Male to Female Prevalence, Incidence and Disability-Adjusted Life-Years (DALYs) of Migraine Diseases in Different Age Subgroups. (A): Male-female Ratio of Prevalence (B):Male-female Ratio of Incidence (C):Male-female Ratio of DALYs

**Additional file 3: Fig S3. **Trends in TTH Prevalence, Incidence and Disability-Adjusted Life-Years (DALYs) From 1990 to 2019. (A) Trends in prevalence cases and prevalence rate (B) Trends in incidence cases and incidence rate (C)Trends in DALYs cases and DALYs rate

**Additional file 4: Fig S4. **Ratio of Male to Female Prevalence, Incidence and Disability-Adjusted Life-Years (DALYs) of TTH in Different Age Subgroups. (A): Male-female Ratio of Prevalence (B): Male-female Ratio of Incidence (C): Male-female Ratio of DALYs 

**Additional file 5: Fig S5. **(A): Association between Age-standardized Migraine Prevalence Rate and Sociodemographic Index. (B): Association between Age-standardized TTH Prevalence Rate and Sociodemographic Index. (C): Association between Age-standardized Migraine Incidence Rate and Sociodemographic Index (D): Association between Age-standardized TTH Incidence Rate and Sociodemographic Index. (E): Association between Age-standardized Migraine DALYs Rate and Sociodemographic Index. (F): Association between Age-standardized TTH DALYs Rate and Sociodemographic Index.

**Additional file 6: Fig S6. **Future Forecasts of GBD in Migraine Prevalence.

**Additional file 7: Fig S7. **Future Forecasts of GBD in TTH Prevalence.

**Additional file 8: Table S1. **Prevalence of Migraine Between 1990 and 2019 in 15 to 39 years at the Global and Regional Level.

**Additional file 9: Table S2. **Prevalence of Tension-Type Headache Between 1990 and 2019 in 15 to 39 years at the Global and Regional Level.

**Additional file 10: Table S3. **DALYs of Migraine and TTH Between 1990 and 2019 in 15 to 39 years at the Global and Regional Level.

**Additional file 11: Table S4. **Prevalence of Migraine Between 1990 and 2019 in 15 to 39 years at the 204 Countries Level.

**Additional file 12: Table S5. **Prevalence of TTH Between 1990 and 2019 in 15 to 39 years at the 204 Countries Level.

**Additional file 13: Table S6. **Incidence of Migraine Between 1990 and 2019 in 15 to 39 years at the 204 Countries Level.

**Additional file 14: Table S7. **Incidence of TTH Between 1990 and 2019 in 15 to 39 years at the 204 Countries Level.

**Additional file 15: Table S8. **DALYs of Migraine Between 1990 and 2019 in 15 to 39 years at the 204 Countries Level.

**Additional file 16: Table S9. **DALYs of TTH Between 1990 and 2019 in 15 to 39 years at the 204 Country Level.

## Data Availability

GBD study 2019 data resources were available online from the Global Health Data Exchange (GHDx) query tool ( http://ghdx.healthdata.org/gbd-results-tool).

## Abbreviations

TTH: Tension-Type Headache
DALYs: Disability-Adjusted Life Years
GBD: Global Burden of Disease
SDI: Socio-Demographic Index
EAPC: Estimated Annual Percentage Changes
ASR: Age-Standardized Rate
ASIR: Age-Standardized Incidence Rate
ASPR: Age-Standardized Prevalence Rate
CI: Confidence Interval
UI: Uncertainty Intervals

## References

[1] Boström A, Scheele D, Stoffel-Wagner B (2019). Saliva molecular inflammatory profiling in female migraine patients responsive to adjunctive cervical non-invasive vagus nerve stimulation: the MOXY Study[J]. J Transl Med.

[2] Duko B, Ayalew M, Toma A (2020). The epidemiology of headaches among patients with epilepsy: a systematic review and meta-analysis[J]. J Headache Pain.

[3] Leonardi M, Grazzi L, D'Amico D, Martelletti P, Guastafierro E, Toppo C, Raggi A (2020). Global burden of headache disorders in children and adolescents 2007–2017. Int J Environ Res Public Health.

[4] GBD 2019 Diseases and Injuries Collaborators (2020) Global burden of 369 diseases and injuries in 204 countries and territories, 1990-2019: a systematic analysis for the Global Burden of Disease Study 2019. Lancet 396(10258):1204–22. 10.1016/S0140-6736(20)30925-9. Erratum in: Lancet. 2020;396(10262):1562 10.1016/S0140-6736(20)30925-9 PMC7567026 33069326

[5] Osman Ali MM, Mohamed Ahmed KAH, Omer MEA (2022). Prevalence of migraine headaches and their impact on the academic performance of Sudanese medical students using ID-Migraine test as a screening tool: A cross-sectional study. Brain behav..

[6] Dodick DW (2018). Migraine. Lancet (London, England).

[7] Koller LS, Diesner SC, Voitl P (2019). Quality of life in children and adolescents with migraine: an Austrian monocentric, cross-sectional questionnaire study.. BMC pediatr.

[8] GBD 2016 Headache Collaborators (2018) Global, regional, and national burden of migraine and tension-type headache, 1990-2016: a systematic analysis for the Global Burden of Disease Study 2016. Lancet Neurol 17(11):954–76. 10.1016/S1474-4422(18)30322-3. Erratum in: Lancet Neurol. 2021;20(12):e7 10.1016/S1474-4422(18)30322-3 PMC6191530 30353868

[9] Haque B, Rahman KM, Hoque A (2012). Precipitating and relieving factors of migraine versus tension type headache. BMC neurol.

[10] Ge R, Chang J (2023). Disease burden of migraine and tension-type headache in non-high-income East and Southeast Asia from 1990 to 2019. The journal of headache and pain.

[11] Collaborators GHL (2021). Hearing loss prevalence and years lived with disability, 1990–2019: findings from the Global Burden of Disease Study 2019[J]. Lancet (London, England).

[12] Headache Classification Committee of the International Headache Society (IHS) (2018). The International Classification of Headache Disorders, 3rd edition. Cephalalgia.

[13] Molassiotis A, Kwok SWH, Leung AYM (2022). Associations between sociodemographic factors, health spending, disease burden, and life expectancy of older adults (70 + years old) in 22 countries in the Western Pacific Region, 1995–2019: estimates from the Global Burden of Disease (GBD) Study 2019. Geroscience.

[14] Collaborators GDAH (2016). Global, regional, and national disability-adjusted life-years (DALYs) for 315 diseases and injuries and healthy life expectancy (HALE), 1990–2015: a systematic analysis for the Global Burden of Disease Study 2015[J]. Lancet (London, England).

[15] Fu M, Zhou H, Li Y (2022). Global, regional, and national burdens of hip osteoarthritis from 1990 to 2019: estimates from the 2019 Global Burden of Disease Study. Arthritis Res Ther.

[16] Ou Z, Li X, Cui J, Zhu S, Feng K, Ma J et al (2023) Global, regional, and national burden of asbestosis from 1990 to 2019 and the implications for prevention and control. Sci Total Environ 904:166346. 10.1016/j.scitotenv.2023.166346 10.1016/j.scitotenv.2023.166346 37591378

[17] Stovner LJ, Hagen K, Linde M (2022). The global prevalence of headache: an update, with analysis of the influences of methodological factors on prevalence estimates. J headache pain.

[18] Ying Y, Cao Yu (2023). Rising trends in the burden of migraine and tension-type headache among adolescents and young adults globally. J Headache Pain.

[19] Safiri S, Pourfathi H, Eagan A (2022). Global, regional, and national burden of migraine in 204 countries and territories, 1990 to 2019. Pain.

[20] Maleki N, Linnman C, Brawn J (2012). Her versus his migraine: multiple sex differences in brain function and structure. Brain..

[21] Delaruelle Z, Ivanova TA, Khan S (2018). Male and female sex hormones in primary headaches. J headache pain.

[22] Wessman M, Terwindt GM, Kaunisto MA (2007). Migraine: a complex genetic disorder. Lancet Neurol.

[23] Dao JM, Qubty W (2018). Headache Diagnosis in Children and Adolescents. Curr pain headache rep.

[24] Xavier MKA, Pitangui ACR, Silva GRR (2015). Prevalence of headache in adolescents and association with use of computer and videogames. Cien saude colet.

[25] Theeler BJ, Erickson JC (2012). Posttraumatic headache in military personnel and veterans of the iraq and afghanistan conflicts. Curr treat options neurol.

[26] Mowat C, Cole A, Windsor A (2011). Guidelines for the management of inflammatory bowel disease in adults. Gut.

[27] Lederbogen F, Kirsch P, Haddad L (2011). City living and urban upbringing affect neural social stress processing in humans. Nature..

[28] Pacheco-Barrios K, Velasquez-Rimachi V, Navarro-Flores A (2023). Primary headache disorders in Latin America and the Caribbean: A meta-analysis of population-based studies. Cephalalgia.

[29] Atun R, de Andrade LOM, Almeida G (2015). Health-system reform and universal health coverage in Latin America. Lancet (London, England).

