# Correction: Global, regional, and national epidemiology of migraine and tension-type headache in youths and young adults aged 15–39 years from 1990 to 2019: findings from the global burden of disease study 2019

**DOI:** 10.1186/s10194-023-01693-z

**Published:** 2023-11-22

**Authors:** Xin-yu Li, Cheng-hao Yang, Jia-jie Lv, Hui Liu, Lu-yu Zhang, Min-yi Yin, Zhi-lin Guo, Ru-hong Zhang

**Affiliations:** 1 Department of Plastic and Reconstructive Surgery, Shanghai Ninth People’s Hospital, Shanghai JiaoTong University School of Medicine, Huangpu District, No.639 Zhizaoju Road, Shanghai, 200011 China; 2 Department of Neurosurgery, Shanghai Ninth People’s Hospital, Shanghai Jiao Tong University, Shanghai, People’s Republic of China; 3 Department of Vascular Surgery, School of Medi- Cine, Shanghai Putuo People’s Hospital, Tongji University, Shanghai, China; 4 Department of Vascular Surgery, Shanghai Ninth People’s Hospital, Shanghai Jiao Tong University, Shanghai, China; 5 Department of Emergency, Shanghai Jiao Tong University Affiliated Sixth People’s Hospital, Shanghai, 200060 China; 6 The Department of Urology, Shangqiu First People’s Hospital, Shangqiu, China

**Correction: J Headache Pain 24, 126 (2023)**

**https://doi.org/10.1186/s10194-023-01659-1**

All data in this article [1] has been sourced from the Global Burden of Disease (GBD) database. While the authors adhered to IHME's terms of use and guidelines (https://www.healthdata.org/Data-tools-practices/data-practices/ihme-free-charge-non-commercial-user-agreement), the source identifier required by the agreement had been omitted: *Source: Institute for Health Metrics and Evaluation. Used with permission. All rights reserved*. Additionally, it has come to our attention that the phrase "Our study, conducted from 1990 to 2019" may appear misleading. Contrary to what may be inferred, the authors were never part of the GBD project.
