# Supplementary figures and images for: Global, regional, and national epidemiology of migraine and tension-type headache in youths and young adults aged 15–39 years from 1990 to 2019: findings from the global burden of disease study 2019
Source: J Headache Pain. 2023 Sep 18;24(1):126. doi: 10.1186/s10194-023-01659-1 (PMC10506184; doi:10.1186/s10194-023-01659-1)

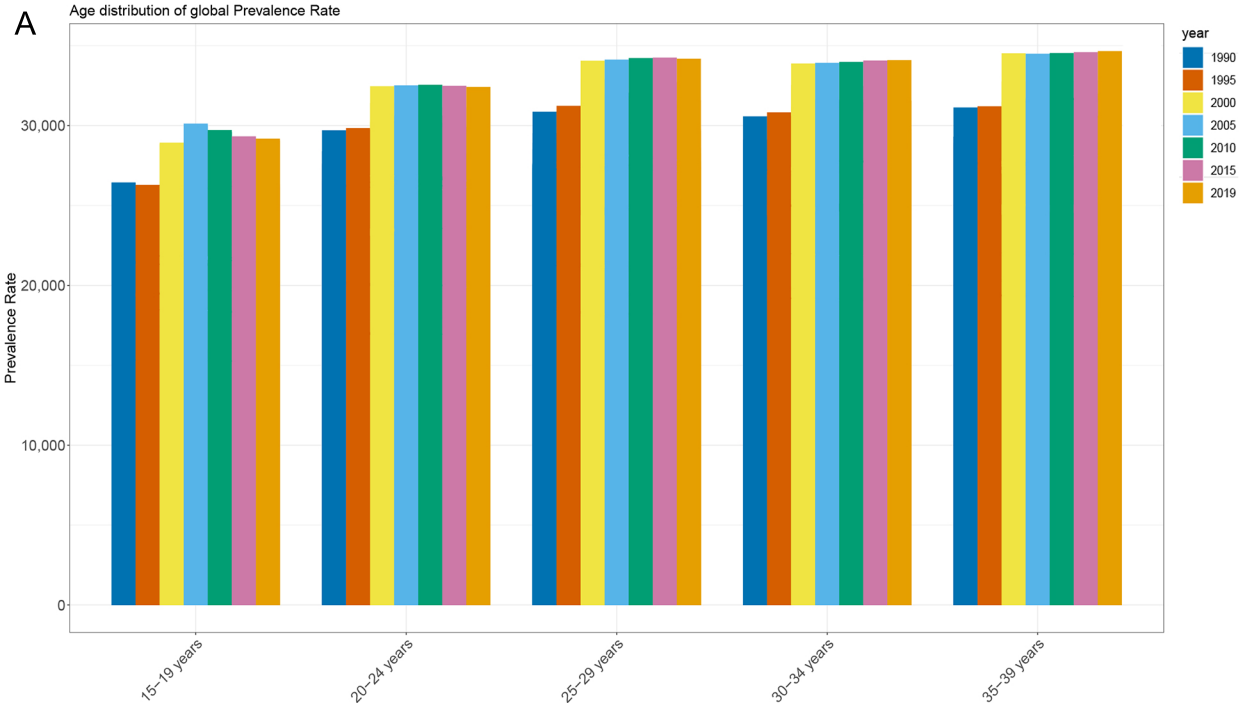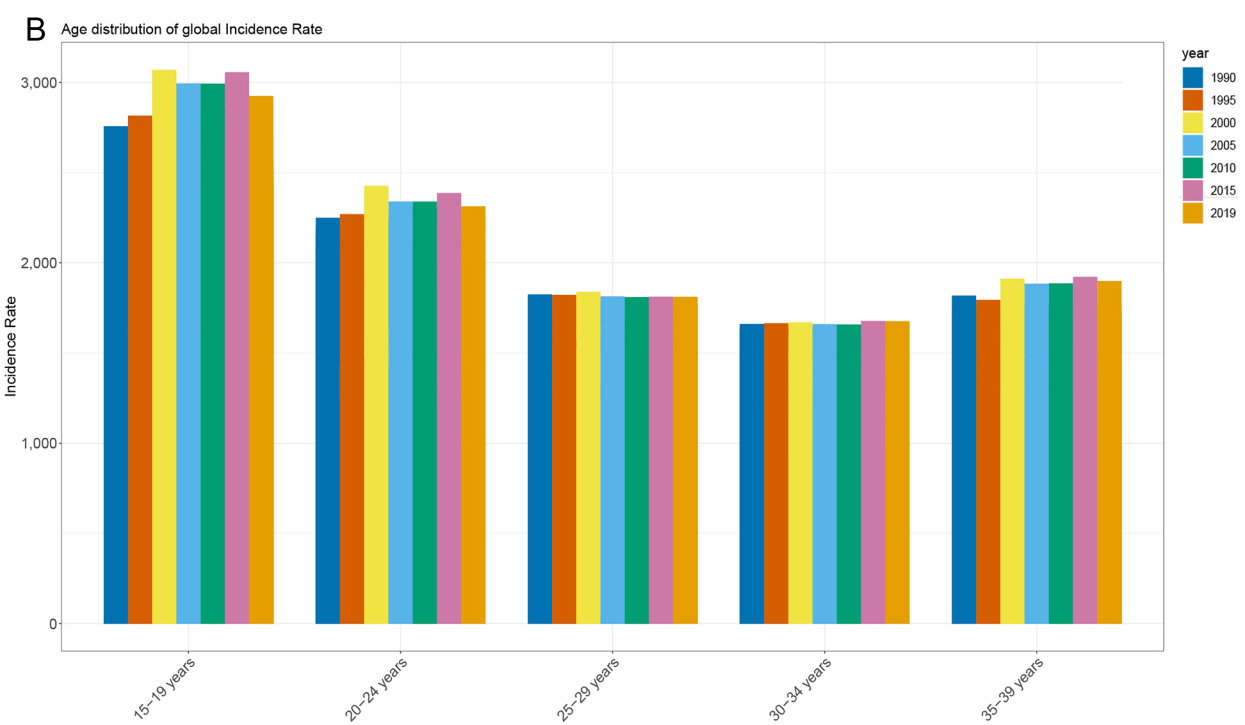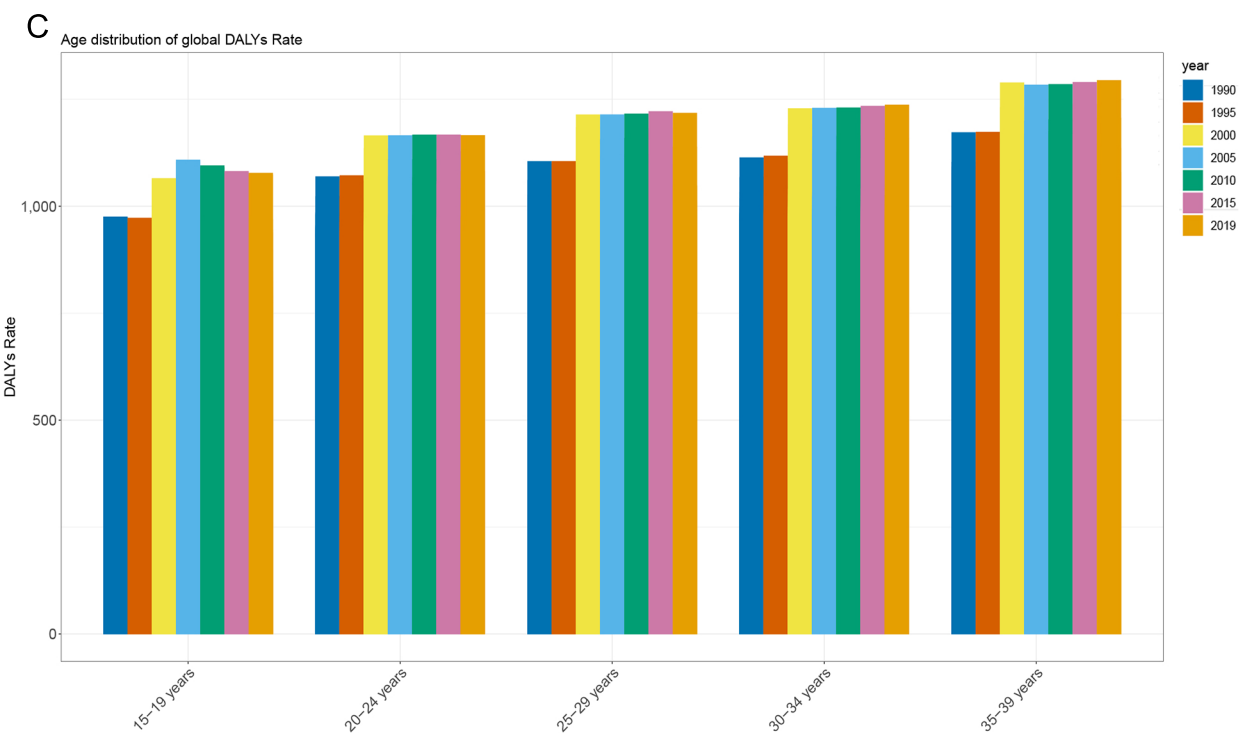

Supplement: Supplementary file 1 — Additional file 1: Fig S1. Trends in Migraine Prevalence, Incidence and Disability-Adjusted Life-Years (DALYs) From 1990 to 2019. (A) Trends in prevalence Rate (B) Trends in incidence Rate (C)Trends in DALYs Rate [file 10194_2023_1659_MOESM1_ESM.pdf]

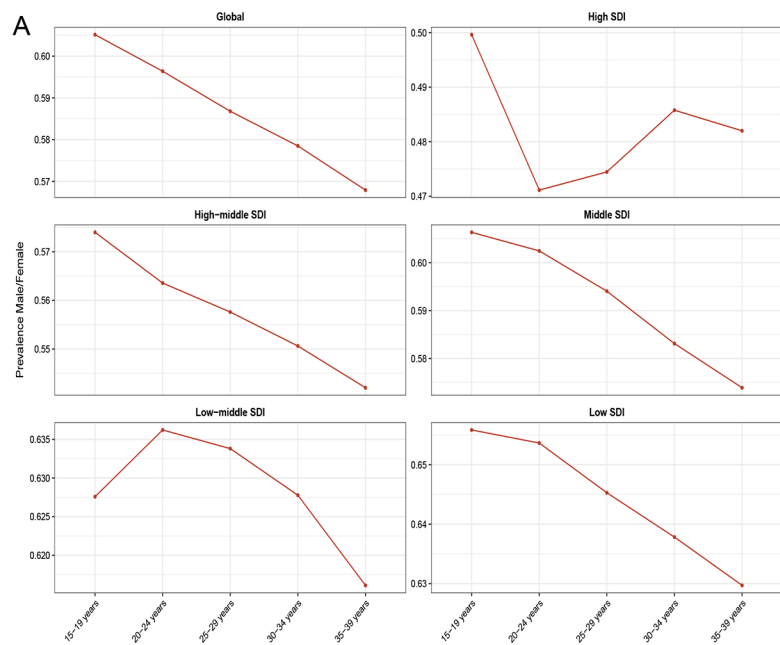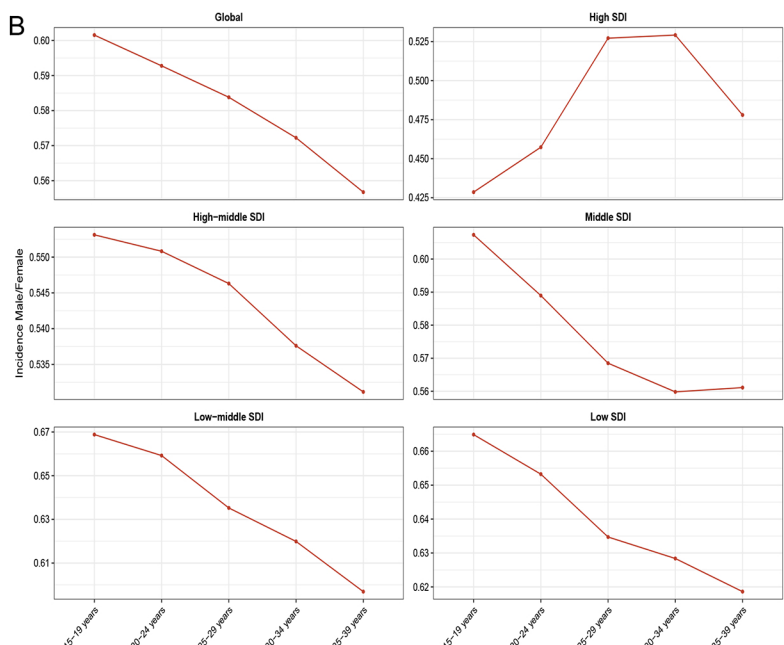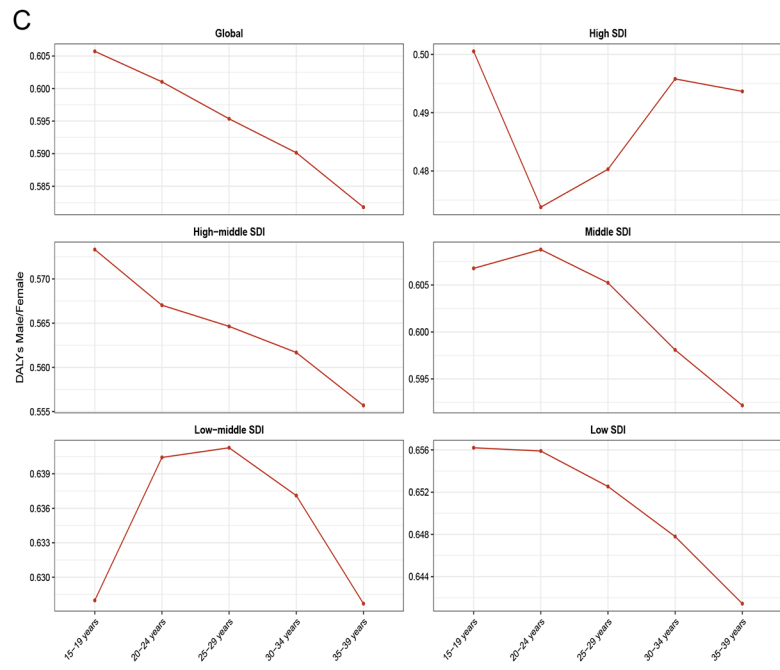

Supplement: Supplementary file 2 — Additional file 2: Fig S2. Ratio of Male to Female Prevalence, Incidence and Disability-Adjusted Life-Years (DALYs) of Migraine Diseases in Different Age Subgroups. (A): Male-female Ratio of Prevalence (B):Male-female Ratio of Incidence (C):Male-female Ratio of DALYs [file 10194_2023_1659_MOESM2_ESM.pdf]

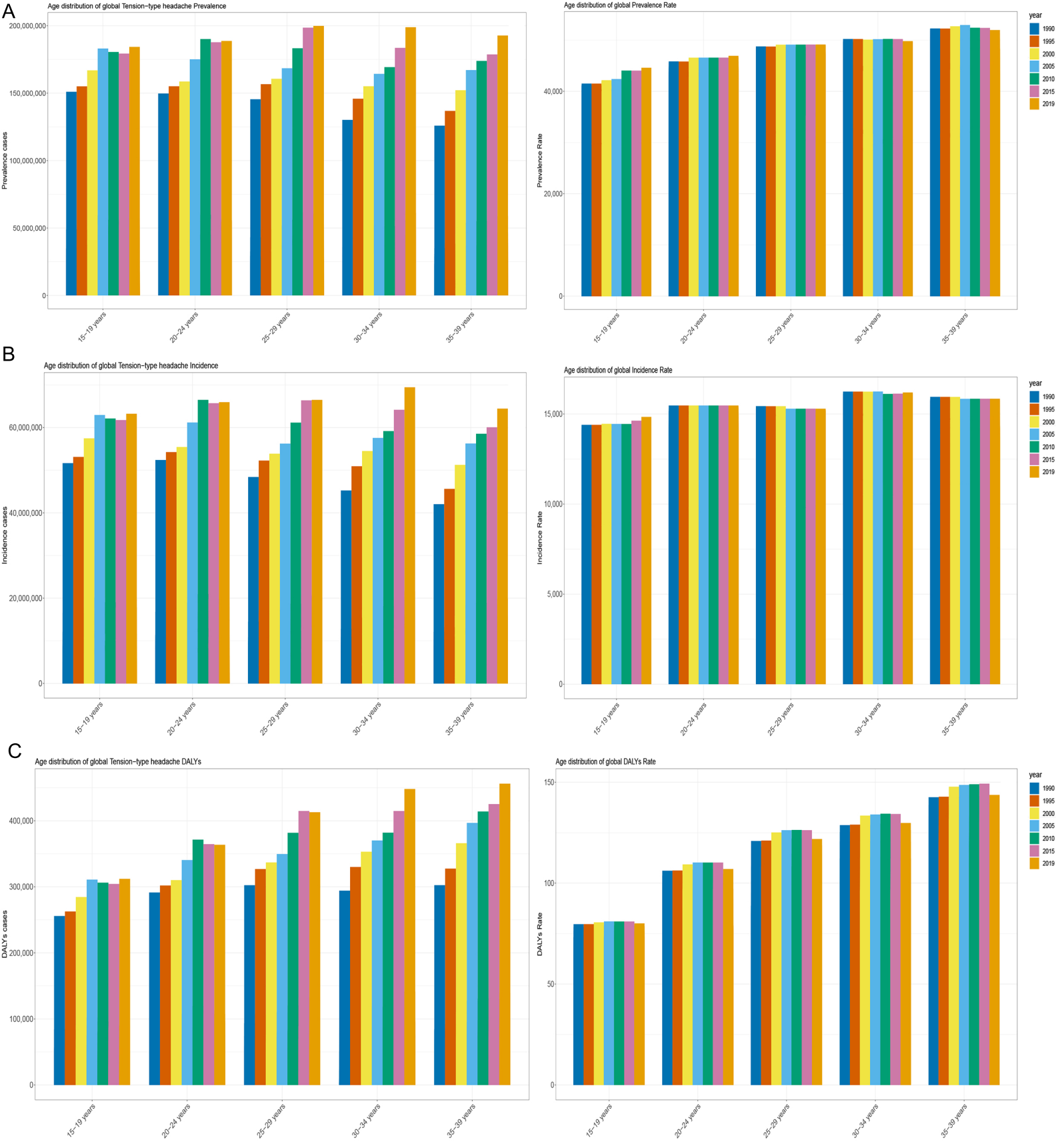

Supplement: Supplementary file 3 — Additional file 3: Fig S3. Trends in TTH Prevalence, Incidence and Disability-Adjusted Life-Years (DALYs) From 1990 to 2019. (A) Trends in prevalence cases and prevalence rate (B) Trends in incidence cases and incidence rate (C)Trends in DALYs cases and DALYs rate [file 10194_2023_1659_MOESM3_ESM.pdf]

**A**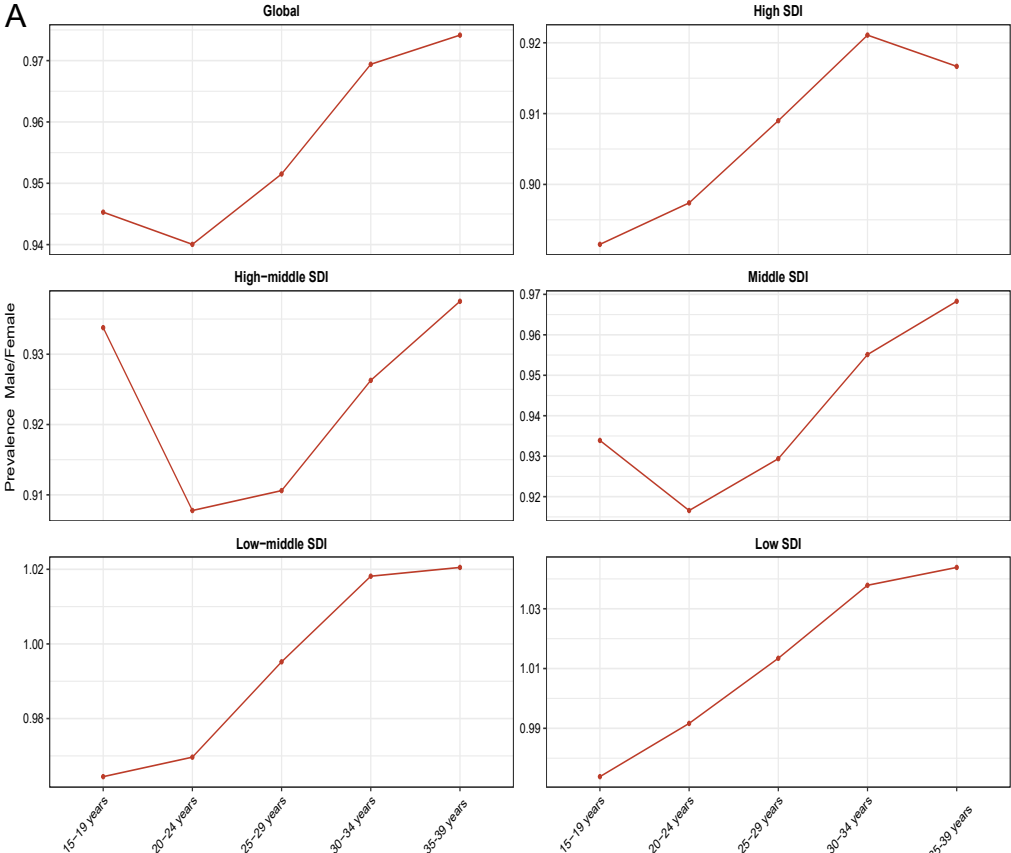**B**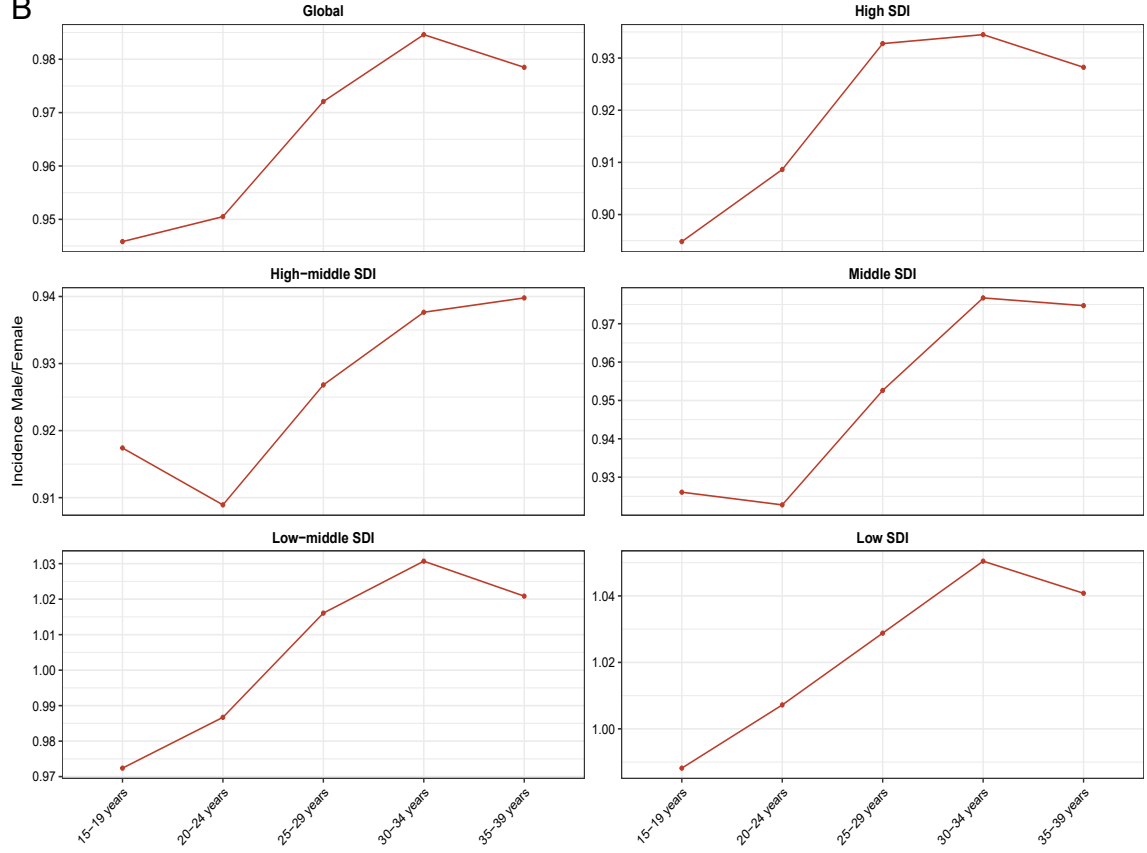**C**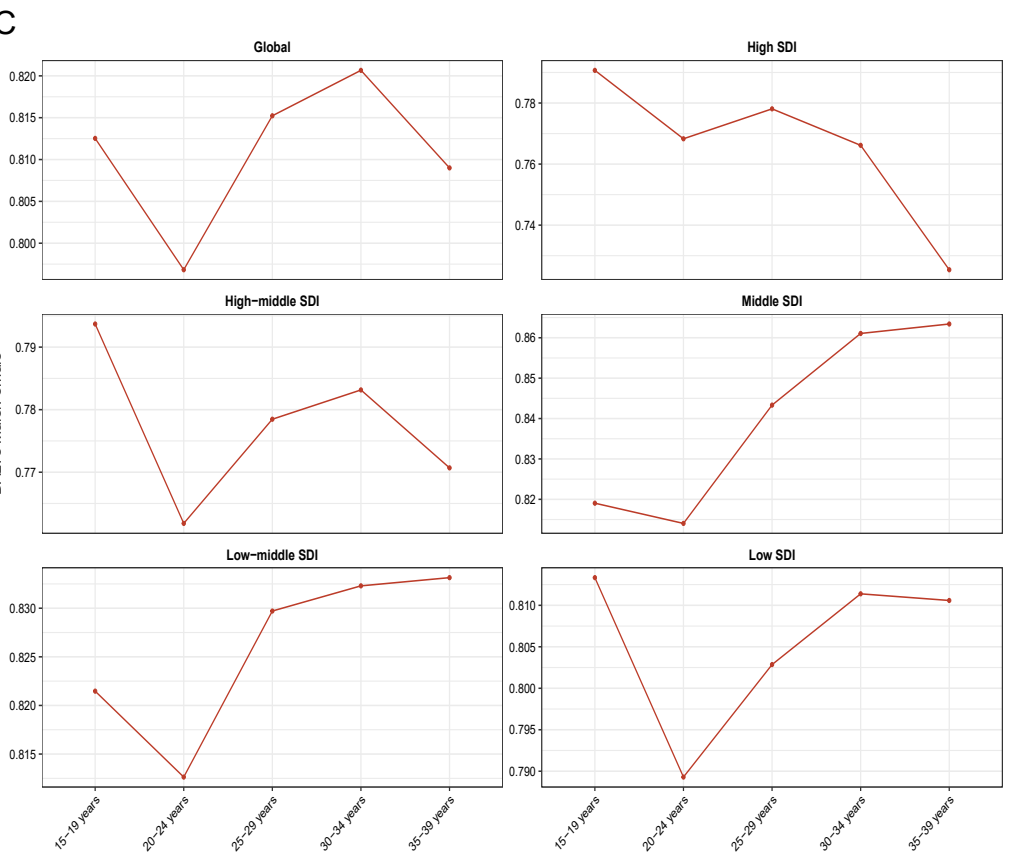

Supplement: Supplementary file 4 — Additional file 4: Fig S4. Ratio of Male to Female Prevalence, Incidence and Disability-Adjusted Life-Years (DALYs) of TTH in Different Age Subgroups. (A): Male-female Ratio of Prevalence (B): Male-female Ratio of Incidence (C): Male-female Ratio of DALYs [file 10194_2023_1659_MOESM4_ESM.pdf]

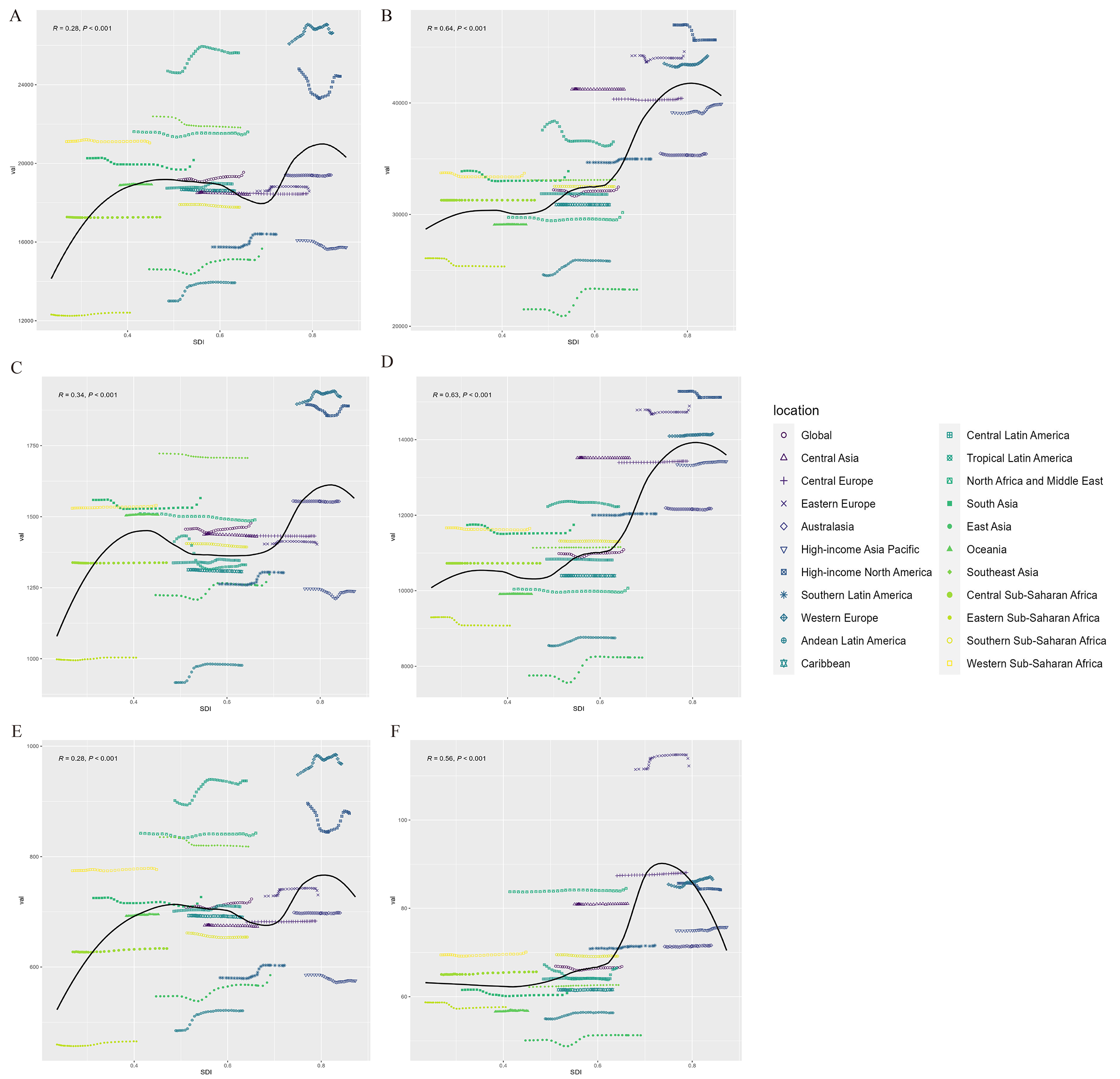

Supplement: Supplementary file 5 — Additional file 5: Fig S5. (A): Association between Age-standardized Migraine Prevalence Rate and Sociodemographic Index. (B): Association between Age-standardized TTH Prevalence Rate and Sociodemographic Index. (C): Association between Age-standardized Migraine Incidence Rate and Sociodemographic Index (D): Association between Age-standardized TTH Incidence Rate and Sociodemographic Index. (E): Association between Age-standardized Migraine DALYs Rate and Sociodemographic Index. (F): Association between Age-standardized TTH DALYs Rate and Sociodemographic Index. [file 10194_2023_1659_MOESM5_ESM.jpg]

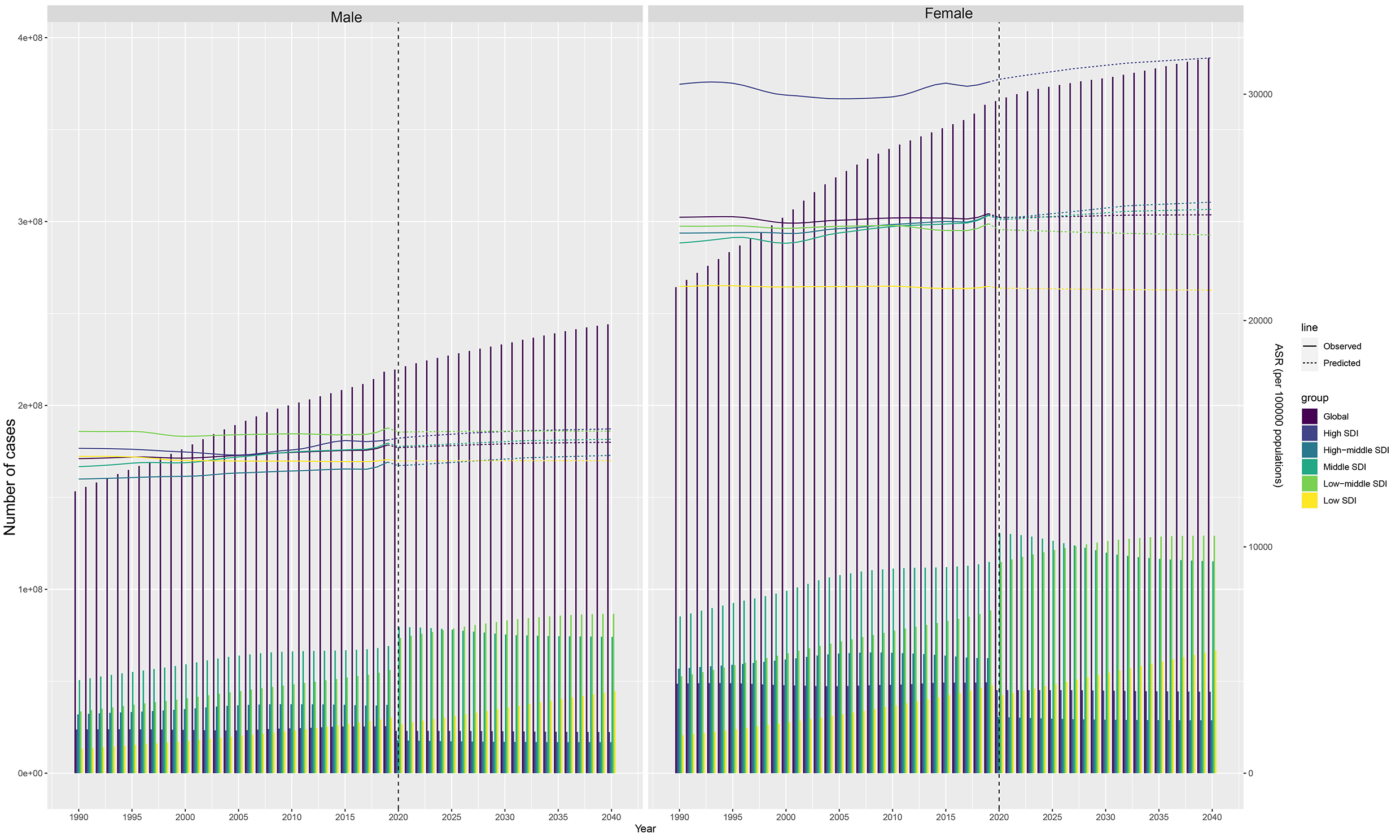

Supplement: Supplementary file 6 — Additional file 6: Fig S6. Future Forecasts of GBD in Migraine Prevalence. [file 10194_2023_1659_MOESM6_ESM.jpg]

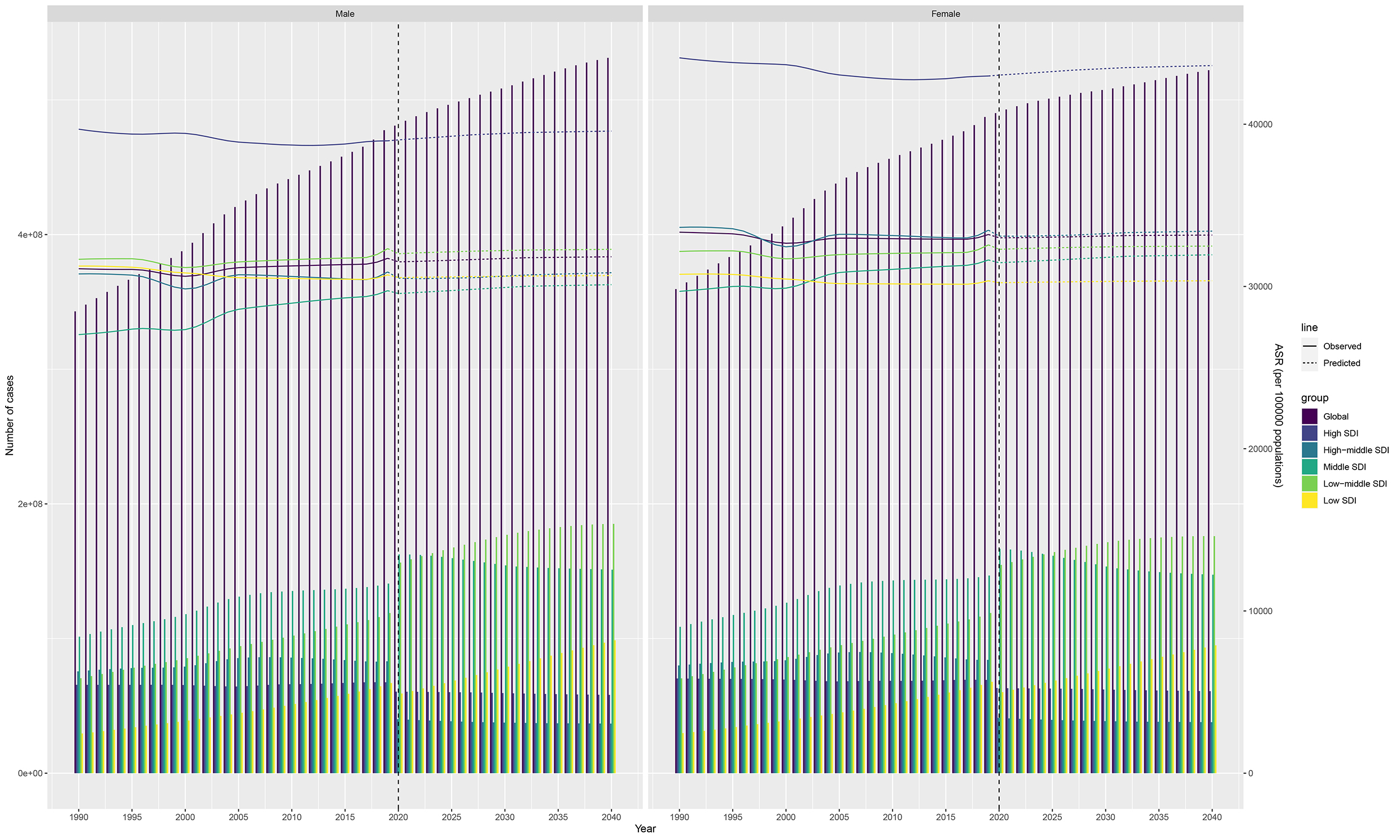

Supplement: Supplementary file 7 — Additional file 7: Fig S7. Future Forecasts of GBD in TTH Prevalence. [file 10194_2023_1659_MOESM7_ESM.jpg]
