# Supplementary material for: Global, regional, and national epidemiology of migraine and tension-type headache in youths and young adults aged 15–39 years from 1990 to 2019: findings from the global burden of disease study 2019
Source: J Headache Pain. 2023 Sep 18;24(1):126. doi: 10.1186/s10194-023-01659-1 (PMC10506184; doi:10.1186/s10194-023-01659-1)
Supplement: Supplementary file 8 — Additional file 8: Table S1. Prevalence of Migraine Between 1990 and 2019 in 15 to 39 years at the Global and Regional Level. [file 10194_2023_1659_MOESM8_ESM.docx]

| **TableS1 Prevalence of Migraine Between 1990 and 2019 in 15 to 39 years at the Global and Regional Level** | | | | | |
| --- | --- | --- | --- | --- | --- |
|  | **1990** | | **2019** | |  |
| **Location** | **Number__95%UI** | **ASR** | **Number__95%UI** | **ASR** | **EAPC_95%CI** |
| Global | 417624741.1 (349971811.1-499587739) | 19039.2 (15955-22775.9) | 581761847.2 (488309998.1-696291713.7) | 19602 (16453.2-23461) | 0.07 (0.06-0.09) |
| High SDI | 72460221.7 (60567734.9-86478983.6) | 22429 (18747.9-26768.3) | 74894713.4 (62659304.8-89532525.3) | 22605.9 (18912.9-27024.2) | -0.01 (-0.05-0.04) |
| High-middle SDI | 88791450.8 (74523982.9-106133380.9) | 18363.8 (15413-21950.4) | 99729417.3 (83404761.5-118944560.7) | 19300.5 (16141.2-23019.2) | 0.14 (0.13-0.16) |
| Middle SDI | 135930245.3 (114202168.7-162206250.2) | 18206.7 (15296.4-21726.1) | 184019869.2 (154215461.1-219685296.3) | 19679.3 (16492-23493.5) | 0.24 (0.22-0.25) |
| Low-middle SDI | 86238213.8 (72037548.9-103468180.5) | 19364.7 (16176-23233.7) | 144619858.3 (121506408.9-172919905.4) | 19668.3 (16524.9-23517.1) | 0.01 (0-0.03) |
| Low SDI | 33969324.5 (28201308.6-40824127.1) | 17526.8 (14550.8-21063.6) | 78152586.7 (65245280.7-94254819) | 17456 (14573.1-21052.6) | -0.02 (-0.03--0.02) |
| Andean Latin America | 2002998.5 (1663714.5-2423510.1) | 12961.4 (10765.9-15682.5) | 3576524.6 (2899284.4-4393549.4) | 13927.8 (11290.5-17109.5) | 0.29 (0.23-0.35) |
| Australasia | 1590327.8 (1299636.8-1935074.7) | 19502.3 (15937.5-23729.9) | 1911178.4 (1556428.7-2330457.8) | 19667.5 (16016.9-23982.3) | 0.01 (0-0.01) |
| Caribbean | 2758373.6 (2243450.6-3413875.7) | 18592.5 (15121.7-23010.8) | 3371678 (2754466.7-4135180) | 18598.9 (15194.2-22810.6) | -0.01 (-0.01--0.01) |
| Central Asia | 5240596.3 (4265521.1-6409968.7) | 18405.1 (14980.6-22512) | 7034580.4 (5741020.3-8608437.5) | 18568 (15153.6-22722.3) | 0 (-0.02-0.02) |
| Central Europe | 8568935.8 (7125229.8-10277418.9) | 18620.4 (15483.2-22333) | 6720792.6 (5605697.1-8079678.2) | 18871.8 (15740.7-22687.5) | 0.07 (0.05-0.09) |
| Central Latin America | 12692312.8 (10602312.1-15160660.7) | 18607.4 (15543.4-22226.1) | 19131220.2 (15851096.9-22995676.1) | 18944.7 (15696.6-22771.5) | 0.07 (0.06-0.08) |
| Central Sub-Saharan Africa | 3523916.8 (2847288.1-4308644.9) | 16973.9 (13714.7-20753.7) | 8799150.9 (7116631.1-10765649.1) | 16986.9 (13738.8-20783.3) | 0 (0-0.01) |
| East Asia | 82011006.3 (68434690.8-97329820.2) | 14464.6 (12070.1-17166.4) | 83238673.5 (69184689.6-99917864.4) | 16140.9 (13415.7-19375.2) | 0.29 (0.26-0.33) |
| Eastern Europe | 16204626.3 (13650028.3-19345670.9) | 18888.2 (15910.6-22549.4) | 13166808 (11076831.4-15760355) | 19185.3 (16140-22964.4) | 0.1 (0.07-0.13) |
| Eastern Sub-Saharan Africa | 8461320.5 (6987349.2-10161717.5) | 12001.6 (9910.9-14413.5) | 20204840.1 (16643264-24335583.8) | 12116.3 (9980.5-14593.4) | 0.07 (0.05-0.08) |
| High-income Asia Pacific | 10852303.8 (9005159.5-12974705.9) | 16066.2 (13331.6-19208.3) | 8384696.9 (6989493.1-9956075.7) | 15958 (13302.6-18948.7) | -0.04 (-0.05--0.03) |
| High-income North America | 28385933.5 (23870052.7-33322575.9) | 25103.9 (21110.2-29469.8) | 29903741.6 (25043559.3-35827543.7) | 24610.4 (20610.5-29485.6) | -0.07 (-0.16-0.03) |
| North Africa and Middle East | 29037116 (24042258.2-35170834.2) | 21385 (17706.4-25902.3) | 56011384 (46743385.1-67159564.7) | 21657.7 (18074.1-25968.4) | 0.05 (0.03-0.06) |
| Oceania | 489481.1 (395984.1-597961.5) | 18593.8 (15042.1-22714.6) | 1022009.1 (830207.7-1243315.4) | 18780.1 (15255.6-22846.7) | 0.02 (0.02-0.03) |
| South Asia | 86768829.2 (72755767.5-103058854.9) | 20086.8 (16842.8-23857.9) | 154490169.8 (130296054.6-182464065.6) | 20093.2 (16946.5-23731.6) | -0.08 (-0.1--0.05) |
| Southeast Asia | 43562228.8 (36292680.1-52048354.1) | 22134.3 (18440.6-26446.2) | 59460477.8 (49406504.5-71297063.5) | 21893.1 (18191.3-26251.3) | -0.05 (-0.07--0.03) |
| Southern Latin America | 2995371.3 (2481143.5-3588974.9) | 15694.4 (13000-18804.6) | 4191998.3 (3429197.4-5126592.2) | 16482.9 (13483.6-20157.7) | 0.23 (0.19-0.26) |
| Southern Sub-Saharan Africa | 3861236.2 (3212762.9-4626879.1) | 17610.4 (14652.8-21102.3) | 6005728.3 (4993052.2-7199685.6) | 17830.7 (14824.2-21375.6) | 0.03 (0.02-0.04) |
| Tropical Latin America | 15953260.3 (13334637.5-19324552.7) | 24804.3 (20732.9-30046) | 22672890.5 (18842076.2-27207662.6) | 25448 (21148.3-30537.8) | 0.13 (0.07-0.19) |
| Western Europe | 37817458.7 (31321689.2-45596663.4) | 26250.2 (21741.3-31649.9) | 35275646.6 (29519314.5-42263963.2) | 26919.8 (22527-32252.7) | 0.07 (0.03-0.11) |
| Western Sub-Saharan Africa | 14847107.5 (12318260.1-17875207.4) | 20880.5 (17324-25139.1) | 37187657.7 (30870824.8-44755999.7) | 20758.2 (17232.1-24982.9) | -0.01 (-0.02-0) |
| Abbreviations: EAPC, estimated annual percentage change; SDI, Sociodemographic Index; UI, uncertainty interval. | | | | | |
