# Supplementary material for: Global, regional, and national epidemiology of migraine and tension-type headache in youths and young adults aged 15–39 years from 1990 to 2019: findings from the global burden of disease study 2019
Source: J Headache Pain. 2023 Sep 18;24(1):126. doi: 10.1186/s10194-023-01659-1 (PMC10506184; doi:10.1186/s10194-023-01659-1)
Supplement: Supplementary file 9 — Additional file 9: Table S2. Prevalence of Tension-Type Headache Between 1990 and 2019 in 15 to 39 years at the Global and Regional Level. [file 10194_2023_1659_MOESM9_ESM.docx]

| **TableS2 Prevalence of Tension-Type Headache Between 1990 and 2019 in 15 to 39 years at the Global and Regional Level** | | | | | |
| --- | --- | --- | --- | --- | --- |
|  | 1990 | | 2019 | |  |
| Location | Number_95%UI | ASR | Number_95%UI | ASR | EAPC_95%CI |
| Global | 702507085.3 (588427399.4-839701792.4) | 32026.9 (26826-38281.5) | 964808567.1 (809582531.8-1155235337.2) | 32508.5 (27278.3-38924.8) | 0.04 (0.02-0.06) |
| High SDI | 136018223.4 (114858793.7-160432314.7) | 42102.4 (35552.8-49659.5) | 136761919.1 (115294101.5-161328346.7) | 41279.7 (34799.9-48694.8) | -0.1 (-0.12--0.08) |
| High-middle SDI | 155680683.5 (131221836-185460921.3) | 32197.8 (27139.2-38356.9) | 167282046.1 (139987562.5-198945303.5) | 32373.9 (27091.6-38501.6) | 0 (-0.04-0.04) |
| Middle SDI | 210136691.6 (174947733.7-251440966) | 28146 (23432.8-33678.4) | 287577250 (240243873.1-344193628.8) | 30753.9 (25692-36808.6) | 0.32 (0.29-0.34) |
| Low-middle SDI | 141020579 (117102663.3-168516197.7) | 31666 (26295.3-37840.1) | 237862300.1 (198586196.6-284798854.6) | 32349.3 (27007.8-38732.7) | 0.04 (0.01-0.06) |
| Low SDI | 59276105.6 (48513175-71725123.3) | 30584.1 (25030.9-37007.3) | 134776962.6 (110855745.6-162899766) | 30103.5 (24760.5-36385) | -0.09 (-0.11--0.07) |
| Andean Latin America | 3797972.9 (3077387.6-4660321.6) | 24576.6 (19913.7-30156.9) | 6632672.7 (5321238.5-8247450) | 25829.2 (20722.1-32117.5) | 0.22 (0.18-0.27) |
| Australasia | 2903811.2 (2389759.4-3521329.1) | 35609.6 (29305.8-43182.3) | 3474934.9 (2865008-4211702.5) | 35759.8 (29483.2-43341.8) | 0 (-0.01-0.01) |
| Caribbean | 4564817.9 (3682745.4-5690931.1) | 30768.6 (24823.1-38359.1) | 5600299.3 (4532685-6931695.3) | 30892.5 (25003.3-38236.7) | 0.01 (0-0.01) |
| Central Asia | 11710913.7 (9739368.4-14170881.1) | 41129 (34204.9-49768.5) | 15674631.3 (12991538.3-18990100.7) | 41373.7 (34291.6-50125) | 0.01 (0-0.02) |
| Central Europe | 18610808.7 (15550390-22207732.2) | 40441.5 (33791.2-48257.7) | 14504218.9 (12125576.1-17375594.6) | 40727.5 (34048.3-48790.2) | 0.03 (0.02-0.04) |
| Central Latin America | 21599944 (17702361.2-26194629) | 31666.4 (25952.4-38402.4) | 32110073.5 (26437732.6-38805225.7) | 31797.1 (26180-38427) | 0.01 (0.01-0.01) |
| Central Sub-Saharan Africa | 6372787 (5131863.4-7930112.7) | 30696.2 (24719-38197.5) | 15918762.1 (12827301.6-19773242.7) | 30731.5 (24763.4-38172.6) | 0 (0-0) |
| East Asia | 121239162.9 (100784961.4-145055719.5) | 21383.4 (17775.8-25584) | 121380145.4 (100582481.4-146574254.9) | 23536.9 (19504.1-28422.4) | 0.46 (0.36-0.56) |
| Eastern Europe | 38125000 (32452511.3-44729569.9) | 44438.7 (37826.9-52137.1) | 30868788.6 (26070630.6-36016012.7) | 44978.9 (37987.5-52478.9) | 0.03 (0-0.06) |
| Eastern Sub-Saharan Africa | 18094528.9 (14477204.1-22242854.6) | 25665.5 (20534.6-31549.5) | 41654591.1 (33469875.6-50768082.8) | 24979.1 (20071-30444.2) | -0.12 (-0.15--0.1) |
| High-income Asia Pacific | 26434627 (22240928.8-31231897.2) | 39135 (32926.4-46237.1) | 21254539.9 (17991527.6-24821876.4) | 40452.2 (34242-47241.7) | 0.14 (0.13-0.15) |
| High-income North America | 53641143.4 (45439575.2-62677985.5) | 47439.1 (40185.8-55431.1) | 55786522.5 (47489624.3-65564427.6) | 45911.6 (39083.4-53958.7) | -0.17 (-0.21--0.14) |
| North Africa and Middle East | 40184596.9 (32739839.1-49387797.7) | 29594.7 (24111.9-36372.6) | 78194087.1 (63812803.2-95202141.4) | 30235 (24674.3-36811.5) | 0.02 (0-0.05) |
| Oceania | 757991.2 (608543.3-948735.2) | 28793.6 (23116.6-36039.4) | 1575549.3 (1266839.4-1969940.4) | 28951.7 (23279-36198.9) | 0.01 (0.01-0.02) |
| South Asia | 145682885.9 (122866876.1-173295558) | 33725.3 (28443.4-40117.6) | 260079112.3 (219342370.5-308660465.3) | 33826.3 (28528-40144.9) | -0.07 (-0.11--0.03) |
| Southeast Asia | 64622781.4 (53683343.1-78800797.1) | 32835.3 (27276.9-40039.3) | 90086185.1 (75004401.3-109374077.6) | 33169.4 (27616.4-40271.1) | 0.03 (0.03-0.03) |
| Southern Latin America | 6600659.3 (5423542.1-8100868.4) | 34584.4 (28416.9-42444.8) | 8928645.3 (7285405.8-10894484.7) | 35107.3 (28646.1-42837) | 0.06 (0.06-0.07) |
| Southern Sub-Saharan Africa | 7012449.2 (5802915.7-8465209.1) | 31982.4 (26466-38608.2) | 10974022.4 (9132409.2-13155355.5) | 32581.4 (27113.7-39057.7) | 0.06 (0.05-0.07) |
| Tropical Latin America | 24119827.4 (20350001.6-28470681) | 37501.8 (31640.4-44266.6) | 32640423 (27475246.5-38440091.6) | 36635.6 (30838.2-43145.1) | -0.18 (-0.22--0.13) |
| Western Europe | 63001344.1 (52841577.3-75075012.9) | 43731 (36678.8-52111.7) | 58546978.1 (48687048.1-69554778.3) | 44678.7 (37154.4-53079.1) | 0.06 (0.05-0.07) |
| Western Sub-Saharan Africa | 23429032.3 (19414015.9-28229094.6) | 32949.8 (27303.2-39700.4) | 58923384 (48819318.4-71086204.8) | 32891.1 (27251-39680.4) | -0.04 (-0.06--0.02) |

Abbreviations: EAPC, estimated annual percentage change; SDI, Sociodemographic Index; UI, uncertainty interval.
