# Supplementary material for: Global, regional, and national epidemiology of migraine and tension-type headache in youths and young adults aged 15–39 years from 1990 to 2019: findings from the global burden of disease study 2019
Source: J Headache Pain. 2023 Sep 18;24(1):126. doi: 10.1186/s10194-023-01659-1 (PMC10506184; doi:10.1186/s10194-023-01659-1)
Supplement: Supplementary file 10 — Additional file 10: Table S3. DALYs of Migraine and TTH Between 1990 and 2019 in 15 to 39 years at the Global and Regional Level. [file 10194_2023_1659_MOESM10_ESM.docx]

| **TableS3.DALYs of Migraine and TTH Between 1990 and 2019 in 15 to 39 years at the Global and Regional Level** | | | | | |
| --- | --- | --- | --- | --- | --- |
| **migraine** | 1990 | | 2019 | |  |
| location | Number_95%UI | ASR | Number_95%UI | ASR | EAPC_95%CI |
| Global | 15424064.1 (1814454.8-35653837.1) | 703.2 (82.7-1625.4) | 21529090.4 (2541050.5-49944979.9) | 725.4 (85.6-1682.9) | 0.08 (0.07-0.1) |
| High SDI | 2638694.3 (313738.4-6117539.9) | 816.8 (97.1-1893.6) | 2734053.3 (334005.1-6307920.8) | 825.2 (100.8-1904) | 0.01 (-0.03-0.05) |
| High-middle SDI | 3332280.1 (478202.2-7603100.2) | 689.2 (98.9-1572.5) | 3745745.3 (517724.9-8670309.5) | 724.9 (100.2-1678) | 0.16 (0.14-0.17) |
| Middle SDI | 5070200.1 (572083.1-11809798.7) | 679.1 (76.6-1581.8) | 6873547.7 (789609.5-16273959.8) | 735.1 (84.4-1740.4) | 0.25 (0.23-0.26) |
| Low-middle SDI | 3135735.4 (321411.9-7389000.7) | 704.1 (72.2-1659.2) | 5289638.4 (551876.2-12373959.4) | 719.4 (75.1-1682.9) | 0.04 (0.03-0.05) |
| Low SDI | 1238411.3 (147761.5-2881409.7) | 639 (76.2-1486.7) | 2873183.4 (345952.2-6654632.8) | 641.7 (77.3-1486.4) | 0.01 (0-0.02) |
| Andean Latin America | 74520.5 (11522.6-169831.3) | 482.2 (74.6-1099) | 133625.5 (20081.4-305831) | 520.4 (78.2-1191) | 0.31 (0.25-0.37) |
| Australasia | 57259.4 (7441.5-132417.1) | 702.2 (91.3-1623.8) | 68832.8 (9080.7-158749.3) | 708.3 (93.4-1633.7) | 0.01 (0-0.01) |
| Caribbean | 102317.6 (11252.5-246054.9) | 689.7 (75.8-1658.5) | 125093.9 (13960.9-300106.5) | 690 (77-1655.5) | -0.01 (-0.01-0) |
| Central Asia | 191158.9 (26320.3-444290.6) | 671.4 (92.4-1560.4) | 257174.9 (36712-595384.4) | 678.8 (96.9-1571.5) | 0.01 (-0.01-0.03) |
| Central Europe | 316106.1 (51567.8-718299) | 686.9 (112.1-1560.9) | 249138.1 (42471.7-571146.5) | 699.6 (119.3-1603.8) | 0.1 (0.08-0.12) |
| Central Latin America | 474473.7 (52887.5-1119576.6) | 695.6 (77.5-1641.3) | 715746.1 (81292.3-1702693.7) | 708.8 (80.5-1686.1) | 0.07 (0.06-0.08) |
| Central Sub-Saharan Africa | 127924.3 (17525.7-297035.3) | 616.2 (84.4-1430.8) | 322667.6 (43600.5-754118.8) | 622.9 (84.2-1455.8) | 0.04 (0.03-0.05) |
| East Asia | 3066848.7 (401677.5-7033697.7) | 540.9 (70.8-1240.6) | 3111009.3 (388972.8-7250261.4) | 603.3 (75.4-1405.9) | 0.3 (0.27-0.33) |
| Eastern Europe | 636023.2 (141309-1378132.3) | 741.4 (164.7-1606.4) | 519706.4 (121569.1-1141285.8) | 757.3 (177.1-1663) | 0.14 (0.11-0.17) |
| Eastern Sub-Saharan Africa | 315691.5 (54684.7-712997.4) | 447.8 (77.6-1011.3) | 757633.2 (129318.6-1719652.5) | 454.3 (77.5-1031.2) | 0.09 (0.07-0.11) |
| High-income Asia Pacific | 395274.8 (59324.2-923684.2) | 585.2 (87.8-1367.5) | 308142.3 (49800.6-719101.6) | 586.5 (94.8-1368.6) | 0 (-0.01-0.01) |
| High-income North America | 1027088.2 (110168.8-2394918.8) | 908.3 (97.4-2118) | 1075763.7 (116797.7-2544021.1) | 885.3 (96.1-2093.7) | -0.08 (-0.17-0.02) |
| North Africa and Middle East | 1131478.5 (167845.1-2696383.6) | 833.3 (123.6-1985.8) | 2185639.1 (340709.4-5121958.6) | 845.1 (131.7-1980.5) | 0.06 (0.04-0.07) |
| Oceania | 17946.3 (1800.2-43912.8) | 681.7 (68.4-1668.1) | 37543.3 (3811.6-91419.1) | 689.9 (70-1679.9) | 0.03 (0.03-0.04) |
| South Asia | 3109259.1 (294340.3-7273875.9) | 719.8 (68.1-1683.9) | 5570590.8 (535111.6-13023883.2) | 724.5 (69.6-1693.9) | -0.05 (-0.07--0.02) |
| Southeast Asia | 1625437.6 (139366.4-4079853.4) | 825.9 (70.8-2073) | 2229495.2 (202801.4-5555201.7) | 820.9 (74.7-2045.4) | -0.03 (-0.05--0.01) |
| Southern Latin America | 110305.2 (16297.3-254699.6) | 577.9 (85.4-1334.5) | 154259.6 (23186.5-352088.6) | 606.5 (91.2-1384.4) | 0.22 (0.19-0.26) |
| Southern Sub-Saharan Africa | 142600.5 (19322.1-325763.9) | 650.4 (88.1-1485.7) | 221006.2 (31446.1-504513.9) | 656.2 (93.4-1497.9) | 0.02 (0-0.05) |
| Tropical Latin America | 582582.8 (46494.1-1433005.7) | 905.8 (72.3-2228.1) | 829001.1 (68208.1-2026738.3) | 930.5 (76.6-2274.8) | 0.14 (0.09-0.19) |
| Western Europe | 1375274.4 (147668.1-3226091.8) | 954.6 (102.5-2239.3) | 1285221.2 (140099.9-3009583.2) | 980.8 (106.9-2296.7) | 0.09 (0.05-0.13) |
| Western Sub-Saharan Africa | 544492.8 (61196.7-1281152.8) | 765.8 (86.1-1801.8) | 1371800 (154463.8-3194713.9) | 765.7 (86.2-1783.3) | 0.01 (0.01-0.02) |
|  |  |  |  |  |  |
| **TTH** |  |  |  |  |  |
| Global | 1446380.2 (386965.6-5450782.5) | 65.9 (17.6-248.5) | 1992931.2 (529490.2-7415829.4) | 67.2 (17.8-249.9) | 0.05 (0.03-0.06) |
| High SDI | 265332.5 (65035-941820.6) | 82.1 (20.1-291.5) | 274441.3 (69173.9-944353.5) | 82.8 (20.9-285) | -0.01 (-0.02-0.01) |
| High-middle SDI | 352068 (99713.8-1185764.4) | 72.8 (20.6-245.2) | 379294.4 (107972-1245256.4) | 73.4 (20.9-241) | 0.04 (0.01-0.07) |
| Middle SDI | 441588.6 (121856-1660354.6) | 59.1 (16.3-222.4) | 606396.7 (163865.3-2242322.2) | 64.8 (17.5-239.8) | 0.3 (0.29-0.31) |
| Low-middle SDI | 268423.3 (69359.1-1087410.9) | 60.3 (15.6-244.2) | 458784.9 (117057.4-1842017.9) | 62.4 (15.9-250.5) | 0.08 (0.06-0.1) |
| Low SDI | 118218.5 (31281-468927.4) | 61 (16.1-241.9) | 272862.6 (72739.1-1056086.4) | 60.9 (16.2-235.9) | -0.03 (-0.05--0.02) |
| Andean Latin America | 8295 (2372.3-33259.6) | 53.7 (15.4-215.2) | 14435.3 (4064.3-56783.3) | 56.2 (15.8-221.1) | 0.18 (0.17-0.2) |
| Australasia | 5875.8 (1510.7-21588.5) | 72.1 (18.5-264.7) | 7142.4 (1821.7-25378.6) | 73.5 (18.7-261.2) | 0.02 (0-0.04) |
| Caribbean | 8959.5 (2339.2-36898.5) | 60.4 (15.8-248.7) | 11176 (2914.9-44619.8) | 61.6 (16.1-246.1) | 0.04 (0.02-0.05) |
| Central Asia | 22823.7 (5418.2-81337.4) | 80.2 (19-285.7) | 31048.7 (7602.3-110657.9) | 82 (20.1-292.1) | 0.03 (0-0.07) |
| Central Europe | 40723.7 (11020.4-134886) | 88.5 (23.9-293.1) | 32488 (8959.4-106540.4) | 91.2 (25.2-299.2) | 0.15 (0.12-0.18) |
| Central Latin America | 42517.4 (11161.6-173493.8) | 62.3 (16.4-254.3) | 64578.1 (17431-254668.7) | 63.9 (17.3-252.2) | 0.07 (0.07-0.08) |
| Central Sub-Saharan Africa | 13037.9 (3533.7-51825.6) | 62.8 (17-249.6) | 32893.3 (8988.9-128515.4) | 63.5 (17.4-248.1) | 0.04 (0.03-0.05) |
| East Asia | 279748.1 (82420.7-1066340.6) | 49.3 (14.5-188.1) | 274375.3 (79618.9-975630.1) | 53.2 (15.4-189.2) | 0.25 (0.22-0.28) |
| Eastern Europe | 97865.8 (29538.6-306009.2) | 114.1 (34.4-356.7) | 80765.1 (24747.4-251834) | 117.7 (36.1-366.9) | 0.2 (0.16-0.25) |
| Eastern Sub-Saharan Africa | 39926.7 (11467.8-149578) | 56.6 (16.3-212.2) | 93058.1 (26572.4-336101) | 55.8 (15.9-201.6) | -0.06 (-0.09--0.04) |
| High-income Asia Pacific | 50678.7 (12229.1-195889.4) | 75 (18.1-290) | 41326.9 (10093.8-152587.7) | 78.7 (19.2-290.4) | 0.21 (0.18-0.23) |
| High-income North America | 99231.3 (22912.9-363926.8) | 87.8 (20.3-321.8) | 103939.5 (24244.5-383464.9) | 85.5 (20-315.6) | -0.16 (-0.19--0.13) |
| North Africa and Middle East | 110614.8 (36402.9-322906.3) | 81.5 (26.8-237.8) | 220379.9 (72001.8-620813) | 85.2 (27.8-240) | 0.15 (0.14-0.16) |
| Oceania | 1450 (364.8-6029.8) | 55.1 (13.9-229.1) | 3048.2 (780.4-12525.4) | 56 (14.3-230.2) | 0.05 (0.04-0.06) |
| South Asia | 261889.3 (63999-1098872.1) | 60.6 (14.8-254.4) | 471509.7 (112341.7-1986731.2) | 61.3 (14.6-258.4) | -0.03 (-0.07-0) |
| Southeast Asia | 119870.2 (29332.3-499468.3) | 60.9 (14.9-253.8) | 171061.4 (42156.6-696974.6) | 63 (15.5-256.6) | 0.11 (0.11-0.12) |
| Southern Latin America | 13399.7 (3431.8-48810.3) | 70.2 (18-255.7) | 18389.2 (4659-64029.3) | 72.3 (18.3-251.8) | 0.1 (0.08-0.11) |
| Southern Sub-Saharan Africa | 14753.6 (4071.4-55693.8) | 67.3 (18.6-254) | 23409.8 (6491.9-87637.1) | 69.5 (19.3-260.2) | 0.1 (0.07-0.13) |
| Tropical Latin America | 42716.8 (9625.4-182878.4) | 66.4 (15-284.3) | 59900.7 (14107.1-245755.9) | 67.2 (15.8-275.8) | 0.01 (-0.06-0.08) |
| Western Europe | 124431 (30056.4-444320.2) | 86.4 (20.9-308.4) | 117260.9 (28411.9-420843.4) | 89.5 (21.7-321.2) | 0.14 (0.11-0.17) |
| Western Sub-Saharan Africa | 47571.2 (12968.7-187556.4) | 66.9 (18.2-263.8) | 120744.7 (32280.3-464023.8) | 67.4 (18-259) | 0.02 (0.01-0.03) |

Abbreviations: EAPC, estimated annual percentage change; SDI, Sociodemographic Index; UI, uncertainty interval.
