# Supplementary material for: Global, regional, and national epidemiology of migraine and tension-type headache in youths and young adults aged 15–39 years from 1990 to 2019: findings from the global burden of disease study 2019
Source: J Headache Pain. 2023 Sep 18;24(1):126. doi: 10.1186/s10194-023-01659-1 (PMC10506184; doi:10.1186/s10194-023-01659-1)
Supplement: Supplementary file 11 — Additional file 11: Table S4. Prevalence of Migraine Between 1990 and 2019 in 15 to 39 years at the 204 Countries Level. [file 10194_2023_1659_MOESM11_ESM.docx]

| **TableS4 Prevalence of migraine Between 1990 and 2019 in 15 to 39 years at the 204 Countries Level** | | | | | |
| --- | --- | --- | --- | --- | --- |
|  | 1990 | | 2019 | |  |
| Location | Number_95%UI | ASR | Number_95%UI | ASR | EAPC_95%CI |
| Mexico | 6647751.2 (5570203.7-7948515.9) | 18650.5 (15627.4-22299.9) | 9561528.6 (7968183.5-11433278) | 19054.4 (15879.2-22784.5) | 0.09 (0.08-0.11) |
| Haiti | 456259.4 (370970.6-562230.5) | 18775 (15265.4-23135.7) | 998939.1 (813535.2-1229186.2) | 18794.6 (15306.3-23126.6) | 0.01 (0.01-0.02) |
| Viet Nam | 6086186.5 (4943052.1-7497325.6) | 21337.7 (17330-26285) | 8538719.4 (6961899.5-10549225.3) | 21645.3 (17648.2-26741.9) | 0.03 (0.01-0.06) |
| Bhutan | 50238.1 (40412.9-61671.9) | 19320.7 (15542.1-23717.9) | 69203 (56115.1-84227.3) | 19705.8 (15979-23984) | 0.05 (0.04-0.06) |
| Jamaica | 182777.6 (148193.3-225824.3) | 18601.4 (15081.7-22982.2) | 222002 (181022-272582.8) | 18601.1 (15167.5-22839.2) | -0.01 (-0.02--0.01) |
| Nicaragua | 276017.6 (224717.6-340873.5) | 18744.4 (15260.6-23148.8) | 521602.3 (427070.1-637355.1) | 18593.9 (15224.1-22720.2) | -0.03 (-0.03--0.03) |
| Kyrgyzstan | 331011.2 (268759.5-404684.8) | 18347 (14896.6-22430.6) | 492366.5 (401540.7-603685.6) | 18489.1 (15078.4-22669.3) | 0 (-0.03-0.02) |
| Georgia | 397707.2 (324946.5-486054.1) | 18677.4 (15260.4-22826.4) | 220980.4 (180497.6-271410.5) | 18675.5 (15254.2-22937.4) | -0.03 (-0.05--0.01) |
| Lebanon | 259973.3 (213052-318040.5) | 21589.9 (17693.2-26412.1) | 449159.2 (369225.9-546303.5) | 21879.1 (17985.4-26611.1) | 0.04 (0.03-0.05) |
| Kazakhstan | 1256487.2 (1024337.6-1536264.2) | 18491.3 (15074.8-22608.7) | 1304508 (1064700.6-1598679.5) | 18773.8 (15322.6-23007.4) | 0.04 (0.01-0.06) |
| Namibia | 95019.1 (76767.8-116046.9) | 16993 (13729-20753.6) | 175649.2 (142235.6-214249.7) | 17203.7 (13931.1-20984.4) | 0.03 (0.02-0.04) |
| Republic of Korea | 3803292.2 (3113650.3-4687824.7) | 18054.2 (14780.4-22253) | 2860088.6 (2356360.4-3435244.2) | 16501.3 (13595-19819.7) | -0.42 (-0.47--0.37) |
| Timor-Leste | 67135.4 (54541.3-82656.8) | 21102.4 (17143.7-25981.1) | 112388 (90945-138442) | 20907.3 (16918.3-25754.1) | -0.05 (-0.06--0.04) |
| China | 79172734.6 (66072301.9-94002586.2) | 14408.2 (12024.1-17107) | 80289498.4 (66739935-96356726.8) | 16131.7 (13409.3-19359.9) | 0.3 (0.27-0.34) |
| Eritrea | 140733.5 (114440-171697.3) | 12156.1 (9885-14830.7) | 349654.1 (285082-427262) | 12162.2 (9916.1-14861.6) | 0 (0-0.01) |
| Iceland | 26745.2 (21743.5-32762.7) | 25746.6 (20931.7-31539.4) | 30868.4 (25105.5-37889.7) | 25739.8 (20934.4-31594.6) | -0.01 (-0.03-0) |
| Panama | 186997 (152630.4-230029.1) | 18504.1 (15103.4-22762.4) | 295857.5 (242425.4-361809.2) | 18509.6 (15166.8-22635.8) | 0 (0-0.01) |
| Serbia | 631551.2 (521998.2-764623.9) | 18365.3 (15179.5-22235) | 519806.5 (429318.4-629834.6) | 18408.6 (15204-22305.2) | 0.04 (0.03-0.05) |
| India | 68843969.6 (57729472.8-81491506.5) | 20202 (16940.5-23913.4) | 120445685 (101406182.2-142027322.6) | 20203.3 (17009.7-23823.4) | -0.1 (-0.13--0.07) |
| Libya | 341940.8 (279083.2-418929.9) | 20869.2 (17032.9-25567.9) | 652719.1 (536580.6-792249.5) | 21569.2 (17731.4-26180) | 0.1 (0.08-0.11) |
| South Africa | 2836176.2 (2378379.4-3374121) | 17836.8 (14957.7-21220) | 4330898.9 (3638473.5-5154007.4) | 18083.1 (15192-21519.9) | 0.03 (0.01-0.04) |
| Democratic People's Republic of Korea | 1303186.9 (1062992.2-1603122.2) | 15787.1 (12877.3-19420.6) | 1571245.3 (1276609.9-1936583.8) | 15639.2 (12706.6-19275.6) | -0.11 (-0.15--0.08) |
| Uruguay | 187422.2 (153380.8-228313.5) | 16501.4 (13504.3-20101.6) | 200423.3 (164091.1-244693.7) | 16595.1 (13586.8-20260.7) | 0.02 (0.02-0.03) |
| Japan | 6857667.3 (5696305.3-8134810.4) | 15290.6 (12701.1-18138.3) | 5193603.6 (4340061.7-6152049.7) | 15795.6 (13199.7-18710.6) | 0.15 (0.12-0.19) |
| Poland | 2787834.4 (2344185-3290874.4) | 19280.4 (16212.1-22759.3) | 2472579.1 (2076074.8-2930458.8) | 19563.4 (16426.2-23186.2) | 0.09 (0.05-0.12) |
| Saint Vincent and the Grenadines | 8416.9 (6823.6-10400) | 18335.6 (14864.9-22655.8) | 7753.5 (6317.9-9506) | 18470.1 (15050.2-22645) | 0.04 (0.03-0.04) |
| Australia | 1311774.6 (1058616.5-1607654.4) | 19369.1 (15631.1-23738) | 1625273.5 (1315650.4-1992417.7) | 19564.9 (15837.7-23984.6) | 0.01 (0-0.02) |
| Cook Islands | 1436.9 (1164.2-1753.1) | 18594 (15065.8-22686.8) | 1129.7 (920.6-1372.9) | 19160.6 (15614.7-23285.4) | 0.1 (0.09-0.11) |
| Liberia | 137864.8 (112956.2-167972.3) | 20651.4 (16920.2-25161.3) | 404612.4 (332026.6-493606) | 20382.8 (16726.3-24866) | -0.03 (-0.04--0.01) |
| Greenland | 6190.4 (5064.6-7504.3) | 23403.3 (19147.1-28370.3) | 4873.9 (4018.3-5894.7) | 23936 (19734-28949.2) | 0.01 (-0.02-0.04) |
| Tajikistan | 385463.7 (313240.4-471983.1) | 18218.5 (14805-22307.8) | 744576.5 (607672.3-911570.4) | 18366.6 (14989.6-22485.9) | -0.01 (-0.03-0.01) |
| Fiji | 60599.9 (49143.2-73756.1) | 18781.1 (15230.4-22858.4) | 67842.5 (55302.4-82370.5) | 18930.3 (15431.2-22984.1) | 0.04 (0.02-0.05) |
| Bermuda | 4822.5 (3943.7-5927.2) | 18734.8 (15320.8-23026.6) | 3406.6 (2803.6-4187.6) | 18774.1 (15450.9-23078.7) | 0 (0-0.01) |
| Israel | 491527.4 (399651.5-603490.8) | 25712.7 (20906.5-31569.7) | 834126.2 (678351.7-1020055.8) | 25706.3 (20905.6-31436.3) | 0.01 (0.01-0.02) |
| United States Virgin Islands | 7456.3 (6076.8-9157) | 18820.3 (15338.3-23112.8) | 5709.1 (4678.7-6999.9) | 18830.9 (15432.2-23088.7) | 0 (-0.01-0.01) |
| Pakistan | 7966858.5 (6599538.1-9443871.8) | 19456.5 (16117.3-23063.6) | 17659063.8 (14775625.8-21042972.4) | 19306.6 (16154.1-23006.2) | -0.03 (-0.04--0.02) |
| Guam | 11799.9 (9612-14360.8) | 18613.8 (15162.4-22653.4) | 11316.6 (9202.3-13773.9) | 18690.3 (15198.3-22748.7) | -0.03 (-0.06-0) |
| Mauritania | 156651.9 (128418.9-191310.4) | 20384.7 (16710.8-24894.7) | 327852.1 (268128.8-400878.2) | 20403.9 (16687.1-24948.7) | 0 (0-0.01) |
| Cambodia | 831622.9 (676481.6-1023291.8) | 21449.9 (17448.4-26393.6) | 1521771.9 (1237952.9-1866541) | 21399.6 (17408.4-26247.8) | -0.03 (-0.05-0) |
| Singapore | 172938.5 (144044.2-205774) | 11458.5 (9544-13634.1) | 300742.5 (244121.7-365791.4) | 14131.6 (11471.1-17188.2) | 0.73 (0.5-0.96) |
| Nepal | 1515035.6 (1227543.8-1846406) | 20745.8 (16809.1-25283.3) | 2741402.3 (2206503.8-3335041.1) | 20966.3 (16875.4-25506.4) | 0.05 (0.04-0.06) |
| South Sudan | 279230 (226835.7-340736.8) | 11959.7 (9715.6-14594.1) | 416723.6 (338968.2-508990.2) | 12189.2 (9914.8-14888) | 0.09 (0.08-0.1) |
| Slovakia | 376131.5 (311229.1-455852.9) | 18370.7 (15200.8-22264.4) | 332821.3 (274738.7-405084.2) | 18632.9 (15381.2-22678.5) | 0.07 (0.06-0.09) |
| Mongolia | 160277.3 (130259.7-196245) | 18150.5 (14751.1-22223.6) | 254526.4 (207745.3-312220.1) | 18786 (15333.2-23044.2) | 0.1 (0.08-0.12) |
| Italy | 5965374.2 (5019681.6-7071313.5) | 27940.1 (23510.8-33120) | 4852450.9 (4120254.3-5707993.5) | 30133.2 (25586.3-35446) | 0.36 (0.24-0.48) |
| Kiribati | 5765.4 (4666.5-7038.9) | 18884.5 (15284.9-23055.8) | 9265.9 (7534.7-11289.7) | 18994.6 (15445.6-23143.2) | -0.01 (-0.03-0.01) |
| Ireland | 351384.4 (285586.2-431947.3) | 25613.9 (20817.6-31486.5) | 419391 (342018.7-514030) | 26081.3 (21269.6-31966.7) | 0.09 (0.08-0.1) |
| Andorra | 6373.9 (5184.2-7829.5) | 25487.3 (20730.1-31307.8) | 6586 (5386.1-8085.5) | 25966.6 (21235.5-31878.4) | 0.05 (0.01-0.08) |
| Kenya | 1116821.6 (927770.7-1324813.1) | 12758.1 (10598.4-15134.1) | 2794712.5 (2322860.8-3330296.9) | 12897.6 (10720-15369.4) | 0.05 (0.04-0.05) |
| Mali | 611490.8 (501618.2-746834.3) | 20543.1 (16851.9-25090) | 1662461.7 (1348668.8-2034843.3) | 20337.8 (16499-24893.3) | -0.02 (-0.03--0.02) |
| Morocco | 2242268.1 (1837412.9-2742806.7) | 21550.3 (17659.2-26360.9) | 3173346.9 (2604970.2-3867777.8) | 21706.5 (17818.7-26456.6) | 0.02 (0.02-0.03) |
| Romania | 1587678.1 (1313549.8-1915838.9) | 18269.1 (15114.8-22045.2) | 1037505.4 (858975.5-1260166.3) | 18468.9 (15290.8-22432.5) | 0.08 (0.07-0.09) |
| Zimbabwe | 672635.5 (543320.3-822647.7) | 16984.6 (13719.3-20772.5) | 1066965.1 (864203.8-1303900.8) | 17203.8 (13934.5-21024.2) | 0.06 (0.05-0.07) |
| Eswatini | 51804.2 (41811.9-63247.2) | 17188.6 (13873.2-20985.4) | 86986.2 (70476.2-106066.1) | 17227.3 (13957.6-21006) | 0.01 (-0.01-0.02) |
| United States of America | 25640064.3 (21580420.5-30127852.2) | 25155.4 (21172.5-29558.4) | 27130848.2 (22739278.3-32436263.7) | 24662.9 (20670.8-29485.7) | -0.06 (-0.17-0.05) |
| Turkmenistan | 281514.2 (228566.5-344959) | 18307.7 (14864.4-22433.7) | 369726.2 (302023.9-453224) | 18182.5 (14853-22288.8) | -0.06 (-0.08--0.04) |
| Venezuela (Bolivarian Republic of) | 1438820 (1191517.1-1715411.1) | 17953.8 (14867.9-21405.2) | 1999480.6 (1640511-2415167) | 18799.9 (15424.7-22708.3) | 0.17 (0.14-0.21) |
| Marshall Islands | 3191.8 (2582.6-3891.4) | 18560.8 (15018.1-22629.1) | 4476.6 (3646.8-5444.7) | 18823.5 (15334.5-22894.2) | 0.07 (0.06-0.09) |
| Trinidad and Tobago | 92814.2 (75556.8-114281.1) | 18501.7 (15061.6-22780.9) | 94863 (77758.3-116515.8) | 18581.8 (15231.4-22823.2) | 0.02 (0.01-0.03) |
| Taiwan (Province of China) | 1535084.7 (1300438.7-1799755) | 16641.4 (14097.7-19510.6) | 1377929.8 (1113117.6-1681327.9) | 17353.4 (14018.4-21174.3) | 0.18 (0.14-0.22) |
| Angola | 667042.2 (539767.5-816999.1) | 16953.7 (13718.8-20765) | 1919419.5 (1553056.5-2347250) | 17131.6 (13861.6-20950.1) | 0.05 (0.04-0.05) |
| Palestine | 162496.4 (132669.4-199335.4) | 21224.7 (17328.8-26036.5) | 447114 (365741.2-547490.1) | 21395.5 (17501.6-26198.7) | 0.03 (0.03-0.03) |
| Suriname | 29551 (23965.2-36627) | 18285.9 (14829.4-22664.4) | 39943.7 (32588.7-48972.6) | 18598.8 (15174.1-22802.9) | 0.06 (0.06-0.06) |
| Saint Lucia | 10440.9 (8457.8-12905.6) | 18567.8 (15041.2-22950.9) | 12531.1 (10247.8-15374.2) | 18558.5 (15176.9-22769.1) | -0.01 (-0.01--0.01) |
| Niger | 567547.6 (463100.8-693543.7) | 20422.4 (16664-24956.2) | 1641024.7 (1332092.7-2012009.1) | 20195.5 (16393.6-24761.1) | -0.03 (-0.04--0.02) |
| Bahamas | 21968.7 (17856.7-27133.9) | 18607 (15124.2-22981.9) | 27985.6 (22892.8-34314.7) | 18695.2 (15293-22923.2) | 0.01 (0.01-0.02) |
| Ethiopia | 2068468.4 (1719469.1-2452513.5) | 11088.1 (9217.3-13146.8) | 4928924.8 (4123543.1-5832138.6) | 11104.5 (9290-13139.4) | 0.04 (0.02-0.06) |
| Micronesia (Federated States of) | 7418.3 (6000.2-9041.3) | 18568.5 (15018.9-22630.8) | 7854.8 (6361.6-9593.9) | 18581 (15048.8-22695) | 0 (-0.01-0) |
| Lao People's Democratic Republic | 328547.6 (266947.5-403510.5) | 21256.1 (17270.7-26106) | 670128.7 (545327.9-825414.4) | 21285.6 (17321.5-26218.1) | -0.01 (-0.02-0.01) |
| Belarus | 693432.3 (565253.5-842541.9) | 17545.9 (14302.6-21318.8) | 541425.7 (444777.5-661016.6) | 17746.9 (14578.9-21666.8) | 0.03 (0-0.06) |
| Malta | 35597.2 (29110.1-43666.3) | 25807.9 (21104.8-31658) | 35807.2 (29238-44009.3) | 25956.4 (21194.5-31902.1) | 0.03 (0.01-0.04) |
| Samoa | 12135 (9801.1-14820.3) | 18190.1 (14691.6-22215.3) | 15402.9 (12462.2-18775.2) | 18510.6 (14976.6-22563.3) | 0.05 (0.03-0.06) |
| Brazil | 15580138.8 (13032605.5-18853289.8) | 24828.8 (20769-30045) | 21973447.1 (18263593-26350779.4) | 25514.1 (21206.4-30596.7) | 0.14 (0.08-0.2) |
| Dominica | 5310.3 (4306.6-6557.3) | 18206 (14764.9-22481.1) | 4746.9 (3867.7-5817.6) | 18412 (15001.7-22565.1) | 0.04 (0.03-0.05) |
| Latvia | 166756.1 (136162.3-202512.5) | 17478 (14271.4-21225.7) | 98913.2 (81075.2-120696.8) | 17619.3 (14441.8-21499.6) | 0.01 (-0.01-0.04) |
| Uzbekistan | 1571144.8 (1275291-1926426.4) | 18289.7 (14845.7-22425.6) | 2638608.3 (2153930.2-3232566.9) | 18496.3 (15098.8-22659.9) | 0.02 (0-0.03) |
| Philippines | 5632340.4 (4728394.5-6643148.6) | 21693.4 (18211.8-25586.7) | 10060239.3 (8442174-11861793.6) | 21809.5 (18301.7-25715.1) | 0.01 (0-0.01) |
| Luxembourg | 36934.4 (30180.9-44746.6) | 25018.6 (20443.9-30310.3) | 53354.6 (43821.5-64507.7) | 24998.4 (20531.8-30224) | -0.09 (-0.13--0.05) |
| Mauritius | 106739.1 (86827-130981) | 21365.4 (17379.7-26217.7) | 100675.9 (82097.1-124175.4) | 21480.5 (17516.5-26494.5) | 0.02 (0.01-0.02) |
| Paraguay | 373121.6 (302985.8-465707.1) | 23821.7 (19343.9-29732.8) | 699443.4 (569883.7-870692.7) | 23534.3 (19175-29296.4) | -0.04 (-0.05--0.03) |
| Benin | 352299 (288255.5-430350.5) | 20687.8 (16927-25271.1) | 996638.4 (815056.8-1218825.7) | 20392 (16676.7-24938.2) | -0.05 (-0.05--0.05) |
| Malaysia | 1384615.4 (1136436.4-1676386.4) | 18683.4 (15334.6-22620.5) | 2673321 (2181229.6-3295782.1) | 19398.3 (15827.5-23915) | 0.16 (0.12-0.19) |
| Ecuador | 637610 (527723.2-782232.2) | 15468.2 (12802.4-18976.7) | 1157093.1 (932247-1436761) | 16045 (12927.1-19923) | 0.19 (0.15-0.22) |
| Monaco | 2404.9 (1959.4-2949.9) | 26269.9 (21403.6-32222.9) | 2416.4 (1975.5-2964.7) | 26036 (21285.3-31944.5) | -0.03 (-0.04--0.02) |
| Qatar | 45167 (36735.3-55175.2) | 19110.8 (15543.2-23345.4) | 314879.2 (254231.6-384451.4) | 18329.7 (14799.3-22379.6) | -0.27 (-0.36--0.19) |
| El Salvador | 391844.8 (319339.8-483736.8) | 18783.9 (15308.2-23188.9) | 487836.2 (398258.6-596408.6) | 18933.4 (15456.8-23147.2) | 0.02 (0.01-0.03) |
| Armenia | 268104.5 (218806.8-327629.1) | 18648.1 (15219.2-22788.3) | 210928.1 (172278.9-259088.4) | 18919.3 (15452.7-23239.1) | 0.03 (-0.01-0.06) |
| Iran (Islamic Republic of) | 5051708.7 (4261128.4-5974327.5) | 22359.2 (18860-26442.7) | 8185990.6 (6934141.6-9622461.8) | 22989.2 (19473.6-27023.4) | 0.11 (0.02-0.19) |
| Cuba | 897090.1 (729419.7-1108215.2) | 18443.5 (14996.3-22784) | 668680.7 (546230.1-820722.9) | 18438 (15061.6-22630.3) | -0.02 (-0.03--0.01) |
| Nigeria | 7263886.6 (6080780-8695432.6) | 21327 (17853.3-25530) | 17751789.8 (14881120.9-21068356.5) | 21169 (17745.7-25124) | -0.01 (-0.02-0.01) |
| Myanmar | 3591949.4 (2915932.8-4420611.3) | 21208.6 (17217.1-26101.4) | 4755326.6 (3873214.6-5834978.6) | 21519 (17527.3-26404.7) | 0.05 (0.05-0.05) |
| Malawi | 443235.1 (360162.6-540823.2) | 12256.6 (9959.4-14955.2) | 932663.4 (756422.1-1139059.1) | 12236.2 (9924-14944.1) | 0.01 (0-0.02) |
| Oman | 159536.8 (130746.2-195377.8) | 19872.4 (16286.2-24336.9) | 498670.5 (405508.3-610589.8) | 19450 (15816.3-23815.3) | -0.11 (-0.2--0.02) |
| Congo | 160717.6 (129812.3-196351.7) | 16943.4 (13685.3-20700.1) | 363382.4 (294951.8-443144.5) | 17256.6 (14006.9-21044.4) | 0.09 (0.08-0.1) |
| Madagascar | 552279.8 (449128.2-673707.2) | 12206.1 (9926.3-14889.8) | 1335416.1 (1088222.1-1631924.3) | 12224.4 (9961.6-14938.7) | 0 (0-0.01) |
| Papua New Guinea | 307310.3 (248451.4-375356.9) | 18568.7 (15012.3-22680.3) | 768327.6 (623747.7-934663.3) | 18774.6 (15241.7-22839.1) | 0.02 (0.02-0.03) |
| Indonesia | 17064101.2 (14335683.5-20121599.3) | 21860.1 (18364.9-25777) | 23350278.9 (19574567-27533467.2) | 22044.5 (18479.9-25993.8) | 0.04 (0.03-0.05) |
| New Zealand | 278553.2 (231452.9-332091.3) | 20154.8 (16746.8-24028.6) | 285904.9 (237655.2-341136.4) | 20272.1 (16850.9-24188.2) | 0 (-0.03-0.02) |
| Bolivia (Plurinational State of) | 353444.8 (285463.4-436097.1) | 14346.6 (11587.2-17701.5) | 689005.9 (556133-845865.9) | 14250.8 (11502.6-17495.2) | -0.03 (-0.04--0.03) |
| Sao Tome and Principe | 8677 (7044.3-10621.6) | 20209.5 (16406.9-24738.8) | 17622.5 (14437.3-21484.1) | 20293.6 (16625.7-24740.5) | 0.02 (0.02-0.03) |
| Antigua and Barbuda | 4826.1 (3929.6-5943.2) | 18709 (15233.6-23039.7) | 6462.3 (5289.9-7924.7) | 18672.8 (15285.1-22898.5) | -0.02 (-0.05-0) |
| Belgium | 1097834.8 (900457-1349943.1) | 29512 (24206.1-36289.2) | 1139702.4 (941907.2-1373104) | 32501.7 (26861-39157.7) | 0.36 (0.27-0.46) |
| Nauru | 760.4 (617.8-924.7) | 18802.4 (15276.5-22864.4) | 856 (694.9-1041.2) | 18761.9 (15230.1-22821.2) | -0.01 (-0.01-0) |
| Burkina Faso | 658103.6 (536336.4-803666.4) | 20610.1 (16796.7-25168.8) | 1748439.1 (1432650.5-2136346.4) | 20482.8 (16783.4-25027.1) | -0.02 (-0.02--0.01) |
| Bosnia and Herzegovina | 350891.4 (289664.2-424050.7) | 18167.2 (14997.2-21955) | 192254.7 (158849.1-232949.3) | 18473.9 (15263.9-22384.3) | 0.02 (-0.01-0.04) |
| Bulgaria | 547199.5 (452819.7-661666.8) | 18385.6 (15214.5-22231.7) | 366683.3 (303311.2-446425) | 18566.3 (15357.6-22603.8) | 0.06 (0.05-0.07) |
| Democratic Republic of the Congo | 2426375.1 (1959487.1-2965618.3) | 16972.2 (13706.4-20744.2) | 5919811.4 (4784786.1-7240467.2) | 16921.1 (13676.8-20696) | -0.02 (-0.03--0.01) |
| Norway | 396007.9 (332412.5-471158) | 24754.6 (20779.3-29452.3) | 503678.5 (424434.5-586741) | 28620.4 (24117.5-33340.2) | 0.66 (0.59-0.72) |
| Algeria | 2158527.6 (1761775-2637916.4) | 21316 (17397.9-26050) | 3740674.8 (3074269-4541872.1) | 21809.4 (17924-26480.6) | 0.08 (0.07-0.09) |
| Slovenia | 141078.7 (116648-171062.6) | 18410.9 (15222.6-22323.8) | 110059.4 (90933.5-133768.2) | 18526.2 (15306.8-22517.1) | 0.03 (0.02-0.03) |
| Portugal | 977433.6 (795203.8-1196183.1) | 25819.1 (21005.4-31597.4) | 793545.2 (648239.6-973258.4) | 26076 (21301.2-31981.4) | 0.05 (0.04-0.07) |
| Chile | 933380.2 (760171.7-1134196.1) | 16298.3 (13273.8-19804.8) | 1154368.1 (939532.3-1414499.1) | 16963.3 (13806.3-20785.9) | 0.16 (0.14-0.19) |
| Solomon Islands | 23652.1 (19101.6-28871.8) | 18406.6 (14865.4-22468.7) | 49069.9 (39792-59779.6) | 18757.1 (15210.6-22851) | 0.09 (0.08-0.1) |
| Cabo Verde | 26661.1 (21598.6-32526.2) | 20415.1 (16538.5-24906.1) | 50629 (41575.1-61873.2) | 20354.6 (16714.6-24875.1) | -0.03 (-0.05--0.01) |
| Czechia | 680032 (563185.6-821893.6) | 18301 (15156.4-22118.7) | 566525.2 (468014.8-689402.1) | 18607.4 (15371.9-22643.3) | 0.11 (0.09-0.12) |
| Netherlands | 1376059.6 (1136185.8-1643667.3) | 22821.7 (18843.4-27259.9) | 1316804.1 (1083221.3-1600322.7) | 25015.6 (20578.2-30401.7) | 0.47 (0.36-0.58) |
| Senegal | 563239.3 (458634.7-687709.4) | 20422.9 (16630-24936.2) | 1243276.6 (1018733.7-1518022.3) | 20283.2 (16619.9-24765.5) | -0.02 (-0.02--0.02) |
| Northern Mariana Islands | 4491.7 (3652.4-5456.3) | 19172.3 (15590-23289.7) | 2531.3 (2034.4-3097.2) | 18181.7 (14612.9-22246.8) | -0.25 (-0.36--0.13) |
| Tunisia | 739821.5 (604941.3-905773.4) | 21469.3 (17555.1-26285.1) | 974023.7 (801534-1180525.5) | 21979 (18086.7-26638.7) | 0.09 (0.08-0.09) |
| Hungary | 681499.5 (564769.3-827097.6) | 18431.2 (15274.2-22368.9) | 528097 (436123.8-640480.5) | 18532.5 (15304.9-22476.3) | 0.07 (0.05-0.08) |
| Sierra Leone | 280854.3 (229832.7-343057.7) | 20588.9 (16848.6-25148.9) | 713222.4 (584236.5-871902.1) | 20399.3 (16710.1-24937.7) | -0.06 (-0.07--0.05) |
| Guyana | 63081.3 (51191.3-77961.6) | 18541.9 (15047-22915.8) | 59984 (48778.8-74078.1) | 18547 (15082.4-22904.9) | -0.01 (-0.01--0.01) |
| Central African Republic | 179279.2 (145095.1-219323.4) | 17075.5 (13819.6-20889.6) | 360610.7 (291773.2-440720.4) | 17089.8 (13827.5-20886.3) | 0 (-0.01-0.01) |
| Germany | 8143073.8 (6654899.3-9985759.3) | 27413.6 (22403.7-33617) | 7283776.4 (6042032.5-8865066.7) | 28582.3 (23709.6-34787.5) | 0 (-0.11-0.12) |
| Kuwait | 178877 (146700.4-217218.4) | 20511 (16821.5-24907.4) | 454135.3 (375015.1-552095.4) | 21751.4 (17961.8-26443.3) | 0.2 (0.15-0.26) |
| Mozambique | 570337.8 (465055.5-696310.6) | 12466.3 (10165.1-15219.8) | 1381361.2 (1120816.2-1685069) | 12307.1 (9985.8-15013) | -0.03 (-0.04--0.02) |
| Grenada | 6133.1 (4976.8-7566.9) | 18399.7 (14931-22701.4) | 7470.3 (6073-9212.1) | 18303.1 (14879.5-22570.8) | -0.01 (-0.01-0) |
| Saudi Arabia | 1369652.1 (1111969.8-1671885.1) | 20425.9 (16583-24933.2) | 3739961.3 (3098632.7-4508099.8) | 20413.3 (16912.8-24605.9) | -0.01 (-0.02-0) |
| Colombia | 2646266.5 (2196248.3-3161368.1) | 18804.2 (15606.4-22464.5) | 3625659.8 (2949615.5-4415120.3) | 18955.2 (15420.8-23082.6) | 0.03 (0.02-0.04) |
| Russian Federation | 11232522.8 (9419820.7-13356090.7) | 19301.6 (16186.7-22950.7) | 9400895.9 (7879748.9-11226581.2) | 19577.2 (16409.5-23379.2) | 0.12 (0.09-0.15) |
| Cameroon | 777254.7 (635306.8-950468.5) | 20462.9 (16725.8-25023.2) | 2461876.6 (2017294.7-3004013.1) | 20374.2 (16694.9-24860.9) | -0.02 (-0.02--0.01) |
| Syrian Arab Republic | 1023633.8 (836027-1254679.1) | 21254.4 (17359-26051.8) | 1207516.6 (989819-1473683.2) | 21920 (17968.2-26751.7) | 0.13 (0.09-0.16) |
| Lithuania | 229782.9 (186395.4-280077.9) | 16493.6 (13379.3-20103.7) | 133753 (111166-162361.1) | 15927 (13237.4-19333.6) | -0.18 (-0.23--0.14) |
| Albania | 257122 (211125.3-311213.7) | 18129.6 (14886.4-21943.6) | 176007 (145040.3-213318.7) | 18139.5 (14948-21984.9) | -0.04 (-0.06--0.02) |
| Chad | 429908.5 (351230.7-525395) | 20485 (16736-25034.9) | 1177766.4 (956152.2-1442266.9) | 20342.8 (16515-24911.3) | -0.02 (-0.02--0.01) |
| Austria | 737581 (601056.9-904420) | 24579.9 (20030.2-30139.8) | 692366.9 (569115.7-843849.8) | 24610 (20229.1-29994.4) | 0.07 (0.01-0.12) |
| Rwanda | 334981.2 (273150.8-409583.6) | 12261.4 (9998.2-14992.1) | 666355.5 (545426.2-814802.8) | 12307.4 (10073.9-15049.2) | 0.02 (0.01-0.04) |
| Belize | 13453.7 (10898.1-16633.7) | 18365.9 (14877.1-22707) | 33273.5 (27091.2-41108) | 18579.6 (15127.4-22954.2) | 0.04 (0.03-0.05) |
| Finland | 470909.3 (385680.6-577514.8) | 25941.5 (21246.4-31814.2) | 432109.9 (353554.8-530320) | 25828.6 (21133.1-31698.9) | -0.01 (-0.02-0) |
| Egypt | 4745790.1 (3875960.5-5764847) | 21658.5 (17688.8-26309.2) | 9257642.3 (7857671.5-10893983.8) | 22570.6 (19157.4-26560.1) | 0.2 (0.17-0.24) |
| Vanuatu | 10989.6 (8903.4-13424.7) | 18752.6 (15192.8-22907.9) | 22303.9 (18110-27165.5) | 18894.8 (15341.9-23013.4) | 0.02 (0.01-0.03) |
| Thailand | 6811635.9 (5700745.6-8114909.9) | 26297.3 (22008.5-31328.7) | 5805433.4 (4753273.2-7034838.1) | 23923.9 (19588-28990.2) | -0.39 (-0.5--0.28) |
| Togo | 280179.1 (228579.4-342285.9) | 20438.2 (16674.2-24968.7) | 654107.3 (534534.2-797818.5) | 20520.1 (16769-25028.5) | 0.02 (0.02-0.03) |
| Spain | 3862072.5 (3166726-4749916.1) | 26041.3 (21352.7-32027.9) | 3432410.9 (2822336.4-4205344) | 26448.3 (21747.4-32404.1) | 0.05 (0.04-0.05) |
| Peru | 1011943.7 (841477.6-1216516.2) | 11411.3 (9489-13718.2) | 1730425.5 (1397931.7-2120956.8) | 12693.3 (10254.3-15558) | 0.42 (0.31-0.54) |
| Niue | 149.2 (121.1-181.7) | 18542.9 (15047.1-22579.7) | 105.1 (85.7-128) | 18803.4 (15339.7-22902.8) | 0.05 (0.04-0.06) |
| Turkey | 5100168.9 (4185113.8-6154009.7) | 20628.7 (16927.5-24891.1) | 7093582.3 (5846274.2-8464935.9) | 20838.4 (17174.3-24867) | -0.03 (-0.07-0.01) |
| Tonga | 6787.7 (5479.7-8299.7) | 18415.3 (14866.6-22517.5) | 7238.1 (5857.7-8826.9) | 18774.6 (15194-22895.7) | 0.08 (0.07-0.08) |
| Gambia | 77230.3 (63033.5-94502.5) | 20432.6 (16676.6-25002.2) | 192269.9 (157365.4-235177.9) | 20411.8 (16706.2-24967) | 0.01 (0-0.01) |
| Sweden | 761657.6 (639890.8-894391) | 25949.8 (21801.2-30472) | 831195.9 (696777.8-985181.8) | 26028.3 (21819.1-30850.3) | 0 (-0.01-0.02) |
| Ukraine | 3475067.1 (2925579.9-4112321.3) | 18303.1 (15408.9-21659.5) | 2689221.1 (2255922.1-3187331.5) | 18618.9 (15619-22067.6) | 0.07 (0.03-0.1) |
| Estonia | 99262.9 (80944-120551.8) | 17476.4 (14251.1-21224.5) | 70613.5 (57907.1-86122.8) | 17551.7 (14393.4-21406.7) | 0 (-0.02-0.03) |
| Cyprus | 79014.1 (64272-96823.8) | 25690.2 (20897-31480.7) | 131219.7 (106901.9-161268.6) | 26215.8 (21357.5-32219.1) | 0.08 (0.07-0.09) |
| Saint Kitts and Nevis | 3214.8 (2617.4-3975.4) | 18522.2 (15080.3-22904.4) | 4216.9 (3449.3-5173.5) | 18610 (15222.5-22831.4) | 0.01 (0-0.02) |
| Palau | 1309.1 (1065-1593.8) | 18716.7 (15227-22786.4) | 1127.3 (917.6-1368.3) | 18468.7 (15032.4-22416.1) | -0.08 (-0.1--0.05) |
| Azerbaijan | 588886.2 (479791-719638.1) | 18515.2 (15085.1-22626.2) | 798360.1 (651340.1-978915.4) | 18706.3 (15261.5-22936.8) | -0.03 (-0.06-0) |
| United Arab Emirates | 186275.4 (151788.2-228466.7) | 19444.9 (15844.8-23849.1) | 862520.5 (706456.2-1050775.3) | 19238.1 (15757.2-23437.1) | -0.11 (-0.15--0.06) |
| Equatorial Guinea | 25912.9 (20979.2-31666.2) | 17273.1 (13984.4-21108.1) | 107240.8 (86385.3-131332.3) | 16477.7 (13273.3-20179.4) | -0.18 (-0.19--0.18) |
| Maldives | 17009.3 (13761-20919.2) | 20925.9 (16929.6-25736.1) | 51730.9 (41921-64084.6) | 20520.9 (16629.5-25421.5) | -0.09 (-0.11--0.07) |
| Canada | 2739029.9 (2305262.3-3254327.9) | 24635.5 (20734.1-29270.2) | 2767544.4 (2255217.2-3352888.8) | 24108.9 (19645.9-29208) | -0.09 (-0.11--0.08) |
| Montenegro | 45545.1 (37590.5-55047.5) | 18167.2 (14994.3-21957.6) | 38745.6 (32073.9-46967.5) | 18375.7 (15211.6-22275.1) | 0.05 (0.04-0.05) |
| C么te d'Ivoire | 957547.9 (783483-1172503.3) | 20243.4 (16563.5-24787.7) | 2188044.9 (1789290.5-2670619.6) | 20321.6 (16618.1-24803.5) | 0.02 (0.01-0.03) |
| United Republic of Tanzania | 1152267.6 (942006.1-1396877.2) | 11903.7 (9731.6-14430.7) | 2798125.5 (2264564.7-3431278.9) | 12672 (10255.7-15539.4) | 0.35 (0.27-0.42) |
| Somalia | 306893.9 (251063.1-374270) | 12155.1 (9943.8-14823.7) | 942325.1 (763777.9-1152165.1) | 12015.3 (9738.7-14690.9) | 0.03 (0-0.06) |
| Croatia | 334343.9 (276200.4-405728.4) | 18430.4 (15225.3-22365.4) | 237346.7 (195522.7-291106.7) | 18416 (15170.8-22587.3) | -0.11 (-0.21--0.01) |
| Bahrain | 52329.1 (42820.3-64003.9) | 20377.8 (16674.9-24924.2) | 126602.9 (104381.2-153153) | 20474.4 (16880.6-24768.1) | -0.09 (-0.14--0.04) |
| Puerto Rico | 264604.3 (215215.4-326123.4) | 18711.6 (15219.1-23062) | 210105.2 (172038.6-257661.8) | 18701.4 (15313.1-22934.4) | -0.01 (-0.01-0) |
| Jordan | 322882 (262478.7-396899.2) | 20901.5 (16991.3-25692.9) | 1053967.7 (864964.8-1287904.7) | 21032.1 (17260.5-25700.4) | 0.02 (0-0.05) |
| Ghana | 1174606.6 (963468.3-1433861.4) | 20430.6 (16758.1-24939.9) | 2796355.1 (2285348.6-3419656.1) | 20541.2 (16787.5-25119.8) | 0.02 (0.01-0.02) |
| Greece | 1024807.4 (836535.1-1247617.7) | 27267.1 (22257.7-33195.4) | 794892.6 (653657.7-972694.2) | 27332.7 (22476.3-33446.5) | 0.02 (0.01-0.02) |
| Yemen | 995805.9 (816196.3-1218265.5) | 21593.9 (17699.1-26418) | 2804560 (2304300.4-3432628.6) | 21558.2 (17712.8-26386.1) | 0.02 (0-0.04) |
| Guatemala | 543028 (443312.1-669483.9) | 18714.6 (15278.1-23072.7) | 1482141.4 (1210444.4-1817885.1) | 18715.3 (15284.5-22954.8) | 0 (-0.01-0.01) |
| Seychelles | 6596.3 (5362.7-8114.2) | 21117.9 (17168.5-25977.4) | 8152.5 (6677.1-10064.8) | 21123.6 (17300.8-26078.7) | -0.04 (-0.06--0.02) |
| Guinea-Bissau | 77008.8 (62921.6-94125.8) | 20513.6 (16761-25073.2) | 164845.7 (134862.9-201354.3) | 20513.6 (16782.5-25056.7) | 0.01 (0-0.02) |
| Costa Rica | 239263.5 (195706.7-292893.2) | 18633.1 (15241-22809.6) | 361046 (296220.1-440840) | 18846.5 (15462.6-23011.7) | 0.04 (0.03-0.05) |
| Zambia | 406666.3 (326669-502452.7) | 13405.7 (10768.6-16563.3) | 1004790.8 (818622.3-1239541.5) | 13242.1 (10788.6-16335.9) | -0.06 (-0.08--0.03) |
| United Kingdom | 5293424.4 (4434388.6-6273667.6) | 25381.7 (21262.7-30082) | 5610478.4 (4696955.3-6664162.1) | 25653.4 (21476.4-30471.2) | 0.01 (-0.02-0.03) |
| Denmark | 417734.8 (342315.8-509116.7) | 21895.2 (17942.2-26684.9) | 411343.4 (336292.2-504117.2) | 22820.6 (18656.9-27967.5) | 0.14 (0.09-0.2) |
| Switzerland | 601466.6 (494794.5-730451.3) | 22826 (18777.7-27721) | 652221.2 (532376.1-799281.6) | 23505.8 (19186.6-28805.8) | 0.11 (0.1-0.13) |
| Republic of Moldova | 307802.2 (251246-373773.6) | 17650.6 (14407.4-21433.6) | 231985.7 (190395.6-282974) | 17679 (14509.5-21564.7) | 0 (-0.03-0.04) |
| France | 5628300.9 (4616875.9-6885863.7) | 25590.2 (20991.5-31307.9) | 4981440.2 (4113707.8-6031877.6) | 25054.7 (20690.3-30338) | -0.06 (-0.09--0.04) |
| Gabon | 64589.8 (52196-79071) | 16922.9 (13675.7-20717.1) | 128686 (104292.1-156892.4) | 17294.5 (14016.1-21085.3) | 0.06 (0.06-0.07) |
| Djibouti | 23788.5 (19343.3-29075.2) | 11854.1 (9639-14488.6) | 62607.6 (51011.7-76633.5) | 12403.5 (10106.2-15182.3) | 0.17 (0.16-0.19) |
| Brunei Darussalam | 18405.8 (14980.2-22738.4) | 14928 (12149.6-18441.9) | 30262.2 (24595.8-37247) | 15003.2 (12193.9-18466.1) | -0.01 (-0.04-0.01) |
| American Samoa | 3797.4 (3072.1-4641) | 18757.1 (15174.5-22924.3) | 3912.6 (3167.8-4774.5) | 18561.3 (15027.6-22649.7) | -0.05 (-0.07--0.04) |
| Sri Lanka | 1575845.4 (1281718.6-1943007.9) | 21302.1 (17326.1-26265.3) | 1734412.3 (1414495.1-2138741.4) | 21580.9 (17600.3-26611.9) | 0.06 (0.05-0.07) |
| Burundi | 255834.7 (209046.7-313307.6) | 12350.1 (10091.5-15124.6) | 567825.6 (462830.5-694796.2) | 12287.9 (10015.8-15035.6) | -0.04 (-0.06--0.01) |
| Iraq | 1398977.6 (1142174.2-1709062) | 21277.6 (17371.8-25993.8) | 3991356.8 (3272352-4893362.5) | 21375.7 (17525-26206.4) | 0.02 (0.02-0.03) |
| Dominican Republic | 573905.5 (465040-709346.9) | 18671.3 (15129.5-23077.7) | 830919.2 (676557.3-1021365.2) | 18475.5 (15043.3-22710.1) | -0.06 (-0.07--0.05) |
| Guinea | 445598.2 (364714.6-543727.6) | 20636.4 (16890.5-25180.9) | 994303.3 (814079.7-1216598.4) | 20615.2 (16878.5-25224.1) | 0.01 (0-0.02) |
| Afghanistan | 833178.1 (673412.9-1022370.2) | 21517.6 (17391.5-26403.6) | 3191373.6 (2602973.5-3920139.4) | 21177.5 (17272.9-26013.5) | -0.04 (-0.06--0.01) |
| North Macedonia | 148028.5 (122160.8-178892.1) | 18220.6 (15036.6-22019.5) | 142361.4 (117553.8-172660.7) | 18425.9 (15215-22347.5) | 0.03 (0.02-0.04) |
| Honduras | 322324.1 (262778.8-397712.9) | 18679.1 (15228.4-23048) | 796067.8 (650047.5-978401.4) | 18838.6 (15383.1-23153.4) | 0.04 (0.04-0.05) |
| Bangladesh | 8392727.5 (6760683.4-10295770.5) | 19663.4 (15839.6-24122) | 13574815.7 (11021513.9-16537412.4) | 20020.4 (16254.7-24389.7) | 0.06 (0.06-0.07) |
| Tokelau | 107.6 (87.3-130.8) | 18664.3 (15141.2-22702) | 90.4 (73.5-110.1) | 18653 (15166.9-22714.1) | -0.02 (-0.04-0) |
| Lesotho | 117411.5 (94886.5-143459.4) | 17018.9 (13753.9-20794.6) | 162244.3 (131587.7-198128.2) | 17078.5 (13851.4-20855.7) | 0.02 (0.01-0.03) |
| Uganda | 782571.3 (634498.1-955020.3) | 12194.2 (9886.9-14881.3) | 1970788.2 (1595985.4-2404384.6) | 12197.7 (9878-14881.4) | 0 (0-0) |
| Argentina | 1874447.7 (1553269.4-2249458.1) | 15336.4 (12708.6-18404.7) | 2836996.2 (2316276.8-3474768) | 16287.4 (13297.9-19948.9) | 0.29 (0.23-0.34) |
| Tuvalu | 691.9 (563.5-843.5) | 19202.5 (15638.5-23409.2) | 887.3 (719.8-1081.1) | 18527.7 (15030-22573.1) | -0.15 (-0.17--0.14) |
| Barbados | 20358.7 (16647.6-24965.7) | 18644.4 (15245.8-22863.4) | 18470.2 (15137.7-22667.4) | 18672.9 (15303.9-22916.2) | 0.01 (0-0.01) |
| San Marino | 2339.4 (1904.8-2865.4) | 25815.1 (21019.3-31618.9) | 2707.8 (2205.8-3321.4) | 26314 (21435.4-32276) | 0.05 (0.02-0.09) |
| Comoros | 21001.8 (17006-25622.9) | 12172.4 (9856.5-14850.8) | 36413.8 (29775.4-44520.6) | 12290.9 (10050.2-15027.2) | 0.03 (0.03-0.04) |
| Botswana | 88189.7 (71250-107716.3) | 17138.8 (13846.7-20933.6) | 182984.6 (148590.2-222609.4) | 17359.4 (14096.5-21118.6) | 0.05 (0.03-0.06) |
| Sudan | 1648576.1 (1345861.7-2013791.9) | 21617.1 (17647.7-26406) | 3734679.9 (3064584.2-4571830.6) | 21640.8 (17757.9-26491.7) | 0.02 (0.01-0.02) |

Abbreviations: EAPC, estimated annual percentage change; SDI, Sociodemographic Index; UI, uncertainty interval.
