# Supplementary material for: Global, regional, and national epidemiology of migraine and tension-type headache in youths and young adults aged 15–39 years from 1990 to 2019: findings from the global burden of disease study 2019
Source: J Headache Pain. 2023 Sep 18;24(1):126. doi: 10.1186/s10194-023-01659-1 (PMC10506184; doi:10.1186/s10194-023-01659-1)
Supplement: Supplementary file 12 — Additional file 12: Table S5. Prevalence of TTH Between 1990 and 2019 in 15 to 39 years at the 204 Countries Level. [file 10194_2023_1659_MOESM12_ESM.docx]

| **TableS5 Prevalence of TTH Between 1990 and 2019 in 15 to 39 years at the 204 Countries Level** | | | | | |
| --- | --- | --- | --- | --- | --- |
|  | 1990 | | 2019 | |  |
| Location | Number_95%UI | ASR | Number_95%UI | ASR | EAPC_95%CI |
| Mexico | 11584726.9 (9589682.3-13846258.1) | 32501.4 (26904.2-38846.2) | 16424371.3 (13671796.4-19579835.4) | 32730.8 (27245.4-39019.1) | 0.02 (0.02-0.02) |
| Haiti | 747593.4 (603665.9-929963.5) | 30763.4 (24840.8-38267.9) | 1639637.1 (1330219.1-2032902.9) | 30849.1 (25027.5-38248.2) | 0.02 (0.01-0.02) |
| Viet Nam | 9115820.7 (7408056.2-11331892.2) | 31959.4 (25972.1-39728.7) | 12787284.6 (10346913.7-15938698.8) | 32415.3 (26229-40404) | 0.04 (0.03-0.04) |
| Bhutan | 85305.5 (68497.4-105441.3) | 32807 (26342.9-40550.8) | 116233.6 (94194.1-143808.8) | 33097.9 (26822.1-40950) | 0.03 (0.03-0.03) |
| Jamaica | 301773.9 (242663.1-376555.2) | 30711.7 (24695.9-38322.2) | 368701.2 (297458.2-456972.4) | 30892.8 (24923.5-38288.9) | 0.01 (0-0.01) |
| Nicaragua | 451056.9 (360453.9-562521) | 30631.4 (24478.5-38200.9) | 865064 (700824.6-1071897.7) | 30837.5 (24982.8-38210.7) | 0.02 (0.02-0.03) |
| Kyrgyzstan | 741383.1 (616237.3-898425.6) | 41092.8 (34156.3-49797.2) | 1099160.9 (913826.6-1332459.3) | 41275.1 (34315.5-50035.8) | 0 (-0.01-0.01) |
| Georgia | 880059.2 (730190.4-1064556.4) | 41330 (34291.8-49994.5) | 491523.7 (406205.5-596920.1) | 41539.5 (34329.2-50446.8) | 0.01 (0-0.02) |
| Lebanon | 349896.7 (281852.7-434571.4) | 29057.7 (23406.9-36089.6) | 601775.5 (484286.8-742862) | 29313.2 (23590.2-36185.7) | 0.03 (0.02-0.03) |
| Kazakhstan | 2803753.2 (2326685.6-3390673.6) | 41261.9 (34241-49899.4) | 2884060.4 (2378466.9-3502011.4) | 41505.9 (34229.7-50399.1) | 0.01 (0-0.03) |
| Namibia | 170863 (137456.9-212222) | 30556.8 (24582.5-37953.4) | 316823.7 (256136.4-391859.9) | 31030.8 (25086.9-38380.1) | 0.05 (0.04-0.05) |
| Republic of Korea | 8002515.6 (6564601-9676200.1) | 37987.8 (31162.1-45932.8) | 7265593.7 (6115743.4-8499365.2) | 41918.9 (35284.8-49037.1) | 0.41 (0.38-0.45) |
| Timor-Leste | 101625.9 (82746.3-126633.9) | 31943.6 (26009.3-39804.2) | 170362 (135821-212869.3) | 31692.1 (25266.5-39599.7) | -0.04 (-0.04--0.03) |
| China | 117315825.8 (97541577-140327136.7) | 21349.6 (17751-25537.3) | 117313271.9 (97138430.4-141537761) | 23570.5 (19517-28437.6) | 0.48 (0.38-0.58) |
| Eritrea | 301369.2 (237557.8-373638.3) | 26031.4 (20519.5-32273.8) | 751267.1 (596078-930770.5) | 26131.7 (20733.6-32375.4) | 0.01 (0.01-0.01) |
| Iceland | 45231 (37094.7-54387.6) | 43542.3 (35709.8-52357) | 52472.1 (43082.2-62939.5) | 43754.2 (35924.4-52482.5) | 0 (0-0.01) |
| Panama | 310610.6 (250622.1-388091) | 30736.2 (24800.1-38403.3) | 493097.5 (399760.9-611160.1) | 30849.5 (25010.1-38235.8) | 0.01 (0.01-0.01) |
| Serbia | 1366807.9 (1125297.2-1645989.6) | 39746.2 (32723.2-47864.7) | 1123608.2 (926089.5-1353944.3) | 39791.8 (32796.8-47949) | 0.01 (0.01-0.02) |
| India | 114782328.8 (97103482.5-136168246.9) | 33682.5 (28494.7-39958.1) | 201394570.3 (170595577.2-239664453) | 33781.5 (28615.3-40200.8) | -0.09 (-0.14--0.04) |
| Libya | 476062.7 (380318-588299.7) | 29054.8 (23211.4-35904.8) | 887647.2 (715463.5-1093136.7) | 29332.4 (23642.6-36122.9) | 0.04 (0.04-0.05) |
| South Africa | 5175143.8 (4316271.3-6182387.4) | 32546.7 (27145.2-38881.3) | 7961803.2 (6667214-9484929.8) | 33243.5 (27838.1-39603.1) | 0.06 (0.04-0.07) |
| Democratic People's Republic of Korea | 1849719.2 (1476828.4-2303209) | 22407.9 (17890.6-27901.6) | 2258500.3 (1809024.2-2807181.8) | 22479.7 (18005.9-27941) | -0.02 (-0.04-0) |
| Uruguay | 396941 (324390.1-484339.2) | 34948.3 (28560.6-42643.2) | 423585 (345900.1-517539.4) | 35073 (28640.7-42852.4) | 0.01 (0.01-0.02) |
| Japan | 17886055.6 (15171158.4-21005030.1) | 39880.8 (33827.4-46835.2) | 13094961.2 (11091689.2-15439911.8) | 39826.6 (33733.9-46958.4) | 0 (-0.01-0.01) |
| Poland | 6082555.9 (5160523.4-7129701.7) | 42066.3 (35689.6-49308.3) | 5345095.6 (4521724.7-6313108.2) | 42291.1 (35776.5-49950.2) | 0.03 (0.02-0.04) |
| Saint Vincent and the Grenadines | 14061.8 (11293.1-17537.4) | 30632.8 (24601.2-38204.1) | 12955.5 (10490.9-16080.8) | 30862.3 (24991-38307.2) | 0.02 (0.02-0.03) |
| Australia | 2389975.7 (1958741.6-2919454.9) | 35289.4 (28922-43107.5) | 2949259.9 (2418838.7-3609637.9) | 35502.9 (29117.8-43452.5) | 0 (0-0.01) |
| Cook Islands | 2229.9 (1787.4-2786.1) | 28856.1 (23130.8-36054.4) | 1716.8 (1387.7-2141.9) | 29118 (23536.7-36328.4) | 0.04 (0.03-0.05) |
| Liberia | 215896.2 (176244.7-264225.5) | 32340 (26400.5-39579.5) | 641200 (519646.2-790181.6) | 32301.2 (26177.8-39806.4) | 0.04 (0.02-0.06) |
| Greenland | 11686.2 (9475.9-14096.5) | 44180.4 (35824.3-53292.5) | 9003.8 (7398.5-10840.2) | 44217.7 (36334.4-53236.4) | -0.06 (-0.08--0.04) |
| Tajikistan | 866162.5 (714630-1058279.1) | 40938.2 (33776.2-50018.4) | 1669719.7 (1387065.1-2015994.4) | 41187.3 (34215-49728.9) | 0.01 (0-0.02) |
| Fiji | 93341.5 (74777.4-116678.3) | 28928.3 (23175-36160.9) | 104399.9 (83945.7-130291.7) | 29131.1 (23423.7-36355.8) | 0.02 (0.02-0.03) |
| Bermuda | 8008.1 (6440.1-9970.8) | 31110.7 (25019.3-38735.5) | 5646.2 (4521.1-7030.1) | 31117.3 (24916.5-38744) | 0 (-0.01-0) |
| Israel | 827582.4 (682292.7-1001283.8) | 43292.4 (35692-52379) | 1410156.5 (1161854.6-1701147.9) | 43458.5 (35806.3-52426.3) | 0.03 (0.03-0.04) |
| United States Virgin Islands | 12212.5 (9940.6-15110.5) | 30825.2 (25090.9-38139.9) | 9379.4 (7578.7-11641.1) | 30937.2 (24997.6-38397.1) | 0.01 (0.01-0.01) |
| Pakistan | 14348958.9 (11978431.1-16929888.3) | 35042.7 (29253.5-41345.8) | 31860520.7 (26805465.1-37660429.2) | 34833 (29306.3-41174) | -0.03 (-0.03--0.02) |
| Guam | 18363.3 (14729.5-23032.7) | 28967.2 (23235.1-36332.9) | 17530 (14111.7-21966.2) | 28952.4 (23306.6-36279.2) | -0.02 (-0.04--0.01) |
| Mauritania | 247446.8 (200992.1-305160.3) | 32199.5 (26154.5-39709.6) | 516269.6 (419413.8-636129.8) | 32130.2 (26102.3-39589.7) | -0.01 (-0.01--0.01) |
| Cambodia | 1238022.9 (1002289.5-1541194.5) | 31932.1 (25851.9-39751.8) | 2286315.9 (1857465.8-2837025.2) | 32150.8 (26120.2-39895) | 0.01 (-0.01-0.02) |
| Singapore | 498885.4 (457913.6-547843) | 33055 (30340.3-36298.8) | 816560.4 (669060.5-1004175.2) | 38369.4 (31438.6-47185.3) | 0.53 (0.37-0.69) |
| Nepal | 2450797.5 (2005786.6-3048918.1) | 33559.4 (27465.7-41749.6) | 4345861.2 (3528558.1-5309441.3) | 33237.2 (26986.5-40606.7) | -0.05 (-0.06--0.04) |
| South Sudan | 606691.5 (475039.2-752342.9) | 25985.3 (20346.5-32223.7) | 883741.7 (698141.3-1097165.3) | 25849.4 (20420.6-32092.1) | -0.01 (-0.01-0) |
| Slovakia | 813776.3 (671114.5-979386.2) | 39745.8 (32778-47834.3) | 714039.1 (589506-867306.6) | 39975.3 (33003.3-48555.9) | 0.03 (0.02-0.04) |
| Mongolia | 361159.9 (297692.1-441011.7) | 40899.2 (33711.9-49942) | 562947.6 (464410.8-683704.6) | 41549.8 (34277.1-50462.6) | 0.05 (0.04-0.06) |
| Italy | 9559769 (8088315-11266639.8) | 44775.3 (37883.4-52769.8) | 7797261.9 (6622646.5-9116835.2) | 48420.1 (41125.9-56614.5) | 0.22 (0.19-0.25) |
| Kiribati | 8829.7 (7048.1-11067.8) | 28921.4 (23085.8-36252.2) | 14170.1 (11405.8-17684.8) | 29047.8 (23381.1-36252.6) | -0.01 (-0.02-0) |
| Ireland | 593278.6 (489383.9-717603.4) | 43246.6 (35673.3-52309.2) | 706637.9 (578832.6-850739.1) | 43944.7 (35996.7-52906.2) | 0.08 (0.07-0.09) |
| Andorra | 10974.8 (8948.4-13260.2) | 43884.8 (35782.1-53023.4) | 11215.1 (9163-13593.2) | 44217.4 (36126.9-53593.4) | 0.03 (0.01-0.05) |
| Kenya | 2415962.2 (2000257.7-2879266.5) | 27598.9 (22850-32891.4) | 6026075.7 (5028336.9-7186103.5) | 27810.4 (23205.8-33163.9) | 0.03 (0.03-0.04) |
| Mali | 958989.9 (777812.4-1179815.4) | 32217.4 (26130.7-39636) | 2602866.6 (2089780.8-3218470.7) | 31842.2 (25565.4-39373.3) | -0.04 (-0.04--0.03) |
| Morocco | 3028678.2 (2436772.6-3767294.7) | 29108.4 (23419.6-36207.2) | 4275019.2 (3443347.2-5267368.4) | 29242.2 (23553.4-36030.2) | 0.02 (0.01-0.02) |
| Romania | 3444635.3 (2837527.9-4157173.7) | 39636.8 (32650.9-47835.8) | 2237980.8 (1840694.7-2709397.7) | 39838.8 (32766.6-48230.6) | 0.03 (0.03-0.04) |
| Zimbabwe | 1206128.6 (968505.7-1492056.9) | 30455.7 (24455.5-37675.6) | 1911818.9 (1543170.8-2365444.3) | 30826.3 (24882.2-38140.6) | 0.06 (0.05-0.07) |
| Eswatini | 91793.8 (73791.6-113686.5) | 30457.1 (24484-37721.1) | 156471 (126486.3-193605.7) | 30988.6 (25050.2-38343) | 0.06 (0.05-0.07) |
| United States of America | 48690585.9 (41325698-56818246.8) | 47770.3 (40544.6-55744.3) | 50683731 (43254625.5-59527601.4) | 46073.2 (39319.9-54112.6) | -0.19 (-0.22--0.15) |
| Turkmenistan | 630684.3 (522479-767993.7) | 41015.3 (33978.4-49945) | 839878.8 (698056.6-1017358.3) | 41303.7 (34329.2-50031.9) | 0.02 (0.01-0.02) |
| Venezuela (Bolivarian Republic of) | 2465383.6 (1994813-3068869.4) | 30763.4 (24891.6-38293.8) | 3290909.6 (2659376.9-4083226.5) | 30942.4 (25004.5-38392) | 0.02 (0.02-0.02) |
| Marshall Islands | 4947 (3995.2-6153) | 28767.9 (23233.1-35781.1) | 6910.2 (5580.7-8622.9) | 29056.7 (23466.3-36258.1) | 0.05 (0.04-0.06) |
| Trinidad and Tobago | 154735 (125508.6-191662.7) | 30845.1 (25019-38206.3) | 158548.8 (127563.4-197228.3) | 31056.7 (24987.2-38633.3) | 0.03 (0.02-0.04) |
| Taiwan (Province of China) | 2073617.9 (1662633-2574660.7) | 22479.5 (18024.1-27911.1) | 1808373.2 (1437873.5-2239458.2) | 22774.3 (18108.3-28203.3) | 0.04 (0.04-0.05) |
| Angola | 1211463.9 (976710.8-1506088.4) | 30790.8 (24824.3-38279) | 3442518.6 (2773835.7-4280867.6) | 30725.8 (24757.6-38208.4) | 0 (0-0) |
| Palestine | 221871.7 (176644.5-274848.5) | 28980.1 (23072.7-35899.8) | 608173.4 (487346.4-753695.1) | 29102.6 (23320.7-36066.1) | 0.01 (0.01-0.01) |
| Suriname | 49600.7 (39673.3-61941.9) | 30692.4 (24549.4-38329) | 66300.1 (53629.8-82170.5) | 30871.1 (24971.4-38260.8) | 0.02 (0.01-0.02) |
| Saint Lucia | 17247.3 (13839.1-21548.5) | 30672.2 (24611-38321.3) | 20899.2 (16868.4-25995.1) | 30951.6 (24982.1-38498.6) | 0.02 (0.02-0.03) |
| Niger | 891867.7 (721270.2-1102509.8) | 32092.6 (25953.9-39672.2) | 2576867.7 (2063427.9-3193135.1) | 31712.6 (25393.9-39296.7) | -0.05 (-0.06--0.03) |
| Bahamas | 36384.2 (29364.1-45197.8) | 30816.6 (24870.8-38281.6) | 46319.1 (37481.1-57486.8) | 30942.5 (25038.5-38402.9) | 0 (0-0.01) |
| Ethiopia | 4401229.9 (3524412.5-5480658.5) | 23593 (18892.8-29379.3) | 9317940.9 (7493193.5-11430872.4) | 20992.6 (16881.6-25752.9) | -0.56 (-0.67--0.46) |
| Micronesia (Federated States of) | 11498 (9287.5-14300.4) | 28780.2 (23247.1-35794.7) | 12180.1 (9781.3-15182.9) | 28812.8 (23138.4-35916.1) | 0 (0-0.01) |
| Lao People's Democratic Republic | 493135.4 (399820.4-611407) | 31904.4 (25867.2-39556.2) | 1009462.3 (820716.4-1251208.9) | 32064.1 (26068.8-39742.8) | 0.01 (0-0.02) |
| Belarus | 1711200.5 (1407559.3-2082930.6) | 43298.4 (35615.4-52704.3) | 1326085.9 (1087701.7-1625620.6) | 43466.5 (35652.7-53284.7) | 0.03 (0.01-0.04) |
| Malta | 60404.5 (49555.2-72596) | 43793.1 (35927.4-52632) | 60916.6 (49666.5-73787.4) | 44158.1 (36003-53488.1) | 0.03 (0.01-0.05) |
| Samoa | 19037.7 (15190.6-23840.1) | 28537.2 (22770.4-35735.8) | 23919.8 (19316.6-29778.5) | 28745.9 (23213.9-35786.7) | 0.02 (0.01-0.03) |
| Brazil | 23582522.5 (19891027.5-27820269.7) | 37581.6 (31698.8-44335) | 31617008.5 (26632582.4-37181492.1) | 36711.5 (30923.9-43172.6) | -0.18 (-0.22--0.14) |
| Dominica | 8939.6 (7179.1-11140.5) | 30648.7 (24613-38194.2) | 7950.1 (6435.5-9845.5) | 30836.2 (24961.8-38188.3) | 0.01 (0.01-0.02) |
| Latvia | 412681.1 (340571.6-504228.4) | 43253.8 (35695.9-52849) | 243858.8 (200321.3-298689.8) | 43438.3 (35683-53205.3) | 0.02 (0.01-0.03) |
| Uzbekistan | 3525039.2 (2922480.3-4284592) | 41035.1 (34020.7-49877) | 5890070.9 (4902275.4-7138116.8) | 41288.6 (34364.3-50037.2) | 0.01 (0-0.02) |
| Philippines | 8705510.7 (7258606-10385935) | 33530 (27957.2-40002.3) | 15543100.4 (12991721.4-18570684.2) | 33695.8 (28164.7-40259.3) | 0.01 (0.01-0.01) |
| Luxembourg | 63993.4 (52553.4-77505.5) | 43347.7 (35598.5-52500.6) | 92748.8 (76227.4-112050.2) | 43455.9 (35715.1-52499.3) | -0.01 (-0.02-0) |
| Mauritius | 160670.2 (130242.2-199704.5) | 32160.5 (26069.8-39973.7) | 151433.9 (122894.5-188072.6) | 32310.4 (26221.2-40127.8) | 0.01 (0.01-0.02) |
| Paraguay | 537304.8 (435040.4-660884.1) | 34303.9 (27774.9-42193.7) | 1023414.5 (829244.6-1254514.9) | 34435.1 (27901.8-42211) | 0.02 (0.01-0.03) |
| Benin | 550254.8 (445755.9-679362.9) | 32312.2 (26175.8-39893.7) | 1570475.4 (1270071.6-1941293.9) | 32133.2 (25986.7-39720.5) | -0.02 (-0.02--0.02) |
| Malaysia | 2364906.6 (1911184.5-2899898.7) | 31911.1 (25788.8-39130.1) | 4432759.2 (3598259.3-5511294.8) | 32165.2 (26109.8-39991.3) | 0.02 (0.01-0.03) |
| Ecuador | 875740.1 (711525.3-1077622.7) | 21245.2 (17261.4-26142.8) | 1695919.5 (1361205.6-2101323.7) | 23516.6 (18875.3-29138.2) | 0.5 (0.4-0.59) |
| Monaco | 4039.3 (3299.2-4871.6) | 44122.9 (36038.6-53214.4) | 4067.1 (3333.9-4879.6) | 43822.7 (35922.1-52576.8) | -0.05 (-0.06--0.03) |
| Qatar | 70030.6 (56366.8-87188) | 29630.9 (23849.5-36890.4) | 508402.7 (401197.1-639309) | 29595.1 (23354.4-37215.4) | 0 (-0.01-0) |
| El Salvador | 639485.2 (511368.7-795882.4) | 30655 (24513.5-38152.2) | 794645.6 (639905.4-986544.7) | 30840.9 (24835.3-38288.7) | 0.01 (0.01-0.02) |
| Armenia | 594153.6 (491936.8-717932.3) | 41326.5 (34216.8-49936) | 464263.1 (381882.9-567886.8) | 41642.3 (34253.2-50936.9) | 0.02 (0-0.03) |
| Iran (Islamic Republic of) | 7557857.4 (6314861.4-9015618.9) | 33451.5 (27949.9-39903.7) | 13283026.1 (11136754.9-15809000) | 37303.6 (31276.1-44397.4) | 0.28 (0.25-0.32) |
| Cuba | 1497721.4 (1202362.4-1861126.8) | 30792 (24719.6-38263.3) | 1122508.3 (909874.4-1392013.3) | 30951.7 (25088.6-38382.9) | 0 (-0.01-0.01) |
| Nigeria | 11508365.3 (9668438.6-13620186.4) | 33788.9 (28386.8-39989.2) | 28233248.5 (23750056.5-33448532.9) | 33668.1 (28321.9-39887.3) | -0.09 (-0.12--0.06) |
| Myanmar | 5411259.2 (4400892.6-6727334.8) | 31950.7 (25985-39721.5) | 7111531.8 (5779919.1-8839558.5) | 32181.5 (26155.6-40001.2) | 0.02 (0.02-0.02) |
| Malawi | 941487.7 (739520-1164765.8) | 26034.6 (20449.7-32208.8) | 1979270 (1560795.9-2456899.1) | 25967.4 (20477.1-32233.7) | 0 (0-0.01) |
| Oman | 235496.2 (189990.6-288785.6) | 29334.2 (23665.8-35972.1) | 759003.6 (607494.4-947915.1) | 29604 (23694.6-36972.3) | 0.03 (0.01-0.04) |
| Congo | 289952.5 (233098-359973.7) | 30567.9 (24574-37949.7) | 655063.7 (530153.4-806378.2) | 31108.1 (25176.3-38293.9) | 0.08 (0.07-0.09) |
| Madagascar | 1178575.6 (930813.2-1461809.9) | 26048 (20572.2-32307.9) | 2851320.7 (2261256.5-3531790.7) | 26101.1 (20699.6-32330.1) | 0.01 (0-0.01) |
| Papua New Guinea | 476245.9 (382631.4-596509.6) | 28776.4 (23119.9-36043.1) | 1184566 (951800.7-1479872.8) | 28945.6 (23257.9-36161.6) | 0.01 (0.01-0.02) |
| Indonesia | 26239623.2 (21943485.1-31323876) | 33614.5 (28110.9-40127.8) | 35913895.8 (30120259.2-42980847.2) | 33905.5 (28435.9-40577.3) | 0.04 (0.03-0.04) |
| New Zealand | 513835.6 (434689.8-605713.5) | 37178.7 (31452.1-43826.6) | 525675.1 (444926.2-621891.4) | 37272.9 (31547.4-44095.1) | 0 (-0.03-0.03) |
| Bolivia (Plurinational State of) | 624890 (493983.2-785191.2) | 25364.8 (20051.2-31871.5) | 1226609.9 (973710.3-1543665.9) | 25370.2 (20139.4-31927.9) | 0 (0-0) |
| Sao Tome and Principe | 13624.5 (10889.4-16872.7) | 31732.8 (25362.5-39298.2) | 28124.2 (22867.2-34670.4) | 32387.1 (26333.3-39925.5) | 0.1 (0.08-0.11) |
| Antigua and Barbuda | 7960.5 (6441.5-9855.7) | 30860.1 (24971.6-38207.1) | 10709.8 (8672.9-13305.4) | 30945.7 (25060.2-38445.7) | 0 (0-0.01) |
| Belgium | 1628679.1 (1337914.6-1954550.2) | 43782.2 (35965.9-52542.3) | 1539375.1 (1261837.8-1850447.4) | 43899.4 (35984.7-52770.5) | -0.01 (-0.01-0) |
| Nauru | 1170.7 (943-1460.6) | 28946.8 (23315.3-36114.8) | 1319.9 (1061.5-1648.4) | 28928.1 (23264.6-36127.9) | 0 (0-0) |
| Burkina Faso | 1021417.2 (827339.6-1262078) | 31988.2 (25910.2-39525.1) | 2745425.1 (2229523.9-3376406.6) | 32162.4 (26118.6-39554.3) | 0.03 (0.02-0.03) |
| Bosnia and Herzegovina | 765841.1 (632414.3-925707.9) | 39651 (32742.9-47928) | 414238.7 (340876.3-500050.9) | 39804.5 (32755-48050.2) | 0.01 (0-0.01) |
| Bulgaria | 1182460.9 (974083.5-1421889.4) | 39730.1 (32728.7-47774.8) | 788924 (650722.7-958281.6) | 39945.6 (32948-48520.7) | 0.03 (0.02-0.03) |
| Democratic Republic of the Congo | 4385087.3 (3528396-5451943.9) | 30673.2 (24680.7-38135.8) | 10744280.3 (8661573.5-13361375.1) | 30711.3 (24758.1-38192) | 0 (-0.01-0) |
| Norway | 748849.3 (637770-868306.6) | 46810.9 (39867.3-54278.3) | 845574.8 (718352.7-985586.2) | 48047.8 (40818.7-56003.6) | 0.12 (0.1-0.14) |
| Algeria | 2942470.8 (2353458.2-3634342.1) | 29057.6 (23241-35890) | 5031234.7 (4058935.9-6196463.4) | 29333.8 (23664.9-36127.4) | 0.04 (0.03-0.04) |
| Slovenia | 304891.3 (250939.4-367307) | 39788.5 (32747.8-47933.8) | 237234.2 (195081.6-287877.6) | 39933.4 (32837.9-48458.2) | 0.02 (0.01-0.02) |
| Portugal | 1642531.3 (1351124-1984804.9) | 43387.7 (35690.2-52429) | 1337084.6 (1095411.8-1605656.2) | 43936.8 (35995.4-52762.1) | 0.07 (0.05-0.08) |
| Chile | 1936798 (1577698.4-2384980.1) | 33819.5 (27549.1-41645.5) | 2393829.7 (1956700.6-2917723.8) | 35177.1 (28753.5-42875.6) | 0.16 (0.14-0.18) |
| Solomon Islands | 36759.2 (29376.7-45960.4) | 28606.9 (22861.7-35767.6) | 75618.1 (60941.9-94355.1) | 28905.3 (23295.2-36067.6) | 0.05 (0.04-0.06) |
| Cabo Verde | 41642.8 (33361.9-51744.9) | 31886.9 (25546-39622.3) | 82266.1 (66938.6-100970.6) | 33073.8 (26911.6-40593.6) | 0.09 (0.06-0.12) |
| Czechia | 1474880.8 (1215848.7-1777154.9) | 39691.8 (32720.8-47826.6) | 1216858.5 (1005019.2-1480418.5) | 39967.5 (33009.7-48624.1) | 0.04 (0.04-0.05) |
| Netherlands | 2818219.9 (2315045.8-3383715.4) | 46739.7 (38394.6-56118.3) | 2458633.4 (2018769.4-2949844.6) | 46707.2 (38351-56038.8) | 0.05 (0.01-0.09) |
| Senegal | 882744.4 (712807.6-1090910.2) | 32008.1 (25846.3-39556.2) | 1970955.6 (1597382-2431248.8) | 32154.8 (26060.2-39664.1) | 0.02 (0.02-0.02) |
| Northern Mariana Islands | 6854.4 (5473.4-8597.8) | 29257.2 (23362.4-36698.9) | 3971.5 (3131.4-4984.4) | 28526.7 (22492.8-35802.5) | -0.09 (-0.15--0.04) |
| Tunisia | 1002673 (804124-1248130.8) | 29097.1 (23335.3-36220.2) | 1300390.9 (1048830.1-1609884.2) | 29343.5 (23667-36327.3) | 0.03 (0.03-0.03) |
| Hungary | 1470326 (1210454.2-1777043.4) | 39765.1 (32736.8-48060.3) | 1136314.7 (936916.6-1374314.6) | 39876.6 (32879.2-48228.7) | 0.03 (0.02-0.03) |
| Sierra Leone | 442354.7 (359225.7-545414.5) | 32428.2 (26334.1-39983.3) | 1127162.7 (911954.6-1393418.2) | 32238.6 (26083.3-39853.9) | -0.04 (-0.04--0.03) |
| Guyana | 104546.6 (84182.1-130327.4) | 30730 (24744.2-38308) | 99652.6 (79950.8-123940.3) | 30812.5 (24720.7-38322.2) | -0.01 (-0.02-0) |
| Central African Republic | 322680.4 (259900.3-401893.2) | 30733.8 (24754.3-38278.5) | 647139.8 (520367.9-804740.6) | 30668.8 (24660.9-38137.7) | -0.01 (-0.01--0.01) |
| Germany | 12981652.7 (10675566.1-15676147.8) | 43702.6 (35939.2-52773.6) | 11413757.9 (9426337.2-13721919.8) | 44788.8 (36990-53846.3) | 0.18 (0.13-0.23) |
| Kuwait | 257654.6 (207627.6-321941.2) | 29544.1 (23807.7-36915.5) | 618263.7 (493434.5-781165) | 29612.5 (23633.7-37414.9) | 0 (0-0.01) |
| Mozambique | 1191395 (945708.6-1477216.3) | 26041.3 (20671.1-32288.7) | 2914952.5 (2286706.2-3605807.6) | 25970.5 (20373.2-32125.6) | 0 (-0.01-0.01) |
| Grenada | 10230.2 (8257.4-12717) | 30691.6 (24772.8-38152.2) | 12583.5 (10152.8-15601.1) | 30831 (24875.7-38224.6) | 0.02 (0.01-0.02) |
| Saudi Arabia | 1846595.5 (1480621-2299440.1) | 27538.7 (22080.8-34292) | 4957812.4 (4028567.9-6158825.5) | 27060.5 (21988.5-33615.8) | -0.08 (-0.12--0.05) |
| Colombia | 4334074.3 (3498870.8-5403548.8) | 30797.7 (24862.8-38397.3) | 5911370.2 (4774570-7338391.4) | 30905.1 (24961.8-38365.7) | 0 (0-0.01) |
| Russian Federation | 25771762.8 (21989522.9-30319487.8) | 44285.3 (37786.1-52100) | 21577591.7 (18233320-25162305.3) | 44935.1 (37970.7-52400.2) | 0.03 (-0.01-0.07) |
| Cameroon | 1220387 (988074.8-1508035.1) | 32129.4 (26013.2-39702.3) | 3901235.7 (3156180.8-4819456.5) | 32286.2 (26120.2-39885.3) | 0.02 (0.01-0.03) |
| Syrian Arab Republic | 1396665.8 (1113038.7-1727559.8) | 29000 (23110.8-35870.5) | 1596120.8 (1276775.6-1984314.4) | 28974.3 (23177.3-36021.2) | 0.01 (0-0.02) |
| Lithuania | 623860.4 (512114.8-746595.7) | 44780.1 (36759.1-53589.9) | 390186.4 (326671.3-459374.6) | 46462.5 (38899.2-54701.3) | 0.14 (0.11-0.17) |
| Albania | 561109.4 (462249.1-682422.2) | 39563.6 (32593-48117.3) | 384492.6 (317279.8-467602.2) | 39626.2 (32699.2-48191.6) | -0.01 (-0.02-0) |
| Chad | 673407.6 (546662.6-830208.9) | 32087.7 (26048.3-39559.2) | 1841492.2 (1481424-2276173.2) | 31806.8 (25587.6-39314.8) | -0.02 (-0.03--0.02) |
| Austria | 1289437.5 (1052523.8-1556246.4) | 42970.5 (35075.4-51861.9) | 1218662.7 (997326.1-1472213.7) | 43317.1 (35449.7-52329.5) | -0.02 (-0.03-0) |
| Rwanda | 713331.3 (566319.9-884873.6) | 26110.2 (20729.1-32389.2) | 1417290.1 (1129124.7-1756305.2) | 26176.9 (20854.6-32438.4) | 0.02 (0-0.04) |
| Belize | 22430.9 (17953.6-27981.3) | 30620.8 (24508.8-38197.8) | 55101.5 (44594-68516.8) | 30768.1 (24900.8-38259) | 0.01 (0.01-0.02) |
| Finland | 798470.8 (652907.1-960369) | 43986.3 (35967.5-52905) | 734204.4 (601426.8-883561.4) | 43885.8 (35949.2-52813.3) | -0.02 (-0.03--0.01) |
| Egypt | 6830273.1 (5460169.6-8477394.7) | 31171.5 (24918.7-38688.5) | 12903119.6 (10485944.7-15733244.1) | 31458.5 (25565.3-38358.5) | 0.04 (0.03-0.04) |
| Vanuatu | 16914.1 (13569-21108.5) | 28862.3 (23154.2-36019.6) | 34177.6 (27356.5-42762.1) | 28953.7 (23175.2-36226.1) | 0.01 (0.01-0.01) |
| Thailand | 8297900.3 (6747013.4-10293564.8) | 32035.2 (26047.8-39739.7) | 7872837.6 (6368467-9826606.3) | 32443.6 (26244.1-40494.9) | 0.04 (0.03-0.04) |
| Togo | 439152.7 (354295.3-543488.9) | 32034.9 (25844.8-39645.9) | 1034253 (840898.1-1272678.2) | 32445.7 (26380-39925.4) | 0.05 (0.04-0.05) |
| Spain | 6231432.3 (5142650.2-7485700.8) | 42017.5 (34676-50474.8) | 5562597.2 (4532887.9-6756854.5) | 42862.3 (34928-52064.6) | -0.01 (-0.04-0.03) |
| Peru | 2297342.7 (1873296-2800690.8) | 25906.2 (21124.4-31582.3) | 3710143.3 (2978050.3-4632418.9) | 27215.3 (21845.1-33980.5) | 0.2 (0.15-0.26) |
| Niue | 232.1 (188.1-288.8) | 28844.3 (23375.7-35879) | 162.3 (131.4-202.2) | 29045.1 (23512.2-36181.5) | 0.03 (0.03-0.03) |
| Turkey | 6552424.6 (5289393.9-8176735) | 26502.6 (21394-33072.4) | 9159940.5 (7419794.6-11382084) | 26908.6 (21796.7-33436.5) | -0.35 (-0.54--0.16) |
| Tonga | 10535.1 (8389.1-13213.4) | 28582.2 (22760.2-35848.6) | 11131.4 (8997.4-13890) | 28873.1 (23337.9-36028.6) | 0.04 (0.04-0.05) |
| Gambia | 121690 (98254.9-150740.9) | 32195.1 (25995-39881) | 302892.5 (245581.5-373711) | 32155.7 (26071.5-39674) | 0 (0-0) |
| Sweden | 1339191 (1143219.9-1558248.9) | 45626.4 (38949.7-53089.8) | 1466912 (1249781.8-1708263.4) | 45935.4 (39136.1-53493.1) | 0.01 (0-0.02) |
| Ukraine | 8605709.6 (7337892.6-10056741.9) | 45326 (38648.5-52968.6) | 6586211 (5566255.3-7713883.8) | 45599.9 (38538.2-53407.4) | 0.03 (0.02-0.04) |
| Estonia | 245600.5 (202644.7-299278.3) | 43240.8 (35677.9-52691.4) | 174795.6 (143634.5-213816.3) | 43447.2 (35701.8-53146.2) | 0.03 (0.02-0.03) |
| Cyprus | 133956.3 (109765.8-161052.2) | 43553.8 (35688.6-52363.6) | 222235.7 (180562.6-269882.1) | 44399.5 (36073.8-53918.5) | 0.05 (0.04-0.07) |
| Saint Kitts and Nevis | 5338.3 (4329.6-6635.6) | 30756.6 (24945.1-38230.9) | 7019.4 (5675.3-8719.2) | 30977.6 (25046.2-38479.2) | 0.02 (0.02-0.02) |
| Palau | 2025.1 (1631.2-2534.3) | 28953.5 (23321.4-36232.4) | 1779.4 (1426.3-2224) | 29150.4 (23366.2-36434.7) | 0 (-0.01-0.01) |
| Azerbaijan | 1308518.7 (1084302.3-1586081.5) | 41141.2 (34091.6-49868.1) | 1773006.1 (1465693.1-2158181.4) | 41543.1 (34342.5-50568.1) | 0.01 (0-0.02) |
| United Arab Emirates | 284230.7 (228606.5-353029.4) | 29670.2 (23863.7-36852) | 1359469.7 (1037042.4-1773054) | 30322.4 (23130.8-39547.2) | 0.06 (0.04-0.08) |
| Equatorial Guinea | 46038.3 (37139.2-57172.2) | 30688.4 (24756.4-38110) | 198941.5 (160330.6-247903.3) | 30567.7 (24635.1-38090.8) | -0.02 (-0.03--0.01) |
| Maldives | 25764 (20537.7-32191.5) | 31696.4 (25266.7-39603.9) | 81582.4 (65329.2-102491.7) | 32362.6 (25915.2-40657) | 0.05 (0.03-0.06) |
| Canada | 4937645.1 (4055246.4-5935497.5) | 44410.4 (36473.9-53385.3) | 5092901.5 (4194573.9-6129441.3) | 44365.8 (36540.2-53395.4) | -0.02 (-0.03--0.01) |
| Montenegro | 99384.1 (81972.6-120208.1) | 39642.7 (32697.6-47949.1) | 83835.6 (69063.6-101071.6) | 39760.3 (32754.5-47934.8) | 0.01 (0.01-0.01) |
| C么te d'Ivoire | 1526896.1 (1233737.2-1892783.3) | 32279.9 (26082.3-40015.1) | 3504963.3 (2854253.7-4311965.6) | 32552.5 (26509-40047.6) | 0.05 (0.04-0.06) |
| United Republic of Tanzania | 2512754.8 (1969678-3112919.6) | 25958.4 (20348.1-32158.5) | 5753705.6 (4551997.9-7125669) | 26057.1 (20614.9-32270.4) | 0.02 (0.02-0.02) |
| Somalia | 659080.9 (527674.5-812672.9) | 26104.2 (20899.6-32187.5) | 2039266.4 (1597259.6-2523502) | 26002.1 (20366.2-32176.5) | 0.04 (0.02-0.06) |
| Croatia | 721967.8 (594288-868852.7) | 39797.8 (32759.6-47894.7) | 513630.3 (423952.2-626596.1) | 39853.2 (32894.9-48618.3) | -0.16 (-0.3--0.01) |
| Bahrain | 75802.3 (60905.1-93723.3) | 29518.7 (23717.5-36497.4) | 185323.8 (148105.7-233204.3) | 29970.8 (23951.8-37714) | 0.05 (0.03-0.07) |
| Puerto Rico | 435885.5 (354204.9-540318.2) | 30823.9 (25047.8-38208.9) | 347598.6 (280765.1-431424) | 30939.7 (24990.8-38401) | 0.01 (0.01-0.01) |
| Jordan | 446883.7 (355619.2-555296.7) | 28928.6 (23020.7-35946.7) | 1462076.8 (1179806.6-1804558.6) | 29176 (23543.2-36010.3) | 0.03 (0.02-0.03) |
| Ghana | 1853412.8 (1503044.5-2289180.5) | 32237.4 (26143.3-39817) | 4430173 (3614065.9-5457285.6) | 32542.7 (26547.8-40087.6) | 0.02 (0.02-0.03) |
| Greece | 1636042.2 (1343142.1-1972949.7) | 43530.2 (35737-52494.3) | 1283439.8 (1048432.4-1551664.4) | 44131.6 (36050.8-53354.6) | 0.06 (0.06-0.07) |
| Yemen | 1340097.5 (1076366.4-1657718.8) | 29059.9 (23340.9-35947.5) | 3789358.4 (3047961.7-4676845.7) | 29128.2 (23429.2-35950.2) | 0.02 (0.01-0.03) |
| Guatemala | 890184.7 (714637-1105074.7) | 30678.8 (24628.9-38084.7) | 2437276.5 (1964733.8-3033716.2) | 30776 (24809.1-38307.4) | 0.01 (0-0.01) |
| Seychelles | 9970.7 (8096.8-12436) | 31921.2 (25921.8-39813.7) | 12466.4 (10070.9-15491.6) | 32301.3 (26094.4-40139.7) | 0.03 (0.02-0.04) |
| Guinea-Bissau | 120423.1 (97552.7-148745.9) | 32078.2 (25986-39622.9) | 260028.9 (210756.8-321238.3) | 32358.3 (26226.8-39975.2) | 0.03 (0.02-0.05) |
| Costa Rica | 395753.7 (319796.5-491302) | 30820 (24904.7-38261) | 593614.3 (479535.2-736263.2) | 30986.5 (25031.6-38432.8) | 0.01 (0.01-0.02) |
| Zambia | 856295.8 (676607.2-1066639.5) | 28227.7 (22304.3-35161.7) | 2081016.8 (1667582.7-2597764.9) | 27425.7 (21977.1-34235.9) | -0.12 (-0.16--0.08) |
| United Kingdom | 9382921.8 (7922512.7-11068418.8) | 44990.7 (37988.1-53072.6) | 9914867.9 (8370934.2-11694181.4) | 45334.8 (38275.3-53470.5) | 0 (-0.02-0.01) |
| Denmark | 893330.7 (745333.9-1061316.5) | 46823.2 (39066.1-55628.1) | 815335.5 (675364.8-979226.8) | 45233.4 (37468.1-54325.8) | -0.15 (-0.18--0.13) |
| Switzerland | 954797.9 (781283.1-1156404.3) | 36235.1 (29650.1-43886.2) | 1130413.1 (929068.7-1375447) | 40739.6 (33483.2-49570.5) | 0.39 (0.3-0.48) |
| Republic of Moldova | 754185.1 (620076.2-917581.9) | 43247.9 (35557.5-52617.6) | 570059.2 (468205.7-698005.4) | 43442.6 (35680.7-53193.1) | 0.03 (0.02-0.04) |
| France | 9300317.7 (7681686.6-11220547.6) | 42285.7 (34926.3-51016.4) | 8412867.6 (6942875.3-10123766.3) | 42313.4 (34919.9-50918.5) | -0.1 (-0.14--0.06) |
| Gabon | 117564.5 (94785-146313.9) | 30802.6 (24834.3-38335.2) | 230818.2 (186575.9-285481.7) | 31020.4 (25074.5-38366.8) | 0.02 (0.01-0.02) |
| Djibouti | 52234.1 (40983.4-64724) | 26028.9 (20422.6-32252.8) | 133785.1 (106382.3-166237.1) | 26504.9 (21076-32934.1) | 0.07 (0.06-0.07) |
| Brunei Darussalam | 47170.4 (38899.3-57555.4) | 38257.3 (31549.1-46680.1) | 77424.7 (63556.2-94076.2) | 38385.1 (31509.4-46640.4) | 0 (0-0.01) |
| American Samoa | 5845.1 (4670.7-7327) | 28871.6 (23070.6-36191.7) | 6059.4 (4876.7-7556.7) | 28745.5 (23134.6-35848.4) | -0.02 (-0.04--0.01) |
| Sri Lanka | 2372673.4 (1931006.4-2943368.8) | 32073.5 (26103.1-39788.1) | 2595130.8 (2103075.2-3224608.6) | 32290.6 (26168.1-40123.1) | 0.03 (0.03-0.03) |
| Burundi | 541716.9 (429403-671266.1) | 26150.8 (20729-32404.6) | 1206833.8 (958615.5-1495951.8) | 26116.2 (20744.7-32372.8) | -0.01 (-0.03-0.01) |
| Iraq | 1908822.9 (1524160-2358944.4) | 29032.1 (23181.6-35878.1) | 5440817.4 (4363350.1-6740108.6) | 29138.2 (23367.9-36096.6) | 0.01 (0.01-0.01) |
| Dominican Republic | 944335.5 (758283.1-1178874.5) | 30722.7 (24669.7-38353.1) | 1388445.7 (1122702.7-1718818.6) | 30872.1 (24963.3-38217.9) | 0.01 (0.01-0.01) |
| Guinea | 698273.5 (566161.9-859668) | 32338.2 (26219.9-39812.6) | 1552660.3 (1258656.4-1917047.8) | 32191.7 (26096.1-39746.7) | -0.02 (-0.03--0.01) |
| Afghanistan | 1117019.9 (885671.5-1403750.3) | 28848.1 (22873.3-36253.1) | 4371334.9 (3478804.2-5424793.3) | 29007.5 (23084.8-35998.1) | 0.01 (0-0.02) |
| North Macedonia | 322171.8 (265590.9-389804.9) | 39655.6 (32691.1-47980.4) | 307966.6 (253115.8-372700.8) | 39860.2 (32760.9-48238.8) | 0.01 (0.01-0.02) |
| Honduras | 528668.1 (423528.4-656033.2) | 30637.1 (24544.1-38018.1) | 1299724.5 (1047273.2-1622379.7) | 30757.4 (24783.3-38392.9) | 0.02 (0.01-0.02) |
| Bangladesh | 14015495.3 (11326840.2-17299385.8) | 32837 (26537.7-40530.8) | 22361926.5 (18145607.4-27509172.1) | 32979.8 (26761.5-40571) | 0.01 (0.01-0.01) |
| Tokelau | 166.6 (134.4-207.7) | 28908.7 (23318-36044.2) | 140.1 (112.9-174.7) | 28885.1 (23278.7-36028.1) | 0.01 (0-0.02) |
| Lesotho | 211221.6 (169906.3-261868) | 30616.8 (24628.1-37958) | 295508.4 (239267.4-364860.2) | 31106.3 (25186.2-38406.6) | 0.07 (0.05-0.08) |
| Uganda | 1664384.1 (1299913.3-2063717) | 25934.8 (20255.5-32157.3) | 4187005.7 (3283818.4-5192841.3) | 25914.5 (20324.4-32139.9) | 0 (-0.01-0) |
| Argentina | 4266653.3 (3491836.6-5215811.4) | 34909 (28569.6-42674.8) | 6110781.9 (4989771.8-7463904.7) | 35082.5 (28646.7-42850.9) | 0.03 (0.02-0.03) |
| Tuvalu | 1050 (846.7-1312.8) | 29141.5 (23499.5-36433.1) | 1381.9 (1107.9-1727.4) | 28853.9 (23134.2-36069.6) | -0.05 (-0.05--0.04) |
| Barbados | 33747.1 (27364.3-41760.7) | 30905.4 (25060.1-38244.2) | 30633.6 (24817.3-38085.8) | 30970 (25089.7-38504) | 0 (0-0) |
| San Marino | 3931.8 (3215.3-4759.5) | 43386.6 (35479.8-52519.4) | 4500.7 (3696.6-5397.3) | 43736.1 (35922.9-52449.6) | 0.02 (0-0.04) |
| Comoros | 44741.5 (35078.2-55431.9) | 25931.7 (20330.9-32127.7) | 77818.8 (61917.1-96305.2) | 26266.5 (20899.1-32506.3) | 0.05 (0.04-0.05) |
| Botswana | 157298.5 (126354.8-194730.1) | 30569.4 (24555.8-37843.8) | 331597.3 (268251.1-406267.7) | 31458 (25448.5-38541.9) | 0.11 (0.09-0.12) |
| Sudan | 2216061.3 (1772284.9-2750292.4) | 29058.3 (23239.2-36063.4) | 5016331.7 (4037667.1-6234195.1) | 29067.4 (23396.5-36124.4) | 0 (0-0) |

Abbreviations: EAPC, estimated annual percentage change; SDI, Sociodemographic Index; UI, uncertainty interval.
