# Supplementary material for: Global, regional, and national epidemiology of migraine and tension-type headache in youths and young adults aged 15–39 years from 1990 to 2019: findings from the global burden of disease study 2019
Source: J Headache Pain. 2023 Sep 18;24(1):126. doi: 10.1186/s10194-023-01659-1 (PMC10506184; doi:10.1186/s10194-023-01659-1)
Supplement: Supplementary file 13 — Additional file 13: Table S6. Incidence of Migraine Between 1990 and 2019 in 15 to 39 years at the 204 Countries Level. [file 10194_2023_1659_MOESM13_ESM.docx]

| **TableS6 Incidence of Migraine Between 1990 and 2019 in 15 to 39 years at the 204 Countries Level** | | | | | |
| --- | --- | --- | --- | --- | --- |
|  | 1990 | | 2019 | |  |
| Location | Number_95%UI | ASR | Number_95%UI | ASR | EAPC_95%CI |
| Mexico | 490728.8 (399104.1-595604.3) | 1376.8 (1119.7-1671) | 694225 (570412-836070.8) | 1383.5 (1136.7-1666.1) | 0.05 (0.03-0.06) |
| Haiti | 32390.1 (24971-40944.8) | 1332.8 (1027.6-1684.9) | 70418.2 (54587.2-88506.3) | 1324.9 (1027-1665.2) | -0.03 (-0.03--0.02) |
| Viet Nam | 475574.4 (366588.5-605290.2) | 1667.3 (1285.2-2122.1) | 644321.7 (503548.3-798386.5) | 1633.3 (1276.5-2023.9) | -0.06 (-0.07--0.06) |
| Bhutan | 3857.3 (2933.3-4959.4) | 1483.5 (1128.1-1907.3) | 5216 (4040.3-6633.8) | 1485.3 (1150.5-1889) | -0.02 (-0.03--0.01) |
| Jamaica | 12967.6 (9917.1-16494.8) | 1319.7 (1009.3-1678.7) | 15562.3 (12022.9-19608) | 1303.9 (1007.4-1642.9) | -0.03 (-0.04--0.02) |
| Nicaragua | 19565.4 (14826.4-25289.6) | 1328.7 (1006.9-1717.4) | 36419.2 (28041.5-46502.9) | 1298.3 (999.6-1657.7) | -0.09 (-0.09--0.08) |
| Kyrgyzstan | 26006.8 (20156.4-32574.2) | 1441.5 (1117.2-1805.5) | 38144.1 (29788.7-47340.2) | 1432.4 (1118.6-1777.7) | -0.01 (-0.02-0) |
| Georgia | 30699 (24026.2-38078) | 1441.7 (1128.3-1788.2) | 16763.6 (13143.1-20759.4) | 1416.7 (1110.7-1754.4) | -0.06 (-0.07--0.05) |
| Lebanon | 18168.3 (14071-23013.4) | 1508.8 (1168.6-1911.2) | 30817.7 (24179.2-38521.9) | 1501.2 (1177.8-1876.4) | -0.01 (-0.02-0) |
| Kazakhstan | 97472.1 (76101-121131.2) | 1434.5 (1120-1782.6) | 99421.5 (78151.3-123661.7) | 1430.8 (1124.7-1779.7) | 0 (-0.01-0.01) |
| Namibia | 7595.3 (5844.5-9627.6) | 1358.3 (1045.2-1721.8) | 13754.6 (10788.8-17394.9) | 1347.2 (1056.7-1703.7) | -0.03 (-0.03--0.03) |
| Republic of Korea | 279446 (216424-348877.4) | 1326.5 (1027.4-1656.1) | 209729.7 (164261.7-260972.8) | 1210 (947.7-1505.7) | -0.33 (-0.35--0.31) |
| Timor-Leste | 5224.5 (4035.7-6617.8) | 1642.2 (1268.5-2080.1) | 8950.3 (6818.4-11391.6) | 1665 (1268.4-2119.1) | 0.06 (0.06-0.06) |
| China | 6724737.4 (5522843.1-8110505.9) | 1223.8 (1005.1-1476) | 6444466 (5314657.2-7757321.7) | 1294.8 (1067.8-1558.6) | 0.18 (0.14-0.23) |
| Eritrea | 11622.9 (8904.7-14727.7) | 1004 (769.2-1272.1) | 28707.4 (22068.7-36281.5) | 998.5 (767.6-1262) | -0.02 (-0.02--0.01) |
| Iceland | 1909.9 (1519-2347.2) | 1838.6 (1462.3-2259.5) | 2173.8 (1733.2-2658.6) | 1812.7 (1445.2-2216.9) | -0.03 (-0.04--0.01) |
| Panama | 13125.1 (9974.4-16846.4) | 1298.8 (987-1667) | 20627.9 (15911.2-26432.3) | 1290.5 (995.5-1653.7) | -0.01 (-0.01-0) |
| Serbia | 48213.9 (38131.5-60057) | 1402 (1108.8-1746.4) | 39449.9 (31160.2-49027.1) | 1397.1 (1103.5-1736.3) | -0.01 (-0.02-0.01) |
| India | 5346695.1 (4379468.7-6446461.3) | 1569 (1285.1-1891.7) | 9385861.7 (7687456.7-11368374.4) | 1574.4 (1289.5-1906.9) | -0.08 (-0.11--0.04) |
| Libya | 23959.9 (18265.3-30599.4) | 1462.3 (1114.8-1867.5) | 44854 (35229.6-56250.4) | 1482.2 (1164.2-1858.8) | 0 (-0.02-0.02) |
| South Africa | 228625.2 (186079.7-276028.4) | 1437.8 (1170.3-1736) | 337436.1 (277227.7-406286.9) | 1408.9 (1157.5-1696.4) | -0.07 (-0.08--0.06) |
| Democratic People's Republic of Korea | 107136.9 (83294.7-135173.8) | 1297.9 (1009.1-1637.5) | 126667.5 (99677-158726.8) | 1260.8 (992.1-1579.9) | -0.13 (-0.14--0.12) |
| Uruguay | 14945.2 (11647.1-18621.5) | 1315.8 (1025.5-1639.5) | 15821.6 (12312-19681.7) | 1310 (1019.4-1629.7) | -0.01 (-0.02--0.01) |
| Japan | 547046.2 (452695.5-654241.9) | 1219.8 (1009.4-1458.8) | 402096.4 (334729.5-480752) | 1222.9 (1018-1462.1) | 0.01 (-0.06-0.08) |
| Poland | 215042.9 (178130.2-256261.5) | 1487.2 (1231.9-1772.3) | 186676.8 (154507.4-222016.9) | 1477 (1222.5-1756.6) | -0.04 (-0.05--0.03) |
| Saint Vincent and the Grenadines | 597.8 (456.3-761.7) | 1302.2 (994.1-1659.3) | 544.8 (422.8-682.2) | 1297.9 (1007.2-1625.1) | 0 (0-0.01) |
| Australia | 103717.4 (81649.7-129200.4) | 1531.4 (1205.6-1907.7) | 125973.5 (99663.3-156296.3) | 1516.5 (1199.7-1881.5) | -0.01 (-0.02--0.01) |
| Cook Islands | 116.6 (90.2-146.9) | 1509.5 (1167.8-1900.7) | 90.5 (70.6-113.3) | 1534.2 (1197.3-1921.9) | 0.07 (0.05-0.08) |
| Liberia | 10124.9 (7759.6-12928.9) | 1516.7 (1162.4-1936.7) | 29631.6 (22767.2-37879.4) | 1492.7 (1146.9-1908.2) | -0.06 (-0.07--0.05) |
| Greenland | 444.1 (348.6-550.3) | 1678.8 (1317.9-2080.5) | 353 (281-433.4) | 1733.7 (1379.9-2128.4) | 0.25 (0.19-0.3) |
| Tajikistan | 30654.4 (23434.7-38532) | 1448.8 (1107.6-1821.2) | 58006.7 (45209.2-72480.2) | 1430.9 (1115.2-1787.9) | -0.03 (-0.04--0.03) |
| Fiji | 4879.4 (3789.9-6107.4) | 1512.2 (1174.6-1892.8) | 5389.1 (4230.3-6759.5) | 1503.7 (1180.4-1886.1) | -0.02 (-0.02--0.02) |
| Bermuda | 334.2 (260.9-417.2) | 1298.3 (1013.6-1620.9) | 237.1 (185.9-295.8) | 1306.8 (1024.4-1630.2) | 0.03 (0.02-0.03) |
| Israel | 36089.5 (28550-44622.6) | 1887.9 (1493.5-2334.3) | 60406.6 (48112.2-74346.5) | 1861.6 (1482.7-2291.2) | -0.09 (-0.11--0.07) |
| United States Virgin Islands | 527.5 (410.6-657.4) | 1331.5 (1036.3-1659.4) | 401.1 (312.2-499.8) | 1323.1 (1029.8-1648.6) | -0.02 (-0.03-0) |
| Pakistan | 640362.8 (522671.6-776895.2) | 1563.9 (1276.5-1897.3) | 1419323.6 (1156317.1-1727099.1) | 1551.7 (1264.2-1888.2) | -0.03 (-0.04--0.01) |
| Guam | 938.1 (731.1-1172.5) | 1479.9 (1153.3-1849.6) | 905.9 (707.2-1137.2) | 1496.2 (1168-1878.2) | 0.05 (0.03-0.07) |
| Mauritania | 11504.3 (8777.9-14699.5) | 1497 (1142.2-1912.8) | 24123.9 (18379.7-30799.8) | 1501.4 (1143.9-1916.8) | 0.01 (0.01-0.01) |
| Cambodia | 65212.6 (50077.8-82734.9) | 1682 (1291.6-2134) | 116893.7 (90805.1-146838.7) | 1643.8 (1276.9-2064.9) | -0.07 (-0.07--0.06) |
| Singapore | 14527.1 (11552.8-17955.6) | 962.5 (765.5-1189.7) | 23024 (18051.8-28472.4) | 1081.9 (848.2-1337.9) | 0.45 (0.29-0.61) |
| Nepal | 113085.1 (86267-143689.1) | 1548.5 (1181.3-1967.6) | 204354.4 (155915.5-256629.8) | 1562.9 (1192.4-1962.7) | 0.05 (0.04-0.05) |
| South Sudan | 23174.4 (17689.9-29329.1) | 992.6 (757.7-1256.2) | 34703.4 (26455.4-44185.1) | 1015.1 (773.8-1292.4) | 0.08 (0.08-0.09) |
| Slovakia | 28740.9 (22767.2-35840.7) | 1403.7 (1112-1750.5) | 24873.4 (19635.1-30905.2) | 1392.5 (1099.3-1730.2) | -0.04 (-0.05--0.03) |
| Mongolia | 12770.3 (9730.4-16046.8) | 1446.2 (1101.9-1817.2) | 19320 (15183.5-24048.9) | 1426 (1120.7-1775) | -0.04 (-0.05--0.04) |
| Italy | 433812.9 (362439.2-516458.1) | 2031.9 (1697.6-2418.9) | 337412.7 (283149.9-401358.1) | 2095.3 (1758.3-2492.4) | 0.22 (0.16-0.27) |
| Kiribati | 464.9 (360.2-584.7) | 1522.8 (1179.8-1915.1) | 742.3 (578.4-925.4) | 1521.7 (1185.8-1897) | -0.01 (-0.01-0) |
| Ireland | 25875 (20442.7-32033.5) | 1886.1 (1490.2-2335.1) | 29635.6 (23590.4-36329.1) | 1843 (1467.1-2259.3) | -0.16 (-0.2--0.12) |
| Andorra | 439.8 (350.3-537.2) | 1758.8 (1400.6-2148.1) | 454.9 (364.2-557.7) | 1793.4 (1435.8-2198.9) | 0.07 (0.03-0.1) |
| Kenya | 94316.1 (76215.5-114873.7) | 1077.4 (870.7-1312.3) | 231734.9 (188368.3-282394.8) | 1069.5 (869.3-1303.3) | -0.03 (-0.03--0.03) |
| Mali | 44877.3 (34244.6-57325.7) | 1507.7 (1150.5-1925.9) | 123126.5 (93506.6-158170.9) | 1506.3 (1143.9-1935) | 0.01 (0-0.01) |
| Morocco | 157167.2 (121569.6-199259.9) | 1510.5 (1168.4-1915.1) | 219593.4 (171514-276147.8) | 1502.1 (1173.2-1888.9) | -0.02 (-0.03--0.01) |
| Romania | 122073.9 (95651.4-153300.5) | 1404.7 (1100.6-1764) | 78333.9 (61404.1-97475.1) | 1394.4 (1093.1-1735.2) | -0.04 (-0.05--0.03) |
| Zimbabwe | 54010 (41318.4-68575.5) | 1363.8 (1043.3-1731.6) | 84269.6 (65538.6-106412.5) | 1358.8 (1056.7-1715.8) | -0.02 (-0.02--0.01) |
| Eswatini | 4158.9 (3180.6-5276) | 1379.9 (1055.3-1750.6) | 6821.4 (5340.5-8624) | 1351 (1057.7-1707.9) | -0.07 (-0.07--0.06) |
| United States of America | 1909352.5 (1600707.3-2257419.1) | 1873.3 (1570.5-2214.8) | 2069927.6 (1726519.9-2447565.2) | 1881.6 (1569.5-2224.9) | 0.03 (0.01-0.05) |
| Turkmenistan | 22235.6 (17132.7-28044.2) | 1446 (1114.2-1823.8) | 28541.9 (22337.3-35460.3) | 1403.6 (1098.5-1743.9) | -0.11 (-0.11--0.11) |
| Venezuela (Bolivarian Republic of) | 102947.1 (80187.1-129502) | 1284.6 (1000.6-1615.9) | 139254.4 (108242.8-174615) | 1309.3 (1017.7-1641.8) | 0.05 (0.03-0.06) |
| Marshall Islands | 260.6 (200.5-328) | 1515.7 (1166-1907.3) | 357.5 (279.6-449.3) | 1503.4 (1175.5-1889.4) | -0.04 (-0.05--0.04) |
| Trinidad and Tobago | 6526 (5047.1-8208.3) | 1300.9 (1006.1-1636.3) | 6598.7 (5169.8-8243.3) | 1292.6 (1012.7-1614.7) | -0.04 (-0.06--0.03) |
| Taiwan (Province of China) | 116640.3 (92101.6-145412.5) | 1264.5 (998.4-1576.4) | 105301.3 (82253-131223.9) | 1326.1 (1035.9-1652.6) | 0.25 (0.2-0.31) |
| Angola | 52824.9 (41136.1-66780.5) | 1342.6 (1045.5-1697.3) | 152342.8 (118267-192724.4) | 1359.7 (1055.6-1720.1) | 0.05 (0.04-0.05) |
| Palestine | 11439.4 (8726.8-14598.4) | 1494.2 (1139.9-1906.8) | 31269.4 (24209.9-39658.7) | 1496.3 (1158.5-1897.8) | 0.02 (0.01-0.02) |
| Suriname | 2087.3 (1595.5-2647.5) | 1291.6 (987.3-1638.2) | 2806.6 (2176.5-3509.9) | 1306.8 (1013.4-1634.3) | 0.05 (0.05-0.05) |
| Saint Lucia | 742.2 (567.2-946.7) | 1319.9 (1008.7-1683.6) | 877.6 (684-1096.6) | 1299.7 (1013.1-1624) | -0.05 (-0.06--0.05) |
| Niger | 41831.9 (31803.7-53488.7) | 1505.3 (1144.4-1924.7) | 122070.8 (92267.8-156473.6) | 1502.3 (1135.5-1925.7) | 0 (0-0.01) |
| Bahamas | 1547 (1191.3-1961.5) | 1310.3 (1009-1661.4) | 1962.6 (1525.9-2452.6) | 1311.1 (1019.3-1638.4) | 0.02 (0.01-0.02) |
| Ethiopia | 177578.8 (144971.3-216134) | 951.9 (777.1-1158.6) | 427497.3 (347594.7-520527.4) | 963.1 (783.1-1172.7) | 0.07 (0.05-0.09) |
| Micronesia (Federated States of) | 605.8 (467-762.1) | 1516.4 (1168.9-1907.5) | 637.8 (492.3-802.8) | 1508.8 (1164.7-1899.1) | -0.03 (-0.03--0.02) |
| Lao People's Democratic Republic | 25849.5 (19833-32887.6) | 1672.4 (1283.1-2127.7) | 51836.3 (40342-65555.1) | 1646.5 (1281.4-2082.3) | -0.05 (-0.05--0.05) |
| Belarus | 51603.3 (40210.7-64490.2) | 1305.7 (1017.4-1631.8) | 39351.7 (30729-49426.5) | 1289.9 (1007.2-1620.1) | -0.03 (-0.03--0.02) |
| Malta | 2515.3 (2009.1-3081) | 1823.6 (1456.6-2233.8) | 2451.5 (1955.8-2988) | 1777.1 (1417.8-2166) | -0.12 (-0.16--0.09) |
| Samoa | 1002.8 (765.5-1270.1) | 1503.2 (1147.5-1903.9) | 1258.6 (968.3-1583.1) | 1512.5 (1163.6-1902.5) | 0.03 (0.02-0.03) |
| Brazil | 887863.4 (713902.9-1087066.1) | 1414.9 (1137.7-1732.4) | 1149021.7 (932368.9-1399950) | 1334.2 (1082.6-1625.5) | -0.29 (-0.38--0.2) |
| Dominica | 376.7 (288.1-478.6) | 1291.6 (987.7-1640.9) | 333.4 (258.4-417.3) | 1293.3 (1002.3-1618.5) | 0.03 (0.01-0.04) |
| Latvia | 12440.5 (9692-15575) | 1303.9 (1015.8-1632.4) | 7241.9 (5668.6-9068.5) | 1290 (1009.7-1615.4) | -0.03 (-0.04--0.03) |
| Uzbekistan | 124039.8 (95761.2-156400.9) | 1443.9 (1114.8-1820.7) | 204107.1 (159705.1-252984.8) | 1430.8 (1119.5-1773.4) | -0.02 (-0.03--0.01) |
| Philippines | 459498.1 (378150.8-549052.9) | 1769.8 (1456.5-2114.7) | 808936 (669331.7-963744.8) | 1753.7 (1451-2089.3) | -0.03 (-0.03--0.03) |
| Luxembourg | 2600.4 (2073.7-3202.9) | 1761.5 (1404.7-2169.6) | 3759.4 (3000.1-4619.5) | 1761.4 (1405.7-2164.4) | 0.01 (-0.01-0.03) |
| Mauritius | 8194.8 (6373.5-10280.8) | 1640.3 (1275.7-2057.8) | 7680.8 (5982.3-9564.5) | 1638.8 (1276.4-2040.7) | -0.01 (-0.01-0) |
| Paraguay | 20261.3 (15554.4-26219) | 1293.6 (993.1-1673.9) | 38298.7 (29638.6-49270.3) | 1288.6 (997.3-1657.8) | -0.03 (-0.03--0.02) |
| Benin | 25807.3 (19754.5-32918.3) | 1515.5 (1160-1933) | 73320.3 (55865.3-93725.9) | 1500.2 (1143-1917.7) | -0.04 (-0.04--0.03) |
| Malaysia | 115150.4 (90419.8-144701.2) | 1553.8 (1220.1-1952.5) | 214354.3 (166344.2-270045.4) | 1555.4 (1207-1959.5) | 0.03 (0.01-0.04) |
| Ecuador | 42021.3 (32439.5-53579.3) | 1019.4 (787-1299.8) | 75750.3 (57767.8-97485.1) | 1050.4 (801-1351.8) | 0.15 (0.12-0.19) |
| Monaco | 164.4 (131.6-200.6) | 1796.1 (1437.2-2191.5) | 171 (136.5-208.7) | 1842.1 (1470.5-2248.3) | 0.17 (0.14-0.21) |
| Qatar | 2996 (2332.6-3779.4) | 1267.7 (986.9-1599.1) | 20402.2 (15711.5-25926.4) | 1187.6 (914.6-1509.2) | -0.37 (-0.48--0.26) |
| El Salvador | 27759.5 (21036.1-35835.8) | 1330.7 (1008.4-1717.9) | 34189.6 (26168.8-43790.9) | 1326.9 (1015.6-1699.6) | 0 (-0.01-0.01) |
| Armenia | 20694.7 (16170.1-25719.7) | 1439.4 (1124.7-1788.9) | 15902.1 (12530-19692.2) | 1426.4 (1123.9-1766.3) | -0.02 (-0.04-0.01) |
| Iran (Islamic Republic of) | 368158.2 (300483.1-448328.3) | 1629.5 (1330-1984.3) | 578853.2 (480486.3-691397.9) | 1625.6 (1349.4-1941.7) | 0.04 (0.01-0.07) |
| Cuba | 63283.4 (48733.1-79986.9) | 1301.1 (1001.9-1644.5) | 46597.3 (36213.4-58341.8) | 1284.9 (998.5-1608.7) | -0.01 (-0.03-0) |
| Nigeria | 541368.8 (444627.1-660410.4) | 1589.5 (1305.4-1939) | 1358930.3 (1101964.2-1647735.3) | 1620.5 (1314.1-1964.9) | 0.05 (0.05-0.06) |
| Myanmar | 280673.5 (217392.1-355504) | 1657.2 (1283.6-2099.1) | 366068.9 (284261.2-458983.5) | 1656.6 (1286.4-2077) | 0 (0-0) |
| Malawi | 36656.9 (28062.3-46260.1) | 1013.7 (776-1279.2) | 77407.2 (59090.6-97894.9) | 1015.6 (775.2-1284.3) | 0.01 (0-0.01) |
| Oman | 10870.3 (8474.7-13731) | 1354 (1055.6-1710.4) | 33256.5 (25863.4-41910.2) | 1297.1 (1008.8-1634.7) | -0.21 (-0.35--0.07) |
| Congo | 12843.8 (9889.3-16280.9) | 1354 (1042.6-1716.4) | 28380.7 (22295.7-35691.8) | 1347.8 (1058.8-1695) | -0.02 (-0.02--0.01) |
| Madagascar | 45633.9 (35015.9-57776.5) | 1008.6 (773.9-1276.9) | 109808.1 (84395-138939.9) | 1005.2 (772.6-1271.9) | -0.01 (-0.01--0.01) |
| Papua New Guinea | 24985.1 (19264.6-31328.2) | 1509.7 (1164-1893) | 61739.7 (48131.5-77351.5) | 1508.6 (1176.1-1890.1) | 0 (-0.01-0) |
| Indonesia | 1384563 (1144163.6-1655072.4) | 1773.7 (1465.7-2120.2) | 1852918.5 (1537645.3-2195700.4) | 1749.3 (1451.7-2072.9) | -0.06 (-0.06--0.05) |
| New Zealand | 22476 (18693.2-26684.5) | 1626.3 (1352.6-1930.8) | 22932.8 (19099.7-27278.7) | 1626 (1354.3-1934.2) | 0.04 (0.02-0.05) |
| Bolivia (Plurinational State of) | 24723.8 (19016-31613.2) | 1003.6 (771.9-1283.2) | 48033.6 (37105-60813.5) | 993.5 (767.4-1257.8) | -0.04 (-0.05--0.04) |
| Sao Tome and Principe | 644.8 (487.5-827) | 1501.7 (1135.4-1926.3) | 1288.6 (990.9-1646.9) | 1483.9 (1141-1896.5) | -0.05 (-0.06--0.05) |
| Antigua and Barbuda | 339.5 (262.6-429) | 1316.1 (1018-1662.9) | 452.4 (352.4-566.2) | 1307.2 (1018.3-1635.9) | -0.03 (-0.05-0) |
| Belgium | 70545.8 (56163.6-87557.4) | 1896.4 (1509.8-2353.7) | 68592.4 (54371.3-84105.1) | 1956.1 (1550.5-2398.5) | 0.17 (0.13-0.21) |
| Nauru | 61.2 (47.5-76.7) | 1513.7 (1174.8-1896.3) | 69 (53.6-86.4) | 1512.5 (1173.9-1893) | -0.01 (-0.01-0) |
| Burkina Faso | 48575 (36951.1-62134.2) | 1521.2 (1157.2-1945.9) | 128476.5 (98010.5-164218.9) | 1505.1 (1148.2-1923.8) | -0.04 (-0.04--0.04) |
| Bosnia and Herzegovina | 26911.5 (21127.1-33602.6) | 1393.3 (1093.8-1739.8) | 14588.1 (11484.8-18250.5) | 1401.8 (1103.6-1753.7) | -0.01 (-0.03-0.01) |
| Bulgaria | 41880.6 (33122.2-52224.9) | 1407.2 (1112.9-1754.7) | 27433.5 (21544.6-34175.4) | 1389 (1090.9-1730.4) | -0.05 (-0.06--0.05) |
| Democratic Republic of the Congo | 192960.2 (149625.7-244703) | 1349.7 (1046.6-1711.7) | 470212.7 (364757-595529.8) | 1344 (1042.6-1702.3) | -0.01 (-0.01--0.01) |
| Norway | 31590.8 (26518.1-37450.1) | 1974.8 (1657.7-2341) | 33755.6 (28305.8-40044.3) | 1918.1 (1608.4-2275.4) | -0.08 (-0.11--0.04) |
| Algeria | 151578.7 (116448.5-193292) | 1496.9 (1150-1908.8) | 257065.1 (202218.1-322002.1) | 1498.8 (1179-1877.4) | 0.01 (0-0.01) |
| Slovenia | 10734.2 (8491.4-13399.5) | 1400.8 (1108.1-1748.6) | 8228.3 (6488.2-10267.8) | 1385.1 (1092.2-1728.4) | -0.06 (-0.06--0.05) |
| Portugal | 71009.6 (56426.8-87619.7) | 1875.7 (1490.5-2314.5) | 55848 (44566.8-68254.7) | 1835.2 (1464.5-2242.9) | -0.12 (-0.16--0.07) |
| Chile | 74876.7 (57325-93408.2) | 1307.5 (1001-1631.1) | 88651.2 (69764.3-109916.9) | 1302.7 (1025.2-1615.2) | 0.07 (0.03-0.1) |
| Solomon Islands | 1952.8 (1493.6-2478.2) | 1519.7 (1162.4-1928.6) | 3969.6 (3071-4982.7) | 1517.4 (1173.9-1904.6) | 0 (-0.01-0) |
| Cabo Verde | 1971.5 (1492.9-2529.2) | 1509.6 (1143.1-1936.7) | 3638.2 (2834.6-4608) | 1462.7 (1139.6-1852.6) | -0.1 (-0.11--0.1) |
| Czechia | 52212.4 (41216.5-65471.8) | 1405.1 (1109.2-1762) | 42383.3 (33478.1-52681.6) | 1392.1 (1099.6-1730.3) | -0.05 (-0.05--0.04) |
| Netherlands | 102677.6 (81028.4-126020.5) | 1702.9 (1343.8-2090) | 95886.5 (75726.8-117453.2) | 1821.6 (1438.6-2231.3) | 0.41 (0.32-0.5) |
| Senegal | 41585.1 (31645.8-53307.8) | 1507.9 (1147.5-1932.9) | 91327.6 (69687.3-116703.6) | 1489.9 (1136.9-1903.9) | -0.04 (-0.04--0.04) |
| Northern Mariana Islands | 352.9 (277.8-441.9) | 1506.5 (1185.8-1886.2) | 210.2 (159.9-266.3) | 1510.2 (1148.4-1913) | -0.02 (-0.08-0.04) |
| Tunisia | 51827.1 (40129.7-65865.1) | 1504 (1164.5-1911.4) | 66882.6 (52497.4-83818.7) | 1509.2 (1184.6-1891.4) | 0.02 (0.02-0.03) |
| Hungary | 51890.4 (40819.4-65035.8) | 1403.4 (1104-1758.9) | 39849.9 (31367.5-49668.5) | 1398.5 (1100.8-1743) | -0.03 (-0.03--0.02) |
| Sierra Leone | 20512.7 (15731.3-26173.1) | 1503.7 (1153.2-1918.7) | 52336.9 (39970.4-66870.5) | 1496.9 (1143.2-1912.6) | -0.04 (-0.05--0.03) |
| Guyana | 4470.7 (3427.2-5672.9) | 1314.1 (1007.4-1667.5) | 4227.3 (3258.7-5327.1) | 1307.1 (1007.6-1647.1) | 0.01 (-0.01-0.02) |
| Central African Republic | 14218.8 (11017.4-18065.9) | 1354.3 (1049.4-1720.7) | 28664.3 (22165.6-36268.5) | 1358.4 (1050.5-1718.8) | 0.02 (0.01-0.03) |
| Germany | 542352.6 (432124-669829.1) | 1825.8 (1454.7-2255) | 475019.8 (371720.6-580541.7) | 1864 (1458.7-2278.1) | 0.1 (0-0.19) |
| Kuwait | 12361.9 (9690.1-15591.2) | 1417.5 (1111.1-1787.8) | 30968.4 (24434.9-38905.1) | 1483.3 (1170.3-1863.4) | 0.18 (0.12-0.24) |
| Mozambique | 47049.7 (36145.1-59361.7) | 1028.4 (790.1-1297.5) | 114697.8 (87560.5-145223.8) | 1021.9 (780.1-1293.9) | -0.03 (-0.03--0.02) |
| Grenada | 434.6 (334.1-551.1) | 1303.7 (1002.2-1653.3) | 523.6 (404.7-660.1) | 1282.8 (991.6-1617.3) | -0.05 (-0.05--0.04) |
| Saudi Arabia | 94893.6 (72657.7-120814.8) | 1415.2 (1083.6-1801.7) | 255792.5 (199532.3-319866.3) | 1396.2 (1089.1-1745.9) | -0.04 (-0.06--0.03) |
| Colombia | 185043.9 (142018.3-234596.8) | 1314.9 (1009.2-1667) | 249059.8 (193188.9-315157.1) | 1302.1 (1010-1647.7) | -0.03 (-0.04--0.03) |
| Russian Federation | 824565.9 (677578.6-991100.8) | 1416.9 (1164.3-1703.1) | 676048.5 (558259.3-818891.1) | 1407.9 (1162.6-1705.3) | 0 (-0.02-0.03) |
| Cameroon | 57210.2 (43565.4-73057.2) | 1506.2 (1147-1923.4) | 180437.9 (138038.4-230515.3) | 1493.3 (1142.4-1907.7) | -0.03 (-0.04--0.03) |
| Syrian Arab Republic | 72106 (54924.6-91981.1) | 1497.2 (1140.4-1909.9) | 84574.3 (64928.1-107547.1) | 1535.3 (1178.6-1952.3) | 0.1 (0.06-0.14) |
| Lithuania | 17715.8 (13750-22438.1) | 1271.6 (987-1610.6) | 10302.3 (8150.8-12795.5) | 1226.8 (970.6-1523.7) | -0.14 (-0.16--0.11) |
| Albania | 19980.8 (15656.5-24994.2) | 1408.8 (1103.9-1762.3) | 13524.6 (10663-16847.2) | 1393.9 (1098.9-1736.3) | -0.04 (-0.06--0.01) |
| Chad | 31667.4 (24152.7-40412.8) | 1508.9 (1150.9-1925.7) | 87370.8 (66303.5-111954) | 1509.1 (1145.2-1933.7) | 0 (0-0) |
| Austria | 53497.5 (41987-66295.3) | 1782.8 (1399.2-2209.3) | 49331.8 (39090.6-61052.7) | 1753.5 (1389.5-2170.1) | 0.06 (0.02-0.11) |
| Rwanda | 27548.6 (21167.9-34822.8) | 1008.4 (774.8-1274.6) | 54416.6 (41757.8-68627.2) | 1005.1 (771.3-1267.5) | -0.02 (-0.04-0) |
| Belize | 957.9 (729.6-1222.1) | 1307.7 (996-1668.3) | 2354.7 (1818.6-2976.3) | 1314.8 (1015.5-1661.9) | 0.02 (0.01-0.03) |
| Finland | 32719.5 (26187.3-39872.4) | 1802.5 (1442.6-2196.5) | 30208.3 (24128.3-36810.8) | 1805.6 (1442.2-2200.3) | 0.01 (-0.02-0.04) |
| Egypt | 330341.4 (250994.8-417852.8) | 1507.6 (1145.5-1907) | 597353.2 (467944.9-744541.6) | 1456.4 (1140.9-1815.2) | -0.16 (-0.18--0.13) |
| Vanuatu | 892.2 (689.1-1120.5) | 1522.4 (1175.9-1912) | 1798.1 (1395-2257.1) | 1523.3 (1181.8-1912.1) | 0 (-0.01-0) |
| Thailand | 460889.2 (349180-580226.4) | 1779.3 (1348.1-2240) | 413013.2 (320372.8-516764.9) | 1702 (1320.2-2129.6) | -0.17 (-0.2--0.13) |
| Togo | 20656.5 (15714.2-26387) | 1506.8 (1146.3-1924.9) | 47671.1 (36751.8-60745.8) | 1495.5 (1152.9-1905.7) | -0.02 (-0.02--0.02) |
| Spain | 278421.9 (220006-346204.1) | 1877.3 (1483.5-2334.4) | 235948.4 (186484.2-292426.7) | 1818.1 (1436.9-2253.3) | -0.17 (-0.23--0.12) |
| Peru | 74049.4 (58102-92684.2) | 835 (655.2-1045.2) | 126870.9 (99320.4-159124.4) | 930.6 (728.6-1167.2) | 0.43 (0.31-0.55) |
| Niue | 12.1 (9.4-15.2) | 1502.4 (1162.8-1887.5) | 8.4 (6.6-10.6) | 1507.9 (1182.8-1893.2) | 0 (-0.01-0.01) |
| Turkey | 366665.8 (282115.3-463830.4) | 1483.1 (1141.1-1876.1) | 497278.9 (393412.2-618271.6) | 1460.8 (1155.7-1816.3) | -0.12 (-0.16--0.08) |
| Tonga | 562 (428.6-713.5) | 1524.7 (1162.7-1935.7) | 588.5 (454.1-739.2) | 1526.5 (1177.9-1917.5) | 0.01 (0-0.02) |
| Gambia | 5675.8 (4329.3-7269.8) | 1501.6 (1145.4-1923.3) | 14137.4 (10773.9-18065.6) | 1500.9 (1143.8-1917.9) | 0 (0-0.01) |
| Sweden | 55988.3 (46846.1-66298) | 1907.5 (1596.1-2258.8) | 60516.1 (50733.5-71873.5) | 1895 (1588.7-2250.7) | 0.06 (0.02-0.1) |
| Ukraine | 262481.4 (215877.8-313745.9) | 1382.5 (1137-1652.5) | 197200 (162869.7-235246.4) | 1365.3 (1127.6-1628.7) | -0.03 (-0.05--0.02) |
| Estonia | 7387.8 (5760-9224.4) | 1300.7 (1014.1-1624.1) | 5166 (4035.7-6466.6) | 1284.1 (1003.1-1607.3) | -0.05 (-0.06--0.05) |
| Cyprus | 5624.1 (4476.5-6906.3) | 1828.6 (1455.4-2245.5) | 8807.2 (7019.5-10711.1) | 1759.6 (1402.4-2139.9) | -0.13 (-0.17--0.09) |
| Saint Kitts and Nevis | 227.1 (175-287.4) | 1308.3 (1008.3-1656) | 294.9 (229.3-369.2) | 1301.3 (1011.8-1629.5) | -0.02 (-0.02--0.01) |
| Palau | 104.9 (81.6-131.6) | 1500.4 (1166-1880.8) | 88.5 (69.8-110.1) | 1450 (1144-1804.1) | -0.09 (-0.13--0.06) |
| Azerbaijan | 46091.9 (35632.2-57757.7) | 1449.2 (1120.3-1816) | 60630.9 (47664.7-75073.1) | 1420.6 (1116.8-1759) | -0.06 (-0.07--0.05) |
| United Arab Emirates | 12487.6 (9746.3-15709.5) | 1303.6 (1017.4-1639.9) | 55537.1 (41592.6-73317.1) | 1238.7 (927.7-1635.3) | -0.24 (-0.29--0.19) |
| Equatorial Guinea | 2058.3 (1589.4-2606.7) | 1372 (1059.5-1737.6) | 8544 (6600.9-10879.2) | 1312.8 (1014.2-1671.6) | -0.17 (-0.19--0.15) |
| Maldives | 1353.2 (1032.8-1717.5) | 1664.8 (1270.6-2112.9) | 3847.2 (3032.4-4777.5) | 1526.1 (1202.9-1895.2) | -0.29 (-0.35--0.23) |
| Canada | 192791.6 (152817-236769.2) | 1734 (1374.5-2129.6) | 197734.5 (156700.1-242086.2) | 1722.5 (1365.1-2108.9) | 0.02 (-0.01-0.05) |
| Montenegro | 3498.7 (2751.4-4382) | 1395.6 (1097.5-1747.9) | 2947.4 (2329.2-3664.4) | 1397.8 (1104.6-1737.9) | 0.01 (-0.01-0.02) |
| C么te d'Ivoire | 70231.4 (53660.8-89824.3) | 1484.8 (1134.4-1899) | 159222.2 (123062.3-203061.7) | 1478.8 (1142.9-1885.9) | -0.02 (-0.02--0.02) |
| United Republic of Tanzania | 96765.2 (74605.3-122052.6) | 999.6 (770.7-1260.9) | 228309.1 (174347.6-292144) | 1034 (789.6-1323) | 0.19 (0.14-0.24) |
| Somalia | 25236.8 (19382.3-32125.2) | 999.6 (767.7-1272.4) | 78104.7 (59458.7-98849.3) | 995.9 (758.1-1260.4) | -0.02 (-0.03--0.02) |
| Croatia | 25405.7 (20105.6-31664.4) | 1400.5 (1108.3-1745.5) | 18010.9 (14148.3-22434.9) | 1397.5 (1097.8-1740.7) | -0.03 (-0.1-0.03) |
| Bahrain | 3576.1 (2791-4501.3) | 1392.6 (1086.9-1752.9) | 8457 (6565.6-10784.3) | 1367.7 (1061.8-1744) | -0.17 (-0.22--0.12) |
| Puerto Rico | 18678.4 (14472.1-23406.5) | 1320.9 (1023.4-1655.2) | 14739.1 (11467.8-18389) | 1311.9 (1020.7-1636.8) | -0.03 (-0.03--0.02) |
| Jordan | 22656.7 (17170.3-28931.9) | 1466.7 (1111.5-1872.9) | 72922.5 (56794.9-92667.8) | 1455.2 (1133.4-1849.2) | -0.03 (-0.06--0.01) |
| Ghana | 86162.4 (65808.1-109978.8) | 1498.7 (1144.6-1912.9) | 203439.6 (157040.3-259096.9) | 1494.4 (1153.6-1903.2) | 0 (-0.01-0) |
| Greece | 70841.6 (55964.4-87671.1) | 1884.9 (1489-2332.7) | 53863.8 (42414.8-66208.7) | 1852.1 (1458.5-2276.6) | -0.09 (-0.13--0.06) |
| Yemen | 69881.6 (53805.5-88931.2) | 1515.4 (1166.8-1928.5) | 195660.9 (151851.7-248424.5) | 1504 (1167.3-1909.6) | 0 (-0.01-0.01) |
| Guatemala | 38394.8 (29192.7-49360) | 1323.2 (1006.1-1701.1) | 103938.4 (79086.9-133136.3) | 1312.5 (998.6-1681.1) | -0.03 (-0.05--0.02) |
| Seychelles | 515 (397.2-655.8) | 1648.7 (1271.6-2099.5) | 617.9 (482.7-764.8) | 1601.1 (1250.7-1981.6) | -0.12 (-0.13--0.11) |
| Guinea-Bissau | 5671.5 (4323.5-7246.7) | 1510.8 (1151.7-1930.4) | 12056.2 (9245-15392.6) | 1500.3 (1150.5-1915.5) | -0.02 (-0.02--0.01) |
| Costa Rica | 16733.4 (12761.6-21379.4) | 1303.1 (993.8-1665) | 25074.2 (19529-31850.1) | 1308.9 (1019.4-1662.6) | 0.02 (0.02-0.02) |
| Zambia | 32736.8 (24457.7-41919.5) | 1079.2 (806.2-1381.9) | 79406.9 (60966.6-101277.5) | 1046.5 (803.5-1334.7) | -0.12 (-0.15--0.09) |
| United Kingdom | 392182.1 (325135.8-469037.7) | 1880.5 (1559-2249) | 409100.5 (339585.5-487397.8) | 1870.6 (1552.7-2228.6) | 0.05 (0.03-0.07) |
| Denmark | 32081.8 (25261.9-39742.8) | 1681.5 (1324.1-2083.1) | 31383.9 (25095.2-38217.1) | 1741.1 (1392.2-2120.2) | 0.21 (0.18-0.23) |
| Switzerland | 44487 (35515.5-54753.6) | 1688.3 (1347.8-2077.9) | 47616.5 (37691.8-58402.2) | 1716.1 (1358.4-2104.8) | 0.14 (0.1-0.18) |
| Republic of Moldova | 22898.6 (17803.9-28622) | 1313.1 (1020.9-1641.3) | 16916.1 (13227.2-21173.8) | 1289.1 (1008-1613.6) | -0.06 (-0.06--0.05) |
| France | 404895.2 (319061.1-498467.1) | 1840.9 (1450.7-2266.4) | 365382.8 (290843.5-447071.7) | 1837.7 (1462.8-2248.6) | 0.02 (0.01-0.03) |
| Gabon | 5118.8 (3976.6-6499.3) | 1341.2 (1041.9-1702.9) | 10086.7 (7906.9-12744.1) | 1355.6 (1062.6-1712.7) | 0.03 (0.03-0.04) |
| Djibouti | 1964.1 (1498.1-2490.6) | 978.8 (746.5-1241.1) | 4984.9 (3895.2-6252.3) | 987.6 (771.7-1238.7) | 0.04 (0.03-0.05) |
| Brunei Darussalam | 1454.5 (1124-1830.8) | 1179.7 (911.6-1484.9) | 2349.2 (1825.3-2937.3) | 1164.7 (905-1456.2) | -0.04 (-0.08--0.01) |
| American Samoa | 307.1 (237.9-386.1) | 1516.7 (1175.3-1907.3) | 320.7 (246.7-404.5) | 1521.3 (1170.3-1918.8) | 0.02 (0.01-0.03) |
| Sri Lanka | 121980.4 (94768.9-153696.7) | 1648.9 (1281.1-2077.7) | 132686.7 (103357.9-165018.2) | 1651 (1286.1-2053.3) | 0 (0-0.01) |
| Burundi | 20973.2 (16152.3-26452.1) | 1012.5 (779.7-1276.9) | 46709.9 (35859.6-59078.6) | 1010.8 (776-1278.5) | -0.01 (-0.01--0.01) |
| Iraq | 98232.6 (75188.9-125054) | 1494.1 (1143.6-1902) | 278538.4 (216457.4-352937.4) | 1491.7 (1159.2-1890.2) | 0.01 (0-0.02) |
| Dominican Republic | 40762.2 (31168.6-51960.7) | 1326.1 (1014-1690.5) | 58339.4 (45125.8-73619.6) | 1297.2 (1003.4-1636.9) | -0.09 (-0.09--0.08) |
| Guinea | 32591.4 (24969.9-41620.3) | 1509.4 (1156.4-1927.5) | 73031.9 (55723.9-93284.7) | 1514.2 (1155.3-1934.1) | 0.03 (0.02-0.04) |
| Afghanistan | 59178.2 (44941.6-75965.2) | 1528.3 (1160.7-1961.9) | 224986.4 (171342.1-286852.8) | 1493 (1137-1903.5) | -0.06 (-0.09--0.03) |
| North Macedonia | 11376.1 (8953.7-14245.7) | 1400.3 (1102.1-1753.5) | 10714.2 (8452.2-13365.5) | 1386.7 (1094-1729.9) | -0.04 (-0.05--0.03) |
| Honduras | 22837 (17366.5-29342.6) | 1323.4 (1006.4-1700.4) | 56019.3 (42617.8-71945.7) | 1325.7 (1008.5-1702.6) | 0.02 (0.01-0.02) |
| Bangladesh | 641521.5 (489210.6-817525.4) | 1503 (1146.2-1915.4) | 1026530.3 (794568.2-1303359.6) | 1513.9 (1171.8-1922.2) | 0.03 (0.03-0.03) |
| Tokelau | 8.7 (6.8-11) | 1514.1 (1174.4-1900.6) | 7.3 (5.6-9.1) | 1499.6 (1163.6-1878.3) | -0.05 (-0.07--0.02) |
| Lesotho | 9369.4 (7227-11863.7) | 1358.1 (1047.6-1719.7) | 12672.9 (9947.3-15969.8) | 1334 (1047.1-1681) | -0.07 (-0.08--0.07) |
| Uganda | 65229.7 (49626.5-82542.8) | 1016.4 (773.3-1286.2) | 164138.4 (125008.1-208077.5) | 1015.9 (773.7-1287.8) | 0 (0-0) |
| Argentina | 152277.1 (118414-189818.6) | 1245.9 (968.8-1553.1) | 224929.4 (172958.4-280333.3) | 1291.3 (993-1609.4) | 0.17 (0.13-0.21) |
| Tuvalu | 55.2 (43.2-69) | 1532.1 (1199.2-1916) | 71.5 (55.5-89.7) | 1493.3 (1159.6-1873.2) | -0.1 (-0.1--0.09) |
| Barbados | 1426.8 (1108.3-1792.1) | 1306.7 (1015-1641.2) | 1294.2 (1009-1616) | 1308.4 (1020.1-1633.7) | 0.01 (0.01-0.01) |
| San Marino | 169.1 (134.3-209.3) | 1866.2 (1482.2-2309.7) | 192.1 (153.6-234.5) | 1867.1 (1492.4-2278.8) | 0.04 (0.01-0.06) |
| Comoros | 1747.7 (1332-2217.5) | 1013 (772-1285.2) | 2961.1 (2289.1-3730.9) | 999.5 (772.7-1259.3) | -0.05 (-0.05--0.04) |
| Botswana | 7049.3 (5429.4-8924.1) | 1370 (1055.1-1734.3) | 14078.6 (11070.3-17572.6) | 1335.6 (1050.2-1667.1) | -0.09 (-0.09--0.09) |
| Sudan | 115988 (89176.5-147268.7) | 1520.9 (1169.3-1931.1) | 261004.1 (202858.1-331161) | 1512.4 (1175.5-1918.9) | 0 (-0.01-0.01) |

Abbreviations: EAPC, estimated annual percentage change; SDI, Sociodemographic Index; UI, uncertainty interval.
