# Supplementary material for: Global, regional, and national epidemiology of migraine and tension-type headache in youths and young adults aged 15–39 years from 1990 to 2019: findings from the global burden of disease study 2019
Source: J Headache Pain. 2023 Sep 18;24(1):126. doi: 10.1186/s10194-023-01659-1 (PMC10506184; doi:10.1186/s10194-023-01659-1)
Supplement: Supplementary file 14 — Additional file 14: Table S7. Incidence of TTH Between 1990 and 2019 in 15 to 39 years at the 204 Countries Level. [file 10194_2023_1659_MOESM14_ESM.docx]

| **TableS7 Incidence of TTH Between 1990 and 2019 in 15 to 39 years at the Countries Level** | | | | | |
| --- | --- | --- | --- | --- | --- |
|  | 1990 | | 2019 | |  |
| Location | Number_95%UI | ASR | Number_95%UI | ASR | EAPC_95%CI |
| Mexico | 3980978 (3214534.7-4790451.8) | 11168.8 (9018.5-13439.8) | 5637700.7 (4593364.4-6747651.5) | 11234.9 (9153.8-13446.9) | 0.02 (0.02-0.02) |
| Haiti | 251766.6 (204395.8-304506.4) | 10360.2 (8410.9-12530.4) | 551957.1 (447839.8-664628.2) | 10384.8 (8425.9-12504.7) | 0.01 (0.01-0.02) |
| Viet Nam | 3009710.7 (2409547.9-3682654.6) | 10551.8 (8447.7-12911.1) | 4232154.9 (3353155.5-5138489.6) | 10728.4 (8500.1-13025.9) | 0.04 (0.03-0.05) |
| Bhutan | 28844.6 (23143.5-35140.4) | 11093.1 (8900.6-13514.4) | 39286.6 (31719.2-47222.2) | 11187 (9032.1-13446.7) | 0.03 (0.02-0.03) |
| Jamaica | 101692.7 (82321.7-123175.9) | 10349.3 (8377.9-12535.7) | 124173.1 (100137.5-149118.8) | 10404.2 (8390.3-12494.4) | 0.01 (0-0.01) |
| Nicaragua | 152034.2 (123022.9-184504.4) | 10324.7 (8354.5-12529.7) | 291366.7 (236337.6-350492.7) | 10386.5 (8424.9-12494.2) | 0.02 (0.02-0.03) |
| Kyrgyzstan | 243408.9 (198254.4-292994.1) | 13491.5 (10988.7-16239.9) | 360570.4 (292093.1-432094.3) | 13539.9 (10968.5-16225.8) | 0 (-0.01-0.01) |
| Georgia | 288404.5 (233806.6-345847.1) | 13544.3 (10980.2-16241.9) | 160813.9 (129914.7-192803.3) | 13590.7 (10979.3-16294.2) | 0.01 (0-0.01) |
| Lebanon | 118330.2 (95698.8-143581) | 9826.9 (7947.4-11923.9) | 202994.9 (162001.4-246728.1) | 9888.1 (7891.3-12018.4) | 0.02 (0.02-0.02) |
| Kazakhstan | 919311.7 (744291.3-1102811.1) | 13529.2 (10953.5-16229.7) | 944370.2 (761254.4-1134255.1) | 13590.9 (10955.6-16323.6) | 0.01 (0-0.02) |
| Namibia | 58764.9 (47524.2-71841.7) | 10509.4 (8499.1-12848) | 108800.8 (87781.8-132470.8) | 10656.3 (8597.7-12974.7) | 0.04 (0.04-0.05) |
| Republic of Korea | 2687239 (2167800.1-3223106.8) | 12756.3 (10290.5-15300) | 2272088 (1817969.9-2752005) | 13108.8 (10488.8-15877.7) | 0.1 (0.09-0.12) |
| Timor-Leste | 33568.1 (26873.4-40931.7) | 10551.3 (8447-12865.9) | 56153.1 (45240.9-69162.8) | 10446.1 (8416.1-12866.2) | -0.05 (-0.05--0.04) |
| China | 42228518.2 (34869667.9-51168687) | 7684.9 (6345.7-9311.9) | 41755406.8 (33436031.5-50548160.1) | 8389.5 (6717.9-10156.1) | 0.39 (0.31-0.46) |
| Eritrea | 105739.3 (85480.5-128265.3) | 9133.4 (7383.6-11079.2) | 263699 (213169.9-320627.4) | 9172.4 (7414.8-11152.5) | 0.01 (0.01-0.02) |
| Iceland | 14271.1 (11604.5-17199.3) | 13738.3 (11171.2-16557.1) | 16508.8 (13385.7-19867.5) | 13765.9 (11161.8-16566.6) | 0 (0-0) |
| Panama | 104687 (84755.8-126454.7) | 10359.2 (8386.9-12513.2) | 166162.1 (134834.7-199383.5) | 10395.5 (8435.6-12474) | 0.01 (0-0.01) |
| Serbia | 450286.2 (365548-542004.6) | 13094.1 (10630-15761.3) | 370003.9 (299406.8-445286.9) | 13103.4 (10603.3-15769.5) | 0.01 (0.01-0.01) |
| India | 39986217.8 (32692038.5-48078942) | 11733.8 (9593.4-14108.6) | 70151341.1 (57190136.3-83938346.5) | 11767 (9593-14079.6) | -0.07 (-0.1--0.03) |
| Libya | 161654.9 (130353.6-196629.9) | 9866 (7955.7-12000.6) | 299999.6 (242407.6-362332.3) | 9913.5 (8010.4-11973.3) | 0.03 (0.02-0.03) |
| South Africa | 1814294.4 (1475162.1-2173114.1) | 11410.2 (9277.4-13666.8) | 2781751.8 (2231194.3-3353992.6) | 11614.9 (9316.1-14004.2) | 0.05 (0.04-0.07) |
| Democratic People's Republic of Korea | 653227.8 (527816.3-804107.8) | 7913.3 (6394.1-9741.1) | 801337.3 (647142.4-981733.3) | 7976 (6441.3-9771.6) | -0.02 (-0.04-0.01) |
| Uruguay | 136614.6 (110276.9-163476) | 12028.1 (9709.2-14393.1) | 145677.6 (117462.2-174436.6) | 12062.2 (9725.9-14443.4) | 0.01 (0.01-0.01) |
| Japan | 6121386.7 (4963068.9-7287685.8) | 13649 (11066.2-16249.5) | 4520881.6 (3680834.6-5417176.9) | 13749.7 (11194.8-16475.6) | 0.04 (0.03-0.04) |
| Poland | 2047335.3 (1660924.9-2439039.5) | 14159.2 (11486.8-16868.1) | 1795417.3 (1445763.5-2147916.2) | 14205.6 (11439.1-16994.6) | 0.02 (0.01-0.03) |
| Saint Vincent and the Grenadines | 4741.4 (3835.5-5753.1) | 10328.8 (8355.4-12532.8) | 4364.1 (3544.7-5223.4) | 10396 (8444.2-12443.1) | 0.02 (0.02-0.02) |
| Australia | 819356.3 (661010.4-989101.8) | 12098.3 (9760.2-14604.7) | 1010893.9 (811622-1218277.9) | 12169.1 (9770.2-14665.5) | 0 (0-0.01) |
| Cook Islands | 757.8 (615.4-916.4) | 9806.7 (7964-11858.5) | 584.6 (473.5-707) | 9915.5 (8030.5-11991.5) | 0.03 (0.02-0.04) |
| Liberia | 73647.3 (58667.4-89034.2) | 11031.9 (8788-13336.8) | 219708 (176139.5-264384.8) | 11068.1 (8873.2-13318.7) | 0.04 (0.03-0.05) |
| Greenland | 3703.2 (2977.8-4481.7) | 14000.1 (11257.8-16943.2) | 2851.7 (2307-3441.3) | 14005 (11329.8-16900.2) | -0.04 (-0.05--0.02) |
| Tajikistan | 284709.7 (230383-343769.6) | 13456.5 (10888.8-16247.9) | 548090.9 (445355.2-657661.3) | 13519.9 (10985.7-16222.6) | 0.01 (0-0.01) |
| Fiji | 31772.3 (25742.6-38519) | 9846.8 (7978.1-11937.8) | 35579.9 (28672.2-43053.6) | 9928 (8000.5-12013.4) | 0.03 (0.02-0.03) |
| Bermuda | 2692.5 (2172.2-3236.1) | 10460.1 (8438.7-12571.8) | 1898.5 (1533.3-2280.7) | 10463.1 (8450.4-12569.6) | 0 (-0.01-0) |
| Israel | 261762.6 (213348.5-316071.4) | 13693.3 (11160.7-16534.3) | 445170.5 (362867.4-537166.5) | 13719.4 (11182.9-16554.5) | 0.02 (0.01-0.02) |
| United States Virgin Islands | 4110.3 (3344.3-4941) | 10374.6 (8441.2-12471.6) | 3155.9 (2566.6-3783) | 10409.5 (8465.7-12478) | 0.01 (0.01-0.01) |
| Pakistan | 4914312.4 (4006078.5-5919342.9) | 12001.6 (9783.6-14456.1) | 10935711.5 (8935761.3-13044297.2) | 11956 (9769.4-14261.3) | -0.02 (-0.02--0.01) |
| Guam | 6261.7 (5058.7-7551.6) | 9877.6 (7979.9-11912.2) | 5972.3 (4839.7-7223) | 9863.8 (7993.2-11929.4) | -0.03 (-0.04--0.01) |
| Mauritania | 84866.6 (67836.5-102353.2) | 11043.4 (8827.4-13318.9) | 177118.4 (141537.3-213953.3) | 11023 (8808.6-13315.4) | -0.01 (-0.01-0) |
| Cambodia | 408624.3 (328575.9-500798.2) | 10539.6 (8474.9-12917) | 755994.2 (602005.1-920086) | 10631 (8465.6-12938.5) | 0.01 (0-0.03) |
| Singapore | 181878.2 (148579.4-212113.9) | 12050.8 (9844.5-14054.2) | 273376.1 (217482.5-330252.1) | 12845.7 (10219.3-15518.3) | 0.23 (0.16-0.3) |
| Nepal | 813249.3 (652345.4-983168.2) | 11136 (8932.7-13462.8) | 1456455.8 (1177670.9-1769568.3) | 11139 (9006.8-13533.7) | 0 (0-0) |
| South Sudan | 212932.6 (171377-259176) | 9120.1 (7340.3-11100.8) | 309866.1 (250059.6-375158.8) | 9063.6 (7314.2-10973.4) | -0.01 (-0.02-0) |
| Slovakia | 268020.4 (218152-322589.7) | 13090.4 (10654.8-15755.6) | 234922.1 (187980.8-282248.9) | 13152 (10524-15801.6) | 0.03 (0.02-0.03) |
| Mongolia | 118764.6 (95979.2-143513.2) | 13449.4 (10869.1-16252) | 184311.9 (148182.8-221456.5) | 13603.6 (10937-16345.2) | 0.03 (0.03-0.04) |
| Italy | 3199420.6 (2600824.6-3834415.9) | 14985.2 (12181.5-17959.3) | 2474639.1 (2007443-2981072.5) | 15367.2 (12466-18512.1) | 0.08 (0.06-0.1) |
| Kiribati | 3005.3 (2433.1-3636.4) | 9843.8 (7969.6-11911.1) | 4823.1 (3910-5837.9) | 9887 (8015.2-11967.3) | -0.01 (-0.02-0.01) |
| Ireland | 187776.6 (153374.8-227115.7) | 13687.8 (11180.2-16555.4) | 221852.7 (179551.1-267370.7) | 13796.7 (11166-16627.4) | 0.04 (0.04-0.05) |
| Andorra | 3450.4 (2776.3-4186.6) | 13797.2 (11101.6-16740.8) | 3509.7 (2838.6-4227.2) | 13837.5 (11191.7-16666.6) | 0.01 (0-0.02) |
| Kenya | 852825 (695590.4-1020380.8) | 9742.3 (7946.1-11656.4) | 2127206.4 (1738713.6-2546658.9) | 9817.1 (8024.2-11752.9) | 0.03 (0.03-0.04) |
| Mali | 328692.4 (262485-396176.4) | 11042.5 (8818.2-13309.6) | 894964.3 (714662.3-1086109.8) | 10948.6 (8742.8-13287) | -0.03 (-0.03--0.02) |
| Morocco | 1025606.2 (829301.1-1245483.9) | 9857 (7970.4-11970.3) | 1444794.9 (1168939-1746617) | 9882.8 (7995.8-11947.3) | 0.01 (0.01-0.01) |
| Romania | 1135389.1 (925482.2-1362176.4) | 13064.7 (10649.3-15674.3) | 736905.3 (594351.5-884331.8) | 13117.8 (10580.2-15742.2) | 0.02 (0.02-0.03) |
| Zimbabwe | 414803.3 (335554.4-507875.5) | 10474.1 (8473-12824.3) | 656586.8 (529963.8-801396) | 10586.9 (8545.2-12921.8) | 0.05 (0.05-0.06) |
| Eswatini | 31564.4 (25563.6-38621.9) | 10473 (8482-12814.7) | 53737.2 (43345.2-65506.9) | 10642.5 (8584.4-12973.4) | 0.05 (0.04-0.06) |
| United States of America | 15801294.6 (12684467.7-18917453.6) | 15502.6 (12444.7-18559.9) | 16821200 (13664564.5-20034736.4) | 15291 (12421.6-18212.3) | -0.07 (-0.09--0.06) |
| Turkmenistan | 207179.7 (168144.3-248971.4) | 13473.5 (10934.9-16191.4) | 275325.7 (222936.1-330159.1) | 13540 (10963.6-16236.6) | 0.01 (0-0.02) |
| Venezuela (Bolivarian Republic of) | 830591 (673121.2-1002714.8) | 10364.2 (8399.3-12512) | 1107260.9 (898953.3-1325089.4) | 10410.9 (8452.3-12459) | 0.02 (0.01-0.02) |
| Marshall Islands | 1681.9 (1362.2-2039) | 9780.6 (7921.5-11857.3) | 2353.9 (1901.1-2847) | 9897.9 (7993.9-11971.4) | 0.06 (0.05-0.07) |
| Trinidad and Tobago | 52075.7 (42179.8-62539.1) | 10380.8 (8408.2-12466.6) | 53342.3 (43121.4-63953.6) | 10448.7 (8446.7-12527.3) | 0.03 (0.02-0.04) |
| Taiwan (Province of China) | 734914 (593982.9-901311.7) | 7967 (6439.2-9770.8) | 643177.4 (517854.7-778831.7) | 8100 (6521.8-9808.4) | 0.06 (0.05-0.06) |
| Angola | 416212 (336027-508139.8) | 10578.5 (8540.5-12915) | 1183035.8 (953683-1447287.9) | 10559.1 (8512-12917.6) | 0 (0-0) |
| Palestine | 75253.5 (60670.7-91971.8) | 9829.4 (7924.6-12013.1) | 205961 (166326.2-250762.7) | 9855.7 (7959.1-11999.6) | 0 (0-0.01) |
| Suriname | 16728.7 (13520.2-20203.4) | 10351.6 (8366.2-12501.7) | 22330.6 (18120.8-26801) | 10397.7 (8437.5-12479.3) | 0.01 (0.01-0.01) |
| Saint Lucia | 5813.8 (4708.5-7039.9) | 10339.1 (8373.5-12519.5) | 7034.5 (5705.9-8404) | 10418.1 (8450.5-12446.3) | 0.02 (0.02-0.02) |
| Niger | 306070.8 (244522.7-370329) | 11013.5 (8798.8-13325.7) | 886834.6 (708124.6-1077217.1) | 10913.9 (8714.6-13256.9) | -0.03 (-0.04--0.03) |
| Bahamas | 12255 (9924.5-14759.5) | 10379.8 (8405.8-12501) | 15590.1 (12637.6-18637.3) | 10414.6 (8442.3-12450.2) | 0 (0-0.01) |
| Ethiopia | 1651770.1 (1334416.6-2008054.4) | 8854.4 (7153.2-10764.3) | 3562147.2 (2897234.5-4344070.1) | 8025.2 (6527.2-9786.9) | -0.47 (-0.56--0.38) |
| Micronesia (Federated States of) | 3908.7 (3158.1-4730.4) | 9783.7 (7904.8-11840.5) | 4143.8 (3353.7-5023.7) | 9802.3 (7933.5-11883.9) | 0.01 (0-0.01) |
| Lao People's Democratic Republic | 162737.5 (130830.1-199105.6) | 10528.6 (8464.3-12881.5) | 333628.2 (266600.8-405646.2) | 10597.2 (8468.2-12884.7) | 0.01 (0-0.02) |
| Belarus | 549218.9 (439890.2-664653.5) | 13896.9 (11130.5-16817.7) | 424601.9 (338540.6-511170.5) | 13917.6 (11096.7-16755.2) | 0.01 (0.01-0.02) |
| Malta | 19011.6 (15327-22942.2) | 13783.4 (11112-16633) | 19089.3 (15376.9-23088.9) | 13837.7 (11146.6-16737) | 0.02 (0.01-0.03) |
| Samoa | 6466.2 (5225.3-7859.8) | 9692.7 (7832.5-11781.6) | 8132.3 (6586.4-9857.3) | 9773 (7915.3-11846.2) | 0.02 (0.01-0.03) |
| Brazil | 7689981.4 (6236250.6-9250542.7) | 12254.9 (9938.2-14741.9) | 10572025.3 (8593520.8-12755378.6) | 12275.5 (9978.2-14810.7) | -0.02 (-0.04-0) |
| Dominica | 3014.5 (2439.2-3642.1) | 10334.8 (8362.7-12486.7) | 2678 (2170.9-3222.3) | 10387.1 (8420.3-12498.4) | 0.01 (0.01-0.01) |
| Latvia | 132554.6 (106721.3-159633) | 13893.3 (11185.6-16731.4) | 78091.1 (62146.1-94273.7) | 13910.3 (11070-16792.9) | 0.01 (0.01-0.02) |
| Uzbekistan | 1157692.1 (941901.2-1391616.2) | 13476.7 (10964.7-16199.8) | 1931514.3 (1564498.8-2314815.6) | 13539.7 (10966.9-16226.5) | 0.01 (0-0.02) |
| Philippines | 2974896.6 (2433881.6-3556479.9) | 11458.1 (9374.3-13698.1) | 5311066.6 (4328761.9-6346023) | 11513.8 (9384.3-13757.5) | 0.01 (0.01-0.01) |
| Luxembourg | 20494.8 (16493.7-24744.8) | 13882.8 (11172.4-16761.6) | 29665.1 (23818.9-35695.4) | 13899.1 (11159.9-16724.5) | -0.02 (-0.03--0.02) |
| Mauritius | 53122.9 (42432.2-64586) | 10633.3 (8493.4-12927.8) | 50058.7 (40173.3-60461.6) | 10680.7 (8571.5-12900.3) | 0.02 (0.01-0.02) |
| Paraguay | 176434.1 (142304.7-214205.7) | 11264.3 (9085.4-13675.8) | 334842.8 (269153.5-404378.1) | 11266.5 (9056.3-13606.2) | 0.01 (0-0.01) |
| Benin | 188462.5 (150592.7-227118.1) | 11066.9 (8843.1-13336.9) | 539062.2 (431349.3-650936.4) | 11029.7 (8825.8-13318.7) | -0.02 (-0.02--0.01) |
| Malaysia | 788378 (633615.3-963154.2) | 10638.1 (8549.7-12996.4) | 1466623 (1167040.3-1783026.1) | 10642.2 (8468.3-12938.1) | -0.02 (-0.03--0.01) |
| Ecuador | 328263.4 (266482.1-402224.6) | 7963.6 (6464.8-9757.9) | 607711.5 (492687.3-736348.5) | 8426.9 (6831.9-10210.7) | 0.27 (0.22-0.32) |
| Monaco | 1266.4 (1018.8-1533.4) | 13833.7 (11129.1-16750.4) | 1279.2 (1036.4-1544.7) | 13783.5 (11167.5-16643.4) | -0.03 (-0.04--0.02) |
| Qatar | 23845.2 (18815.8-29053.7) | 10089.2 (7961.2-12293) | 173516.3 (135611.7-213239.8) | 10100.7 (7894.2-12413.1) | 0.01 (0-0.02) |
| El Salvador | 215513.3 (174403.8-261332.4) | 10331.1 (8360.4-12527.5) | 267637.4 (216676.7-322064) | 10387.3 (8409.4-12499.6) | 0.01 (0.01-0.02) |
| Armenia | 194841.4 (157551.9-234141.4) | 13552.2 (10958.6-16285.8) | 151833.7 (121574.6-182728.8) | 13618.8 (10904.7-16390) | 0.01 (0-0.03) |
| Iran (Islamic Republic of) | 2483683.2 (2009718.8-3001737.4) | 10992.9 (8895.1-13285.9) | 4075190.6 (3281468.1-4927032.7) | 11444.6 (9215.6-13836.9) | 0.13 (0.11-0.15) |
| Cuba | 504360.5 (407507.5-608088.1) | 10369.3 (8378-12501.8) | 377955.7 (305729.4-455423.4) | 10421.6 (8430.1-12557.7) | 0 (-0.01-0.01) |
| Nigeria | 4058141.4 (3315223.8-4910759) | 11914.8 (9733.6-14418.1) | 9959044.1 (8107928.2-12052440.8) | 11876.1 (9668.7-14372.5) | -0.03 (-0.04--0.02) |
| Myanmar | 1786787.8 (1431033.2-2179328.4) | 10550.1 (8449.5-12867.8) | 2350723 (1880124.8-2852426.4) | 10637.6 (8508-12907.9) | 0.03 (0.03-0.03) |
| Malawi | 330376.8 (266953.3-402220.7) | 9135.8 (7382-11122.5) | 693960.3 (560636-837944.4) | 9104.5 (7355.4-10993.6) | 0 (-0.01-0) |
| Oman | 80065.6 (64511-97241.8) | 9973.2 (8035.7-12112.8) | 258379.6 (203486.4-316985.9) | 10077.8 (7936.7-12363.6) | 0.03 (0.01-0.05) |
| Congo | 99710.7 (80562.7-121872.9) | 10511.9 (8493.2-12848.3) | 224674.2 (181500.7-274130.2) | 10669.5 (8619.2-13018.1) | 0.07 (0.06-0.07) |
| Madagascar | 413429 (333928.6-501175.1) | 9137.3 (7380.3-11076.6) | 1000674.2 (809211.5-1214129.8) | 9160.2 (7407.5-11114.2) | 0.01 (0.01-0.01) |
| Papua New Guinea | 161999.5 (130978.6-196609.4) | 9788.6 (7914.2-11879.8) | 403303.5 (327643.2-488441.3) | 9855 (8006.2-11935.4) | 0.02 (0.01-0.02) |
| Indonesia | 8968214.7 (7337532.9-10720662.7) | 11488.8 (9399.8-13733.8) | 12273790.6 (9984914.7-14649147.1) | 11587.4 (9426.5-13829.9) | 0.04 (0.03-0.04) |
| New Zealand | 178491 (144626.9-210993.4) | 12914.8 (10464.5-15266.5) | 182393.1 (148101.5-215061.5) | 12932.6 (10501.1-15248.9) | -0.01 (-0.04-0.01) |
| Bolivia (Plurinational State of) | 213784.6 (173851.7-260555.6) | 8677.7 (7056.8-10576.2) | 421056.9 (343059.2-511247.2) | 8708.8 (7095.6-10574.2) | 0.01 (0.01-0.02) |
| Sao Tome and Principe | 4688.6 (3741.6-5704.8) | 10920.1 (8714.6-13287.2) | 9630.6 (7699-11590.7) | 11090.3 (8866-13347.5) | 0.08 (0.07-0.09) |
| Antigua and Barbuda | 2680.5 (2169.7-3223.8) | 10391.4 (8411.2-12497.6) | 3605.7 (2925.9-4314.1) | 10418.7 (8454.4-12465.6) | 0 (0-0.01) |
| Belgium | 512551.9 (414359.6-618630.5) | 13778.4 (11138.8-16630) | 483757.9 (392041.3-582884.3) | 13795.7 (11180.1-16622.5) | -0.01 (-0.01-0) |
| Nauru | 398.6 (323.5-483.3) | 9856.4 (7997.5-11949.6) | 449.2 (364.1-544.2) | 9845.7 (7979-11926.6) | 0 (0-0) |
| Burkina Faso | 350429.7 (280333.4-424436.1) | 10974.6 (8779.3-13292.3) | 941399.2 (752263.2-1135544.8) | 11028.4 (8812.7-13302.8) | 0.03 (0.02-0.03) |
| Bosnia and Herzegovina | 252521.1 (205250.9-303741.9) | 13074.1 (10626.7-15726.1) | 136414.3 (110136.2-163663.1) | 13108.1 (10583.1-15726.5) | 0.01 (0-0.01) |
| Bulgaria | 389454.1 (317452.8-467494.9) | 13085.5 (10666.3-15707.6) | 259622.7 (207450.9-312108.4) | 13145.5 (10503.9-15803) | 0.02 (0.02-0.03) |
| Democratic Republic of the Congo | 1507576.1 (1215461.9-1843060.6) | 10545.3 (8502-12892) | 3693313.6 (2978707.5-4514820.1) | 10556.9 (8514.3-12905.1) | 0 (-0.01-0) |
| Norway | 242407.8 (196160.5-292091.6) | 15153 (12262.1-18258.8) | 268479.2 (215740-323874.1) | 15255.7 (12258.9-18403.4) | 0.01 (-0.01-0.02) |
| Algeria | 997675.4 (804190.6-1214917.8) | 9852.3 (7941.6-11997.6) | 1698594.9 (1367825.2-2057625.9) | 9903.4 (7974.9-11996.6) | 0.02 (0.02-0.02) |
| Slovenia | 100411.4 (81391.5-120826.3) | 13103.8 (10621.6-15767.9) | 78056.5 (62634.3-93735.9) | 13139.2 (10543.2-15778.5) | 0.02 (0.01-0.02) |
| Portugal | 519117.9 (423051.7-626378.2) | 13712.6 (11175-16545.9) | 419794.7 (340024.8-508150.2) | 13794.5 (11173.3-16697.9) | 0.03 (0.03-0.04) |
| Chile | 679872.4 (557804.4-813045.4) | 11871.6 (9740.1-14197) | 822742.8 (662110.4-986057.5) | 12090.1 (9729.6-14490) | 0.06 (0.05-0.07) |
| Solomon Islands | 12489.7 (10099.2-15186.1) | 9719.8 (7859.5-11818.2) | 25727.7 (20794.4-31169.4) | 9834.5 (7948.7-11914.6) | 0.06 (0.05-0.06) |
| Cabo Verde | 14330.8 (11437.4-17387.2) | 10973.4 (8757.9-13313.8) | 28075.2 (22265.4-34038.9) | 11287.2 (8951.5-13684.8) | 0.08 (0.05-0.1) |
| Czechia | 485642.4 (396106.6-581748.3) | 13069.6 (10660-15656) | 400334.1 (319704.7-480684.8) | 13148.9 (10500.6-15788) | 0.03 (0.03-0.04) |
| Netherlands | 828507.2 (659265.2-1010117.5) | 13740.6 (10933.8-16752.6) | 723242.3 (576996.7-880902.8) | 13739.6 (10961.3-16734.7) | -0.02 (-0.03--0.01) |
| Senegal | 303109.8 (242083.9-367281.7) | 10990.7 (8777.9-13317.6) | 676427.5 (540551.3-815995.1) | 11035.4 (8818.7-13312.4) | 0.02 (0.02-0.02) |
| Northern Mariana Islands | 2339.5 (1869.5-2831.5) | 9985.9 (7979.8-12085.8) | 1347.1 (1089.9-1639.2) | 9675.8 (7828.6-11774) | -0.12 (-0.17--0.06) |
| Tunisia | 339625.4 (274446-413734.7) | 9855.8 (7964.3-12006.4) | 438604.6 (353186.3-530614.2) | 9897.2 (7969.7-11973.4) | 0.02 (0.01-0.02) |
| Hungary | 484068.1 (392626.2-580301.9) | 13091.7 (10618.6-15694.3) | 373967.5 (302167.2-447974.9) | 13123.6 (10603.9-15720.8) | 0.02 (0.02-0.03) |
| Sierra Leone | 151480.5 (120941.1-182369.6) | 11104.7 (8866-13369.2) | 386669.2 (308542.6-466694.7) | 11059.3 (8824.8-13348.2) | -0.02 (-0.03--0.02) |
| Guyana | 35232.4 (28518.5-42614.1) | 10356.1 (8382.6-12525.8) | 33587.5 (27148.4-40452.7) | 10385.2 (8394.3-12507.9) | -0.01 (-0.01-0) |
| Central African Republic | 110964.8 (89262-135724.9) | 10568.9 (8501.8-12927.2) | 222471.5 (179502.7-271529.2) | 10543.2 (8506.9-12868.1) | -0.01 (-0.01--0.01) |
| Germany | 4103015 (3314031.1-4960222.8) | 13812.8 (11156.7-16698.6) | 3536412.8 (2836392.3-4311233.1) | 13877.3 (11130.3-16917.8) | 0.01 (-0.01-0.02) |
| Kuwait | 87225.4 (69699-107107.9) | 10001.7 (7992.1-12281.6) | 208090.2 (164172.5-253666.8) | 9966.7 (7863.2-12149.7) | -0.02 (-0.02--0.01) |
| Mozambique | 417639.3 (337315.7-503115) | 9128.7 (7373-10997) | 1022431.6 (826059.8-1239846.2) | 9109.3 (7359.7-11046.3) | 0 (-0.01-0.01) |
| Grenada | 3448.2 (2796.1-4178.4) | 10345 (8388.4-12535.5) | 4240.3 (3420.5-5096.3) | 10389.2 (8380.7-12486.6) | 0.01 (0.01-0.02) |
| Saudi Arabia | 649650.4 (523674.4-791116.8) | 9688.4 (7809.7-11798.1) | 1769821.5 (1407104.4-2133635) | 9660 (7680.2-11645.7) | -0.02 (-0.04-0) |
| Colombia | 1459840.6 (1180509.6-1760037.6) | 10373.5 (8388.6-12506.7) | 1990376.5 (1612264.5-2380887.8) | 10405.8 (8429-12447.5) | 0 (0-0.01) |
| Russian Federation | 8641030.6 (6962138-10264808.1) | 14848.5 (11963.5-17638.7) | 7195297.2 (5758140.6-8587212.4) | 14984.1 (11991.3-17882.8) | 0.01 (-0.01-0.03) |
| Cameroon | 418669.5 (334719.7-505684) | 11022.4 (8812.2-13313.2) | 1337264.2 (1068674.4-1614286.2) | 11067.1 (8844.2-13359.7) | 0.02 (0.01-0.03) |
| Syrian Arab Republic | 473661.6 (381746.6-578995.6) | 9835 (7926.5-12022.1) | 540738.6 (438294.5-660615.2) | 9816 (7956.4-11992.1) | 0 (0-0.01) |
| Lithuania | 194066.7 (155768.8-234250.1) | 13929.9 (11180.9-16814.2) | 117382 (93927.2-142994.6) | 13977.6 (11184.6-17027.5) | 0.01 (0.01-0.01) |
| Albania | 185133.4 (150105.4-222550.8) | 13053.7 (10583.9-15692) | 126818.3 (102831.8-152350.2) | 13070 (10598-15701.4) | -0.01 (-0.01-0) |
| Chad | 230973.9 (184937.2-279076.2) | 11005.8 (8812.2-13297.9) | 632958.2 (506121.3-769102) | 10932.7 (8741.9-13284.2) | -0.02 (-0.02--0.01) |
| Austria | 412855.6 (333442-495041.7) | 13758.4 (11112-16497.3) | 388757.3 (313391.4-469641.7) | 13818.3 (11139.4-16693.3) | 0 (-0.01-0.01) |
| Rwanda | 250253.9 (201570.8-303175.9) | 9160.1 (7378.1-11097.2) | 497582.4 (401578.8-602435.5) | 9190.2 (7417-11126.8) | 0.02 (0.01-0.04) |
| Belize | 7563.2 (6114-9194.6) | 10324.7 (8346.4-12551.7) | 18564.1 (15049.2-22414.8) | 10366 (8403.3-12516.2) | 0.01 (0.01-0.01) |
| Finland | 250609.3 (202716-301800.7) | 13805.6 (11167.3-16625.6) | 230696.1 (187069.6-277937.1) | 13789.5 (11181.8-16613.2) | -0.01 (-0.01-0) |
| Egypt | 2211246.2 (1784391.8-2698614.5) | 10091.5 (8143.5-12315.7) | 4150548.5 (3313894.5-5070995.1) | 10119.3 (8079.5-12363.4) | 0.01 (0.01-0.01) |
| Vanuatu | 5751.7 (4648.8-6977.8) | 9814.7 (7932.7-11907) | 11629.9 (9428.2-14098.1) | 9852.3 (7987.1-11943.3) | 0.01 (0.01-0.01) |
| Thailand | 2742858.1 (2195171.2-3332803.7) | 10589.2 (8474.8-12866.8) | 2606256.5 (2081477-3155128.5) | 10740.3 (8577.7-13002.1) | 0.04 (0.04-0.05) |
| Togo | 150778.1 (120540-182453.6) | 10998.8 (8793-13309.5) | 354043 (282755.6-426695.9) | 11106.7 (8870.4-13386) | 0.03 (0.03-0.04) |
| Spain | 2034618.5 (1650773.7-2424825.8) | 13719.1 (11130.9-16350.2) | 1798862.6 (1446689.7-2146196.8) | 13861.1 (11147.4-16537.4) | 0.03 (0.03-0.04) |
| Peru | 772037.1 (620521.6-939393) | 8705.9 (6997.4-10593.2) | 1218983.7 (991477.9-1485410.3) | 8941.7 (7272.9-10896) | 0.11 (0.08-0.13) |
| Niue | 79 (63.9-95.6) | 9812.9 (7943.5-11877.6) | 55.2 (44.7-66.8) | 9882.9 (8000.8-11956.9) | 0.03 (0.03-0.03) |
| Turkey | 2347143.3 (1903278.4-2842224.5) | 9493.5 (7698.2-11495.9) | 3251114 (2638558.5-3904985.4) | 9550.6 (7751.1-11471.5) | -0.22 (-0.34--0.11) |
| Tonga | 3577.6 (2889.5-4357.2) | 9706.2 (7839.3-11821.3) | 3785 (3073.1-4585.6) | 9817.7 (7971.1-11894.3) | 0.04 (0.04-0.05) |
| Gambia | 41759.3 (33352.8-50457.5) | 11048.1 (8824-13349.4) | 103924.7 (83014.3-125380.8) | 11032.9 (8813-13310.7) | 0 (0-0) |
| Sweden | 440604.8 (355414.6-526154.9) | 15011.5 (12109-17926.2) | 481515.5 (388566.5-578997.5) | 15078.3 (12167.7-18130.9) | 0 (0-0.01) |
| Ukraine | 2864510 (2320029.7-3414335.3) | 15087.3 (12219.5-17983.2) | 2183752 (1756967.6-2619317.6) | 15119.3 (12164.4-18135) | 0.01 (0.01-0.02) |
| Estonia | 78879.5 (63508.4-94933.7) | 13887.6 (11181.4-16714.2) | 55984 (44646.6-67583.7) | 13915.4 (11097.4-16798.6) | 0.01 (0.01-0.02) |
| Cyprus | 42259.2 (34279.7-50968) | 13739.9 (11145.5-16571.4) | 69510.1 (55475.8-84304.6) | 13887.1 (11083.3-16842.8) | 0.03 (0.02-0.04) |
| Saint Kitts and Nevis | 1798.8 (1457.9-2173.3) | 10363.6 (8399.8-12521.4) | 2362.7 (1914.1-2833.3) | 10427 (8447.4-12503.9) | 0.02 (0.02-0.02) |
| Palau | 689.8 (558.5-835.9) | 9861.7 (7984.7-11951.3) | 607.5 (490.4-731.4) | 9951.9 (8034.2-11982.3) | 0 (-0.01-0.02) |
| Azerbaijan | 429662.3 (348689.2-515383.5) | 13509 (10963.2-16204.2) | 580458.3 (467390.8-697826.7) | 13600.6 (10951.4-16350.7) | 0.01 (0-0.02) |
| United Arab Emirates | 96672.4 (76623-117354.9) | 10091.4 (7998.5-12250.4) | 463431.7 (352742-581005.9) | 10336.6 (7867.8-12959.1) | 0.07 (0.05-0.09) |
| Equatorial Guinea | 15811.9 (12787.4-19258) | 10540 (8523.9-12837.1) | 68487.5 (55207.7-83758.6) | 10523.2 (8482.8-12869.7) | -0.01 (-0.02-0) |
| Maldives | 8496.1 (6822.7-10465.2) | 10452.4 (8393.7-12874.9) | 27078.2 (21106.6-32816.1) | 10741.5 (8372.7-13017.7) | 0.07 (0.05-0.09) |
| Canada | 1561246.6 (1256188.1-1880458.4) | 14042.2 (11298.5-16913.3) | 1610558.1 (1299520.7-1933783.7) | 14030.1 (11320.5-16845.8) | -0.01 (-0.02--0.01) |
| Montenegro | 32766.9 (26654.9-39380.3) | 13070.2 (10632.2-15708.2) | 27611.7 (22393.8-33192.1) | 13095.3 (10620.6-15741.9) | 0.01 (0.01-0.01) |
| C么te d'Ivoire | 524082.2 (419514.1-634277.6) | 11079.6 (8868.9-13409.2) | 1199411.4 (956773.7-1444117.9) | 11139.6 (8886.1-13412.3) | 0.03 (0.03-0.04) |
| United Republic of Tanzania | 881115.7 (710567-1067024.2) | 9102.5 (7340.6-11023.1) | 2018593.5 (1631943.7-2441243.2) | 9141.7 (7390.7-11055.8) | 0.02 (0.02-0.02) |
| Somalia | 230751.7 (186033.7-278900) | 9139.4 (7368.2-11046.4) | 715427.6 (578087.7-868286.1) | 9122.2 (7371-11071.3) | 0.04 (0.02-0.06) |
| Croatia | 237772.5 (192621-286273.9) | 13107 (10618.1-15780.6) | 169253.7 (137560.3-202212.1) | 13132.6 (10673.5-15689.9) | -0.02 (-0.04-0.01) |
| Bahrain | 25731.2 (20495.9-31311.6) | 10020.2 (7981.5-12193.2) | 62580.3 (49860.2-75740.5) | 10120.6 (8063.4-12248.8) | 0.05 (0.03-0.07) |
| Puerto Rico | 146749.9 (119247.5-176542.3) | 10377.5 (8432.7-12484.3) | 116995.1 (95063.6-139796.8) | 10413.7 (8461.6-12443.3) | 0.01 (0.01-0.01) |
| Jordan | 151717 (122466.4-186124.3) | 9821.3 (7927.8-12048.6) | 495344.6 (401517.3-598119.2) | 9884.7 (8012.3-11935.6) | 0.02 (0.02-0.02) |
| Ghana | 635489.6 (506950.3-766644.4) | 11053.4 (8817.7-13334.7) | 1516351.9 (1209657.5-1828878.2) | 11138.7 (8885.8-13434.4) | 0.02 (0.02-0.03) |
| Greece | 516158 (420499-622067.2) | 13733.4 (11188.2-16551.4) | 402048.9 (324891.7-483649.5) | 13824.6 (11171.5-16630.5) | 0.03 (0.03-0.04) |
| Yemen | 453565.2 (365897.3-549813.5) | 9835.5 (7934.4-11922.6) | 1282335.6 (1042473.5-1548318.7) | 9857.1 (8013.3-11901.7) | 0.02 (0.01-0.02) |
| Guatemala | 300009.6 (242701.9-363170.7) | 10339.4 (8364.3-12516.1) | 821116.8 (664545.4-989365.5) | 10368.4 (8391.4-12492.9) | 0.01 (0-0.01) |
| Seychelles | 3293.7 (2632.2-4031.9) | 10544.8 (8426.8-12908.1) | 4127.4 (3275.7-4988.6) | 10694.3 (8487.5-12925.9) | 0.04 (0.03-0.04) |
| Guinea-Bissau | 41316.1 (33051.2-49957.3) | 11005.8 (8804.2-13307.6) | 89101.9 (71130.4-107681.4) | 11087.9 (8851.5-13400) | 0.03 (0.02-0.04) |
| Costa Rica | 133295.9 (107886.1-160480.8) | 10380.7 (8401.8-12497.7) | 199802.5 (161700.9-239906.5) | 10429.6 (8440.8-12523.1) | 0.01 (0.01-0.02) |
| Zambia | 283878.8 (227367.5-347328.7) | 9358 (7495.2-11449.7) | 704491.6 (569409.9-858448.3) | 9284.5 (7504.2-11313.5) | -0.04 (-0.06--0.02) |
| United Kingdom | 3098776.6 (2538318.2-3724284.2) | 14858.5 (12171.1-17857.8) | 3266989.8 (2664796.7-3919496.5) | 14938 (12184.5-17921.5) | 0 (-0.01-0.01) |
| Denmark | 261066.7 (208820.5-316457.9) | 13683.6 (10945.2-16586.9) | 247754.4 (199033.6-300706.7) | 13745 (11042-16682.7) | 0.01 (-0.01-0.02) |
| Switzerland | 348462.9 (283302.3-416098.4) | 13224.4 (10751.5-15791.2) | 380617.6 (305141.2-454213.7) | 13717.3 (10997.2-16369.7) | 0.11 (0.09-0.14) |
| Republic of Moldova | 242086.9 (194767-291759.3) | 13882.2 (11168.7-16730.6) | 182625.4 (145672-220145) | 13917.4 (11101.3-16776.6) | 0.02 (0.01-0.02) |
| France | 3020906.4 (2419377.5-3616213.3) | 13735.1 (11000.2-16441.8) | 2732101.3 (2196328.3-3263413.8) | 13741.4 (11046.7-16413.7) | -0.02 (-0.03--0.02) |
| Gabon | 40394.6 (32555.1-49306.4) | 10583.6 (8529.6-12918.6) | 79248.5 (64032.5-96457.7) | 10650.5 (8605.5-12963.3) | 0.02 (0.01-0.02) |
| Djibouti | 18351.5 (14751.4-22367.5) | 9144.8 (7350.8-11146) | 47004 (37676.8-57138.3) | 9312.2 (7464.4-11320) | 0.07 (0.07-0.08) |
| Brunei Darussalam | 15717.3 (12647.8-19014.8) | 12747.4 (10257.9-15421.9) | 25765.5 (20711.7-31137.4) | 12773.8 (10268.3-15437.1) | 0 (0-0) |
| American Samoa | 1989.1 (1608.9-2410) | 9825.2 (7947.4-11904.1) | 2059.4 (1671.2-2494.8) | 9769.8 (7927.8-11835) | -0.03 (-0.04--0.01) |
| Sri Lanka | 784015.5 (628254.8-952906.9) | 10598.2 (8492.7-12881.3) | 857953.3 (688059.9-1037435.3) | 10675.3 (8561.4-12908.6) | 0.03 (0.03-0.04) |
| Burundi | 190103.8 (153141.5-230988.6) | 9177.1 (7392.7-11150.7) | 423363.2 (341296-512626.8) | 9161.7 (7385.7-11093.4) | -0.01 (-0.02-0.01) |
| Iraq | 647120.3 (521929.4-786963) | 9842.3 (7938.2-11969.2) | 1842394.2 (1486199.3-2239920.5) | 9866.9 (7959.3-11995.9) | 0.01 (0-0.01) |
| Dominican Republic | 318259.4 (257477.6-384803.1) | 10354.1 (8376.7-12519.1) | 467663.6 (378445.9-561086.4) | 10398.5 (8414.7-12475.8) | 0.01 (0.01-0.01) |
| Guinea | 239108.4 (191261.5-287823.3) | 11073.5 (8857.6-13329.6) | 532249.3 (424577.1-641866.4) | 11035.3 (8802.9-13308) | -0.01 (-0.02-0) |
| Afghanistan | 378799 (303847.2-466828.4) | 9782.8 (7847.1-12056.3) | 1482353.9 (1194303.2-1813392.4) | 9836.7 (7925.2-12033.4) | 0.01 (0-0.02) |
| North Macedonia | 106203 (86445.7-127530.4) | 13072.4 (10640.5-15697.5) | 101421.2 (81684.7-121904.8) | 13127 (10572.5-15778.2) | 0.01 (0.01-0.02) |
| Honduras | 178128.8 (144277.3-215954.1) | 10322.8 (8361.1-12514.8) | 437888 (355130.9-528715.9) | 10362.4 (8404-12511.8) | 0.02 (0.01-0.02) |
| Bangladesh | 4736323.8 (3813678.5-5729477.5) | 11096.8 (8935.1-13423.6) | 7556005.5 (6103719.1-9099702.9) | 11143.7 (9001.9-13420.4) | 0.01 (0.01-0.01) |
| Tokelau | 56.6 (46.1-68.5) | 9818.9 (7991.7-11881.6) | 47.7 (38.5-57.9) | 9847.3 (7937.9-11933.8) | 0.01 (0-0.02) |
| Lesotho | 72598.6 (58563.2-88760.3) | 10523.2 (8488.8-12865.9) | 101440 (81765.3-123508.7) | 10678 (8606.9-13001) | 0.07 (0.05-0.08) |
| Uganda | 583775.6 (470089.1-709244.4) | 9096.5 (7325-11051.6) | 1468552.7 (1183485.3-1782012.3) | 9089.3 (7324.9-11029.4) | 0 (-0.01-0) |
| Argentina | 1468748.2 (1185992.3-1756155) | 12017 (9703.6-14368.5) | 2101411.7 (1696256.5-2512931.1) | 12064.4 (9738.3-14426.9) | 0.02 (0.02-0.03) |
| Tuvalu | 357.6 (289.2-433) | 9924.2 (8026-12016.7) | 470.5 (381.2-570.3) | 9824.9 (7958.6-11907.9) | -0.05 (-0.06--0.04) |
| Barbados | 11364.9 (9211.2-13626) | 10407.9 (8435.6-12478.6) | 10304.6 (8374.9-12327) | 10417.7 (8466.9-12462.3) | 0 (0-0) |
| San Marino | 1242.5 (1012.6-1500) | 13710.5 (11173.6-16552.5) | 1417.4 (1151.5-1705.8) | 13773.9 (11190.2-16576.2) | 0.01 (0-0.02) |
| Comoros | 15691.5 (12660.8-19018.1) | 9094.6 (7338.1-11022.7) | 27319.8 (22064-33127.1) | 9221.4 (7447.3-11181.5) | 0.05 (0.04-0.05) |
| Botswana | 54072.7 (43662.7-66182.6) | 10508.5 (8485.4-12861.9) | 113628.3 (91160.3-137959.3) | 10779.7 (8648.2-13088) | 0.09 (0.08-0.11) |
| Sudan | 750640.9 (604947.4-911013.7) | 9842.8 (7932.4-11945.7) | 1697373.2 (1373829.5-2060702.9) | 9835.5 (7960.7-11940.8) | 0 (-0.01-0) |

Abbreviations: EAPC, estimated annual percentage change; SDI, Sociodemographic Index; UI, uncertainty interval.
