# Supplementary material for: Global, regional, and national epidemiology of migraine and tension-type headache in youths and young adults aged 15–39 years from 1990 to 2019: findings from the global burden of disease study 2019
Source: J Headache Pain. 2023 Sep 18;24(1):126. doi: 10.1186/s10194-023-01659-1 (PMC10506184; doi:10.1186/s10194-023-01659-1)
Supplement: Supplementary file 15 — Additional file 15: Table S8. DALYs of Migraine Between 1990 and 2019 in 15 to 39 years at the 204 Countries Level. [file 10194_2023_1659_MOESM15_ESM.docx]

| **TableS8 DALYs of Migraine Between 1990 and 2019 in 15 to 39 years at the Countries Level** | | | | | |
| --- | --- | --- | --- | --- | --- |
|  | 1990 | | 2019 | |  |
| Location | Number_95%UI | ASR | Number_95%UI | ASR | EAPC_95%CI |
| Mexico | 249698.2 (28171.3-587962.8) | 700.5 (79-1649.6) | 358849.2 (42526-839950.7) | 715.1 (84.7-1673.9) | 0.08 (0.07-0.09) |
| Haiti | 16760.4 (1794.2-40319.5) | 689.7 (73.8-1659.1) | 36772.2 (4042.7-88163.7) | 691.9 (76.1-1658.8) | 0.02 (0.02-0.03) |
| Viet Nam | 227533.8 (20202.5-571830) | 797.7 (70.8-2004.8) | 321234.9 (29947-803404.4) | 814.3 (75.9-2036.6) | 0.05 (0.03-0.07) |
| Bhutan | 1813.9 (166-4403.7) | 697.6 (63.8-1693.6) | 2504.1 (231.4-6018.4) | 713.1 (65.9-1713.8) | 0.06 (0.05-0.07) |
| Jamaica | 6817.1 (742.6-16568.3) | 693.8 (75.6-1686.2) | 8263.2 (918.9-19665) | 692.4 (77-1647.7) | -0.02 (-0.02--0.01) |
| Nicaragua | 10208 (1095.3-24800.6) | 693.2 (74.4-1684.2) | 19465.9 (2200-46008.4) | 693.9 (78.4-1640.1) | 0.01 (0-0.01) |
| Kyrgyzstan | 12071.1 (1672.7-28348.8) | 669.1 (92.7-1571.3) | 18034.3 (2514.3-42053.2) | 677.2 (94.4-1579.2) | 0.01 (-0.01-0.04) |
| Georgia | 14552.7 (2070-33565.9) | 683.4 (97.2-1576.3) | 8093.7 (1155.9-18825) | 684 (97.7-1590.9) | -0.04 (-0.06--0.02) |
| Lebanon | 10066.4 (1491.7-23938) | 836 (123.9-1988) | 17508.5 (2804.3-40894.6) | 852.9 (136.6-1992) | 0.06 (0.05-0.07) |
| Kazakhstan | 45824.7 (6486.8-106865.5) | 674.4 (95.5-1572.7) | 47605.2 (6884.2-110676.8) | 685.1 (99.1-1592.8) | 0.04 (0.01-0.07) |
| Namibia | 3497.2 (461.4-8152.2) | 625.4 (82.5-1457.9) | 6478.7 (872.8-15004.6) | 634.5 (85.5-1469.6) | 0.05 (0.03-0.06) |
| Republic of Korea | 136080.3 (17331.9-318340.1) | 646 (82.3-1511.2) | 104163.1 (15884.7-241506.8) | 601 (91.6-1393.4) | -0.36 (-0.41--0.31) |
| Timor-Leste | 2485.1 (224.7-6229) | 781.1 (70.6-1957.9) | 4202.9 (366.1-10629.4) | 781.8 (68.1-1977.4) | 0.01 (-0.01-0.03) |
| China | 2961555.8 (389143.6-6786188.5) | 539 (70.8-1235) | 3001293.8 (376020.8-6990363.3) | 603 (75.5-1404.5) | 0.31 (0.28-0.34) |
| Eritrea | 5202.8 (911.3-11689.7) | 449.4 (78.7-1009.7) | 13181.8 (2337.3-29681.3) | 458.5 (81.3-1032.4) | 0.08 (0.07-0.09) |
| Iceland | 973.8 (104.7-2345.3) | 937.4 (100.8-2257.8) | 1125 (119-2682.2) | 938.1 (99.2-2236.5) | -0.01 (-0.03-0) |
| Panama | 6951.6 (754.1-16613.4) | 687.9 (74.6-1644) | 11025.1 (1253.3-26061.1) | 689.8 (78.4-1630.5) | 0.01 (0.01-0.02) |
| Serbia | 23306.6 (3985.9-52806.3) | 677.7 (115.9-1535.6) | 19232.7 (3313.5-43735.8) | 681.1 (117.3-1548.9) | 0.05 (0.04-0.06) |
| India | 2463644.1 (236685.2-5773916) | 722.9 (69.5-1694.3) | 4347052.5 (429668.3-10213067.8) | 729.2 (72.1-1713.1) | -0.06 (-0.09--0.03) |
| Libya | 13346 (1950-31877.2) | 814.5 (119-1945.5) | 25399.1 (3938.1-59282.6) | 839.3 (130.1-1959) | 0.09 (0.08-0.1) |
| South Africa | 104795.4 (14397.1-239151.6) | 659.1 (90.5-1504) | 159276.9 (23106.2-362783.1) | 665 (96.5-1514.8) | 0.01 (-0.01-0.04) |
| Democratic People's Republic of Korea | 48372.1 (5412.6-113038.1) | 586 (65.6-1369.4) | 58492.4 (6753.3-135371.7) | 582.2 (67.2-1347.4) | -0.1 (-0.13--0.07) |
| Uruguay | 6880.9 (992.4-16075.2) | 605.8 (87.4-1415.3) | 7358.4 (1063.8-17013) | 609.3 (88.1-1408.7) | 0.03 (0.02-0.03) |
| Japan | 251929.2 (40938-581303) | 561.7 (91.3-1296.1) | 191561 (31802.3-450712.3) | 582.6 (96.7-1370.8) | 0.18 (0.15-0.21) |
| Poland | 103090.3 (17638.9-237515.2) | 713 (122-1642.6) | 92010.6 (16266-212750.8) | 728 (128.7-1683.3) | 0.11 (0.08-0.15) |
| Saint Vincent and the Grenadines | 313.2 (34.9-764) | 682.2 (76.1-1664.3) | 288.4 (32.6-695.9) | 687.1 (77.6-1657.8) | 0.04 (0.03-0.04) |
| Australia | 47253.6 (6168.1-109299.4) | 697.7 (91.1-1613.9) | 58532.9 (7689.2-135006.4) | 704.6 (92.6-1625.2) | 0.01 (0-0.02) |
| Cook Islands | 53.2 (5.4-129.6) | 688.8 (69.9-1677) | 41.8 (4.2-101.5) | 708.7 (71.8-1721.5) | 0.09 (0.08-0.11) |
| Liberia | 4950.8 (559.7-11575.2) | 741.6 (83.8-1733.9) | 14759.1 (1656.5-34900.2) | 743.5 (83.5-1758.1) | 0.03 (0.01-0.05) |
| Greenland | 221.7 (22.7-528.1) | 838 (85.9-1996.5) | 175.7 (18-416.9) | 862.9 (88.2-2047.3) | 0.02 (-0.01-0.05) |
| Tajikistan | 14081 (1963.8-32640.8) | 665.5 (92.8-1542.7) | 27221.3 (3786.9-63602.4) | 671.5 (93.4-1568.9) | 0 (-0.02-0.02) |
| Fiji | 2231.8 (225-5444.5) | 691.7 (69.7-1687.4) | 2499.4 (263-6087.4) | 697.4 (73.4-1698.6) | 0.04 (0.03-0.06) |
| Bermuda | 180.1 (21.3-429.3) | 699.6 (82.8-1668) | 127.4 (15.4-303.8) | 701.9 (84.9-1674.5) | 0.01 (0-0.01) |
| Israel | 17919.4 (1861.4-43020.8) | 937.4 (97.4-2250.5) | 30457 (3160.5-73254.3) | 938.6 (97.4-2257.6) | 0.02 (0.01-0.03) |
| United States Virgin Islands | 278.3 (32.5-669.7) | 702.4 (82.1-1690.3) | 213 (24.9-507.9) | 702.5 (82-1675.3) | 0 (-0.01-0.01) |
| Pakistan | 287285.4 (25952.5-682642.3) | 701.6 (63.4-1667.1) | 630331.4 (49066-1515935.8) | 689.1 (53.6-1657.4) | -0.08 (-0.1--0.06) |
| Guam | 437.5 (45.1-1063.5) | 690.1 (71.1-1677.7) | 418.6 (43.6-1015) | 691.4 (72.1-1676.4) | -0.03 (-0.06-0) |
| Mauritania | 5786.7 (655.2-13614.9) | 753 (85.3-1771.7) | 12138.3 (1363.4-29103.9) | 755.4 (84.8-1811.3) | 0.01 (0.01-0.02) |
| Cambodia | 30737.5 (2616.6-77305.8) | 792.8 (67.5-1993.9) | 56911.6 (5186-141414.8) | 800.3 (72.9-1988.6) | 0.02 (-0.01-0.05) |
| Singapore | 6593.8 (1263.4-14576.8) | 436.9 (83.7-965.8) | 11309.2 (2111.4-25185.7) | 531.4 (99.2-1183.5) | 0.66 (0.46-0.87) |
| Nepal | 54463.4 (5280.4-129111.3) | 745.8 (72.3-1768) | 99493 (9629.2-234784.6) | 760.9 (73.6-1795.6) | 0.08 (0.07-0.09) |
| South Sudan | 10408.5 (1846.8-23539.3) | 445.8 (79.1-1008.2) | 15539.9 (2782.3-35591) | 454.5 (81.4-1041) | 0.09 (0.08-0.11) |
| Slovakia | 13876.5 (2330.3-31288.6) | 677.7 (113.8-1528.2) | 12324.5 (2082.3-28183.8) | 690 (116.6-1577.9) | 0.09 (0.07-0.11) |
| Mongolia | 5843.1 (806.8-13631.7) | 661.7 (91.4-1543.7) | 9295.6 (1308.9-21381.4) | 686.1 (96.6-1578.1) | 0.11 (0.09-0.13) |
| Italy | 217587.2 (20854.4-520303.4) | 1019.1 (97.7-2437) | 177940.1 (16256.7-428042.2) | 1105 (101-2658.1) | 0.39 (0.27-0.52) |
| Kiribati | 210.7 (20.6-517.2) | 690.2 (67.4-1694) | 339.9 (34.1-825.2) | 696.8 (69.8-1691.7) | 0 (-0.01-0.02) |
| Ireland | 12751 (1297.7-30358.6) | 929.5 (94.6-2213) | 15252.6 (1698.5-35826.1) | 948.5 (105.6-2228) | 0.1 (0.09-0.11) |
| Andorra | 232.1 (24.9-553.6) | 927.9 (99.4-2213.5) | 240.6 (28.1-571.8) | 948.8 (110.6-2254.3) | 0.07 (0.03-0.1) |
| Kenya | 42158 (7547.8-95285) | 481.6 (86.2-1088.5) | 105865.4 (19032.8-239475.6) | 488.6 (87.8-1105.2) | 0.08 (0.06-0.09) |
| Mali | 22400.4 (2529.1-52961.2) | 752.5 (85-1779.2) | 61443.4 (6806.4-146752.1) | 751.7 (83.3-1795.3) | 0.01 (0-0.03) |
| Morocco | 87114.2 (12815.6-208408.1) | 837.2 (123.2-2003) | 123586.7 (19188.3-290345.8) | 845.4 (131.3-1986) | 0.03 (0.03-0.04) |
| Romania | 58441.1 (9151.4-132636.7) | 672.5 (105.3-1526.2) | 38428.2 (6382.4-86742.4) | 684.1 (113.6-1544.1) | 0.1 (0.09-0.12) |
| Zimbabwe | 24821 (3237.2-58339.4) | 626.8 (81.7-1473.1) | 39425.1 (5530.8-90928.8) | 635.7 (89.2-1466.1) | 0.08 (0.06-0.11) |
| Eswatini | 1918.7 (258.7-4464.8) | 636.6 (85.8-1481.4) | 3179.2 (426.1-7374.5) | 629.6 (84.4-1460.5) | -0.04 (-0.07--0.01) |
| United States of America | 926976.5 (100139.3-2167683.4) | 909.5 (98.2-2126.7) | 974777.6 (105787.2-2302461.1) | 886.1 (96.2-2093) | -0.07 (-0.18-0.03) |
| Turkmenistan | 10273.2 (1370.6-23915.9) | 668.1 (89.1-1555.3) | 13559.9 (1937.5-31367.1) | 666.9 (95.3-1542.6) | -0.04 (-0.06--0.02) |
| Venezuela (Bolivarian Republic of) | 53715.3 (6334.4-127720.3) | 670.3 (79-1593.7) | 74660.3 (8536.3-177532.7) | 702 (80.3-1669.2) | 0.17 (0.14-0.2) |
| Marshall Islands | 117.7 (11.6-285.2) | 684.4 (67.4-1658.7) | 164.9 (17.7-395.6) | 693.4 (74.4-1663.3) | 0.07 (0.05-0.08) |
| Trinidad and Tobago | 3452.5 (396.5-8258.3) | 688.2 (79-1646.2) | 3532.6 (428.9-8427.5) | 692 (84-1650.8) | 0.02 (0.01-0.03) |
| Taiwan (Province of China) | 56920.8 (6469.7-133016.4) | 617.1 (70.1-1442) | 51223.1 (5852-121059.9) | 645.1 (73.7-1524.6) | 0.18 (0.15-0.22) |
| Angola | 24364.1 (3326.2-56626.7) | 619.2 (84.5-1439.2) | 70686.4 (9432.9-166115.2) | 630.9 (84.2-1482.6) | 0.08 (0.08-0.09) |
| Palestine | 6284.4 (921.8-15025.7) | 820.9 (120.4-1962.6) | 17329.6 (2536-41172.9) | 829.3 (121.4-1970.2) | 0.03 (0.03-0.04) |
| Suriname | 1095.9 (119.3-2597.1) | 678.1 (73.8-1607.1) | 1479.1 (169.9-3553.5) | 688.7 (79.1-1654.6) | 0.05 (0.05-0.06) |
| Saint Lucia | 387.9 (42.5-934.9) | 689.9 (75.5-1662.7) | 465.6 (52.3-1118.5) | 689.6 (77.4-1656.5) | -0.01 (-0.01-0) |
| Niger | 20841.5 (2317.2-49132.2) | 749.9 (83.4-1768) | 60723.6 (6557.3-145200) | 747.3 (80.7-1786.9) | 0 (-0.01-0.01) |
| Bahamas | 817.8 (91.1-1970.5) | 692.7 (77.2-1668.9) | 1040.9 (118.7-2488.7) | 695.4 (79.3-1662.5) | 0.01 (0-0.02) |
| Ethiopia | 76784 (13505-177529.2) | 411.6 (72.4-951.7) | 182182.6 (29586.5-421638.3) | 410.4 (66.7-949.9) | 0.01 (0-0.03) |
| Micronesia (Federated States of) | 272.9 (26.8-663.1) | 683 (67.2-1659.7) | 289.6 (29.1-710.5) | 685.1 (68.9-1680.8) | 0.01 (0-0.01) |
| Lao People's Democratic Republic | 12227.2 (1077.9-30799.8) | 791.1 (69.7-1992.7) | 25042.4 (2213.3-62676.8) | 795.4 (70.3-1990.8) | 0.01 (0-0.03) |
| Belarus | 26949.1 (5709.6-58736.9) | 681.9 (144.5-1486.2) | 21163.3 (4589.1-45955.8) | 693.7 (150.4-1506.3) | 0.05 (0.01-0.08) |
| Malta | 1297.9 (143.1-3074.2) | 941 (103.7-2228.8) | 1303.9 (146.6-3080.4) | 945.2 (106.2-2233) | 0.01 (0-0.03) |
| Samoa | 449 (44.3-1095.7) | 673 (66.3-1642.4) | 569.8 (57.1-1400.3) | 684.8 (68.7-1682.9) | 0.05 (0.03-0.06) |
| Brazil | 568919.2 (45383.3-1398387.4) | 906.6 (72.3-2228.5) | 803380.6 (66095.6-1961361.2) | 932.8 (76.7-2277.4) | 0.15 (0.09-0.2) |
| Dominica | 197.9 (21.6-475.1) | 678.5 (73.9-1628.9) | 176.5 (19.5-417.8) | 684.5 (75.5-1620.7) | 0.03 (0.02-0.04) |
| Latvia | 6472.4 (1350-14130.8) | 678.4 (141.5-1481.1) | 3861.2 (817.2-8348.1) | 687.8 (145.6-1487) | 0.04 (0.01-0.06) |
| Uzbekistan | 57208.4 (7855.8-133036.2) | 666 (91.4-1548.7) | 96380.3 (13620-223448.3) | 675.6 (95.5-1566.3) | 0.03 (0.02-0.05) |
| Philippines | 209510.1 (18668.2-520263.6) | 806.9 (71.9-2003.8) | 376542.8 (34436.4-932986.6) | 816.3 (74.7-2022.6) | 0.03 (0.03-0.04) |
| Luxembourg | 1363.3 (172.2-3156.5) | 923.5 (116.6-2138.2) | 1974.7 (261.9-4570.8) | 925.2 (122.7-2141.6) | -0.06 (-0.08--0.03) |
| Mauritius | 3978.4 (349.5-9988.3) | 796.3 (70-1999.3) | 3756.1 (345.7-9351.4) | 801.4 (73.8-1995.2) | 0.02 (0.01-0.03) |
| Paraguay | 13663.5 (1058.8-33945) | 872.3 (67.6-2167.2) | 25620.5 (2113.2-63504.7) | 862.1 (71.1-2136.8) | -0.04 (-0.05--0.03) |
| Benin | 12902.3 (1432.2-30463) | 757.6 (84.1-1788.9) | 36804.6 (4138.7-86705.4) | 753.1 (84.7-1774.1) | -0.02 (-0.03--0.02) |
| Malaysia | 52084.5 (5268.9-129076.5) | 702.8 (71.1-1741.7) | 100607.5 (10119.6-251890.8) | 730 (73.4-1827.8) | 0.16 (0.12-0.19) |
| Ecuador | 23701.1 (3147.6-54026) | 575 (76.4-1310.7) | 43068.3 (5552.2-101805.9) | 597.2 (77-1411.7) | 0.19 (0.16-0.22) |
| Monaco | 87.6 (9.5-208.3) | 957.3 (103.8-2274.9) | 88 (9.6-207.3) | 947.7 (103.5-2233.8) | -0.04 (-0.04--0.04) |
| Qatar | 1778.2 (294.1-4108.1) | 752.4 (124.5-1738.2) | 12459.1 (2093.7-27957) | 725.3 (121.9-1627.4) | -0.24 (-0.32--0.17) |
| El Salvador | 14456.8 (1566.1-34609.2) | 693 (75.1-1659.1) | 18159.4 (1950.1-43599.3) | 704.8 (75.7-1692.1) | 0.05 (0.04-0.07) |
| Armenia | 9782.7 (1396.1-22664.1) | 680.4 (97.1-1576.4) | 7733.5 (1136.6-17721.8) | 693.7 (101.9-1589.6) | 0.04 (0.01-0.08) |
| Iran (Islamic Republic of) | 196519.3 (30935.2-468871.4) | 869.8 (136.9-2075.3) | 319782.5 (55308.5-734457.9) | 898.1 (155.3-2062.6) | 0.12 (0.04-0.2) |
| Cuba | 33328.6 (3760.7-79211.5) | 685.2 (77.3-1628.5) | 24943.2 (2931.1-59993.1) | 687.8 (80.8-1654.2) | 0 (-0.02-0.01) |
| Nigeria | 266977.4 (30341.5-628621) | 783.9 (89.1-1845.6) | 654897.1 (75623.4-1519303.9) | 781 (90.2-1811.8) | 0.01 (0-0.02) |
| Myanmar | 133583.1 (12037.9-332420.9) | 788.7 (71.1-1962.8) | 177742.9 (15883.9-442936.7) | 804.3 (71.9-2004.4) | 0.07 (0.07-0.08) |
| Malawi | 16526 (2898.1-37650.8) | 457 (80.1-1041.1) | 35043.4 (6059.3-79348.5) | 459.8 (79.5-1041) | 0.07 (0.05-0.08) |
| Oman | 6269.6 (982.3-14733) | 781 (122.4-1835.2) | 19652.4 (3186.4-45087.9) | 766.5 (124.3-1758.6) | -0.1 (-0.18--0.02) |
| Congo | 5885.9 (781.3-13581.6) | 620.5 (82.4-1431.8) | 13373.2 (1805.9-30887.6) | 635.1 (85.8-1466.8) | 0.11 (0.1-0.12) |
| Madagascar | 20691.9 (3604.9-47167.6) | 457.3 (79.7-1042.5) | 50439 (8953.2-114418.3) | 461.7 (82-1047.4) | 0.04 (0.04-0.05) |
| Papua New Guinea | 11237 (1148.8-27296.8) | 679 (69.4-1649.4) | 28198.8 (2856.3-68685.4) | 689.1 (69.8-1678.4) | 0.04 (0.03-0.05) |
| Indonesia | 637944.6 (56747.5-1577646.3) | 817.2 (72.7-2021.1) | 876626.7 (82436.6-2174066.8) | 827.6 (77.8-2052.5) | 0.06 (0.05-0.06) |
| New Zealand | 10005.8 (1332.4-23121.2) | 724 (96.4-1672.9) | 10299.9 (1358.4-23748.7) | 730.3 (96.3-1683.9) | 0 (-0.02-0.02) |
| Bolivia (Plurinational State of) | 13028.9 (1867.6-29722.6) | 528.9 (75.8-1206.5) | 25608.1 (3740.2-57833) | 529.7 (77.4-1196.2) | 0 (-0.01-0) |
| Sao Tome and Principe | 320.3 (35.6-765.5) | 746.1 (82.8-1783) | 652.1 (76.1-1537.4) | 751 (87.6-1770.5) | 0.03 (0.03-0.04) |
| Antigua and Barbuda | 179.9 (19.6-432.2) | 697.2 (76.1-1675.6) | 240.6 (28-582.5) | 695.1 (80.8-1683.1) | -0.02 (-0.05-0.01) |
| Belgium | 39991 (3852.1-98297.8) | 1075 (103.6-2642.4) | 41379.9 (3607.4-98833.5) | 1180.1 (102.9-2818.5) | 0.34 (0.25-0.43) |
| Nauru | 28.1 (2.8-68.2) | 695.5 (69-1685.2) | 31.6 (3.2-76.3) | 693.4 (70.2-1671.8) | -0.01 (-0.02-0) |
| Burkina Faso | 24029.7 (2725.2-56703.7) | 752.5 (85.3-1775.8) | 64611.2 (7396.3-151176) | 756.9 (86.6-1771) | 0.03 (0.02-0.04) |
| Bosnia and Herzegovina | 12916.1 (2107.5-29232.8) | 668.7 (109.1-1513.5) | 7091.3 (1205.3-16051.8) | 681.4 (115.8-1542.4) | 0.03 (0-0.06) |
| Bulgaria | 20167.4 (3289.8-45141.5) | 677.6 (110.5-1516.7) | 13563.2 (2324.8-30863.4) | 686.7 (117.7-1562.7) | 0.07 (0.06-0.08) |
| Democratic Republic of the Congo | 87862.7 (12094.8-203644.5) | 614.6 (84.6-1424.5) | 216729.2 (29344.4-505198.1) | 619.5 (83.9-1444.1) | 0.03 (0.01-0.04) |
| Norway | 14288.8 (1418-33295) | 893.2 (88.6-2081.3) | 18108 (1656.1-42675.1) | 1028.9 (94.1-2424.9) | 0.63 (0.57-0.7) |
| Algeria | 84351 (12341.3-202245.7) | 833 (121.9-1997.2) | 146268.3 (22752.2-342601.1) | 852.8 (132.7-1997.5) | 0.08 (0.07-0.09) |
| Slovenia | 5200.9 (853.7-11711.4) | 678.7 (111.4-1528.3) | 4068.7 (683.2-9174.7) | 684.9 (115-1544.4) | 0.04 (0.03-0.05) |
| Portugal | 35373.1 (3695.2-84516.9) | 934.4 (97.6-2232.5) | 28923.4 (3166.6-68231.9) | 950.4 (104.1-2242.1) | 0.09 (0.08-0.1) |
| Chile | 34217.5 (4906.2-78748.7) | 597.5 (85.7-1375.1) | 42266.4 (6401.2-95889.6) | 621.1 (94.1-1409.1) | 0.16 (0.14-0.18) |
| Solomon Islands | 871.2 (85.5-2128.8) | 678 (66.5-1656.7) | 1808.1 (182.5-4396.2) | 691.1 (69.8-1680.5) | 0.09 (0.08-0.1) |
| Cabo Verde | 985.3 (106.4-2331.8) | 754.4 (81.4-1785.5) | 1879.9 (222.8-4384.5) | 755.8 (89.6-1762.7) | -0.02 (-0.04-0) |
| Czechia | 25072 (4179.9-56117.7) | 674.7 (112.5-1510.2) | 20918.7 (3641.9-47202.1) | 687.1 (119.6-1550.3) | 0.11 (0.1-0.13) |
| Netherlands | 49986.5 (5220.9-116405.8) | 829 (86.6-1930.6) | 47670.2 (4652.1-113768.2) | 905.6 (88.4-2161.3) | 0.47 (0.36-0.58) |
| Senegal | 20668.8 (2313.9-48879.1) | 749.4 (83.9-1772.3) | 45826.8 (5197.3-108018.8) | 747.6 (84.8-1762.3) | 0 (0-0.01) |
| Northern Mariana Islands | 166.5 (17.5-405) | 710.8 (74.9-1728.7) | 93.7 (9.4-229.6) | 673.1 (67.2-1648.9) | -0.25 (-0.36--0.14) |
| Tunisia | 28926.4 (4284.5-69017.4) | 839.4 (124.3-2002.9) | 38101.4 (5962.7-87894.2) | 859.8 (134.5-1983.3) | 0.09 (0.08-0.09) |
| Hungary | 25123.8 (4090.1-56633.6) | 679.5 (110.6-1531.7) | 19525.6 (3273.5-44493.7) | 685.2 (114.9-1561.4) | 0.09 (0.07-0.1) |
| Sierra Leone | 10275.1 (1157.9-24053.8) | 753.2 (84.9-1763.3) | 26254.1 (2939.9-62291.9) | 750.9 (84.1-1781.6) | -0.03 (-0.04--0.02) |
| Guyana | 2318 (249.8-5625) | 681.3 (73.4-1653.4) | 2210.8 (248-5338.1) | 683.6 (76.7-1650.5) | 0.01 (0.01-0.01) |
| Central African Republic | 6502.1 (880-15090.9) | 619.3 (83.8-1437.3) | 13204.5 (1803.5-30642.9) | 625.8 (85.5-1452.2) | 0.05 (0.04-0.06) |
| Germany | 293720.2 (30638.1-698990) | 988.8 (103.1-2353.1) | 261806.1 (26684.4-620025.3) | 1027.4 (104.7-2433) | 0 (-0.11-0.11) |
| Kuwait | 6960 (1069.9-16430.9) | 798.1 (122.7-1884.1) | 17611.7 (2887.8-40722.5) | 843.5 (138.3-1950.5) | 0.19 (0.14-0.25) |
| Mozambique | 21187.2 (3807.8-47840.9) | 463.1 (83.2-1045.7) | 51577.3 (8867.4-117721.3) | 459.5 (79-1048.8) | -0.01 (-0.02-0) |
| Grenada | 228.2 (24.5-550.1) | 684.7 (73.6-1650.4) | 278.1 (31.2-667.8) | 681.3 (76.4-1636.2) | -0.01 (-0.02-0) |
| Saudi Arabia | 53591.3 (8204.9-129119.7) | 799.2 (122.4-1925.6) | 146842.3 (24171.8-337974.1) | 801.5 (131.9-1844.7) | 0 (-0.01-0.01) |
| Colombia | 98550.5 (10838.4-231501.8) | 700.3 (77-1645) | 135352.2 (15228.5-326627.8) | 707.6 (79.6-1707.6) | 0.04 (0.03-0.05) |
| Russian Federation | 442308.6 (99748.9-958718.9) | 760 (171.4-1647.4) | 372319.7 (88695.6-809152.3) | 775.4 (184.7-1685) | 0.16 (0.12-0.19) |
| Cameroon | 28384.4 (3265.2-67383.2) | 747.3 (86-1774) | 90707.6 (10488.2-213801.6) | 750.7 (86.8-1769.4) | 0.02 (0.01-0.03) |
| Syrian Arab Republic | 39866.8 (5816.9-94963.8) | 827.8 (120.8-1971.8) | 46783.8 (7017.9-113249.7) | 849.3 (127.4-2055.8) | 0.11 (0.09-0.14) |
| Lithuania | 8788.9 (1680-19534.8) | 630.9 (120.6-1402.2) | 5140.1 (1019.9-11418.3) | 612.1 (121.5-1359.7) | -0.18 (-0.23--0.13) |
| Albania | 9451.2 (1505.5-21469.5) | 666.4 (106.2-1513.8) | 6496.9 (1078.6-14675.9) | 669.6 (111.2-1512.5) | -0.03 (-0.05--0.01) |
| Chad | 15775.2 (1774.6-37430.9) | 751.7 (84.6-1783.6) | 43377.3 (4781.1-103644.3) | 749.2 (82.6-1790.2) | -0.01 (-0.01-0) |
| Austria | 27038.5 (3513.4-63473.3) | 901.1 (117.1-2115.2) | 25481.7 (3404.1-59427.1) | 905.7 (121-2112.3) | 0.07 (0.03-0.12) |
| Rwanda | 12531 (2226.4-28664.9) | 458.7 (81.5-1049.2) | 25039.1 (4386.1-56643.6) | 462.5 (81-1046.2) | 0.07 (0.03-0.1) |
| Belize | 501 (54.9-1203.7) | 684 (75-1643.3) | 1236.5 (137.3-2975.1) | 690.5 (76.7-1661.3) | 0.03 (0.02-0.04) |
| Finland | 17117.7 (1837.9-40738.8) | 943 (101.2-2244.2) | 15702.8 (1703.1-37193.7) | 938.6 (101.8-2223.2) | -0.02 (-0.03--0.01) |
| Egypt | 185277.2 (27321.2-445832.6) | 845.6 (124.7-2034.7) | 361507 (52397.1-855415) | 881.4 (127.7-2085.5) | 0.21 (0.17-0.24) |
| Vanuatu | 403.4 (41.3-978.7) | 688.4 (70.5-1670) | 820.3 (84-2004.4) | 695 (71.2-1698) | 0.03 (0.02-0.03) |
| Thailand | 253585.8 (18414.6-648660.1) | 979 (71.1-2504.2) | 216755.2 (18383-543103.4) | 893.2 (75.8-2238.1) | -0.38 (-0.49--0.27) |
| Togo | 10276.9 (1151.5-24206.2) | 749.7 (84-1765.8) | 24159.1 (2788.1-57356) | 757.9 (87.5-1799.3) | 0.04 (0.04-0.05) |
| Spain | 140818.1 (14660.6-330653.4) | 949.5 (98.9-2229.5) | 126487.8 (15852.1-296205.6) | 974.6 (122.1-2282.4) | 0.13 (0.12-0.14) |
| Peru | 37790.5 (6579-85344) | 426.1 (74.2-962.4) | 64949.1 (10597.6-148241.4) | 476.4 (77.7-1087.4) | 0.44 (0.33-0.56) |
| Niue | 5.5 (0.6-13.3) | 686.3 (69.6-1655.4) | 3.9 (0.4-9.4) | 693.6 (69.6-1681.6) | 0.04 (0.03-0.05) |
| Turkey | 200494.4 (32535-473448.6) | 810.9 (131.6-1915) | 279340.4 (47094.4-652297.7) | 820.6 (138.3-1916.2) | -0.01 (-0.04-0.02) |
| Tonga | 250.6 (24.2-613.1) | 680 (65.7-1663.4) | 267.5 (26.3-651.4) | 693.9 (68.2-1689.7) | 0.09 (0.08-0.09) |
| Gambia | 2835 (333.9-6732.1) | 750.1 (88.3-1781.1) | 7069.5 (803.4-16885.2) | 750.5 (85.3-1792.6) | 0.01 (0-0.01) |
| Sweden | 27647.8 (2726.6-65094) | 942 (92.9-2217.8) | 30085.5 (2903-71376.7) | 942.1 (90.9-2235.1) | -0.01 (-0.02-0.01) |
| Ukraine | 135695.1 (29523.6-299686.4) | 714.7 (155.5-1578.4) | 105405.6 (23958.4-232030.9) | 729.8 (165.9-1606.5) | 0.08 (0.05-0.12) |
| Estonia | 3857.8 (809.3-8341.1) | 679.2 (142.5-1468.6) | 2754.3 (606.2-5970.2) | 684.6 (150.7-1483.9) | 0.02 (0-0.04) |
| Cyprus | 2878 (306.9-6851.6) | 935.7 (99.8-2227.7) | 4799.2 (547.4-11351.7) | 958.8 (109.4-2267.9) | 0.09 (0.07-0.1) |
| Saint Kitts and Nevis | 119.7 (13.1-288.4) | 689.8 (75.3-1661.6) | 157.2 (17.9-375.9) | 693.9 (78.9-1659) | 0.02 (0.01-0.03) |
| Palau | 48.4 (5-118.4) | 691.5 (71.9-1692.5) | 41.7 (4.4-100) | 682.7 (72.8-1638.8) | -0.08 (-0.11--0.05) |
| Azerbaijan | 21522 (2958.9-50033.8) | 676.7 (93-1573.1) | 29251 (4220.6-68011.2) | 685.4 (98.9-1593.6) | -0.02 (-0.05-0.01) |
| United Arab Emirates | 7282.3 (1190.3-16512.7) | 760.2 (124.3-1723.7) | 33921.7 (6348.2-75404.6) | 756.6 (141.6-1681.9) | -0.09 (-0.13--0.04) |
| Equatorial Guinea | 940.8 (127.2-2188.5) | 627.1 (84.8-1458.8) | 3942.6 (526.3-9122.6) | 605.8 (80.9-1401.7) | -0.13 (-0.14--0.12) |
| Maldives | 633.2 (56-1587.6) | 779 (68.9-1953.2) | 1939.1 (192-4734.4) | 769.2 (76.2-1878.1) | -0.07 (-0.09--0.04) |
| Canada | 99866.6 (10245.6-233209.7) | 898.2 (92.2-2097.5) | 100793.3 (11006.9-238155.6) | 878 (95.9-2074.6) | -0.1 (-0.12--0.09) |
| Montenegro | 1680.5 (273.4-3801.6) | 670.3 (109-1516.4) | 1431.7 (241.7-3262.3) | 679 (114.6-1547.2) | 0.06 (0.05-0.06) |
| C么te d'Ivoire | 34836.5 (3981.9-81260) | 736.5 (84.2-1717.9) | 80552.4 (9097.7-190051.9) | 748.1 (84.5-1765.1) | 0.06 (0.05-0.07) |
| United Republic of Tanzania | 43004.2 (7798.9-93793.6) | 444.3 (80.6-969) | 105496.7 (17869-238345.8) | 477.8 (80.9-1079.4) | 0.39 (0.31-0.46) |
| Somalia | 11433.5 (1970.5-26078.9) | 452.8 (78-1032.9) | 35335.6 (6208-81189.2) | 450.6 (79.2-1035.2) | 0.06 (0.03-0.09) |
| Croatia | 12319.9 (2043.8-27959.6) | 679.1 (112.7-1541.2) | 8775.1 (1449.8-20086.8) | 680.9 (112.5-1558.6) | -0.09 (-0.18-0) |
| Bahrain | 2045.1 (324.6-4746.4) | 796.4 (126.4-1848.3) | 4969 (842.7-11214.3) | 803.6 (136.3-1813.6) | -0.07 (-0.11--0.03) |
| Puerto Rico | 9846.2 (1089.8-23665.6) | 696.3 (77.1-1673.5) | 7837 (916.5-18594.4) | 697.6 (81.6-1655.1) | 0 (0-0.01) |
| Jordan | 12615.6 (1836.5-30458.1) | 816.7 (118.9-1971.7) | 41252.4 (6289.7-97831.2) | 823.2 (125.5-1952.2) | 0.03 (0.01-0.06) |
| Ghana | 43073.5 (4909.6-101534.5) | 749.2 (85.4-1766) | 103178 (11584-241047.6) | 757.9 (85.1-1770.7) | 0.04 (0.04-0.04) |
| Greece | 37002.4 (3671.6-87179) | 984.5 (97.7-2319.6) | 28802.8 (3108.6-67534.5) | 990.4 (106.9-2322.2) | 0.03 (0.02-0.04) |
| Yemen | 38334.1 (5722.3-92078) | 831.3 (124.1-1996.7) | 108093.1 (16217.1-256451) | 830.9 (124.7-1971.3) | 0.03 (0.01-0.05) |
| Guatemala | 19992.4 (2169.7-48003.6) | 689 (74.8-1654.4) | 55108.3 (5965.6-131747.5) | 695.9 (75.3-1663.6) | 0.04 (0.03-0.05) |
| Seychelles | 247.4 (22-621.7) | 791.9 (70.4-1990.4) | 305.7 (28.6-755.9) | 792.2 (74-1958.6) | -0.03 (-0.05--0.01) |
| Guinea-Bissau | 2821.8 (311.8-6713.1) | 751.7 (83.1-1788.2) | 6067.2 (678-14337.6) | 755 (84.4-1784.2) | 0.02 (0.01-0.03) |
| Costa Rica | 8920.3 (973.4-21432.9) | 694.7 (75.8-1669.1) | 13469.1 (1526.3-31731.9) | 703.1 (79.7-1656.4) | 0.04 (0.03-0.05) |
| Zambia | 15299.1 (2831.4-34612.5) | 504.3 (93.3-1141) | 38234.5 (7495.4-85194.1) | 503.9 (98.8-1122.8) | 0 (-0.03-0.03) |
| United Kingdom | 191595.5 (21660.4-443491.1) | 918.7 (103.9-2126.5) | 203245.5 (23856.5-468439.5) | 929.3 (109.1-2141.9) | 0.01 (-0.01-0.02) |
| Denmark | 15371.6 (1906.1-35616.7) | 805.7 (99.9-1866.8) | 15061.9 (1719.7-35142.4) | 835.6 (95.4-1949.6) | 0.13 (0.07-0.18) |
| Switzerland | 21844.8 (2692.7-50009.2) | 829 (102.2-1897.9) | 23832.5 (2877.5-55064.4) | 858.9 (103.7-1984.5) | 0.12 (0.1-0.13) |
| Republic of Moldova | 11951.4 (2603.4-26253.2) | 685.3 (149.3-1505.5) | 9062.1 (2010.3-19751.8) | 690.6 (153.2-1505.2) | 0.02 (-0.02-0.07) |
| France | 207161.2 (25808-482622.7) | 941.9 (117.3-2194.3) | 184232.8 (23243.8-425766.8) | 926.6 (116.9-2141.4) | -0.04 (-0.07--0.01) |
| Gabon | 2368.7 (319.9-5454) | 620.6 (83.8-1429) | 4731.7 (642.1-10938.4) | 635.9 (86.3-1470.1) | 0.08 (0.07-0.08) |
| Djibouti | 900.2 (161.4-2031.6) | 448.6 (80.4-1012.4) | 2370.9 (426.4-5353.3) | 469.7 (84.5-1060.6) | 0.18 (0.17-0.2) |
| Brunei Darussalam | 671.5 (100.6-1566.4) | 544.6 (81.6-1270.4) | 1109 (173.5-2567.4) | 549.8 (86-1272.8) | 0 (-0.02-0.03) |
| American Samoa | 140.2 (13.8-345.2) | 692.7 (68.3-1705.2) | 144.3 (14-350) | 684.8 (66.5-1660.4) | -0.05 (-0.07--0.04) |
| Sri Lanka | 58726.3 (5170.5-146508.2) | 793.9 (69.9-1980.5) | 64906.6 (6016.2-162449) | 807.6 (74.9-2021.3) | 0.08 (0.07-0.09) |
| Burundi | 9569.2 (1646.8-21559.9) | 461.9 (79.5-1040.8) | 21189.8 (3703.6-48071.8) | 458.6 (80.1-1040.3) | -0.04 (-0.09-0) |
| Iraq | 54119.9 (8017.1-128495.6) | 823.1 (121.9-1954.3) | 154928 (23571.2-371832.3) | 829.7 (126.2-1991.3) | 0.04 (0.03-0.05) |
| Dominican Republic | 21326.6 (2341-51802.3) | 693.8 (76.2-1685.3) | 30904.8 (3441.5-74008.4) | 687.2 (76.5-1645.6) | -0.05 (-0.06--0.05) |
| Guinea | 16332.8 (1862.3-38387) | 756.4 (86.2-1777.8) | 36679.4 (4225.7-86433.7) | 760.5 (87.6-1792.1) | 0.03 (0.02-0.04) |
| Afghanistan | 31427.3 (4444.3-73873.6) | 811.6 (114.8-1907.9) | 122751.4 (18028.6-293408) | 814.6 (119.6-1947) | 0.05 (0.03-0.07) |
| North Macedonia | 5459.7 (889.9-12482.9) | 672 (109.5-1536.5) | 5271.1 (882.2-11844.4) | 682.2 (114.2-1533) | 0.04 (0.03-0.05) |
| Honduras | 11980.8 (1251.3-28838.2) | 694.3 (72.5-1671.2) | 29656.7 (3236.5-71110.1) | 701.8 (76.6-1682.8) | 0.05 (0.04-0.06) |
| Bangladesh | 302052.3 (27638.8-718545.9) | 707.7 (64.8-1683.5) | 491209.8 (44979.9-1183483.4) | 724.4 (66.3-1745.4) | 0.08 (0.08-0.09) |
| Tokelau | 4 (0.4-9.7) | 690.4 (68.4-1688) | 3.3 (0.3-8.1) | 690.1 (70-1678.2) | -0.02 (-0.04-0) |
| Lesotho | 4328 (587.4-10013.3) | 627.3 (85.1-1451.4) | 5922.2 (799.7-13791.8) | 623.4 (84.2-1451.8) | -0.01 (-0.04-0.01) |
| Uganda | 28975.5 (5086.3-64935.2) | 451.5 (79.3-1011.8) | 74153.3 (13142.7-168839.6) | 459 (81.3-1045) | 0.07 (0.06-0.07) |
| Argentina | 69202.2 (10446.1-158682.6) | 566.2 (85.5-1298.3) | 104627.1 (15630.9-238652.4) | 600.7 (89.7-1370.1) | 0.28 (0.23-0.33) |
| Tuvalu | 25.5 (2.6-62.1) | 708.9 (72.1-1724) | 32.8 (3.4-80) | 684.8 (70.4-1669.9) | -0.15 (-0.16--0.13) |
| Barbados | 760 (86.8-1821.6) | 696 (79.5-1668.2) | 689.2 (79.7-1655.6) | 696.8 (80.6-1673.8) | 0 (0-0.01) |
| San Marino | 85 (8.5-205.2) | 937.4 (94-2263.9) | 98.6 (10.8-233.4) | 958 (105.4-2268.4) | 0.07 (0.03-0.1) |
| Comoros | 788.7 (136-1791.6) | 457.1 (78.8-1038.4) | 1378.3 (236.8-3117.3) | 465.2 (79.9-1052.2) | 0.06 (0.06-0.07) |
| Botswana | 3240.3 (420.7-7476.3) | 629.7 (81.8-1452.9) | 6724.2 (941.2-15712.6) | 637.9 (89.3-1490.6) | 0.06 (0.04-0.09) |
| Sudan | 64048 (9373.5-152535.9) | 839.8 (122.9-2000.1) | 145330.2 (22026.9-346363.7) | 842.1 (127.6-2007) | 0.03 (0.02-0.03) |

Abbreviations: EAPC, estimated annual percentage change; SDI, Sociodemographic Index; UI, uncertainty interval.
