# Supplementary material for: Global, regional, and national epidemiology of migraine and tension-type headache in youths and young adults aged 15–39 years from 1990 to 2019: findings from the global burden of disease study 2019
Source: J Headache Pain. 2023 Sep 18;24(1):126. doi: 10.1186/s10194-023-01659-1 (PMC10506184; doi:10.1186/s10194-023-01659-1)
Supplement: Supplementary file 16 — Additional file 16: Table S9. DALYs of TTH Between 1990 and 2019 in 15 to 39 years at the 204 Country Level. [file 10194_2023_1659_MOESM16_ESM.docx]

| **TableS9.DALYs of TTH Between 1990 and 2019 in 15 to 39 years at the 204 Country Level** | | | | | |
| --- | --- | --- | --- | --- | --- |
|  | 1990 | | 2019 | |  |
| location | Number_95%UI | ASR | Number_95%UI | ASR | EAPC_95%CI |
| Mexico | 22850.8 (6002.2-92809.2) | 64.1 (16.8-260.4) | 33253 (8929.4-129736.7) | 66.3 (17.8-258.5) | 0.1 (0.09-0.12) |
| Haiti | 1459.5 (388.9-6134.7) | 60.1 (16-252.4) | 3242.1 (850.2-13127.6) | 61 (16-247) | 0.07 (0.05-0.1) |
| Viet Nam | 16823 (4000-71351.4) | 59 (14-250.2) | 24447.6 (6005.2-101033.2) | 62 (15.2-256.1) | 0.14 (0.12-0.16) |
| Bhutan | 148.5 (33.8-658.3) | 57.1 (13-253.2) | 208 (48.3-894.1) | 59.2 (13.8-254.6) | 0.11 (0.1-0.13) |
| Jamaica | 590.3 (150.5-2418.6) | 60.1 (15.3-246.1) | 736.9 (188.1-2914.9) | 61.7 (15.8-244.2) | 0.05 (0.02-0.07) |
| Nicaragua | 874 (224.5-3684) | 59.4 (15.2-250.2) | 1721.2 (452-6898.9) | 61.4 (16.1-245.9) | 0.12 (0.11-0.14) |
| Kyrgyzstan | 1442 (341.5-5216.3) | 79.9 (18.9-289.1) | 2166.9 (518.1-7828.2) | 81.4 (19.5-294) | 0.02 (-0.01-0.05) |
| Georgia | 1747.8 (418.1-6286) | 82.1 (19.6-295.2) | 981.8 (232.3-3512) | 83 (19.6-296.8) | -0.03 (-0.07-0.01) |
| Lebanon | 967.8 (319.7-2863.8) | 80.4 (26.5-237.8) | 1747.9 (575.1-4907.6) | 85.1 (28-239.1) | 0.17 (0.16-0.19) |
| Kazakhstan | 5508 (1308.8-19697.9) | 81.1 (19.3-289.9) | 5774.6 (1407.6-20413.1) | 83.1 (20.3-293.8) | 0.05 (0.01-0.09) |
| Namibia | 353.7 (97.4-1352.5) | 63.3 (17.4-241.9) | 666.4 (179.9-2521.9) | 65.3 (17.6-247) | 0.1 (0.08-0.11) |
| Republic of Korea | 15012.7 (3590.4-60553.8) | 71.3 (17-287.4) | 13539.4 (3057.1-52160) | 78.1 (17.6-300.9) | 0.31 (0.28-0.34) |
| Timor-Leste | 186.2 (43.7-811.4) | 58.5 (13.7-255) | 310.1 (71.5-1322.6) | 57.7 (13.3-246) | -0.06 (-0.08--0.05) |
| China | 271137.9 (79981.1-1033189.5) | 49.3 (14.6-188) | 265254.1 (76838.9-943708.8) | 53.3 (15.4-189.6) | 0.26 (0.23-0.29) |
| Eritrea | 657 (187-2471) | 56.7 (16.2-213.4) | 1669.3 (480.1-6253.4) | 58.1 (16.7-217.5) | 0.08 (0.07-0.09) |
| Iceland | 87.9 (21.1-322.7) | 84.6 (20.3-310.6) | 103 (23.5-379.2) | 85.9 (19.6-316.2) | 0.01 (-0.01-0.02) |
| Panama | 608.2 (153.9-2436.9) | 60.2 (15.2-241.1) | 983.5 (244.7-3909.9) | 61.5 (15.3-244.6) | 0.06 (0.05-0.08) |
| Serbia | 2991 (784-10136.4) | 87 (22.8-294.8) | 2483.8 (664-8305.9) | 88 (23.5-294.1) | 0.08 (0.06-0.09) |
| India | 208328.5 (51005.8-866833.9) | 61.1 (15-254.4) | 371811.8 (90505.2-1555151.7) | 62.4 (15.2-260.9) | -0.01 (-0.06-0.03) |
| Libya | 1290.7 (407.8-3898.7) | 78.8 (24.9-237.9) | 2542.2 (809.4-7215) | 84 (26.7-238.4) | 0.25 (0.24-0.27) |
| South Africa | 10953.6 (3015.8-41086.3) | 68.9 (19-258.4) | 17087.4 (4791.3-63803.4) | 71.3 (20-266.4) | 0.09 (0.06-0.13) |
| Democratic People's Republic of Korea | 4021.5 (1098.6-15461) | 48.7 (13.3-187.3) | 5016.3 (1421.4-19053.3) | 49.9 (14.1-189.6) | 0 (-0.04-0.04) |
| Uruguay | 806.3 (209.3-2949.7) | 71 (18.4-259.7) | 870.1 (227.4-3057) | 72 (18.8-253.1) | 0.05 (0.04-0.06) |
| Japan | 34572.5 (8432.9-128758.4) | 77.1 (18.8-287.1) | 26007.2 (6420-93722.5) | 79.1 (19.5-285) | 0.16 (0.12-0.2) |
| Poland | 13480.2 (3748.1-43564.8) | 93.2 (25.9-301.3) | 12154.7 (3355.5-39028.9) | 96.2 (26.5-308.8) | 0.15 (0.1-0.21) |
| Saint Vincent and the Grenadines | 27.3 (7.1-114.3) | 59.5 (15.4-249) | 25.8 (6.6-102.7) | 61.4 (15.8-244.6) | 0.11 (0.1-0.12) |
| Australia | 4837.7 (1233.7-17733.2) | 71.4 (18.2-261.8) | 6070.1 (1540.1-21571.2) | 73.1 (18.5-259.7) | 0.03 (0.01-0.05) |
| Cook Islands | 4.3 (1.1-17.5) | 56.1 (14.2-226.7) | 3.4 (0.8-13.5) | 57.5 (14.1-228.6) | 0.08 (0.05-0.11) |
| Liberia | 434.4 (114.6-1745.4) | 65.1 (17.2-261.5) | 1297.7 (339.7-5128) | 65.4 (17.1-258.3) | 0.09 (0.05-0.12) |
| Greenland | 21.2 (4.5-80.3) | 80 (17.2-303.4) | 16.6 (3.7-63) | 81.4 (18.2-309.6) | -0.13 (-0.2--0.05) |
| Tajikistan | 1668.7 (399.2-6137.5) | 78.9 (18.9-290.1) | 3261.7 (774-11635.1) | 80.5 (19.1-287) | 0.02 (-0.01-0.05) |
| Fiji | 181 (45-730.6) | 56.1 (14-226.4) | 205.6 (52.1-819.7) | 57.4 (14.5-228.7) | 0.09 (0.08-0.1) |
| Bermuda | 16.4 (4.2-64.4) | 63.5 (16.4-250.4) | 11.7 (3.1-45.4) | 64.2 (17.1-250.4) | 0.02 (0-0.03) |
| Israel | 1607.8 (375-5895.6) | 84.1 (19.6-308.4) | 2760.4 (643.4-10011.6) | 85.1 (19.8-308.5) | 0.09 (0.07-0.11) |
| United States Virgin Islands | 24.6 (6.5-96.9) | 62 (16.5-244.6) | 19.1 (4.9-74.5) | 62.9 (16-245.6) | 0.04 (0.03-0.05) |
| Pakistan | 24308 (5381.2-107525.8) | 59.4 (13.1-262.6) | 51058 (10121.4-233634.7) | 55.8 (11.1-255.4) | -0.31 (-0.37--0.25) |
| Guam | 36.2 (9-145.5) | 57.1 (14.2-229.6) | 34.4 (8.7-135.1) | 56.9 (14.3-223.1) | -0.07 (-0.11--0.03) |
| Mauritania | 502.9 (131.1-1960.4) | 65.4 (17.1-255.1) | 1056.4 (282.7-4116.2) | 65.7 (17.6-256.2) | 0.01 (0.01-0.02) |
| Cambodia | 2253.4 (525.5-9744) | 58.1 (13.6-251.3) | 4275.9 (1046.2-18248.1) | 60.1 (14.7-256.6) | 0.08 (0.04-0.11) |
| Singapore | 1005.2 (265.4-4107.6) | 66.6 (17.6-272.2) | 1632.4 (422-6449.3) | 76.7 (19.8-303) | 0.47 (0.34-0.6) |
| Nepal | 4445.2 (1085.8-18975) | 60.9 (14.9-259.8) | 8093.1 (1997.8-33315.6) | 61.9 (15.3-254.8) | 0.06 (0.05-0.06) |
| South Sudan | 1318.2 (367.1-4919.4) | 56.5 (15.7-210.7) | 1950.9 (565.9-6975.8) | 57.1 (16.6-204) | 0.06 (0.04-0.08) |
| Slovakia | 1775.1 (477.6-5961.3) | 86.7 (23.3-291.2) | 1600.3 (427.5-5316.2) | 89.6 (23.9-297.6) | 0.15 (0.12-0.18) |
| Mongolia | 692.6 (165.2-2515.1) | 78.4 (18.7-284.8) | 1123.4 (278.3-3998.6) | 82.9 (20.5-295.1) | 0.18 (0.15-0.2) |
| Italy | 18445.3 (4272.2-70485) | 86.4 (20-330.1) | 14990.1 (3392-59091) | 93.1 (21.1-366.9) | 0.28 (0.21-0.35) |
| Kiribati | 17 (4.3-70.5) | 55.7 (14.1-230.9) | 27.6 (7.1-112.2) | 56.6 (14.6-230) | 0 (-0.02-0.03) |
| Ireland | 1143.2 (264.5-4171.8) | 83.3 (19.3-304.1) | 1417.7 (342.1-5106.9) | 88.2 (21.3-317.6) | 0.25 (0.23-0.27) |
| Andorra | 21.5 (5-77.7) | 85.8 (20.1-310.5) | 22.7 (5.5-81.7) | 89.3 (21.8-322.2) | 0.17 (0.11-0.23) |
| Kenya | 5362.3 (1590.4-20019.6) | 61.3 (18.2-228.7) | 13580.4 (3965.9-48689.2) | 62.7 (18.3-224.7) | 0.1 (0.09-0.11) |
| Mali | 1943.9 (518.6-7802.5) | 65.3 (17.4-262.1) | 5275.8 (1391.1-20427.2) | 64.5 (17-249.9) | -0.02 (-0.03--0.01) |
| Morocco | 8385.8 (2684.6-25305.4) | 80.6 (25.8-243.2) | 12150.3 (3988.3-34765.5) | 83.1 (27.3-237.8) | 0.11 (0.1-0.11) |
| Romania | 7455 (1978.1-25229.6) | 85.8 (22.8-290.3) | 4964.9 (1336.6-16576.5) | 88.4 (23.8-295.1) | 0.17 (0.15-0.19) |
| Zimbabwe | 2491 (685.7-9810.9) | 62.9 (17.3-247.7) | 4012 (1110.9-15213.5) | 64.7 (17.9-245.3) | 0.13 (0.11-0.16) |
| Eswatini | 192.1 (54.6-741.1) | 63.7 (18.1-245.9) | 326.4 (89-1238.8) | 64.6 (17.6-245.3) | 0.05 (0.02-0.09) |
| United States of America | 89955.1 (20777.4-332181.1) | 88.3 (20.4-325.9) | 94372.7 (22031.9-346699.1) | 85.8 (20-315.2) | -0.17 (-0.2--0.14) |
| Turkmenistan | 1223.4 (289.1-4462.8) | 79.6 (18.8-290.2) | 1650.7 (388.9-5935.8) | 81.2 (19.1-291.9) | 0.03 (0.01-0.06) |
| Venezuela (Bolivarian Republic of) | 4849.3 (1255.4-19665.6) | 60.5 (15.7-245.4) | 6646.1 (1804-26476.3) | 62.5 (17-248.9) | 0.09 (0.07-0.11) |
| Marshall Islands | 9.5 (2.3-39.1) | 55.2 (13.6-227.3) | 13.5 (3.5-53.5) | 56.9 (14.7-224.9) | 0.15 (0.12-0.17) |
| Trinidad and Tobago | 306.5 (76.9-1235.5) | 61.1 (15.3-246.3) | 323.8 (82.8-1263.4) | 63.4 (16.2-247.5) | 0.12 (0.09-0.16) |
| Taiwan (Province of China) | 4588.6 (1261.5-17444) | 49.7 (13.7-189.1) | 4104.8 (1174.5-14802.2) | 51.7 (14.8-186.4) | 0.16 (0.15-0.17) |
| Angola | 2490.7 (670.3-9845.4) | 63.3 (17-250.2) | 7182.5 (1942.3-27434.4) | 64.1 (17.3-244.9) | 0.06 (0.06-0.07) |
| Palestine | 600.8 (187.5-1803.7) | 78.5 (24.5-235.6) | 1686.2 (542.2-4899.9) | 80.7 (25.9-234.5) | 0.07 (0.05-0.08) |
| Suriname | 96.5 (24-401.9) | 59.7 (14.8-248.7) | 132.1 (33.8-532.6) | 61.5 (15.7-248) | 0.08 (0.07-0.1) |
| Saint Lucia | 33.6 (8.5-138.3) | 59.8 (15.1-245.9) | 41.9 (10.9-166.9) | 62 (16.1-247.2) | 0.11 (0.1-0.12) |
| Niger | 1795.8 (472.4-7306.7) | 64.6 (17-262.9) | 5181.7 (1357.5-20832.1) | 63.8 (16.7-256.4) | -0.04 (-0.06--0.02) |
| Bahamas | 72.1 (18.4-287.1) | 61 (15.6-243.1) | 93.5 (24.2-370.3) | 62.5 (16.2-247.4) | 0.05 (0.03-0.07) |
| Ethiopia | 9685.5 (2727.8-37943.4) | 51.9 (14.6-203.4) | 20598.6 (5872-78630.1) | 46.4 (13.2-177.1) | -0.53 (-0.63--0.42) |
| Micronesia (Federated States of) | 22 (5.6-89.7) | 55.1 (14-224.5) | 23.5 (5.8-98.1) | 55.7 (13.8-232.1) | 0.03 (0.02-0.04) |
| Lao People's Democratic Republic | 898.9 (216-3906.5) | 58.2 (14-252.7) | 1881.1 (447.8-7925.3) | 59.7 (14.2-251.7) | 0.07 (0.05-0.09) |
| Belarus | 4103.2 (1161.3-13222.9) | 103.8 (29.4-334.6) | 3250.7 (944.4-10377.6) | 106.6 (31-340.2) | 0.08 (0.03-0.13) |
| Malta | 119 (29.3-430.4) | 86.3 (21.3-312.1) | 121.5 (29.3-443) | 88.1 (21.2-321.1) | 0.05 (0-0.1) |
| Samoa | 36.3 (9.1-150.6) | 54.4 (13.6-225.7) | 46 (11.4-186.3) | 55.3 (13.7-223.9) | 0.04 (0.01-0.07) |
| Brazil | 41761.1 (9396.7-178835.7) | 66.6 (15-285) | 58059.3 (13736.8-237707) | 67.4 (16-276) | 0.01 (-0.06-0.08) |
| Dominica | 17.4 (4.5-71.8) | 59.7 (15.5-246.1) | 15.8 (4.1-62.3) | 61.4 (16-241.6) | 0.05 (0.03-0.08) |
| Latvia | 985.3 (277.5-3161.8) | 103.3 (29.1-331.4) | 593.5 (166.9-1910.7) | 105.7 (29.7-340.4) | 0.07 (0.03-0.11) |
| Uzbekistan | 6808 (1582.7-24671.1) | 79.3 (18.4-287.2) | 11602.1 (2735.4-41617.5) | 81.3 (19.2-291.7) | 0.06 (0.03-0.08) |
| Philippines | 16119.7 (3981.4-66832.3) | 62.1 (15.3-257.4) | 29269.9 (7290.6-119634.5) | 63.5 (15.8-259.4) | 0.06 (0.06-0.07) |
| Luxembourg | 136.1 (35-468.9) | 92.2 (23.7-317.6) | 200.2 (53.4-680.6) | 93.8 (25-318.9) | 0.09 (0.03-0.15) |
| Mauritius | 300.6 (72.8-1264.9) | 60.2 (14.6-253.2) | 285.4 (68.2-1176.8) | 60.9 (14.5-251.1) | 0.05 (0.04-0.06) |
| Paraguay | 955.6 (216-4095.7) | 61 (13.8-261.5) | 1841.4 (422.7-7808.8) | 62 (14.2-262.7) | 0.05 (0.03-0.06) |
| Benin | 1119.3 (304.2-4408.1) | 65.7 (17.9-258.9) | 3199.2 (845.4-12677.4) | 65.5 (17.3-259.4) | -0.03 (-0.03--0.02) |
| Malaysia | 4383.6 (1040.1-18287.9) | 59.2 (14-246.8) | 8356.5 (1921.2-34824.5) | 60.6 (13.9-252.7) | 0.05 (0.04-0.07) |
| Ecuador | 2076.9 (639.1-8044.7) | 50.4 (15.5-195.2) | 3858.6 (1145.1-15206) | 53.5 (15.9-210.9) | 0.27 (0.23-0.31) |
| Monaco | 8.1 (1.9-29) | 88.1 (21.1-317.3) | 8.1 (2-29.2) | 87.4 (21.2-314.3) | -0.07 (-0.13--0.02) |
| Qatar | 193.4 (60.8-557.4) | 81.8 (25.7-235.8) | 1405.8 (436-4052.4) | 81.8 (25.4-235.9) | -0.05 (-0.08--0.03) |
| El Salvador | 1237.2 (326.4-5115.6) | 59.3 (15.6-245.2) | 1577.8 (400.1-6342.8) | 61.2 (15.5-246.2) | 0.1 (0.08-0.11) |
| Armenia | 1171.3 (291.2-4140) | 81.5 (20.3-288) | 937.8 (231.7-3382) | 84.1 (20.8-303.3) | 0.06 (0-0.12) |
| Iran (Islamic Republic of) | 19956.5 (6328.8-57578.5) | 88.3 (28-254.8) | 35741.4 (10988.3-105337.1) | 100.4 (30.9-295.8) | 0.42 (0.39-0.46) |
| Cuba | 2937.8 (742.3-12055.4) | 60.4 (15.3-247.8) | 2262.2 (577.6-9044.2) | 62.4 (15.9-249.4) | 0.03 (-0.02-0.08) |
| Nigeria | 23471 (6340.5-93940) | 68.9 (18.6-275.8) | 58227.5 (15599.9-219404.6) | 69.4 (18.6-261.6) | 0 (-0.01-0.02) |
| Myanmar | 9913.5 (2361.2-42584.9) | 58.5 (13.9-251.4) | 13314.6 (3135.5-55678) | 60.3 (14.2-252) | 0.1 (0.1-0.11) |
| Malawi | 2075.6 (587.1-7745.6) | 57.4 (16.2-214.2) | 4392.6 (1239.2-15887.5) | 57.6 (16.3-208.4) | 0.06 (0.05-0.07) |
| Oman | 643.2 (209.2-1914.8) | 80.1 (26.1-238.5) | 2128.4 (671.1-6102) | 83 (26.2-238) | 0.13 (0.11-0.16) |
| Congo | 596.8 (161.4-2307.1) | 62.9 (17-243.2) | 1380.6 (368.9-5170.1) | 65.6 (17.5-245.5) | 0.18 (0.16-0.19) |
| Madagascar | 2605.9 (757.2-9526.4) | 57.6 (16.7-210.5) | 6381.9 (1829.3-23698.8) | 58.4 (16.7-216.9) | 0.05 (0.05-0.06) |
| Papua New Guinea | 907 (227.8-3821.9) | 54.8 (13.8-230.9) | 2287.6 (585.5-9468.8) | 55.9 (14.3-231.4) | 0.06 (0.05-0.07) |
| Indonesia | 48928.3 (12048.3-202509) | 62.7 (15.4-259.4) | 68569.6 (16971.8-273844.8) | 64.7 (16-258.5) | 0.13 (0.12-0.14) |
| New Zealand | 1038.1 (265.4-3770.1) | 75.1 (19.2-272.8) | 1072.3 (281.7-3752.9) | 76 (20-266.1) | -0.02 (-0.05-0.02) |
| Bolivia (Plurinational State of) | 1336.4 (379.7-5478.6) | 54.2 (15.4-222.4) | 2671.5 (779.1-10667.1) | 55.3 (16.1-220.6) | 0.06 (0.05-0.08) |
| Sao Tome and Principe | 27.5 (7.2-109.9) | 64 (16.7-256.1) | 57.6 (15.5-224.2) | 66.4 (17.8-258.1) | 0.16 (0.14-0.18) |
| Antigua and Barbuda | 15.8 (4-62.5) | 61.4 (15.7-242.2) | 21.6 (5.6-84.3) | 62.4 (16.1-243.5) | 0.02 (-0.01-0.05) |
| Belgium | 3192.4 (754.3-11705.7) | 85.8 (20.3-314.7) | 3048.9 (718.3-11054.9) | 86.9 (20.5-315.3) | -0.01 (-0.03-0.01) |
| Nauru | 2.3 (0.6-9.3) | 56.6 (13.9-229.2) | 2.6 (0.6-10.5) | 56.4 (13.9-230.3) | -0.01 (-0.01-0) |
| Burkina Faso | 2062.9 (555.2-8149) | 64.6 (17.4-255.2) | 5594.9 (1454.8-22077.7) | 65.5 (17-258.6) | 0.07 (0.06-0.08) |
| Bosnia and Herzegovina | 1652.3 (449.1-5583) | 85.5 (23.2-289.1) | 911.4 (244.1-3038.7) | 87.6 (23.5-292) | 0.05 (0.03-0.08) |
| Bulgaria | 2575.3 (672.7-8730.6) | 86.5 (22.6-293.3) | 1762.4 (474.1-5854.4) | 89.2 (24-296.4) | 0.14 (0.13-0.16) |
| Democratic Republic of the Congo | 8952.7 (2419.8-35443.3) | 62.6 (16.9-247.9) | 22100.5 (5900.7-87222.3) | 63.2 (16.9-249.3) | 0.02 (0.01-0.04) |
| Norway | 1361.3 (295.5-5243.7) | 85.1 (18.5-327.8) | 1566.7 (344.3-6066.5) | 89 (19.6-344.7) | 0.07 (0.04-0.1) |
| Algeria | 8078.8 (2594-24191.9) | 79.8 (25.6-238.9) | 14564.2 (4773.6-41625.9) | 84.9 (27.8-242.7) | 0.21 (0.19-0.24) |
| Slovenia | 668.2 (181.4-2261) | 87.2 (23.7-295.1) | 530 (142.1-1778.6) | 89.2 (23.9-299.4) | 0.1 (0.08-0.12) |
| Portugal | 3171.4 (745.7-11561.9) | 83.8 (19.7-305.4) | 2670.6 (652.2-9426.8) | 87.8 (21.4-309.8) | 0.23 (0.2-0.27) |
| Chile | 3961.6 (1001.3-14487.8) | 69.2 (17.5-253) | 4942 (1227.7-16850.3) | 72.6 (18-247.6) | 0.12 (0.1-0.14) |
| Solomon Islands | 69.8 (16.9-296.3) | 54.4 (13.1-230.6) | 146 (36.3-604.9) | 55.8 (13.9-231.2) | 0.13 (0.12-0.15) |
| Cabo Verde | 84.7 (22.5-332.6) | 64.8 (17.3-254.7) | 169.6 (45.4-665.3) | 68.2 (18.2-267.5) | 0.11 (0.06-0.15) |
| Czechia | 3206.2 (848-10873.4) | 86.3 (22.8-292.6) | 2723.2 (724.8-9057.3) | 89.4 (23.8-297.5) | 0.2 (0.17-0.22) |
| Netherlands | 5061.9 (1040.7-20433.8) | 84 (17.3-338.9) | 4430.4 (929.3-18336.2) | 84.2 (17.7-348.3) | 0.09 (0.06-0.12) |
| Senegal | 1779.4 (475.6-7019.5) | 64.5 (17.2-254.5) | 4005.6 (1069.4-15980.8) | 65.3 (17.4-260.7) | 0.05 (0.05-0.05) |
| Northern Mariana Islands | 13.7 (3.4-53.9) | 58.5 (14.7-229.9) | 7.6 (1.8-31.1) | 54.3 (13.3-223.4) | -0.28 (-0.41--0.15) |
| Tunisia | 2786.5 (914.9-8277.2) | 80.9 (26.5-240.2) | 3786.9 (1247.7-10662.4) | 85.5 (28.2-240.6) | 0.2 (0.18-0.21) |
| Hungary | 3224.8 (858.6-10796.8) | 87.2 (23.2-292) | 2523.1 (683.5-8449.1) | 88.5 (24-296.5) | 0.14 (0.11-0.17) |
| Sierra Leone | 897 (249.6-3592) | 65.8 (18.3-263.3) | 2290.5 (608.9-9063.4) | 65.5 (17.4-259.2) | -0.05 (-0.06--0.03) |
| Guyana | 202.5 (51.6-836.6) | 59.5 (15.2-245.9) | 195.4 (51-798.2) | 60.4 (15.8-246.8) | 0.01 (-0.01-0.04) |
| Central African Republic | 660.2 (182.3-2585.5) | 62.9 (17.4-246.3) | 1336.3 (369.4-5263.8) | 63.3 (17.5-249.5) | 0.03 (0.02-0.03) |
| Germany | 25369.1 (6029.5-90971.8) | 85.4 (20.3-306.3) | 22288 (5251.5-79179.2) | 87.5 (20.6-310.7) | 0.07 (0.02-0.12) |
| Kuwait | 691.9 (219.7-2040.7) | 79.3 (25.2-234) | 1757.9 (562.2-4924.4) | 84.2 (26.9-235.9) | 0.19 (0.15-0.23) |
| Mozambique | 2645.4 (765.5-10018.9) | 57.8 (16.7-219) | 6463.6 (1820.5-23360.7) | 57.6 (16.2-208.1) | 0.01 (0-0.02) |
| Grenada | 20 (5.2-81.6) | 60 (15.5-244.9) | 24.8 (6.3-99.1) | 60.8 (15.5-242.7) | 0.05 (0.04-0.06) |
| Saudi Arabia | 5214.6 (1668-15360.6) | 77.8 (24.9-229.1) | 14995.1 (5029.2-41751.6) | 81.8 (27.5-227.9) | 0.17 (0.14-0.19) |
| Colombia | 8563.4 (2139.1-34501.8) | 60.9 (15.2-245.2) | 11835.6 (3065.6-46758.8) | 61.9 (16-244.5) | 0.04 (0.03-0.05) |
| Russian Federation | 68238.1 (21334.4-212062) | 117.3 (36.7-364.4) | 57972.2 (18294.9-179673.8) | 120.7 (38.1-374.2) | 0.23 (0.19-0.28) |
| Cameroon | 2472.2 (647.3-9747.1) | 65.1 (17-256.6) | 7952.3 (2110.9-30868.2) | 65.8 (17.5-255.5) | 0.05 (0.03-0.07) |
| Syrian Arab Republic | 3804.9 (1241.3-11276.1) | 79 (25.8-234.1) | 4426.6 (1420.1-13224.6) | 80.4 (25.8-240.1) | 0.12 (0.09-0.15) |
| Lithuania | 1337.7 (340.3-4659.4) | 96 (24.4-334.4) | 823.3 (208.9-2853.3) | 98 (24.9-339.8) | -0.01 (-0.04-0.02) |
| Albania | 1199.3 (311.2-4094.5) | 84.6 (21.9-288.7) | 829.4 (213.5-2798.1) | 85.5 (22-288.4) | -0.02 (-0.04-0) |
| Chad | 1360.6 (366.2-5506.6) | 64.8 (17.4-262.4) | 3722.6 (992.2-14972) | 64.3 (17.1-258.6) | -0.01 (-0.02--0.01) |
| Austria | 2742 (704.4-9404.2) | 91.4 (23.5-313.4) | 2636.6 (680.1-9097.1) | 93.7 (24.2-323.4) | 0.04 (-0.03-0.11) |
| Rwanda | 1577.3 (437.4-5840.3) | 57.7 (16-213.8) | 3172.2 (918.2-11465.8) | 58.6 (17-211.8) | 0.08 (0.04-0.12) |
| Belize | 43.4 (11.1-181.9) | 59.3 (15.1-248.4) | 108.9 (27.4-439.5) | 60.8 (15.3-245.4) | 0.08 (0.07-0.09) |
| Finland | 1580 (373.7-5666.4) | 87 (20.6-312.2) | 1450.4 (347.7-5321.5) | 86.7 (20.8-318.1) | -0.04 (-0.07--0.02) |
| Egypt | 18118.9 (5674-54742.4) | 82.7 (25.9-249.8) | 34829.7 (10866.3-102861.1) | 84.9 (26.5-250.8) | 0.1 (0.08-0.12) |
| Vanuatu | 32.5 (8.1-134) | 55.4 (13.9-228.6) | 66.2 (16.8-271.8) | 56.1 (14.2-230.2) | 0.04 (0.03-0.04) |
| Thailand | 15430.5 (3673.4-64824.9) | 59.6 (14.2-250.3) | 15042.5 (3707.3-61520.3) | 62 (15.3-253.5) | 0.12 (0.1-0.14) |
| Togo | 889.2 (238.6-3512) | 64.9 (17.4-256.2) | 2117.7 (576.5-8348.4) | 66.4 (18.1-261.9) | 0.1 (0.1-0.11) |
| Spain | 12450.1 (3077.3-44871.6) | 83.9 (20.7-302.6) | 11994.7 (3020.5-42196.4) | 92.4 (23.3-325.1) | 0.59 (0.51-0.66) |
| Peru | 4881.7 (1351.6-19995.2) | 55 (15.2-225.5) | 7905.2 (2150.6-30878.5) | 58 (15.8-226.5) | 0.2 (0.17-0.22) |
| Niue | 0.5 (0.1-1.8) | 55.9 (13.7-228) | 0.3 (0.1-1.3) | 57.1 (14.8-226.2) | 0.08 (0.07-0.09) |
| Turkey | 19707.8 (6667.1-55578) | 79.7 (27-224.8) | 28394.1 (9746.9-76952.7) | 83.4 (28.6-226.1) | 0.07 (0.04-0.11) |
| Tonga | 20 (4.9-82.7) | 54.3 (13.4-224.3) | 21.6 (5.4-87.2) | 56.1 (14.1-226.3) | 0.12 (0.11-0.14) |
| Gambia | 246 (65.1-980.6) | 65.1 (17.2-259.4) | 614.4 (161-2403.8) | 65.2 (17.1-255.2) | 0.02 (0.02-0.03) |
| Sweden | 2460.1 (552-9409.8) | 83.8 (18.8-320.6) | 2653.2 (576.7-10054.1) | 83.1 (18.1-314.8) | -0.09 (-0.11--0.07) |
| Ukraine | 20802 (5992.6-67201.8) | 109.6 (31.6-353.9) | 16310.9 (4641.9-51921.3) | 112.9 (32.1-359.5) | 0.12 (0.07-0.17) |
| Estonia | 587.2 (163.5-1868.5) | 103.4 (28.8-329) | 424.7 (119.6-1374.3) | 105.6 (29.7-341.6) | 0.07 (0.04-0.1) |
| Cyprus | 259.7 (61-949.2) | 84.5 (19.8-308.6) | 450.9 (108-1633.3) | 90.1 (21.6-326.3) | 0.16 (0.12-0.2) |
| Saint Kitts and Nevis | 10.5 (2.7-43.2) | 60.8 (15.7-248.8) | 14.2 (3.7-56.6) | 62.7 (16.3-249.8) | 0.09 (0.08-0.1) |
| Palau | 4 (1-15.8) | 56.7 (13.9-226.6) | 3.5 (0.9-13.9) | 57.9 (15.1-227.8) | 0.02 (-0.02-0.05) |
| Azerbaijan | 2561.9 (628.1-9135.3) | 80.6 (19.7-287.2) | 3549.6 (874.7-12618.4) | 83.2 (20.5-295.7) | 0.02 (-0.02-0.06) |
| United Arab Emirates | 787.2 (249.5-2262.3) | 82.2 (26-236.2) | 3929.1 (1186.1-10997.3) | 87.6 (26.5-245.3) | 0.19 (0.13-0.26) |
| Equatorial Guinea | 94.6 (26.2-375.5) | 63.1 (17.5-250.3) | 407.7 (107.4-1556.1) | 62.6 (16.5-239.1) | -0.03 (-0.04--0.02) |
| Maldives | 46.5 (11-203.1) | 57.2 (13.5-249.9) | 156.8 (37.8-641) | 62.2 (15-254.3) | 0.22 (0.18-0.27) |
| Canada | 9252.8 (2069.5-34538.7) | 83.2 (18.6-310.6) | 9548.6 (2195-35167.9) | 83.2 (19.1-306.4) | -0.07 (-0.09--0.04) |
| Montenegro | 215.4 (57.5-730.4) | 85.9 (22.9-291.3) | 184.5 (48.1-619.3) | 87.5 (22.8-293.7) | 0.07 (0.06-0.08) |
| C么te d'Ivoire | 3054.1 (817.1-12139.8) | 64.6 (17.3-256.6) | 7143.5 (1860.4-28219.8) | 66.3 (17.3-262.1) | 0.11 (0.09-0.13) |
| United Republic of Tanzania | 5531.7 (1628.5-20182) | 57.1 (16.8-208.5) | 12889.2 (3682.2-46353.3) | 58.4 (16.7-209.9) | 0.1 (0.09-0.11) |
| Somalia | 1439.5 (422.4-5567.1) | 57 (16.7-220.5) | 4456.4 (1260.6-16914.6) | 56.8 (16.1-215.7) | 0.09 (0.06-0.13) |
| Croatia | 1581.9 (421.1-5416.4) | 87.2 (23.2-298.6) | 1137.8 (301.8-3782.9) | 88.3 (23.4-293.5) | -0.04 (-0.12-0.04) |
| Bahrain | 212.2 (68.5-603.9) | 82.6 (26.7-235.2) | 532.1 (172.9-1491.1) | 86.1 (28-241.1) | 0.12 (0.07-0.16) |
| Puerto Rico | 871.4 (223.7-3476.1) | 61.6 (15.8-245.8) | 702.6 (183.8-2731.4) | 62.5 (16.4-243.1) | 0.05 (0.04-0.05) |
| Jordan | 1204.8 (375.6-3645.2) | 78 (24.3-236) | 4078 (1324.6-12023.5) | 81.4 (26.4-239.9) | 0.15 (0.12-0.17) |
| Ghana | 3761.8 (1017.6-14853.9) | 65.4 (17.7-258.4) | 9135 (2432.8-35095.1) | 67.1 (17.9-257.8) | 0.07 (0.06-0.08) |
| Greece | 3188.5 (766.1-11619.2) | 84.8 (20.4-309.2) | 2587 (638.6-9350.8) | 89 (22-321.5) | 0.21 (0.2-0.23) |
| Yemen | 3663.9 (1198.5-11123) | 79.5 (26-241.2) | 10511.8 (3342.1-30226.8) | 80.8 (25.7-232.3) | 0.12 (0.08-0.15) |
| Guatemala | 1722.2 (452-7164.1) | 59.4 (15.6-246.9) | 4802.7 (1242.7-19264.9) | 60.6 (15.7-243.3) | 0.08 (0.07-0.09) |
| Seychelles | 18.5 (4.5-77.9) | 59.2 (14.3-249.3) | 23.8 (6-95.7) | 61.6 (15.5-248) | 0.1 (0.08-0.12) |
| Guinea-Bissau | 243.4 (66-980.1) | 64.8 (17.6-261.1) | 530.4 (144.6-2083.9) | 66 (18-259.3) | 0.06 (0.04-0.09) |
| Costa Rica | 783.3 (199.8-3162.1) | 61 (15.6-246.3) | 1199.6 (317-4779.4) | 62.6 (16.5-249.5) | 0.06 (0.04-0.08) |
| Zambia | 1949.8 (555.8-7233.9) | 64.3 (18.3-238.5) | 4980.4 (1511.9-17551.5) | 65.6 (19.9-231.3) | 0.09 (0.07-0.11) |
| United Kingdom | 18342.1 (4466.2-65760.4) | 87.9 (21.4-315.3) | 19726.3 (4824.4-70183.3) | 90.2 (22.1-320.9) | 0 (-0.04-0.03) |
| Denmark | 1677.6 (394.7-6238.9) | 87.9 (20.7-327) | 1539.8 (347-5703.7) | 85.4 (19.3-316.4) | -0.13 (-0.16--0.11) |
| Switzerland | 2040.6 (531.8-6891.4) | 77.4 (20.2-261.5) | 2342.7 (601.6-8207.4) | 84.4 (21.7-295.8) | 0.22 (0.15-0.3) |
| Republic of Moldova | 1812.4 (490.7-5741.9) | 103.9 (28.1-329.3) | 1389.7 (401.2-4421.3) | 105.9 (30.6-336.9) | 0.07 (0.02-0.13) |
| France | 19854.5 (5087.1-67517.9) | 90.3 (23.1-307) | 18139.9 (4723.7-61519.3) | 91.2 (23.8-309.4) | 0.03 (0.01-0.04) |
| Gabon | 242.9 (66.1-946.3) | 63.6 (17.3-247.9) | 485.8 (131.3-1834.2) | 65.3 (17.7-246.5) | 0.08 (0.07-0.08) |
| Djibouti | 114.4 (33.7-427.9) | 57 (16.8-213.2) | 303.7 (85.2-1093) | 60.2 (16.9-216.5) | 0.21 (0.19-0.23) |
| Brunei Darussalam | 88.3 (20.6-354.6) | 71.6 (16.7-287.6) | 147.9 (34.8-587.7) | 73.3 (17.3-291.3) | 0.04 (0.03-0.06) |
| American Samoa | 11.3 (2.9-46.4) | 56 (14.3-229.4) | 11.7 (3-47.4) | 55.4 (14-224.9) | -0.06 (-0.1--0.02) |
| Sri Lanka | 4408.2 (1092.5-18608.8) | 59.6 (14.8-251.6) | 4903.7 (1208.4-19910.2) | 61 (15-247.7) | 0.1 (0.09-0.11) |
| Burundi | 1205 (340.2-4438.9) | 58.2 (16.4-214.3) | 2677.8 (754.1-9855.6) | 57.9 (16.3-213.3) | -0.04 (-0.09-0.01) |
| Iraq | 5199.8 (1648-15691.6) | 79.1 (25.1-238.7) | 15151.9 (4827-44119.5) | 81.1 (25.9-236.3) | 0.08 (0.06-0.09) |
| Dominican Republic | 1847.6 (467.3-7594.1) | 60.1 (15.2-247.1) | 2763.1 (723.1-11128.6) | 61.4 (16.1-247.4) | 0.05 (0.04-0.06) |
| Guinea | 1423.5 (383.4-5729.2) | 65.9 (17.8-265.3) | 3170.4 (849.6-12444.4) | 65.7 (17.6-258) | -0.01 (-0.03-0.01) |
| Afghanistan | 2940.3 (923.8-9049.9) | 75.9 (23.9-233.7) | 11775.6 (3790-35564.4) | 78.1 (25.2-236) | 0.07 (0.01-0.13) |
| North Macedonia | 699 (187.5-2362.8) | 86 (23.1-290.8) | 682.5 (185.3-2247.6) | 88.3 (24-290.9) | 0.08 (0.06-0.1) |
| Honduras | 1029.1 (258.9-4296.6) | 59.6 (15-249) | 2558.6 (662.4-10298.6) | 60.5 (15.7-243.7) | 0.07 (0.06-0.08) |
| Bangladesh | 24659 (5638.3-109498.1) | 57.8 (13.2-256.5) | 40338.8 (9166.4-169343.8) | 59.5 (13.5-249.8) | 0.11 (0.1-0.11) |
| Tokelau | 0.3 (0.1-1.3) | 56 (13.8-228.6) | 0.3 (0.1-1.1) | 56.5 (14.3-225.8) | 0.03 (0.01-0.05) |
| Lesotho | 437.2 (118.2-1669.2) | 63.4 (17.1-242) | 615.4 (168.2-2340) | 64.8 (17.7-246.3) | 0.09 (0.06-0.12) |
| Uganda | 3630.7 (1061.7-13944) | 56.6 (16.5-217.3) | 9291.4 (2647.7-33299.4) | 57.5 (16.4-206.1) | 0.06 (0.06-0.07) |
| Argentina | 8631.2 (2188.5-31395.2) | 70.6 (17.9-256.9) | 12576.3 (3170-43880.6) | 72.2 (18.2-251.9) | 0.09 (0.07-0.11) |
| Tuvalu | 2.1 (0.5-8.4) | 57.2 (14.9-232.7) | 2.7 (0.7-10.8) | 56 (14.1-226.5) | -0.1 (-0.11--0.09) |
| Barbados | 67.7 (17.5-268.5) | 62 (16.1-245.9) | 62 (16.6-240.6) | 62.7 (16.8-243.2) | 0.01 (0-0.03) |
| San Marino | 7.6 (1.8-27.6) | 83.7 (19.5-304.4) | 8.9 (2.1-32.5) | 86.9 (20.6-316.2) | 0.15 (0.08-0.21) |
| Comoros | 99.1 (28.2-359.4) | 57.4 (16.3-208.3) | 175.2 (51.8-638.6) | 59.1 (17.5-215.6) | 0.1 (0.1-0.11) |
| Botswana | 326.1 (90.8-1234.8) | 63.4 (17.6-240) | 702.2 (194.2-2650) | 66.6 (18.4-251.4) | 0.18 (0.15-0.21) |
| Sudan | 6090.7 (2012-18554) | 79.9 (26.4-243.3) | 14020.8 (4568.9-41227.5) | 81.2 (26.5-238.9) | 0.07 (0.06-0.07) |

Abbreviations: EAPC, estimated annual percentage change; SDI, Sociodemographic Index; UI, uncertainty interval.
